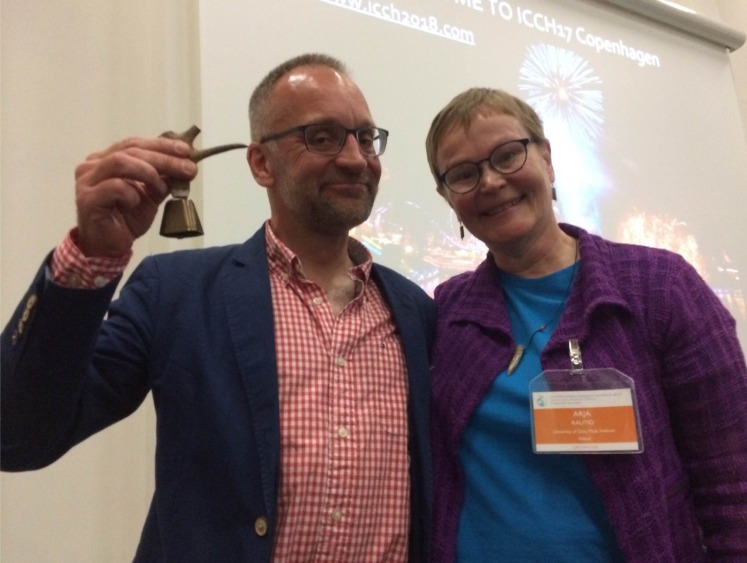# Abstracts from the 16th International Congress on Circumpolar Health

**DOI:** 10.3402/ijch.v75.33200

**Published:** 2016-11-04

## Preface

The city of Oulu (Finland) has been chosen twice as the main location of the International Congress on Circumpolar Health (ICCH). The 2nd ICCH was organized in June 21–24, 1971 by the Nordic Council of Arctic Medical Research, and the 16th ICCH in June 8–12, 2015 by the University of Oulu as the main local organizer. On the basis of the Proceedings of the 2nd ICCH the topics have been very similar to those we have addressed now, 44 years later: environmental aspects, human adaptability to darkness and cold, environmental induced diseases, psychiatry, genetics, nutrition, odontology, ophthalmology, and infections. In addition, the 16th ICCH has highlighted some new, current topics including indigenous health, mental health, contaminants, educational issues and ethical questions in health research.

During the conference week there were organized 36 scientific sessions with 10 keynote speakers and almost 300 oral and poster presentations were made. The ICCH16 was preceded by an intensive Summer School for PhD students and young researchers. I hope that all these activities contributed to the building of new networks and collaboration for the future of Arctic health and well-being. Many of the current challenges affecting human health and well-being are not only of Arctic, but also of a global nature including climate change, changes in economics, and migration. We as researchers and educators should be ready to participate in and contribute to processes aimed at solving these challenges.

It has been a great pleasure for me to be in the lead in arranging this Congress together with colleagues of the Nordic Society of Circumpolar Health and the International Union of Circumpolar Health. Without the financial support of our main sponsors, the University of Oulu, The Finnish Ministry of Health and Welfare, the Federation of Finnish Learned Societies, and the contribution of our competent and enthusiastic participants, the ICCH16 would not have been possible to arrange. I would like to thank the Organizing Committee and all volunteers for the great work and also all participants for coming to Oulu. It was you who made the Congress a wonderful success!

Arja Rautio, MD, PhD

Chair of the Organizing Committee

ICCH16

Click here to see a video from the conference.

**Figure:**
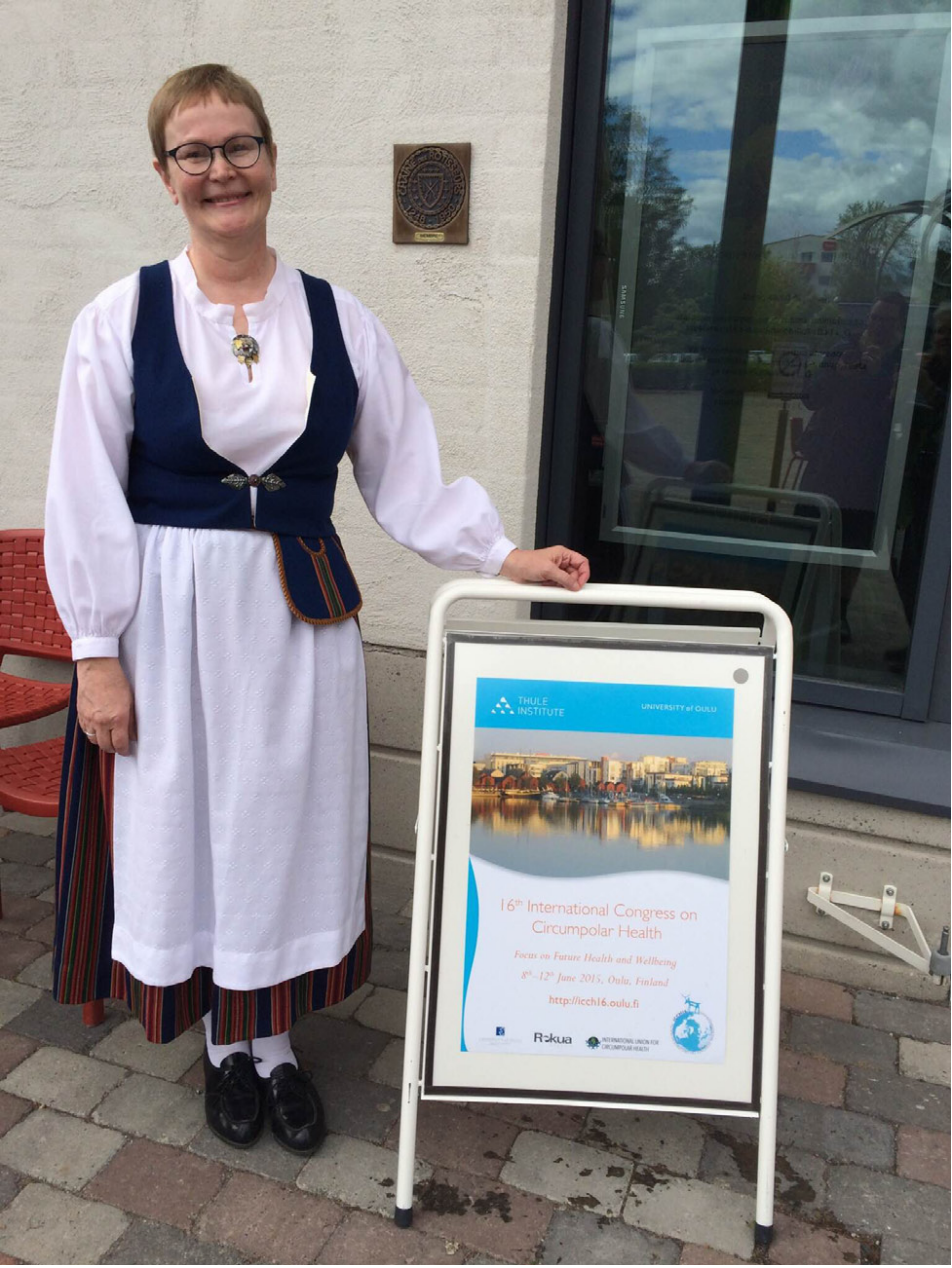


## President’s Reflections

It was a very sunny beginning of the ICCH16 in June in Oulu, Finland. And even if the clouds gathered above the city later in the week the sunny mood stayed with all through the conference. It was a pleasure indeed to on behalf of the Nordic Society of Circumpolar Health welcome all the 350 participants from 12 countries to the Nordic countries and then in particular Finland and beautiful Oulu. The venue was an old hospital transformed into a modern conference area but with the charm kept intact. Later in the week after three intense days of talks and poster-sessions we traveled by bus an hour to a conference- and spa-hotel in Rokua world heritage Geopark, where the lively and fun conference dinner with dance half the night was held and the last scientific session before waving goodbye.

The Nordic Countries have a well established cooperation in among many institutions, the Nordic Council of Ministers, the official inter-governmental body for co-operation in the Nordic Region. We also have Nordforsk, an organization that facilitates and provides funding for Nordic research cooperation and research infrastructure.

I was pleased to say that the well functioning collaboration in the Nordic region had also been a signum of the preparations for ICCH16. The country representatives were involved along the whole process so without doubt ICCH16 was a product of efforts of us all in Norden but surely perfectly executed by Professor Arja Rautio and her team in Oulu.

The Nordic organizations mentioned also have collaborations with our closest neighbors as well as good connections with countries on the other side of the pond and on the islands in between. And our communication with our colleagues from the other side of the globe worked just fine; we asked for input, we received it and we paid attention to it.

Why had we then met up in northern Finland? We were there because the North matters. And the change occurring in this part of the world is outpacing our ability to understand it; for better and for worse, opportunities and challenges are growing. The fast and widespread climate change requires large, international bodies to cooperate, also in the area of health and how it is affected. And we, researchers, we cooperate. We study, analyze, publish and meet to discuss and spread our results and to get reenergized. The mindset to solve northern problems are more likely to be found here than further south. And there are not only problems, but also opportunities – there are stories to be told, and facts to be shown to the world, and shared.

We also teach, to have knowledge passed on but even more important – to have it enlarged and renewed, to stimulate creativity and respect for each others’ insights and knowledge. And also to enjoy each others company.

So during the week science was presented and discussed, new themes presented, teaching was occurring, books were launched and many, many discussions took place. A number of societies managed to squeeze in annual meetings and other satellite-meetings during the week. A summer school was held with 18 students and 6 mentors/faculty on the topics “Community Based Participatory Research Principles and Practices in the North” and “Healthy Populations in the Arctic”. The One Health concept launched from Alaska held a symposium and presented plans for the future.

We did enjoy a week full of knowledge, teaching, presentations, discussions, and laughs! And we look forward to ICCH17 in Copenhagen 2018.

Professor Birgitta Evengard

President, Nordic Society of Circumpolar Health, and Chair

ICCH16

## TABLE OF CONTENTS

**Oral sessions**

*Tuesday June 9th 2015*

Plenary Session

One Health Symposium

Infectious Diseases I

Infectious Diseases II

AMAP Report

Environmental Contaminants I

Environmental Contaminants II

Indigenous Health I: “Health Status Overview”

Indigenous Health II: “Mental Health and Bereavement”

Indigenous Health III: “Life Spectrum and Relationships”

Working in the Circumpolar Regions: Accidents and Injuries (Session I)

Working in the Circumpolar Regions: Accidents and Injuries (Session II)

Environmental Health

Women’s Health and Wellbeing

Community Driven Research

Nature and Health

*Wednesday June 10th 2015*

Plenary Session

Infectious Diseases III

Educational Programs

Development of Health Care I

Mental Wellbeing in the North I

Indigenous Health IV: “Food Insecurity, Obesity and Diabetes”

Indigenous Health V: “Health systems”

Population Health I

Population Health II

Environmental Contaminants III

*Thursday June 11th 2015*

Plenary Session

Improved Health Knowledge in the Arctic I

Improved Health Knowledge in the Arctic II

Mental Wellbeing in the North II

Mental Wellbeing in the North III

Disease Profiles in the Changing Climate

Population Health III

Indigenous Health VI: “Health status overview (2)”

Smart Technology (workshop)

Sexually Transmitted Infections (STIs)

*Friday July 12th 2015*

Plenary Session

Development of Health Care II

Smart Technology

Living in the Arctic

Helicobacter Pylori

**Poster sessions**

*Tuesday June 9th 2015*

A. Infectious Diseases

B. Environmental Contaminants

C. Indigenous Health

D. Working in the Circumpolar Regions: Accidents and Injuries

E. Environmental Health

F. Women’s Health and Wellbeing

G. Community Driven Research

H. Nature and Health

I. Educational Programs

*Wednesday June 10th 2015*

J. Sexually Transmitted Infections (STIs)

K. Development of Health Care

L. Mental Wellbeing in the North

M. Population Health

N. Disease Profiles in the Changing Climate

O. Health Monitoring

P. Smart Technology

Q. Living in the Arctic

**Figure:**
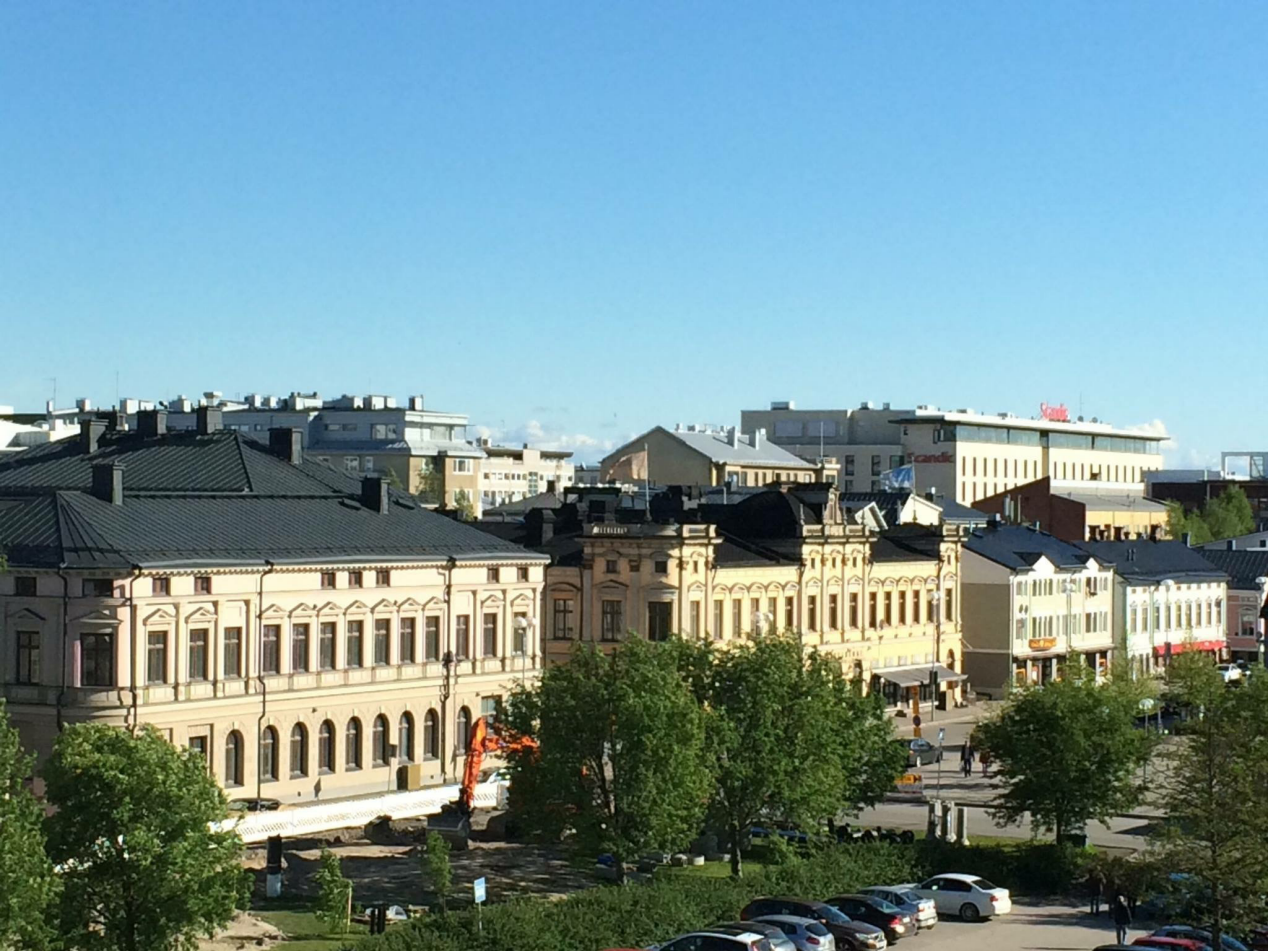


## ORAL SESSIONS Tuesday June 9th 2015

### Plenary Session

#### The AMAP HHAG 2015 report

##### Jon Øyvind Odland
AMAP HHAG, Norway, jon.oyvind.odland@uit.no

###### 

The AMAP 2015 Human Health report is presented as an update of the previous full human health assessment report in 2009. The objectives of the report are to report on recent AMAP human health-related work to fulfill requests from the Arctic Council Ministerial Meeting in Kiruna in 2013 to AMAP regarding the health of Arctic peoples in relation to pollution, climate change and combined effects of stressors and, in addition, to fulfill obligations to UNEP and the Stockholm Convention on human health-related issues [on mercury for UNEP and on persistent organic pollutants (POPs) for the Stockholm Convention]. The report provides further information and policy relevant scientific recommendations for Arctic governments in their efforts to take remedial and preventive actions relating to contaminants. In addition, for the first time, this report addresses the effects of multiple stressors on small remote communities. Most of these are highly dependent on local wildlife resources for food, and are also highly impacted by the climate regime shift.

- Human exposure to most POPs and mercury is declining across some parts of the Arctic, but remains high among certain populations. Even in some regions where levels of contaminants have declined significantly, current levels of exposure are still a concern.
- Newer contaminants can be transported to the Arctic, and may still be in use and not yet internationally regulated. Compounds such as certain brominated flame retardants and perfluorinated contaminants (PFCs) have been recently measured in human tissues from populations living in the Arctic.
- Development of dietary advice can be complex due to varying contaminant levels in different traditional foods and different areas of the Arctic, limited access to, or choice of, imported foods in remote communities and shifting food preferences among Arctic populations.
- The ongoing cohort studies provide increasing evidence of cardiovascular effects and neurodevelopmental effects from methylmercury. In addition, recent data point out a reduced vaccine response associated with PFCs in children.
- Interactions between climate change and contaminant transport and behavior have the potential to change human exposure in the Arctic significantly. Current understanding is inadequate to determine the likelihood and magnitude of the health impacts of exposure changes.

Details of the report will be further elaborated by the different chapter leads.

## Impacts of environmental change on food security in the Canadian Arctic

### Myriam Fillion, Tiff-Annie Kenny, Sonia Weshe, Aline Philibert and Laurie Hing Man Chan
University of Ottawa, Ottawa, Canada, laurie.chan@uottawa.ca

#### 

Recent studies indicate elevated rates of household food insecurity in the Canadian Arctic region. Community remoteness and northern latitude often restrict the affordable access to fresh and nutritious market foods. Consumption of locally harvested, cultural foods, therefore, remains deeply embedded in the food security, nutrition and health of Inuit. Despite this, shifts in traditional/country food consumption have been taking place over the past two decades related to a variety of changes in northern ecological, social, political and economic systems. Those related to ecological shifts have been in part previously associated with reduced confidence in food safety due to identified threats from environmental contaminants, and more recently the changes in species availability and accessibility due to shifting climatic conditions. Inuit communities have witnessed a general decline in the health and population status of many wildlife species consumed as country foods associated with climate change. Moreover, changes in ice, snow, precipitation regimes and other environmental factors have the potential to influence human travel and transportation in the North, and thus Inuit access to these wildlife resources. As such, climate change and variability has the potential to influence nutrition and health status among Inuit via impacts on aspects (availability, accessibility and quality) of traditional/country food security. The overall goal of our research program is to improve the status of food security among Inuit in the Canadian Arctic by understanding the determinants of food security and developing culturally appropriate adaptation strategies. The search was conducted in partnership with regional Inuit organizations and health authorities to facilitate knowledge translation into public health policy. It is our hope that methods developed and experienced gained in Canada can be shared with other circumpolar countries.

**References**

1. Fillion M, Laird B, Douglas V, Van Pelt L, Archie D, Chan HM. Development of a strategic plan for food security and safety in the Inuvialuit Settlement Region, Canada. Int J Circumpolar Health. 2014;73. doi: http://dx.doi.org/10.3402/ijch.v73.25091

2. Huet C, Rosol R, Egeland GM. The prevalence of food insecurity is high and the diet quality poor in Inuit communities. J Nutr. 2012;142:541–7.

3. CCA. Aboriginal food security in northern Canada: an assessment of the state of knowledge. Ottawa, ON: Council of Canadian Academies; 2014.

## One Health Symposium

### One Health: exploring One Health and expanded circumpolar collaborations

#### Alan Parkinson
Arctic Investigations Program, Centers for Disease Control and Prevention, Atlanta, GA, USA, parkinsonalanj@gmail.com

##### 

The globally interconnected nature of the ecosystem, animal and human health represents the foundation of the “One Health” concept defined as the “collaborative effort of multiple disciplines, working locally, nationally and internationally, to obtain optimal health for people, animals and the environment.” This concept is pertinent in the Arctic where the consequences of climate change already impact the environment and population dynamics of wildlife resources of critical and cultural importance of indigenous people’s health. Capitalizing on the “One health” approach in the Arctic requires interdisciplinary and community-based networks to monitor the impacts of climate and environmental change on human health. Observations grounded in local and traditional knowledge as well as local monitoring of key subsistence species for climate sensitive environmental threats can provide real time data on emerging one health issues and help to inform policy and management decisions. Arctic networks include the International Union for Circumpolar Health (www.iuch.net) and the Circumpolar Health Research Network (www.circhnet.org; Local Environmental Observer networks comprise tribal environmental, natural resources, and health professionals who share information on local events with subject matter experts who then provide feedback and adaptation strategies to tribal organizations. In addition, the Arctic Council (www.arctic-council.org) provides working group and indigenous peoples organization networks and the opportunity to engage circumpolar expertise. This One Health Symposium provides the opportunity to bring together veterinarians, public and environmental health professionals, tribal health practitioners, animal science researchers to address the challengers of how to operationalize the “One Health concept in the Arctic to improve the health and well-being of people living in the North.”

## “One Health”: a useful model for the development of a village-based environmental monitoring program to detect established and emerging threats to rural food and water safety in rural Alaska communities

### Jim Berner
Alaska Native Tribal Health Consortium, Anchorage, AK, USA, jberner@anthc.org

#### 

The term One Health is recent, but the belief that the physical environment and the ecosystem are linked, and changes in any component will eventually affect multiple parts of the system is an integral part of every Alaska Native culture, and is central to their understanding of the world around them. The current Arctic warming trend, with many visible impacts to the land, sea ice and wildlife, combined with the knowledge from western science that man-made contaminants, which cannot be seen or tasted, are now being transported to the circumpolar north, and are in the food chain. In addition, northward movement of the treeline in Alaska has brought species from more southern regions to the Arctic, with new diseases that can infect human residents. At the request of tribes in Alaska, the Alaska Native Tribal Health Consortium has utilized the combined traditional knowledge of communities, and principals of western science, to develop a village-based, resident-operated environmental monitoring program called the Rural Alaska Monitoring Program (RAMP), which is designed to monitor known threats, and detect emerging threats, and to follow trends. The RAMP can be modified to fit a community’s needs, and to develop adaptation strategies that will allow risk reduction for the most vulnerable residents, and continuation of the traditional diet for all residents. The RAMP operationalizes the “One Health” model, and takes advantage of traditional knowledge, and the most useful applications of western science, in response to the challenges of the changing environment.

## The Alaska One Health Group

### Mike Brubaker
Alaska Native Tribal Health Consortium, Anchorage, AK, USA, mbrubaker@anthc.org

#### 

The Alaska One Health Group was formed in early 2013, an initiative of the Alaska Native Tribal Health Consortium, Center for Climate and Health, and the US Centers for Disease Control, Arctic Investigations Program. The group includes environmental, health and wildlife professionals from Western Canada and Alaska. The group meets quarterly to share information on activities, to discuss emerging One Health issues, to consider events that are indicative of environment and climate change and to provide a forum for identifying areas of common interest and collaboration. The group is innovative in its use of social media, mapping and a network of local environmental observers to inform about emerging One Health issues. This presentation will describe the One Health Group structure and present a case study of how local observations have been applied within the group to raise awareness and develop collaborative responses.

## One Health in the Canadian North – priorities, perceptions, and programs

### Emily Jenkins^1^, Janna Schurer^1^, Audrey Simon^2^, Nicholas Bachand^1^ and Craig Stephen^3^
^1^University of Saskatchewan, Saskatoon, Canada, emily.jenkins@usask.ca; ^2^Université de Montréal, Boulevard Edouard-Montpetit, Canada; ^3^Canadian Wildlife Health Cooperative, Saskatoon, Canada

#### 

One Health practitioners seek to collaboratively address shared challenges to the health of people, domestic animals and wildlife. One Health is a living concept in Canada’s North, where Inuit are strongly connected to the land and wildlife, and these connections are critical to their health, food security and culture. However, arctic ecosystems are undergoing marked changes (climatic and anthropogenic), and these changes may be occurring so rapidly that they exceed the ability of Arctic indigenous peoples and wildlife to adapt, necessitating more effective and rapid means to translate research into action. One Health is not just a framework for surveillance or research, but also for action in a resource-limited environment for scientific research and public health programming. We propose a framework for prioritizing One Health issues that considers the following questions: 1) Does the pathogen in wildlife represent a risk to human health? 2) Does the pathogen have potential to adversely affect wildlife populations of conservation concern? 3) Is the pathogen perceived as a threat to people who rely on the health and sustainability of the wildlife resource? Successful actions within a One Health framework have in common a perception of risk (i.e. perceived threat), there is something practical that can be done about it (perceived feasibility), and there is support from stakeholders at multiple levels (i.e. communities, policy-makers and researchers), generally tied to a perceived benefit. We review programs that have been implemented in the Canadian North to help manage One Health issues, including food-borne parasites in harvested wildlife and disease transmission and injury from free-ranging dogs. We suggest future needs for surveillance, diagnostics and community-based research to address disease transmission at the human/animal/environmental interface in northern and indigenous communities in Canada.

**References**

1. Jenkins EJ, et al. Wildlife parasitology in a One Health world. Trends Parasitol. 2015;31.

2. Schurer JM, et al. Stabilizing dog populations and improving animal and public health through a participatory approach in indigenous communities. Zoonoses Public Health. 2015;62:445–55. doi: http://dx.doi.org/10.1111/zph.12173

## One Health challenges in Greenland

### Asbjørn Brandt
Chief Veterinary and Food Officer, Veterinary & Food Authority Government of Greenland, Nuuk, Greenland, asbr@nanoq.gl

#### 

In the Greenlandic National health plan, there is a goal for Greenland to be capable of preventing, detecting, containing, eliminating and responding to animal and public health risks attributable to pollution, zoonoses and animal diseases with an impact on food security through multi-sectoral cooperation and strong partnerships both in the Nordic and the artic area.

- Addressing health risks at the human animal-ecosystems and environmental interfaces requires strong partnerships among players who may have different perspectives on some issues and different levels of resources (international organizations, governments, civil society and donors).
- Strong coordination to minimize the burden of multiple monitoring, reporting and delivery systems, and to avoid duplicated efforts and fragmented outcomes.
- Framework for collaboration and manage existing and novel diseases that will be of public health, agricultural, social and economic importance in the future.
- Protocols and standards for managing emerging zoonotic diseases should be jointly developed.
- Cultural and educational challenges will be addressed.
- Examples of initiatives with One Health implications will be outlined – for example, traditional foods, utilization (slaughter) of marine and terrestrial mammals. Drinking water derived from melting “permanent” ice.

## Operationalizing One Health in the Arctic: towards a regional operational norm

### Joshua Glasser
Bureau of Oceans and International Environmental and Scientific Affairs, Department of State, Arlington, Virginia, USA, GlasserJL@state.gov

#### 

International circumpolar collaboration on health has enhanced partnerships and improved the health of Arctic residents and communities. However, significant challenges are already underway, and are likely to continue. In an era of global climate change, understanding the interface between the environment, and human and animal health systems will become increasingly critical for policy and practice. One Health is an interdisciplinary approach to assess health issues at the interface between humans, animals and ecosystems, and is thus well suited to address the multiple, complex and overlapping health risks associated with climate change in the Arctic. Enhancing the regional practice of One Health will improve scientific understanding, bolster the resilience of Arctic communities, and inform health policymaking. This presentation will lay out the case for a phased approach to operationalize One Health in the Arctic, based on co-equal and inclusive collaborations across scientific disciplines and stronger networks in the circumpolar region.

## Infectious Diseases I

### Water, sanitation and health in the Circumpolar North: addressing a long-standing health disparity

#### Thomas Hennessy
CDC Arctic Investigations Program, Anchorage, AK, USA, tbh0@cdc.gov

##### 

The relationship between a safe, plentiful water supply and health is an accepted public health principle, but is not well described in the circumpolar north. The historic focus of public water service has been to provide safe drinking water to prevent diarrheal illnesses such as cholera and typhoid. However, recent studies have shown that access to adequate water quantity for hand and body hygiene is also important for preventing illnesses that can be spread person-to-person, such as respiratory and skin infections. The Survey of Living Conditions in the Arctic in 2001 revealed that many people in Alaska, Canada, Greenland and Russia do not have access to in-home water and sanitation services. In rural Alaska, where approximately 20% of homes lack piped water service, water use averages 5.7 l/person/day; far below WHO recommendations for health. Compared to villages with in-home water service, hospitalizations for pneumonia among infants are 85% higher and skin infection hospitalizations among all ages are two-times higher. Similarly, invasive pneumococcal infections such as meningitis and bacteremia among children are two-times higher. A recent study of Alaska villages receiving in-home water service for the first time showed that water and soap use increased while respiratory, skin and intestinal infections decreased. Thus, health disparities can be reduced by providing adequate volumes of safe water. However, providing water service for all persons is prevented by factors of cost, engineering, regulations and political will. Climate change further threatens many existing northern water/sewer systems through erosion, permafrost melt, source water changes and new microbial threats. A reassessment of water/sanitation services and related health outcomes in the circumpolar north is needed to describe the current status, to compare best practices and to develop new approaches to improve access to water service and improve health.

**References**

1. Kruse J, Poppel B, Abryutina L, et al. Survey of Living Conditions in the Arctic (SliCA). In: Møller V, Huschka D, Michalos AC, editors. Barometers of quality of life around the globe how are we doing? Springer: Netherlands, Social Indicators Research Series; 2009. Vol. 33, p. 107–34.

2. Hennessy TW, Ritter T, Holman RC, et al. The relationship between in-home water service and the risk of respiratory tract, skin, and gastrointestinal tract infections among rural Alaska natives. Am J Public Health. 2008;98:2072–8.

3. Wenger JD, Zulz T, Bruden D, et al. Invasive pneumococcal disease in Alaskan children: impact of the seven-valent pneumococcal conjugate vaccine and the role of water supply. Pediatr Infect Dis J. 2010;29;251–6.

## Impact of in-home water service on the rates of infectious diseases: results from four communities in Western Alaska

### Timothy Thomas^1^, Troy Ritter^1^, Dana Bruden^2^, Michael Bruce^2^, Kathy Byrd^2^, Thomas Hennessy^2^, Rachel Goldberg^1^, Korie Hickel^1^ and Jeff Smith^1^
^1^Alaska Native Tribal Health Consortium, Anchorage, AK, USA, tkthomas@anthc.org; ^2^Centers for Disease Control and Prevention, Atlanta, GA, USA

#### 

*Background.* About 20% of rural Alaskan homes lack in-home piped water; residents must haul water to their homes. Recent studies in Alaska demonstrated associations between increased rates of skin and respiratory tract infections and lack of in-home piped water, presumably due to a reduced quantity of water available for handwashing, bathing and laundry (termed “water-washed” infections). We assessed rates of water-related infections in residents of communities transitioning to in-home piped water. *Methods*. Residents of four communities consented to review of medical records for the period 3 years before and 3 years after their community received piped water. We selected clinic and hospital encounters with ICD-9CM codes for respiratory, skin and gastrointestinal (GI) infection and calculated annual illness episodes for each infection category after adjusting for age and removing repeat encounters within 14 days of initial report. *Results.* We enrolled 1032 individuals (72% of total 2010 four-community population) and obtained 5,477 person-years of observation. There were 9,840 illness episodes with at least one ICD-9CM code of interest; 8,155 (83%) respiratory, 1,666 (17%) skin, 241 (2%) GI. Water use increased from average 5.7 l/capita/day (l/c/d) to 97.3 l/c/d. There were significant (p<0.05) declines in respiratory [16.4%, 95% confidence interval (CI): 11.5–21.0%], skin (20.4%, 95% CI: 10.0–29.7%), and GI infections (37.8%, 95% CI: 13.3–55.3%) in homes that received piped water, mostly among those aged 0–19 years. *Discussion.* Households that must haul water are severely limited in the amount of water available for personal hygiene. We demonstrated significant declines in respiratory, skin and GI infection rates among individuals in communities that transitioned from hauling water to in-home piped water. This study reinforces the importance of adequate *quantities* of water to address the morbidity caused by water-washed infection.

## Alaska Native consumers of modern sanitation services provide insights to inform infrastructure designs and health promotion planning

### Troy Ritter^1^, Ellen Lopez^2^, Korie Hickel^1^, Jennifer Dobson^3^, Jeffrey Smith^1^, Rhonda Johnson^4^ and Andrea Bersamin^2^
^1^Alaska Native Tribal Health Consortium, Anchorage, AK, USA, khickel@anthc.org; ^2^University of Alaska, Fairbanks AK, USA; ^3^Yukon Kuskokwim Health Corporation, Whitehorse, USA; ^4^University of Alaska, Anchorage, AK, USA

#### 

Acute respiratory infections, diarrhea and skin infections are diseases of public health significance in Alaska. Installation of modern sanitation services, such as pressurized running water piped to the home and flush toilets, is a common preventative strategy. While previous research has documented lower rates of disease among residents with modern services, it is not known if and to what degree residents recognize the health benefits. In-person surveys were conducted with residents of four Alaska Native communities who had recently received modern services. Most participants (n=101; 74%) reported improved community health. A prominent theme among participants who reported improved health was that better convenience and access to water resulted in an increase in the volume of water used. Participants further explained how installation of modern sanitation services increased their ability to perform six healthy water use practices known to prevent disease. Our findings suggest that the health benefits of modern services are recognized by consumers. We recommend providing modern sanitation services where possible and augmenting provision of infrastructure with education to encourage healthy water use. New water system designs for communities that cannot support modern infrastructure should prioritize making the water supply convenient and plentiful to encourage healthy practices.

## Epidemiology of *Haemophilus influenzae* serotype a from 2000 to 2013, an emerging pathogen in northern Canada and Alaska

### Michael Bruce^1^, Tammy Zulz^1^, Anita Li^2^, Debby Hurlburt^1^, Karen Rudolph^1^ and Debyle Caorolyn^1^
^1^Centers for Disease Control and Prevention, Atlanta, GA, USA, zwa8@cdc.gov; ^2^Public Health Agency of Canada, Montréal, Canada

#### 

*Background.* Prior to introduction of the *Haemophilus influenzae* type b (Hib) conjugate vaccines, rates of Hib disease among indigenous people living in Alaska (AK) and northern Canada (N Can) were among the highest reported in the world. Routine vaccination has reduced these rates to very low levels; however, serotype replacement with non-type b strains is of concern. *Methods.* We identified cases of invasive Hi disease in AK and N Can from 2000 to 2013 through the International Circumpolar Surveillance (ICS) network. Medical charts were reviewed on laboratory-confirmed cases using standard forms to verify clinical presentation. AK and N Can estimated populations as of 2013 were 736,399 and 157,602, respectively; Aboriginal peoples comprised 19% of the population in AK and 56% in N Can. *Results.* During the study period, a total of 413 cases of invasive Hi disease were reported from AK (242) and N Can (171); 126 (52%) in AK and 143 (88%) in N Can were typeable Hi. In AK, 40 (32%) were serotype a, 37 (29%) were serotype b, 32 (25%) were serotype f. In N Can, 106 (74%) were serotype a, 21 (15%) were serotype b, 4 (3%) were serotype f. Among 146 Hia isolates (data from AK and N Can combined), 129 (88%) occurred in indigenous people; median age was 1.0 year (range: newborn to 80 years); 58% were male. 8% of Hia cases were fatal. Common clinical presentations included: meningitis (29%), pneumonia (26%) and septic arthritis (12%). There were no cases of epiglotittis. Overall, annual Hia incidence was 0.4, and 5.4 cases/100,000 population in AK and N Can, respectively. Annual incidence rates among indigenous children <2 years old in AK and N Can were 36 and 107 cases/100,000 persons, respectively. *Conclusions.* Serotype a is now the most common Hi serotype in the North American Arctic, especially in northern Canada, with highest rates among indigenous children. Further research is needed to investigate regional differences in rates, and to determine clinical severity and sequelae.

## Antimicrobial susceptibilities of isolates from patients with invasive bacterial diseases in northern Canada

### Yuanyuan Li^1^, Gregory Tyrrell^2^, Irene Martin^1^, Walter Demczuk^1^, Raymond Tsang^1^, Susan Squires^1^, Shalini Desai^1^; Invasive Bacterial Diseases Working Group^3^
^1^Public Health Agency of Canada, Montréal, Canada, y.anita.li@phac-aspc.gc.ca; ^2^Alberta Provincial Laboratory for Public Health, Calgary, Canada; ^3^International Circumpolar Surveillance (ICS), Atlanta, GA, USA

#### 

The incidence rates of invasive bacterial diseases (IBDs) are known to be higher in northern Canada (N Can) than the rest of the country. This drives increased antibiotic use which potentially may lead to increased numbers of antimicrobial resistant organisms (AROs). The objective of this study was to document the rates of AROs for select IBDs in N Can. Data from the International Circumpolar Surveillance (ICS) network for invasive pneumococcal disease (IPD) (1999–2013), invasive group A streptococcal disease (iGAS) and invasive Haemophilus influenzae disease (Hi) (2000–2013) in N Can were analysed. Antimicrobial susceptibility (AMS) and antimicrobial classification were determined according to Clinical and Laboratory Standards Institute guidelines. AMS testing was conducted for 92% (478/520) of IPD, 75% (117/155) of iGAS and 32% (55/171) of Hi cases. Resistance to a single antimicrobial was observed in 10.9% (N=52) IPD isolates, 1.5% (7) to two classes and 2.9% (14) to three or more classes; of the IPD isolates tested, 8.2% (39) were resistant to trimethoprim/sulfamethoxazole, 6.5% (31) to erythromycin and 4.3% (21) to penicillin (4.3%). Among iGAS isolates, 9.4% (N=11) were resistant to one antimicrobial class and 5.2% (6) to two or three classes; 10.3% (12) were resistant to erythromycin, 5.1% (6) to clindamycin and 2.6% (3) to trimethoprim/sulfamethoxazole. Among Hi isolates, 20% (N=11) were resistant to one antimicrobial class and 3.6% (2) to two or three classes; 12.7% (7) were resistant to ampicillin and 3.6% (2) to trimethoprim/sulfamethoxazole. Isolates from IPD and iGAS cases were resistant to various antimicrobials whereas isolates from Hi cases were resistant primarily to ampicillin. Multi-class resistance continues to be a concern for IBDs. Further research of the temporal and geographical distributions of AROs is required to better formulate effective public health interventions and reduce the burden of IBDs in N Can.

## The Epidemiology of Invasive Group A Streptococcal Infections in the North American Arctic, 2000–2012

### Michael Bruce^1^, Tammy Zulz^1^, Karen Rudolph^1^, Dana Bruden^1^, Anita Li^2^ and Irene Martin^2^
^1^Centers for Disease Control and Prevention, Atlanta, GA, USA, zwa8@cdc.gov; ^2^Public Health Agency of Canada, Montréal, Canada

#### 

*Background.* Invasive group A Streptococcus (GAS) infection causes morbidity and mortality in the Arctic; however, the epidemiology of this disease is not well described in this region. *Methods.* We reviewed population-based data on invasive GAS disease in Alaska (AK) and northern Canada (N Can) collected from 2000 to 2012 through the International Circumpolar Surveillance (ICS) network. We defined invasive GAS as an isolate from a normally sterile site (or surgical wound/deep soft tissue infection) from a surveillance region resident. In AK and N Can, emm typing and/or M typing were performed, respectively. Chart reviews were conducted on lab-confirmed cases. *Results.* During the study period, a total of 627 cases of invasive GAS disease were reported from AK (483) and N Can (144); 346 (55%) occurred in indigenous persons. Median age was 46.6 years, 55% were male; 63 (10%) cases were fatal. Common clinical presentations included cellulitis with bacteremia (40%), pneumonia with bacteremia (18%) and bacteremia of unknown source (23%); 49 (8%) cases of necrotizing fasciitis occurred; the case fatality ratio for necrotizing fasciitis was 43%. In AK, the three most common emm types were 1 (12%), 82 (9%) and 49 (8%); in N Can, the most common emm types were 1 (13%), 59 (10%) and 91 (7%). In AK, annualized incidence rates were 13 and 3.7 cases per 100,000 persons among indigenous and non-indigenous persons, respectively (RR=3.5, p<0.01). In N Can, rates were 11.8 and 1.7 cases per 100,000 among indigenous and non-indigenous persons, respectively (RR=7.4, p<0.01). Incidence rates of invasive GAS increased 1.6 and 2.3 times in AK and N Can, respectively, when comparing baseline study years (2000–2002) to the last three years (2010–2012). *Conclusions.* GAS remains an important cause of invasive bacterial disease in the North American Arctic. Rates of invasive disease with GAS were higher among indigenous persons. Incidence rates in AK and N Can appear to be rising, which merits further evaluation.

## Toxoplasmosis in wildlife and people in the Canadian Arctic

### Emily Jenkins, Janna Schurer and Stacey Elmore
University of Saskatchewan, Saskatoon, Canada, emily.jenkins@usask.ca

#### 

*Toxoplasmosis*, caused by the protozoan parasite *Toxoplasma gondii*, can cause long-term consequences for congenitally infected infants, and clinical disease, especially for those with compromised immunity. National seroprevalence is unknown in the general Canadian population, but is thought to be comparable to the United States (approximately 15–20%). We reviewed national data on clinical toxoplasmosis in Canada. The annual incidence rate of toxoplasmosis was 1.65 cases per 1 million persons. Human immunodeficiency virus (HIV) was a co-morbidity in 40% (209/523) of cases. Toxoplasmosis was diagnosed most frequently in patients who resided in the lowest neighbourhood income quintile. Males were more likely to experience clinical toxoplasmosis severe enough to require medical care, whereas in other studies, more females than males were seropositive for *T. gondii*. We observed an urban bias, where people are presumably exposed to *T. gondii* through direct contact with oocysts shed by pet cats, contaminated garden produce, and other foodborne routes of exposure. This is in contrast with toxoplasmosis in the Canadian North, where pet cats are rare, and consumption of harvested wildlife and untreated drinking water are thought to be significant risk factors. In the Canadian North, some indigenous communities have high levels of exposure (60–87%) and outbreaks of congenital toxoplasmosis have been reported. We found that migratory geese could be a source of toxoplasmosis in terrestrial ecosystems in the central Canadian Arctic. Differences in seroprevalence, incidence of clinical disease and sources of exposure to *T. gondii* between the general urban population and northern indigenous populations in Canada emphasizes that targeted control and education measures need to consider heterogeneity in exposure, co-morbidity and cultural practices. As a zoonosis, toxoplasmosis should be managed through a One Health approach, integrating physicians and veterinarians.

**References**

1. Elmore SA, et al. *Toxoplasma gondii* exposure in arctic-nesting geese: a multi-state occupancy framework and comparison of serological assays. Int J Parasitol Parasites Wildl. 2014;3:147–53.

2. Elmore SA, et al. *Toxoplasma gondii* in people and wildlife of the circumpolar north. Vector Borne Zoonotic Dis. 2012;12:1–9.

3. Jenkins EJ, et al. Tradition and transition: parasitic zoonoses of people and animals in Alaska, northern Canada, and Greenland. Adv Parasitol. 2013;82:33–204.

## Infectious Diseases II

### Summary of current viral hepatitis surveillance, treatment and prevention programs in Arctic countries

#### Prabhu Gounder^1^, Brian McMahon^2^ and Thomas Hennessy^1^
^1^Centers for Disease Control and Prevention, Atlanta, GA, USA, iym4@cdc.gov; ^2^Alaska Native Tribal Health Consortium, Anchorage, AK, USA

##### 

Viral hepatitis is an important problem in many Arctic countries. Hepatitis A virus infection remains endemic with periodic outbreaks despite the availability of an effective vaccine. The World Health Organization (WHO) will release new guidelines in 2015 for the management of persons with chronic hepatitis B virus infection living in resource-limited settings. Similarly, WHO and many Arctic countries are updating guidelines for the management of chronic hepatitis C virus infection to guide use of recently approved and highly-effective direct-acting antiviral agents. To provide a baseline for evaluating the impact of these changes, we sought to describe the surveillance systems and treatment/prevention programs currently in place for hepatitis A, B and C in countries with Arctic populations. We developed and distributed a survey to representatives at public health agencies in the eight Arctic countries (USA [Alaska], Canada [3 northern provinces], Greenland, Iceland, Finland, Norway, Sweden and Russia). The survey included questions regarding key elements of the surveillance system (e.g. case definitions, screening programs, requirements for case reporting, any follow-up/linkage to care of cases), prevention programs (e.g. vaccination guidelines, funding for vaccinations) and treatment programs (e.g. existence of treatment registries, funding for treatment, treatment guidelines). Based on the survey responses, we will compare the viral hepatitis management programs among the Arctic countries/regions. Our survey results could help countries identify opportunities to improve their surveillance systems and treatment/prevention programs. In addition, the survey can identify areas where all countries collect similar data; synchronizing and sharing those data between Arctic countries can facilitate collaboration on epidemiologic research questions of shared interest.

## New prospects for hepatitis C treatment and new WHO guidelines for the management of hepatitis B and C

### Brian McMahon^1^, Lisa Townshend-Bulson^1^, Prabhu Gounder^2^, Michael Bruce^2^ and Brenna Simmons^1^
^1^Alaska Native Tribal Health Consortium, Anchorage, AK, USA, bmcmahon@anthc.org; ^2^Arctic Investigations Program, CDC, Anchorage, USA

#### 

Worldwide, >400 million persons have chronic hepatitis B virus (HBV) infection and >200 million have chronic hepatitis C virus (HCV) infection. In the Circumpolar Region, HCV and HBV are major health problems, with endemic areas of chronic HBV in Western Alaska, Greenland, indigenous populations in Canada and the Russian Federation and HCV in many areas in Alaska, Canada, Russia, Denmark, Sweden, Norway and Finland. In these regions, delivery of services to manage HBV and HCV is challenging due to distances involved between infected patients and health care facilities, lack of providers trained in the management of chronic hepatitis and often harsh weather conditions. WHO published guidelines for the management of HCV in 2014 and HBV in 2015. To establish programs to implement HBV and HCV Guidelines in the Arctic several components are needed: 1) programs to screen at risk patients and high prevalence populations, 2) establishment of laboratory capabilities to test for liver function viral levels, 3) capabilities to determine the stage of liver disease using serologic markers, transient elastography or liver biopsy, 4) access to drugs to cure HCV and control HBV at reasonable prices, 5) personnel trained in the screening, diagnosis and management, 5) training programs for healthcare workers on the management of HBV and HCV, 6) programs for screening high risk infected patients for hepatocellular carcinoma with liver ultrasound and/or alpha-fetoprotein and 7) educational programs for the general public on screening for HBV and HCV and Programs for infected patients on general health recommendations and importance of follow-up. The Liver Disease and Hepatitis Program of the Alaska Native Tribal Health Consortium has comprehensive programs for the targeted screening of persons for HCV and management of chronically infected persons with both HBV and HCV that can serve as a model for other circumpolar regions.

**References**

1. McMahon BJ, Hennessy TW, Christensen C, Bruden D, Sullivan DG, Homan C, et al. Epidemiology and risk factors for hepatitis C in Alaska Natives. Hepatol. 2004;39:325–32.

2. McMahon BJ, Bulkow LR, Singleton RJ, Williams J, Snowball M, Homan C, et al. Elimination of hepatocellular carcinoma and acute hepatitis B in children 25 years after a hepatitis B newborn and catch-up immunization program. Hepatol. 2011;54:801–7.

3. Singleton RJ, Hess S, Bulkow LR, Castrodale L, Provo G, McMahon BJ. Impact of a statewide childhood vaccine program in controlling hepatitis A virus infections in Alaska. Vaccine. 2010;28:6298–304.

## Serological and molecular epidemiological outcomes after two decades of universal infant hepatitis B virus (HBV) vaccination in Nunavut, Canada

### Carla Osiowy^1^, Chris Huynh^1^, Gerald Minuk^2^, Julia Uhanova^2^, Maureen Baikie^3^, Lianne Vardy^4^ and Thomas Wong^4^
^1^National Microbiology Laboratory, Public Health Agency of Canada, Winnipeg, Canada, carla.osiowy@phac-aspc.gc.ca; ^2^Department of Medicine, University of Manitoba, Winnipeg, Canada; ^3^Nunavut Department of Health, Iqaluit, Canada; ^4^Infectious Disease Prevention and Control Branch, Public Health Agency of Canada, Toronto, Canada

#### 

*Background.* Chronic hepatitis B virus (HBV) is considered endemic (>2% prevalence) in Arctic regions (1). To control HBV infection in Nunavut, vaccination programs were initiated almost 20 years ago, targeted at newborns and grade school students, such that those born after 1980 should be vaccinated. This study investigates the effectiveness of these programs and is the first post-vaccination era survey to determine HBV prevalence in Nunavut. *Methods.* Anonymized serum specimens scheduled for destruction following medical testing were collected between April 2013 and May 2014 from individuals granting consent. Date of birth, gender and health centre community were recorded for all specimens. Specimens were tested for anti-HBs, anti-HBc and anti-hepatitis C virus (HCV) antibodies. Anti-HBc+ samples were tested for HBsAg and those positive underwent HBV DNA characterization. *Results.* 4,802 specimens (approximately 13.5% of the population) were collected according to the age distribution of Nunavut. Anti-HCV+ prevalence was 0.5%. Total anti-HBc+ prevalence was 9.4%, with a 10-fold decrease in the rate of HBV exposure noted among those born after 1980 compared with those born before (1.89% vs. 20.1%, p<0.001). HBsAg positivity was primarily in individuals born before 1980 (2.55%), although cases still occurred among the vaccine age cohort (0.21%). HBV subgenotype B6, known to be unique among Inuit and Alaska Native people (2), was the most prevalent genotype observed (84%). Vaccine-based antibody as the sole serological marker was evident in the vaccine age cohort, although the rate of decay with increasing age was much greater than anticipated. *Conclusion.* HBV vaccination programs in Nunavut have resulted in a decreased HBV prevalence to 1.17%, indicating a non-endemic state. However, the persistence of infection and a lower than expected prevalence of vaccine-based immunity in the vaccine age cohort will require further investigation to understand the causes and consequences.

**References**

1. Osiowy C, Simons B, Rempel J. Distribution of viral hepatitis in indigenous populations of North America and the circumpolar Arctic. Antivir Ther. 2013;18:467–73.

2. Osiowy C, Larke B, Giles E. Distinct geographic and demographic distribution of Hepatitis B virus genotypes in the Canadian Arctic as revealed through an extensive molecular epidemiological survey. J Viral Hepat. 2011;18:e11–9.

## Preliminary association of hepatitis B-specific, immune-mediated inflammatory responses with chronic hepatitis b disease activity

### Brenna Simons-Petrusa^1^, Minjun Apodaca^2^, Syn Lim^3^, Tammy Choromanski^1^, Danielle Pratt^1^, Susan Negus^1^, Mary Snowball^1^, Chriss Homan^1^, Chihiro Morishima^2^, Cindy Knall^3^ and Brian J. McMahon^1^
^1^Alaska Native Tribal Health Consortium, Anchorage, AK, USA, bcsimons@anthc.org; ^2^University of Washington, USA; ^3^University of Alaska, Anchorage, AK, USA

#### 

High prevalence of chronic hepatitis B (CHB) infection disproportionately affects indigenous Arctic populations. During CHB infection, the hepatitis B virus (HBV) can activate immune-mediated inflammation and liver damage. Some chronic HBV carriers experience active disease with high HBV DNA levels and liver inflammation, some of whom may benefit by antiviral treatment, while others experience inactive disease with low HBV DNA levels and no liver inflammation. Clinical management of chronic HBV can be challenging, often requiring ultrasound or liver biopsy, procedures that may not be available in many rural Arctic communities. Biomarkers of CHB disease status that can easily be measured from a small blood draw are needed. The role of adaptive immunity in CHB infection and and whether disease status can be inferred from immune biomarkers is incompletely understood. In this study, we compared HBV-specific immune responses between persons with inactive (n=9) and active (n=9) CHB. Peripheral blood mononuclear cells were assessed by flow cytometry for the frequency and phenotype of immune cells. Ex vivo cultures were established to measure HBV-specific IFNγ cytokine response by flow cytometry and ELISpot. A trend toward an increase in regulatory T cells (Treg) amongst inactive carriers was identified. CD45RO+CD4+FoxP3+ Treg cell frequency was significantly (p=0.019) higher amongst active versus inactive carriers. Pro-inflammatory, HBV-specific IFNγ responses were significantly (p=0.05) increased, while anti-inflammatory HBV-specific IL-10 responses were significantly (p=0.028) decreased, among active carriers. Collectively, these data suggest increased immune inflammatory responses among active carriers. Recent improvements in laboratory methods may allow for the expansion of cellular immune testing in remote settings, paving the way for needed population-based immune studies of CHB infection in the Arctic.

## Network analysis of chlamydia and gonorrhea in the Northwest Territories, 2014

### Yalda Jafari^1^ and Ann Jolly^2^
^1^Government of Northwest Territories, Yellowknife, Canada, yjafari01@gmail.com; ^2^University of Ottawa, Ottawa, Canada

#### 

*Background.* Compared to the latest Canadian rates, in 2012, the rates of Chlamydia and Gonorrhea in Northwest Territories (NWT) were seven and almost 12 time the Canadian rates, respectively. A sexual network analysis was conducted in order to better understand the burden of these diseases in NWT and identify possible interventions to curtail higher than national rates. *Methods.* Territorial data on confirmed positive chlamydia and gonorrhea cases in 2014 were retrieved, including contact information. This data were used to conduct a network analysis using the graphing program Gephi. *Results.* A total of 817 individuals confirmed positive for either chlamydia or gonorrhea along with 432 contacts were included in this study, compromising a network of 1249 individuals with 929 links. The final network contained 354 weakly connected components, ranging from 1 individual to 389 individuals. The weighted degree centrality ranged from 0 to 24. The average path length was 11.9. Additional analyses by community and seasonality were conducted. *Conclusion.* Despite challenges in conducting partner notification across a large territory, we found large clusters of connected people. Identifying these groups through routine social network analysis will facilitate improved interventions.

**Reference**

1. Public Health Agency of Canada. Notifiable diseases on-line. [cited 2014 Mar 31]. Available from: http://dsol-smed.phac-aspc.gc.ca/dsol-smed/ndis/charts.php?c=pl

## Tuberculosis outbreak in East Greenland. Groups at risk in an isolated Arctic setting

### Karen Bjorn-Mortensen^1,2^, Aase Bengaard Andersen^3,4^, Anders Koch^1^, Karin Ladefoged^5^, Troels Lillebaek^2^, Sascha Wilk Michelsen^1^, Thomas Rendal^6^, Mikael Andersson^1^, Jacob Simonsen^1^ and Bolette Soborg^1^
^1^Department of Epidemiology Reseach Statens Serum Institut, Copenhagen, Denmark, Kabm@ssi.dk; ^2^International Reference Laboratory of Mycobacteriology, Copenhagen, Denmark; ^3^Department of Infectious Diseases, Copenhagen University Hospital, Copenhagen, Denmark; ^4^University of Southern Denmark, Odense, Denmark; ^5^Department of Internal Medicine, Queen Ingrid′s Hospital, Nuuk, Greenland; ^6^National Board of Health, Nuuk, Greenland

#### 

*Introduction*. A tuberculosis (TB) outbreak emerged in East Greenland in 2009. Prior to the outbreak East Greenland had a TB incidence of 319 per 100,000 inhabitants and, due to the isolated nature of habitation, areas with ongoing Mycobacterium tuberculosis (Mtb) transmission are areas free of transmission. This study aims to identify groups at risk during the outbreak, and to evaluate whether individuals from a previous Mtb transmission-free environment carry a particular risk. *Methods.* A cohort including all inhabitants living in East Greenland on 1 January 2008 was created. Study participants were identified through the Civil Registration System and followed until TB, emigration or 31 December 2012. TB cases were identified through the mandatory TB notification system. Mtb infection (MTI) was determined by interferon-γ release assay (IGRA) results. TB incidences (IR) were estimated by Cox proportional hazard model and MTI prevalence by interval censoring of IGRA results. *Results.* 3,541 individuals were followed for 17,028 person-years (PY). The TB IR was 1,730/100,000 PY for the previous Mtb transmission-free settlement and 704/100,000 PY for the rest of East Greenland. Likewise, the estimated MTI prevalence at the end of 2012 was 60.9 and 37.5%, respectively. For teenagers, TB IR was 7,389/100,000 PY for the settlement and 2,210/100,000 PY for the rest of East Greenland, and the estimated MTI prevalence was 79.1 and 41.9%. *Conclusion.* The TB incidence rates more than doubled during the outbreak. Teenagers were a particular group at risk. Individuals from a previously Mtb transmission-free settlement carried an extra two-fold increased risk of MTI and TB disease.

## AMAP Report

## Human exposure to pollutants and their health endpoints

### Jon Øyvind Odland
AMAP HHAG, the Arctic University of Norway, Tromsø, Norway, jon.oyvind.odland@uit.no

#### 

Studies of longitudinal design are ongoing in all Arctic countries, as well as many countries in the southern hemisphere. For both ethical and scientific reasons, many studies have a cohort design, with a long term follow up of mothers and their respective children. The most vulnerable period in a human life is before you are born and early childhood. A number of reports and publications are now coming out of these studies and the report will go into details of the most important studies and their defined health endpoints. Different epidemiological studies in the circumpolar area have shown associations between contaminants and different health outcomes. It is important to note that associations are not the same as causal relationships between exposure to a single substance (or substances) and an effect. When finding an association between a contaminant and an effect, it is important to bear in mind the possibility that the studied substance is a proxy for other substances in the mixture of contaminants to which the study population has been exposed – both harmful and beneficial. The effects reported are findings from different circumpolar communities. However, due to differences in genetic composition, socio-cultural practices, local food consumption patterns and exposure mixtures, a finding in one population should not be extrapolated to another population without careful investigation and comparative information. Different health outcomes are presented; neurotoxic effects, immunotoxic effects, reproductive effects, cardiovascular effects, endocrine effects, carcinogenic effects, as well as risks and benefit of breast feeding. Risk assessment of the effects of environmental pollutants is an absolutely essential tool in the overall process for protecting health of Arctic residents. Different tools and methods are available. One of the biggest challenges is how to translate contaminant concentrations measured in blood into information useful for risk characterization (the likelihood that specific effects will occur at these concentrations). Climate change and future change of exposure will further complicate the assessment, providing good rationale to continue monitoring and assessment of the exposure and related health effects.

## Environmental Contaminants I

## Metal Content in Water Sources and in Drinking Water in the Industrial Cities of Murmansk oblast, Russian Arctic

### Eugenia Dushkina, Alexey Dudarev, Yuliya Sladkova, Valery Chupakhin and Alexander Nikanov
Northwest Public Health Research Center, Saint Petersburg, Russia, dushka9005@mail.ru

#### 

Currently Murmansk oblast is extracting dozens of minerals from the ores: copper-nickel, apatite-nepheline, iron, rare earth metals. The largest production sites (mining-ore-processing plants) of the area are “Apatite” in Kirovsk and Apatity cities, “Severonickel” in Monchegorsk city, “Pechenganickel” in Nickel and Zapolyarny cities, and “Olenegorsky” in Olenegorsk city. Industrial emissions from these sites have significant environmental influence also on surface waters and groundwater. Water sampling from water bodies, centralized and non-centralized sources of drinking water was conducted in the fall 2013 in the frames of Kolarctic project “Food and health security in the Norwegian, Russian and Finnish border regions” in six cities of Murmansk oblast mostly exposed to industrial emissions. Chemical analysis of 15 metals (Fe, Mn, Cu, Ni, Zn, Cd, Pb, Co, Hg, As, Cr, Sr, Al, Ba and Sn) in water samples has been carried out. It was found that most of the cities have no sanitary-protection zones of water sources (lakes) and no water pre-treatment facilities; water disinfection is carried out mainly by chlorination. The content of 13 metals in water samples in six cities at three points (water intake from the source, water treatment and distribution net) does not exceed Russian hygienic limits. However 2 metals in water samples (incl. drinking water) revealed significant excess (2–5 times) over the maximum permissible concentration – Al in Kirovsk city and Ni in Zapolyarny and Nickel cities. Measures aiming at cleaning of drinking water from the metals in three cities are of great importance. In all the cities surveyed, the water pipe transportation from water source to distribution network is characterized by the multiple increase of concentrations of Fe, Mn, Zn, etc. in water; this fact indicates the need of replacement of worn-out water networks by modern ones. Water of springs located nearby Nickel and Zapolyarny cities demonstrate significantly lower metal content except Sr and Ba.

## Metal content in local foods in Pechenga District of Murmansk oblast, Arctic Russia

### Alexey Dudarev, Eugenia Dushkina, Valery Chupakhin and Yuliya Sladkova
Northwest Public Health Research Center, St. Petersburg, Russia, alexey.d@inbox.ru

#### 

Nickel and Zapolyarny are two main cities of Pechenga district of Murmansk oblast; they are located in close proximity to industrial sites of “Pechenganikel” mining-ore-processing-metallurgical plant. In 2009, the plant air emissions of Ni were about 340 tons/year and of Cu – about 180 tons/year. In the frames of Kolarctic project, “Food and health security in the Norwegian, Finnish and Russian border region” in autumn 2013 in Pechenga district we carried out sampling of local foods, including fish (from six lakes), game, mushrooms, wild and garden berries, vegetables from private gardens – at various distances and directions from Nickel and Zapolyarny cities; also questioning of 400 residents was conducted. Analysis of 13 metals (Pb, As, Cd, Hg, Cu, Zn, Ni, Cr, Fe, Mn, Co, Sr and V) in food products was carried out. With the help of questionnaire, it was found out that local foods in Pechenga district constitute a significant proportion of the diet of the population. Excesses of metals in food over Russian maximum permissible concentrations (MPC) were identified for Cd in mushrooms (laminar and tubular) by 1.5–2 times, for Hg in orange-cap boletus – up to three times. Highest concentrations of Hg (close to MPC) were found in freshwater fish. While assessing the content of other metals, regulated earlier in the USSR, the excesses of Cu in milk-cap mushrooms (1.5 MPC), of Ni in wild berries (up to 4.5 MPC), garden berries (up to 2.5 MPC), potatoes (up to 2 MPC) and mushrooms (from 2.5 to 30 MPC) were found. Mushrooms should be considered as the main “sorbents” of almost all metals valued. Increase of distance from the plant was associated with reduction of content of Ni, Co, Mn, Cd, As and Sr but with rise of Hg and Cu in freshwater fish. Ni should be considered as the most significant factor of exposure (and health risks) of the population surveyed. The data obtained will help to develop recommendations for restraining the consumption of certain local foods.

## Lifestyle factors and dietary habits among pregnant women in Greenland

### Eva Cecilie Bonefeld-Jørgensen^1^, Ane-Kersti Knudsen Knudsen^1^, Manhai Long^1^ and Henning Sloth Pedersen^2^
^1^University of Aarhus, Aarhus, Denmark, ebj@ph.au.dk; ^2^Primary Health Care Center, Nuuk, Greenland

#### 

The Greenland population has changed their lifestyle and diet for the past decades. The living conditions have changed from hunter to a more western lifestyle including less intake of traditional food to a higher intake of imported food. These changes are thought to impact culture and public health in Greenland.The aim was to examine lifestyle factors and dietary habits among pregnant women in Greenland in a cross-sectional study of 189 pregnant women in Greenland. Inclusion criteria: >18 years and lived >50% of their life in Greenland. Data were collected in 2010–11. Information regarding lifestyle factors and dietary habits was obtained from two questionnaires. The age groups for comparison were women <27 years versus >27 years. A tendency of higher alcohol consumption during pregnancy, higher parity rate and miscarriages was seen for women >27 years of age, while women <27 years old had more doubt about planned breastfeeding period. Women <27 years of age consumed more dried fish and fast food. No significant age difference was seen for other traditional Greenlandic or imported food items, or lifestyle factors. The participants were divided in relation to where they had lived >50% of their life. Compared to the city Nuuk, women from remote towns smoked more, were in earlier gestational week, had higher parity and planned to breastfeed less. Comparing the regions West, Disko Bay, South, North and East, women living longest in West, North, and South, had a higher alcohol intake during pregnancy and women in North had less plans to breastfeed. Moreover, pregnant women in the North region consumed less fruits and vegetables and women in Disko Bay had the lowest intake of terrestrial species. No significant geographical differences were found for marine mammals or seabirds. The present study found several age and geographical differences regarding lifestyle factors and dietary habits among pregnant women in Greenland. Data related to the birth outcome will be present.

## Prenatal exposure to Hg and cognitive development: evidence that beneficial effect from maternal DHA intake can obscure adverse Hg effects

### Gina Muckle^1^, Sandra W. Jacobson^2^, Pierre Ayotte^1^ and Joseph L. Jacobson^2^
^1^Laval University, Quebec City, Canada, gina.muckle@psy.ulaval.ca; ^2^Wayne State university, Midtown Detroit MI, USA

#### 

*Background.* Although prenatal methylmercury exposure has been linked to poorer intellectual function in several studies, data from two major prospective, longitudinal studies yielded contradictory results. Associations with cognitive deficits were reported in a Faroe Islands’ cohort, but few were found in a study in the Seychelles Islands. It has been suggested that co-exposure to another contaminant, polychlorinated biphenyls (PCBs), may be responsible for the positive findings in the former study and that co-exposure to nutrients in methylmercury-contaminated fish may have obscured and/or protected against adverse effects in the latter. *Objectives.* To determine the degree to which co-exposure to PCBs may account for the adverse effects of methylmercury and the degree to which co-exposure to docosahexaenoic acid (DHA) may obscure these effects in a sample of Inuit children in Arctic Québec. *Methods.* IQ was estimated in 286 school-age children from Nunavik whom umbilical cord blood samples had been obtained and analyzed for mercury and other environmental exposures. *Results.* Prenatal mercury was related to poorer estimated IQ after adjustment for potential confounding variables. The entry of DHA into the model significantly strengthened the association with mercury, supporting the hypothesis that beneficial effects from DHA intake can obscure adverse effects of mercury exposure. Children with higher prenatal mercury exposure were four times more likely to score below the clinical cut-off for borderline intellectual disability. Co-exposure to PCBs did not alter the association of mercury with IQ. *Conclusions.* This is the first study to document an association of prenatal mercury exposure with poorer performance on a school-age assessment of IQ, a measure whose relevance for occupational success in adulthood is well established.

## Regional differences in subsistence practices and exposure to environmental contaminants and nutrients in Alaska’s Yukon-Kuskokwim Delta

### Marylynne Kostick^1^ and James Berner^2^
^1^Alaska Department of Fish and Game, Division of Subsistence, Juneau AK, USA, mlkostick@hotmail.com; ^2^Alaska Native Tribal Health Consortium, Anchorage, AK, USA

#### 

Environmental contaminants are found throughout the environment, making their way into our dietary sources. Regional differences in lifestyle and access to dietary sources may influence a population’s exposure to dietary sources of environmental contaminants and nutrients. We present an overview of subsistence harvest practices and maternal and umbilical cord blood levels of selected contaminants and nutrients in coastal and riverine communities from the Yukon-Kuskokwim Delta. Data for this study come from the Alaska Department of Fish and Game, Division Of Subsistence Community Subsistence Information System and the Alaska Maternal Organics Monitoring Study, a community-based participatory research study developed for the Yukon-Kuskokwim Health Corporation by the Alaska Native Tribal Health Consortium. Results present information on public health issues in rural Alaska and assist in the biomonitoring of environmental contaminants and their effect on maternal and infant health in the circumpolar north.

## Environmental Contaminants II

## Twenty-five years of research on environmental contaminants in Nunavik (Arctic Quebec, Canada)

### Pierre Ayotte^1^, Mélanie Lemire^1^, Thérèse Adamou^2^, Adel Achouba^2^ and Pierre Dumas^3^
^1^Departement of Social and Preventive Medicine, Laval University, Quebec City, Canada, pierre.ayotte@inspq.qc.ca; ^2^Population Health and Optimal Health Practices Research Unit, CHU de Québec Research Centre, Quebec, Canada; ^3^Quebec Toxicology Centre, Quebec Public Health Institute, Quebec, Canada

#### 

The Inuit of Nunavik (Arctic Quebec, Canada) have traditionally relied on foods harvested from the land and the sea for subsistence. While marine foods are rich in key nutrients such as omega-3 fatty acids and selenium (Se), some of them are also an important source of exposure to toxicants such as polychlorinated biphenyls (PCBs), organochlorine pesticides (OCPs) and methylmercury (MeHg). Biomonitoring data gathered over the last two decades in pregnant Inuit women from Nunavik indicate a substantial decrease in plasma levels of PCBs and most OCPs during this period, whereas the decline in blood mercury levels is more modest. While these classic food chain contaminants are still cause for concern, especially MeHg, public health authorities are now confronted to emerging persistent organic pollutants with lesser known toxicity. In addition, the exceptionally high Se intake of Inuit is now under scrutiny. Se is an essential element present in very high concentrations in beluga mattaq, a delicacy highly praised by Inuit. In fish and marine mammal eating populations, there is increasing evidence to suggest that the high Se intake may play a role in offsetting some deleterious effects of MeHg exposure. However, in other populations, elevated plasma Se concentrations have been recently associated to adverse health effects. Se species may differ between populations and this could explain why toxicity is encountered in some populations but not in others. We have recently completed Se speciation analysis (selenoproteins, selenocompounds) in blood samples of Inuit adults who participated to the 2004 Inuit Health Survey in Nunanik. Our results suggest that selenoneine is the Se compound accumulating in blood of high marine mammal consumers. We are currently measuring this recently discovered Se compound in red blood cells of participants and in Se-rich marine foods. These data will improve our capacity to assess the risks and benefits of the Inuit traditional marine diet.

## Arsenic and old mines: trust in risk communication about the Giant Mine Remediation Plan in the Canadian Northwest Territories

### Cindy Jardine^1^, S. Michelle Driedger^2^ and Chris Furgal^3^
^1^University of Alberta, Edmonton, Canada, cindy.jardine@ualberta.ca; ^2^University of Manitoba, Winnipeg, Canada; ^3^Trent University, Peterborough, Canada

#### 

Resource extraction activities, and the attendant harms to the environment and human health, are a major source of conflict and contention in the Canadian north. Risk communication and decision-making is further complicated by a postcolonial legacy of cumulative mistrust of government decision-makers, such as exists amongst many indigenous peoples. This research explored the elements of trust associated with the development and implementation of the Giant Mine Remediation Plan in the Canadian Northwest Territories, where residual arsenic trioxide continues to pose a risk to human health and the environment. Complementing earlier research which looked at this issue from the perspective of major government and non-government key informants (1), interviews and focus groups were conducted in 2013 and 2014 with representative members of the Yellowknives Dene First Nation communities of N’Dilo and Dettah. Although participants did express concerns about the consultation process and the viability of the proposed remediation plan, unresolved issues related to the operation of the original mine were the dominant focus of the discussions. Three main themes arose: 1) Fairness: participants felt their communities had incurred all of the risk while others received all of the benefits from the mine, and there should be appropriate compensation; 2) Loss: mining activities resulted in loss of land and traditional way of life; and 3) Fear: participants spoke of past incidents of arsenic poisoning and their fear for future generations. Overall, the results indicated that a lack of both shared values and confidence in government agencies underlies the lack of cooperation with the proposed remediation strategy, consistent with the trust, confidence and cooperation model (2) of risk communication. This research also demonstrated that communication cannot be successful unless unresolved historical issues are first recognized and addressed.

**References**

1. Jardine CG, Banfield L, Driedger SM, Furgal CM. Risk communication and trust in decision-maker action: a case study of the Giant Mine Remediation Plan. Int J Circumpolar Health. 2013;72:21184. doi: http://dx.doi.org/10.3402/ijch.v72i0.21184

2. Michael S, Zingg A. The role of public trust during pandemics: implications for crisis communication. Eur Psychol. 2014;19:23–32.

## Indigenous Health I: “Health Status overview”

## The Circumpolar Inuit Health Strategy 2010–2014: a strategic initiative to improve Inuit health and wellness across the Arctic

### Minnie Grey^1^, Percy Ballot^2^, Heather Dingman^3^, Leanna Ellsworth^4^, Eva Kruemmel^4^, Gert Mulvad^5^, Natan Obed^6^, Annmaree O’Keeffe^4^ and Galine Zagoruiko^7^
^1^Nunavik Regional Board of Health and Social Services, Québec City, Canada, minnie.grey@ssss.gouv.qc.ca; ^2^Maniilaq Association, Kotzebue AK, USA; ^3^North Slope Borough, Alaska AK, USA; ^4^Inuit Circumpolar Council (ICC), Ottawa, Canada; ^5^Greenland Centre for Health Research, Manutooq, Greenland; ^6^Nunavut Tunngavik Inc., Iqaluit, Canada; ^7^Central Chukotka Hospital, Anadyr, Russia

#### 

Inuit are indigenous peoples from four of the eight Arctic countries; in Alaska (US), Canada, Greenland and Chukotka (RU), and are represented internationally by the Inuit Circumpolar Council (ICC). Inuit health and well being is a policy priority for ICC. *The Circumpolar Inuit Health Strategy* was developed based on consultation and input from the Circumpolar Inuit Health Steering Committee, and participants from the Inuit Health Summit that was held in 2009 in Yellowknife just prior to ICCH14. The outcomes of the Inuit Health Summit were presented at ICCH14, and a more detailed paper was published as part of the proceedings in the *International Journal of Circumpolar Health* (EM Krümmel. The Circumpolar Inuit Health Summit: A Summary”, 68: 5, 2009). The Circumpolar Inuit Health Strategy’s overall objective is to improve Inuit health and wellness across the Arctic, through its five goals:

1. Influence international, regional and national policies and programs that impact on Inuit health and well being.
2. Improve awareness of Inuit health and wellness across the Arctic.
3. Encourage greater focus on Inuit health and wellness through ICC’s representation at international fora.
4. Support improved understanding by health professionals of Arctic/Inuit specific issues.
5. Promote research to improve Inuit health and wellness.

This presentation will provide an overview of work accomplished during the 2010–2014 Circumpolar Inuit Health Strategy. Mental health and wellness continues to be a priority for ICC, and new actions on health as determined by circumpolar Inuit representatives at the ICC General Assembly held in Inuvik, Canada, in July 2014 will also be presented.

## Funder Collaborative Model for on-the-land healing programs in the Northwest Territories: The importance of partnerships with Aboriginal governments

### Sabrina Broadhead^1^, Karen Blondin-Hall^1^, Debbie DeLancey^1^ and Steve Ellis^2^
^1^GNWT-HSS, Yellowknife, NT, Canada, debbie_delancey@gov.nt.ca; ^2^TIDES Canada, Vancouver, Canada

#### 

Aboriginal governments and communities in the Northwest Territories (NWT) have identified the need for on-the-land healing programs to address disparities in health and wellness. This comes at a time when there is increasing recognition, supported by research, that cultural identity is linked to improved mental health in Aboriginal communities. Until recently, on-the-land programs have been fragmented, siloed and housed within specific government departments with mandates and budgets. In response, the Government of the NWT partnered with a non-profit organization and the Aboriginal Leadership Initiative to host the NWT On-the-Land Funder Collaborative workshop in November 2014. Over 70 representatives from NWT Aboriginal governments, territorial and federal governments, major corporations and non-profit organizations came together to discuss how to strengthen on-the-land programs through partnership and coordination of resources in the form of a funder collaborative. Funder collaboratives are designed to address common issues faced by diverse funders across multiple sectors and have proven to deliver better outcomes in communities through the pooling of resources and the exchange of knowledge. In an effort to secure additional funding and support for on-the-land healing programs, the Department of Health and Social Services (HSS) identified the need to further research the health impacts of land-based activities. HSS is committed to listening and exploring solutions identified by communities who understand their health needs best. Meaningful partnerships with Aboriginal governments must serve as the foundation for any initiative related to Aboriginal health – from research to building health promotion programs. This presentation will speak to the collaborative process between the Government of the Northwest Territories (GNWT) and Aboriginal governments, the commitments made at the workshop, and the power of a shared vision towards improving health and well-being of Aboriginal people in the NWT.

## In the light of change: correspondence between observational data and perceptions of climate in northern Sweden – a mixed methods study

### Maria Furberg^1^, David Hondula^2^, Michael Saha^3^ and Maria Nilsson^1^
^1^Umea University, Umeå, Sweden, maria.furberg@climi.umu.se; ^2^University of Arizona, Tucson AZ, USA; ^3^University of Virginia, Charlottesville VA, USA

#### 

*Background*. Climate change (CC) is an extremely complex phenomenon that has been dominated by studies using quantitative numerical data like changes in temperature and precipitation. People all over the world have at times experienced and reported dramatic changes, experiences that are not as clearly visible in the numbers. *Aim.* Our objective was to explore weather data support the Swedish indigenous reindeer herders’ observations and perceptions of CC and whether there are any discrepancies. *Method.* We performed an exploratory sequential mixed methods study. Swedish Sami reindeer herders were interviewed about their experiences and perceptions of CC. From their experiences, four hypotheses were created and compared with 50 years of weather data from stations in the corresponding area. *Results.* The preliminary results converged for the following three hypotheses: 1. “increased winter temperature”; 2. “shorter and less frequent long, cold, stable periods in wintertime” and 3. “shorter snow cover season”. The fourth hypothesis, “rapid fluctuations in temperature are becoming more common,” could not be supported in the weather data. This phenomenon of unstable temperatures has been reported by many indigenous peoples from Arctic and tropical countries, but has never been possible to demonstrate using meteorological data. We suggest that this divergent result is indicative of a lower sensitivity in the statistical quantitative data compared to human perceptions. Unstable temperature might represent a hitherto, by triangulation unconfirmed, CC phenomenon. Our study illustrates that the qualitative and quantitative methods are complementary and supports the importance of methodological triangulation in CC research.

**Reference**

1. Furberg Maria, Evengård Birgitta, Maria Nilsson. Facing the limit of resilience: perceptions of climate change among reindeer herding Sami in Sweden. Glob Health Action. 2011;4:8417, doi: http://dx.doi.org/10.3402/gha.v4i0.8417

## Improving healthy living in the Northwest Territories through chronic disease prevention and screening (CDPS)

### Kami Kandola^1^, Vee Faria^1^, Donna Manca^2^, Carolina Aguilar^2^ and Nicolette Sopcak^2^
^1^Department of Health and Social Services, Government of the Northwest Territories, Yellowknife, Canada, kami_kandola@gov.nt.ca; ^2^University of Alberta, Edmonton, Canada

#### 

Seven out of ten deaths in the Northwest Territories (NWT) are related to chronic diseases, with cancer and cardiovascular disease accounting for approximately half the deaths. There is a demographic shift in our population, with significant increases in middle-age to elderly population. Modifiable risk factors for these three most common chronic diseases include unhealthy eating and obesity, physical inactivity, alcohol misuse and tobacco use. In the NWT, there was no coordinated approach to chronic disease prevention and screening prior to the adaption of the Building on Existing Tools to Improve Chronic Disease Prevention and Screening in Primary Care (BETTER 2) program. The main strategy used in this approach is internal practice facilitation through the use of a prevention practitioner. Because of gaps in preventative care, there was significant departmental interest in adapting the BETTER program. The desired goals of the program were improved clinical outcomes, reduction in the burden of chronic disease, and improved sustainability of the health-care system through improved CDPS in primary care. In the NWT BETTER program, evidence based screening guidelines, healthy living modules and public information resources were therefore developed and are available on-line through the Choose NWT Healthy Choices Framework website. Training was provided in all the regional health authorities with specific pilot projects in three communities, three diamond mines and Aurora College nursing program. Preliminary results of the impact of training will be available at the time of the conference.

**References**

1. Campbell-Scherer D, Rogers J, Manca D, Lang-Robertson K, Bell S, Salvalaggio G, et al. Guideline harmonization and implementation plan for the BETTER Trial: Building on Existing Tools to Improve Chronic Disease Prevention and Screening in Family Practice. CMAJ Open. 2014;2:E1–10.

2. Grunfeld E, Manca D, Moineddin R, Thorpe KE, Hoch JS, Campbell-Scherer D, et al. Improving chronic disease prevention and screening in primary care: results of the BETTER pragmatic cluster randomized controlled trial. BMC Fam Pract. 2013;14:175.

3. Manca DP, Aubrey-Bassler K, Kandola K, Aguilar C, Campbell-Scherer D, Sopcak N, et al. Implementing and evaluating a program to facilitate chronic disease prevention and screening in primary care: a mixed methods program evaluation. Implement Sci. 2014;9:135.

## DNA Methylation Patterns Are Associated With N-3 Fatty Acid Intake In Yup’ik People

### Stella Aslibekyan^1^, Howard Wiener^1^, Peter Havel^2^, Kimber Stanhope^2^, Diane O’Brien^3^, Scarlett Hopkins^3^, Devin Absher^4^, Hemant Tiwari^1^ and Bert Boyer^3^
^1^University of Alabama, Birmingham AL, USA; ^2^University of California, Davis CA, USA; ^3^University of Alaska, Fairbanks AK, USA, bert.boyer@gmail.com; ^4^Hudson Alpha Institute for Biotechnology, Huntsville AL, USA

#### 

A large body of evidence links a high dietary intake of n-3 (ω-3) polyunsaturated fatty acids (PUFAs) with improved cardiometabolic outcomes. Recent studies suggested that the biologic processes underlying the observed associations may involve epigenetic changes, specifically DNA methylation. To evaluate changes in methylation associated with n-3 PUFA intake, we conducted an epigenome-wide methylation association study of long-chain n-3 PUFA intake and tested associations between the diabetes- and cardiovascular disease-related traits. We assessed DNA methylation at ~470,000 cytosine-phosphate-guanine (CpG) sites in a cross-sectional study of 185 Yup’ik Alaska Native individuals representing the top and bottom deciles of PUFA intake. Linear regression models were used to test for the associations of interest, adjusting for age, sex and community group. We identified 27 differentially methylated CpG sites at biologically relevant regions that reached epigenome-wide significance (P<1×10^−7^). Specifically, regions on chromosomes 3 (helicase-like transcription factor), 10 (actin α 2 smooth muscle/Fas cell surface death receptor) and 16 (protease serine 36/C16 open reading frame 67) each harbored 2 significant correlates of n-3 PUFA intake. In conclusion, we present promising evidence of association between several biologically relevant epigenetic markers and long-term intake of marine-derived n-3 PUFAs.

## Health Research in Nunavut: An Inuit Governance Perspective

### Natan Obed and Sharon Edmunds-Potvin
Nunavut Tunngavik Inc., Iqaluit, Canada, sedmunds-potvin@tunngavik.com

#### 

Research in Nunavut has a tainted legacy, characterized by uneven power relationships between Inuit and researchers. While great strides have been made in the area of participatory research, aspects of this legacy are still present in research occurring in the Nunavut Settlement Area. Further, research is increasingly viewed as an important means of exercising Inuit self-determination, by communities and institutions of Inuit governance in Nunavut – this statement holds true for Nunavut Tunngavik Incorporated (NTI), the holder of the Nunavut Land Claims Agreement. Research has the ability to positively impact the lives of Inuit in Nunavut, particularly when research results meaningfully inform interventions and policy. The latter, from an Inuit governance perspective, is best achieved through co-driven research that is participatory in nature. Essentially when Inuit institutions, including governments, are invested in the research question(s) and co-shaping the research with academic partners our entire society can benefit. A new research reality is emerging in Nunavut with strong emphasis being placed on meaningful engagement – this includes the conceptualization phase and ranges to the dissemination of results and formulating the narrative.

## Disparities in infant mortality, stillbirth, and preterm birth among First Nations, Inuit and Métis populations in Canada

### Amanda J. Sheppard^1^, Seungmi Yang^2^, Tracey Bushnik^3^, Mourad Dahhou^2^, Serenity Perry^4^, Jay S. Kaufman^2^, Russell Wilkins^5^ and Michael Kramer^2^
^1^The Hospital for Sick Children, Toronto, Canada, amanda.sheppard@sickkids.ca; ^2^McGill University, Montréal, Canada; ^3^Statistics Canada, Ottawa, Canada; ^4^Ontario Native Women’s Association, Thunder Bay, Canada; ^5^University of Ottawa, Ottawa, Canada

#### 

*Introduction.* The term “Aboriginal” comprises three constitutionally recognized populations in Canada: First Nations, Métis and Inuit; each with unique cultural identities, social determinants of health and health care funding inequities. Data on birth outcomes in Canada have been limited due to the lack of Aboriginal birth identifiers on birth registrations in most provinces and territories, and almost entirely lacking for Métis. Based on data of limited quality, birth outcomes have been reported to be significantly poorer among Aboriginal peoples compared to their non-Aboriginal counterparts and suggest disparities among the three Aboriginal groups. *Objective.* To compare risks of infant mortality, stillbirth and preterm birth (<37 completed weeks) among the three Aboriginal populations. *Methods.* We analysed a cohort of births between May 2004 and May 2006 created by linking the Canadian perinatal health database with the long form of the 2006 Canadian census, which includes an Aboriginal self-reported identifier. *Results.* The crude infant mortality rate for the overall Aboriginal population was 9.8 (8.3, 11.4) per 1,000, and 8.6 (7.1, 10.2), 10.8 (6.6, 15.1) and 11.9 (6.8, 17.0) per 1,000 live births among First Nations, Métis and Inuit, respectively. For stillbirths, the corresponding rates were 8.9 (7.5, 10.4) per 1,000 total births overall and 9.2 (7.6, 10.8), 6.1 (2.9, 9.2) and 8.5 (4.2, 12.8) in the three groups. Preterm birth rates were 99.1 (94.4, 103.7) per 1,000 total births overall and 96.0 (91.1, 101.0), 81.6 (70.4, 92.8) and 124.3 (108.8, 139.8) in the three groups. *Conclusions.* The pan-Aboriginal approach to birth outcome reporting in Canada has masked substantial disparities across Canada’s three Aboriginal populations. The data demonstrate the need for targeted maternal and infant health programs to reduce adverse birth outcomes in these populations.

## Indigenous Health II: “Mental Health and Bereavement”

### Mapping Inuit mental health and wellness on the atlas of community-based monitoring (CBM) and traditional knowledge in a changing Arctic

#### Leanna Ellsworth^1^, Laura Petrunka^1^, Noor Johnson^2^, Eva Kruemmel^1^ and Peter Pulsifer^3^
^1^Inuit Circumpolar Council (ICC), Ottawa, Canada, lellsworth@inuitcircumpolar.com; ^2^Institute for the Study of Environment and Society, Brown University, Providence RI, USA; ^3^Exchange for Local Knowledge and Observations of the Arctic (ELOKA), National Snow and Ice Data Centre, Boulder CO, USA

##### 

Inuit are indigenous peoples from four of the eight Arctic countries, in Alaska (US), Canada, Greenland and Chukotka (RU), and are represented internationally by the Inuit Circumpolar Council (ICC). Inuit health and well-being is a policy priority for ICC. A part of this work is to document the different health and wellness experiences of Inuit and to advocate for solutions within international forums. In 2012, ICC completed a report on *Circumpolar Inuit Health Priorities: Best Health Practices and Research*. This report documents and assesses a comprehensive range of best practice programs and studies that have been implemented across the Arctic in the main health areas of service delivery, chronic disease, food security and mental health and wellness. Each of the four countries has responded differently in terms of effort or investment of resources applied to a particular health area. The concluding recommendations of the report stated the importance to share this information more broadly via a clearinghouse, and the need for better integration of Inuit health related information. Since Inuit health and wellness continues to be a priority for the ICC, ICC-Canada with its partners developed an online map of the Inuit mental health and wellness programs based on the 2012 ICC report on *Circumpolar Inuit Health Priorities: Best Health Practices and Research*, as well as new programs identified in 2014. The Inuit mental health and wellness map is housed on the CBM Atlas, which maps CBM and traditional knowledge projects and has expanded to include community-based health projects. This map allows users to search by keyword and to quickly identify programs that are relevant to specific regions or communities, and to learn how Inuit are addressing the challenges and finding solutions with mental health and wellness. This presentation will provide an overview and demonstration of the Inuit mental health and wellness map.

## Experiences of Psychiatric Care among Young Sami in Northern Sweden

### Lars Jacobsson^1^, Anna Fagerström^2^, Laila Daerga^2^ and Anette Edin-Liljegren^2^
^1^Department of Psychiatry, Arcum, Umeå university, Umeå, Sweden; ^2^Centre for Rural Medicine, County Council of Västerbotten, Umeå University, Umeå, Sweden, anette.liljegren@vll.se

#### 

Earlier studies have shown that young Sámi people feel a vulnerability because of experiences of ethnic discrimination and the constant need to explain and defend their existence as a Sami and the Sami culture. Anxiety and stress are more common among young Sami compared with Swedish youth, and Sami girls and young women report more symptoms and have a poorer self-esteem, than Sami boys (1). There are subgroups among the Sami adolescents who report more experiences of suicidal plans and suicide attempts, which is worrying. Young reindeer-herding men report a sense of powerlessness. Other studies among both Norwegian and Swedish Sami show that men and women have a lower confidence for personnel in primary health care, psychiatry and social services (2). This can lead to a lower frequency of seeking care early, resulting in disorders becoming more difficult and more costly to treat and the individual has to suffer unnecessarily. Therefore, the Sami have not to the same extent as the majority population, access to health care on equal terms. A study among Sami and Norwegian youth shows that there are culture-specific factors affecting the help-seeking process (3). The experiences of seeking care for mental health problems among the Sami youth in Sweden is unknown. This study will focus on young Sami people’s experience of seeking care and getting treatment in psychiatry in Sweden. This project will be performed as a qualitative study with semi-structured interviews among Sami adolescents, 15–30 years of age. The interviews will be transcribed and the text analysed using content analysis with a phenomenological approach. The selection of informants is supposed to reflect the Sami youth in Sweden, with experiences of mental health care. The adolescence will further be selected based on gender, geographical area and by various Sami groups such as reindeer herding and non-reindeer herding Sami. Preliminary results from this study will be presented at the conference.

**References**

1. Omma L, Jacobsson LH, Petersen S. The health of young Swedish Sami with special reference to mental health. Int J Circumpolar Health. 2012;71:18381. doi: http://dx.doi.org/10.3402/18381

2. Daerga L, Sjölander P, Jacobsson L, Edin-Liljegren A. The confidence in health care and social services in northern Sweden – a comparison between reindeer-herding Sami and the non-Sami majority population. Scand J Public Health. 2012;40:516.

3. Turi AL, Bals M, Skre IB, Kvernmo S. Health service use in indigenous Sami and non-indigenous youth in North Norway: a population based survey. BMC Public Health. 2009;9:378, doi: http://dx.doi.org/10.1186/1471-2458-9-378

## Sudden and unexpected death in Sámi areas in Norway – a qualitative study of the significance of religiosity in the bereavement process

### Anne Silviken^1,2^, Lena Gundersen Slettli^1^, Gro Berntsen^3^ and Kari Dyregrov^4,5,6^
^1^Centre for Sami Health Research, University of Tromso – The Arctic University of Norway, Tromso, Norway, anne.silviken@uit.no; ^2^Sámi Norwegian National Advisory Unit on Mental Health and Substance Use, Karasjok, Norway; ^3^Northern Norway Violence, Traumatic Stress and Suicide Prevention Resource Centre, University Hospital of North Norway, Tromsø, Norway; ^4^Norwegian Institute of Public Health, Division of Public Health, Oslo, Norway; ^5^Bergen University College, Bergen, Norway; ^6^Center for Crisis Psychology, Bergen, Norway

#### 

*Background.* Sudden and unexpected death represents a severe life event incorporating multiple stressors and is potentially more traumatizing than natural deaths. Religiosity is an important resource in everyday life and may be especially important during times of loss. *Objectives.* The aim of this presentation is to explore whether and how religiosity and folk religiosity are important in the coping process after sudden and unexpected death in Sámi areas in Norway. *Method.* The data are part of “The North Norwegian Bereavement Study” consisting of two samples, “the community sample” and “the bereaved sample”. Both quantitative (self-administered questionnaire) and qualitative methodology (in-depth interviews) were applied, and this presentation concerns the findings from the latter sample. A hermeneutic phenomenological research method was applied, using a semi-structural in-depth interview guide to investigate the experiences of 30 bereaved people from different Sámi areas in Northern Norway. *Results.* Three major themes of importance for religious coping were found: rituals, after death communication, and signs and warnings. The findings will be discussed in relation to: 1) the pre-Christian Sámi worldview and 2) the function of a safe place to grieve and the significance of accepting death. *Conclusions.* Religiosity may be a great resource of help in the grieving process of the bereaved, and especially coping strategies based on local culture as it is integrated in everyday life. It is important that health care personnel are culturally sensitive and acknowledge the experience and significance of religiosity in the bereavement process.

**Reference**

1. Silviken A, Gundersen LS, Berntsen G, Berntsen K. (in press). Sudden and unexpected death in Sámi areas in Norway – a qualitative study of the significance of religiosity in the bereavement process. Suicidology online.

## Challenges in the encounter with local support services for bereaved after sudden and unexpected death in Sami areas in northern Norway

### Gro Berntsen^1^, Anne Silviken^2^ and Kari Dyregrov^3,4^
^1^Resource Centre on Violence, Traumatic Stress and Suicide Prevention, University Hospital of North Norway, Tromsø, Norway, gro.berntsen2@unn.no; ^2^Centre for Sami Health Research, University of Tromso – The Arctic University of Norway, Tromsø, Norway; ^3^Center for Crisis Psychology, Bergen, Norway; ^4^The Norwegian Institute of Public Health, Oslo, Norway

#### 

This presentation is based on a study of the situation of traumatic bereaved from indigenous Sámi areas in the North of Norway. The main aim of the study was to secure appropriate follow-up for bereaved after traumatic deaths in multi ethnic areas of North Norway, with a special focus on indigenous Sami bereaved. Thus, the study aimed at getting specific and local knowledge of their psychosocial situation and bereavement process, their needs for help, and examine whether they seek and get help from the health care system when needed. The North Norwegian Bereavement Study included both qualitative and quantitative data. The total sample consisted of 182 close bereaved after unnatural/violent deaths (suicides, accidents, SIDS and murders, RR 75%) from northern Norway. A majority of the bereaved sample had lost their children, siblings and partners by suicide (52%). From the bereaved that consented to be interviewed, 34 were invited to participate. Thirty-one of the latter agreed to take part in in-depth interviews. The presentation focuses on challenges experienced by bereaved after sudden and unexpected death in different Sámi areas in northern Norway in the encounter with local support services. The qualitative content analysis revealed three main groups of challenges: 1) tight and multiplex relations, 2) lack of confidence in the local support services and 3) lack of flexibility and availability of local support services. These challenges are discussed in view of the cultural context and relevant theory. The results showed that a cultural sensitive support service demands knowledge and understanding of the bereaved’s situation and the cultural context that he or she lives in.

## Provided assistance from local support services after sudden and unexpected death in Northern Norway (in progress)

### Marianne Larssen^1^, Kari Dyregrov^2,3^, Gro Berntsen^1^ and Anne Silviken^4^
^1^Resource Centre on Violence, Traumatic Stress and Suicide Prevention, University Hospital of North Norway, Tromsø, Norway, marianne.larssen@unn.no; ^2^Center for Crisis Psychology, Bergen, Norway; ^3^The Norwegian Institute of Public Health, Oslo, Norway; ^4^Centre for Sami Health Research, Institute of Community Medicine, University of Tromso - The Arctic University of Norway, Tromsø, Norway

#### 

*Background.* This presentation is based on data from the North Norwegian Bereavement Study. The main aim of the study was to secure appropriate follow-up for bereaved after traumatic deaths in multi-ethnic areas of Northern Norway, with a special focus on bereaved from Sami areas. Thus, the study aimed at getting specific and local knowledge of bereaved psychosocial situation and bereavement process, their needs for help, and examine whether they seek and get help from the local support services when needed. Another important aim of the study was to explore what kind of help local support services in Northern Norway offered bereaved after sudden and unexpected death. The presentation focuses on describing: 1) the type of assistance provided from local support services, 2) the caregivers, 3) how contact is established and 4) follow-up time. *Method and sample.* The study includes data from both bereaved and municipalities. The Municipality Study included quantitative data. The presentation is based on data from the 58 northern Norwegian municipalities in the sample (57% response rate). *Results.* The preliminary results show differences in provided assistance from local support services according to organizing factors and to what degree the local support services are able to meet the reported needs of bereaved. The results are discussed in light of demographic and organizing factors (e.g. written routines, coordinator and whether they have a crisis outreach and support team) and in light of the needs reported by bereaved: 1) early help, 2) proactive help and 3) flexible and available help. *Preliminary conclusion.* The local support services in Northern Norway offers a wide range of assistance to bereaved. Measures taken to organize the psychosocial safeguarding of bereaved seem to have a positive effect on meeting the needs reported by bereaved.

## Indigenous Health III: “Life Spectrum and Relationships”

### “Makimautiksat”: building a foundation within one’s self: an evidence-based youth wellness and empowerment intervention for Nunavut youth

#### Ceporah Mearns and Gwen Healey
Qaujigiartiit Health Research Centre, Iqaluit, Canada, ceporah.mearns@qhrc.ca

##### 

*Background.* Nunavummiut experience the highest rate of suicide in Canada. Nunavut is in need of preventive child and youth mental health and wellness interventions that focus on northern and community-based ways of understanding healthy children and youth. Community/land-based youth health summer camps have been delivered in Nunavut in the past, but facilitators reported a desire for more materials that are grounded in Inuit ways of knowing and understanding wellness. *Method.* Interviews with community partners/programs, community members, service providers, youth and a literature review, contributed to the development of Qaujigiartiit’s evidence-based Eight Ujarait/Rocks Model for a 2-week youth wellness and empowerment camp program. The model focuses on eight pillars of wellness for Nunavut youth such as, building healthy relationships, strengthening coping skills, making informed choicesand celebrating Inuit culture, to name a few. This model was piloted six times in 2011–2013 as the Makimautiksat Youth Wellness and Empowerment Camp. *Results.* Approximately 50 adolescents between the ages of 11 and 15, and 10 youth mentors between the ages of 16 and 19, participated in the Makimautiksat camp pilots in five Nunavut communities between June and August 2011–2013. Pre- and post- intervention data collection revealed an improvement in self-reported wellness, self-esteem, ability to cope, pride in Inuit culture and confidence in ability. Pre- and post-intervention data collection with facilitators and parents identified a strong connection to the material covered during the camp and noticeable improvements in behaviour and attitude among the youth who participated. *Conclusion.* Child and youth wellness interventions developed by and for Nunavummiut that focus on northern and community ways of knowing and learning about wellness are critical for building confidence and self-esteem among Nunavut youth and, ultimately, will play a critical role in suicide prevention efforts.

## Sami elders – two films about how to manage life in old age in the Artic

### Gunn Minde
Harstad University college, Harstad, Norway, gunn.minde@hih.no

#### 

After a short introduction why I choose making a film, I will show two films Between Past and Present” and Home and Cultural Landscape – A Space for Rehabilitation. The films is about elders from northern Norway from the coastal area. They are both Sami indigenous people. Reidun is 91 years old, and is living with her son Ralfh. She tells in her own voice what is important for her to maintain good health. Trygve is 77 years old and is living alone. He is still a fisherman, and has his own boat. He has suffered from stroke, and his motivation to recover, is to continue living as a fisherman. What he has done to recover, he tells us in this film; both films are in 10 minutes each. The overall goal is to demonstrate how older indigenous people live and recover within their own social and cultural environment after being at the hospital/rehabilitation centre.

**References**

1. Ann Mari Andersen, University of Tromsø, Department of Social work, Campus Alta.

2. Janne Engenes, University of Tromsø, Department of nursing, Campus Hammerfest.

3. Ketil L. Hansen, University of Tromsø, Faculty of Health Sciences, Dep. of Community Medicine.

## Visual graphics as a tool for communication and relationship building

### Melody Morton Ninomiya
Memorial University, St John’S, Canada, melodym@mun.ca

#### 

Ethical and authentic relationships between (indigenous and non-indigenous) researchers and indigenous peoples are, in part, facilitated through effective and appropriate communication. In this poster, I illustrate and describe how researchers can improve communication of information through the use of visual graphics throughout all research phases. Visual graphics such as graphs, charts, figures and photographs are common in scientific research (especially dissemination) materials however they are less common as communication tool in research. Using examples from my own community-based health research project with a rural indigenous community in eastern Canada, I demonstrate how visual graphics made communication of information both accessible and engaging with community members, research informants and (indigenous and non-indigenous) government stakeholders at different phases of research. While I advocate that researchers use visual graphics to enhance and improve communication and build authentic relationships, I also argue that researchers must carefully consider how visual graphics are used and what they represent.

## Working in the Circumpolar Regions: Accidents and Injuries (Session I)

## Thermal responses of workers in Arctic open-pit mines in the winter

### Hannu Rintamäki^1,2^, Sirkka Rissanen^1,2^, Kirsi Jussila^1,2^, Juha Oksa^1,2^ and Satu Mänttäri^1,2^
^1^Finnish Institute of Occupational Health, Helsinki, Finland; ^2^University of Oulu, Oulu, Finland, satu.manttari@ttl.fi

#### 

The unique working environment of mining in Arctic region, characterized by exposure to cold and windy conditions, sets out challenges to maintain miners capacity for work. The aim of this study was to evaluate the thermal responses of miners in cold work in Arctic open-pit mines. A questionnaire and a field study were carried out in Kevitsa (Finland) and Aitik (Sweden) mines among workers with duties consisting mainly of outdoor work. Ambient (Ta), core (Tcore) and skin temperatures, dry heat loss and thermal insulation of the clothing were measured during typical work shift at a temperature range of +20°C to −18°C and wind speed up to 4 m/s.The questionnaire study revealed that only very few sensations of cold appear at Ta above −10°C. At −20°C to −10°C, ca. 25% of the workers had cold thermal sensation of fingers and toes, and 11% in the whole body. Tcore showed an increasing trend during the work shift. On the contrary, a positive linear correlation between peripheral skin temperatures and Ta was found. Of the total working time spent outside, the proportion of mean skin (Tsk) and finger temperatures below the limit values indicating marked performance decrement (30°C and 15°C, respectively) were 26 and 21%. Outdoors, finger and toe temperatures typically decreased at first with a rate of 0.57±0.04 and 0.14±0.02°C/min, but after ca. 30 min the temperatures started to recover. Indoors, it took 20 and 30 min to rewarm fingers and toes, respectively. Heat loss was the highest in the calf (246±55 W/m2). Wind lowered the effective thermal insulation of clothing by 18–30%.To conclude, the exposure to cold working environment causes apparent thermal responses in miners. Based on skin temperature data, both whole body and manual performance were degraded for ca. quarter of the work shift indicating an apparent need for improved thermal protection, especially of hands and feet, in cold sensitive workers and in tasks with high cold stress. This study is funded by Kolartic ENPI.

## Work in the cold: how much we can trust our thermal sensations?

### Hannu Rintamäki, Sirkka Rissanen, Kirsi Jussila, Juha Oksa, Pertti Tuhkanen and Satu Mänttäri
Finnish Institute of Occupational Health, Oulu, Finland, hannu.rintamaki@ttl.fi

#### 

Messages from the thermoreceptors in the skin are delivered to the hypothalamus, which guides the autonomic responses. The same stimulus creates also the thermal sensations in the cortex of the brain motivating to changes in behavior which affects heat loss or heat production. The aim of the present study was to find out the thermal sensations of the workers in open pit mines and how the sensations are related to the measured skin temperatures. A questionnaire study collecting information from the thermal sensations of workers was carried out in the Kevitsa open pit mine in Finland. Moreover, in the field study the thermal sensations (general, face, finger and toe) were recorded together with core and skin temperature measurements in Kevitsa and Aitik (Sweden) open pit mines among 14 male and 2 female mine workers with duties consisting mainly of outdoor work. Altogether, 199 workers responded to the questionnaire. Only very few sensations of cold appear at ambient temperatures (Ta) above −10°C. At −20°C to −10°C, about 25% of the workers had generally cold thermal sensation of fingers and toes, and 11% in the whole body. In the 18 field measurements (duration 2 h 41 min–7 h 34 min, Ta −3°C–−16°C, wind velocity up to 4 m/s) the thermal sensations ranged from hot to cool in whole body level, warm to cool in face and warm to cold in fingers and toes. A statistically significant correlation between skin temperatures and corresponding thermal sensations was found. However, there was a very wide variation in this relationship. In conclusion, the relationship between skin temperatures and thermal sensations shows that the workers were well adapted to cold. The proportion of cold thermal sensations suggest a need for improved thermal protection of hands and feet in cold sensitive workers and in tasks with high cold stress. This study is part of the MineHealth project funded by Kolartic ENPI CBC.

## Gender transition in the Swedish mining industry – challenges and opportunities

### Anita Pettersson-Strömbäck
Umeå University, Umeå, Sweden, anita.p.stromback@envmed.umu.se

#### 

*Introduction.* Traditionally, mining is a working place dominated by men. But in recent years, especially in Sweden, a greater number of women have been recruited. According to IF Metall (the trade union that enrolls mine workers in Sweden), the proportion of women in the mining industry has increased from 8.9% in 2009 to 13.7% in 2013. This development is very positive from a equality perspective, but could implicate challenges as the mining industry traditionally is characterised by what researchers calls a “macho masculinity”. The effect could be differences in psychosocial and socioeconomical outcomes. In order to investigate that question, a substudy in the bigger MineHealth (financed by EU, Kolarctic ENPI CBC, www.minehealth.eu) in the Boliden mine in Aitik, Gällivare municipality, was performed. *Method.* Several data sources were used. A survey was performed at the Aitk mine where 153 mine workers (96 men, 57 women) answered questions about perceived stress, sleep and health. Interviews with stakeholders were performed probing the respondent’s view of future challenges in the region, and statistical data about socioeconomical variables were gathered from National Statistics, Sweden. *Results:* The results indicate that in the age group between 18 and 40, there are more women (53%) then men (47%) employed in the mine. Perceived high stress in the work environment is more common among female workers (Pearson chi-square 0.007). Women perceive their present work in the mine as more positive in relation to their previous work than men do (Pearson chi-square 0.03). All interviewed stakeholders state that the employment of women in the mine is positive. Statistical data indicate that there are more women in the municipality with tertiary education. *Conclusions.* When women enter the mining industry, the work environment needs to adapt in order to fit both sexes. The male role is changing, which can cause a lack of identity, distress and health problems.

## A field evaluation of the thermal responses of wilderness guides and tourists in northern Finland during winter season

### Kirsi Jussila and Sirkka Rissanen
Finnish Institute of Occupational Health, Oulu, Finland, sirkka.rissanen@ttl.fi

#### 

In northern countries winter tourism is an important business which involves large amount of people from all around the world. Most of the tourists come to northern tourist resorts from countries where climate is warmer. Cold is therefore a new experience for many of them. Moreover, winter clothing may be unfamiliar and the knowledge of what and how to dress in winter might be insufficient. Wilderness guides are exposed to the same environment as their customers. On the other hand, guides’ thermal responses and sensation may differ from those of their customers in several reasons. The aim of the study was to evaluate thermal responses and cardiovascular strain of wilderness guides and tourists during snowmobile safari and snowshoe walking. Three guides and four customers participated in two snowmobile safaris (SM) and five male costumers in the snowshoe walking (SW). Ambient temperature was 15°C–31°C. Core and skin temperatures and heart rate (HR) were measured. SM lasted for 3 and 7 hours including breaks and SW for one hour. Mean skin temperature (Tsk) was 2°C –3°C lower for the guides than for the customers during SM. Thermal sensations were colder for the customers. Mean HR was 130 and 110 bpm for the guides and customers, respectively during SM. The customers wore on an average one layer more clothing than the guides. Core temperature of customers increased above 38°C due to the physical activity during SW. Mean decrement of Tsk was 1.7°C/h, with great individual variation. Mean HR was 120 bpm, occasionally over 150 bpm during SW. The wilderness guides were acclimatized to cold, were physically more active and able to adjust the thermal protection according to physical activity and ambient conditions in comparison to the customers during SM. Decrement in Tsk was mainly caused by the decrease of skin temperatures in legs and hands. Guidance of clothing (what and how), especially for customers, is needed for different kind of activities in cold winter conditions.

## Muscular, cardiorespiratory and thermal strain of mast and pole workers

### Juha Oksa, Sanna Hosio, Harri Lindholm, Hannu Rintamäki, Sirkka Rissanen and Panu Oksa
Finnish Institute of Occupational Health, Oulu, Finland, juha.oksa@ttl.fi

#### 

Mast and pole work is defined as erecting and pulling down masts and poles, putting up and taking down antennas, installing and transposing air traffic guiding lights, installing pipes and cables and carrying out their maintenance. Since physical strain during mast and pole work is not known this study evaluated the level of muscular, cardiorespiratory and thermal strain of mast and pole workers with special emphasis on winter. Fourteen voluntary male mast and pole workers participated in the study. We measured their muscular strain using electromyography, expressed as percentage in relation to maximal EMG activity (%MEMG). We estimated VO_2_ from HR measured during work (using individual VO_2_–HR relationship) and expressed it as%VO_2_max. To quantify thermal strain skin and deep body temperatures were measured using temperature sensors and telemetric pill and receiver. We found the highest average muscular strain in the wrist flexor (24±2%MEMG) and extensor (21±1%MEMG) muscles, exceeding the recommendation of 14%MEMG. Average cardiorespiratory strain was 48±3% VO_2_max. Nearly half (40%) of the subjects exceeded the recommended 50% VO_2_max. Winter condition increased both muscular and cardiovascular strain on average by 4 and 2%, respectively. Deep body temperature varied between 36.8°C and 38.0°C and mean skin temperature between 28.6°C and 33.4°C indicating possible occasional superficial cooling. Cooling was most pronounced in extremities during winter. Lowest single temperatures in middle finger, hand and big toe varied between 6.4°C and 18.5°C, 9.4°C and 24.9°C, and 15.4°C and 24.6°C, respectively. In conclusion, this field study shows that workers may be at risk for local and/or systemic muscular and cardiorespiratory overloading (the winter enhancing this effect slightly) and thus for excessive fatigue, reduced work efficiency and increased risk for musculoskeletal symptoms. Generally, thermal strain remained at a tolerable level.

**Reference**

1. Oksa J, Hosio S, Mäkinen T, Rintamäki H, Rissanen S, Latvala J, et al. Muscular, cardiorespiratory and thermal strain of mast and pole workers. Ergonomics. 2014;57:669–78.

## Distance education for nurses working in remote Regions of northern Quebec:does it influence their sense of workplace empowerment?

### Norma Ponzoni, Antonia Arnaert and Susanne Lajoie
McGill University, Montréal, Canada, norma.ponzoni@mcgill.ca

#### 

In northern Quebec, diagnostic and curative care is provided by nurses working in nursing stations in remote and isolated communities. Despite the need for continuing education to support their expanded scope, these nurses have reduced access to peer support within their domain and professional development which therefore may impact their ability to ensure that their practice is based on current evidence. Advances in telecommunication technology today offer the opportunity to overcome the barriers of professional isolation and serve as a vehicle for the creation of communities of practice. Little is known about the understanding of how ICT may support nursing practice in Quebec. Therefore, this study, which used a mixed method approach, examined the impact of a distance-delivered continuing education program on nurses’ sense of workplace empowerment. A purposive sample of 40 participants were recruited through the “Centre d’Education et Formation à Distance” (CEFD) network of community health organizations. The outcome variable, workplace empowerment, was measured using Laschinger Workplace Empowerment Questionnaire (1) and semi-structured phone interviews were conducted to further explore their perceptions regarding their current workplace environment and how the developed continuing education program contributed to supporting their practice. Descriptive statistics were used to analyse the quantitative data; and content analysis methodology was used to analyse interview transcribed data. The main themes that emerged from the qualitative data were: exhilaration from professional challenges; comradery among nursing peers; distance education, a lifeline to competent practice; and managerial disconnect. Within the challenging context of northern practice, peer support and distance education were the factors that contributed to satisfaction and a feeling of workplace empowerment.

**Reference**

1. Laschinger HK, Finegan J, Shamian J. Impact of structural and psychological empowerment on strain in nursing work setting: expanding kanter’s model. J Nurs Admin. 2001;31:260–72.

## Working in the Circumpolar Regions: Accidents and Injuries (Session II)

### Psychological risks management of shift workers in the circumpolar regions andthe Arctic

#### Yana Korneeva^1^, Natalia Simonova^1^ and Galina Degteva^2^
^1^Northern (Arctic) Federal University named after MV Lomonosov, Northern State Medical University, Arkhangelsk Oblast, Russia, amazonkca@mail.ru; ^2^Northern State Medical University, Arkhangelsk, Russia

##### 

Arctic regions are characterized by extreme climatic factors and conditions of life (group isolation, remoteness from the major industrial centers, resource-intensive economic activities and livelihoods of the population, high labor intensity) (1). So shift method of work organization is used in these areas. Professional activities of shift workers in the Arctic have high requirements for the creation of conditions for the physiological, psychological and socio-psychological adaptation (2). It’s annoying inner resources, leads to a decrease in efficiency and reduction in psychological well-being of workers in these extreme conditions. According to the report of the International Labour Organization psychosocial factors is now recognized as an issue of global importance, relevant for all countries, professions and all employees. The growing number of disorders associated with stress at work, due to the proliferation of flexible and precarious forms of employment, an increase in the intensity of labor. As a result of our studies psychological risks in professional work shift workers in the Arctic defined. We have developed a technology to reduce these risks. This is the most preferred and most common direction, which involves preventive measures of psychological support of shift personnel. To optimize performance, we offer the following technologies: the selection of a working group on the basis of psychological compatibility; conduct relaxation exercises to remove emotional stress; training in self-control and conflict resolution; development of communicative competence; development of individual recommendations for adaptation; conducting individual psychological consultations on adaptation and professional activities; testing of skills to respond to emergency situations; improving vocational education.

**References**

1. Korneeva Ya, Simonova N. Professional qualities of fly-in-fly-out workers in oil and gas companies in the Arctic (Australian science review). 2014;2;488–99.

2. Korneeva Ya.A, Simonova NN, Degteva GN. Determination of professional fitness of shift workers in accordance with the phase assessment of environmental factors in the Far North (Society of Petroleum Engineers – SPE Arctic and Extreme Environments Conference and Exhibition). 2013;3:2558–89.

## Evaluation of cold protective clothing and its use in Arctic open-pit mines

### Kirsi Jussila^1^, Sirkka Rissanen^1^, Pertti Tuhkanen^1^, Juha Oksa^1^, Satu Mänttäri^1^ and Hannu Rintamäki^1,2^
^1^Finnish Institute of Occupational Health, Oulu, Finland, kirsi.jussila@ttl.fi; ^2^Institute of Biomedicine, University of Oulu, Oulu, Finland

#### 

Working in Arctic open-pit mines requires protection against extreme cold temperatures and high wind speed, and fluent adjustability of the protective clothing. Work load fluctuates between light and heavy causing occasional moisture accumulation into clothing due to sweating. Work environment creates a need for frequent washing of clothing. This study aimed to evaluate the selection criteria for open-pit miners’ winter clothing and its thermal protection at work, and to identify the effects of use on the clothing physiological properties. A questionnaire study evaluating open-pit miners’ cold experiences and the use of clothing in different ambient conditions was performed in three different open-pit mines in Finland, Sweden and Russia (n=1,104). A field experiment was carried out to determine thermal insulation and users’ experiences at work in two open-pit mines in northern Finland and Sweden. Thermal protective properties of clothing ensembles from the mines were compared in controlled laboratory measurements by using thermal manikin. The questionnaire study showed that clothing was selected based on cold exposure time, work load, environmental conditions and individual sensitivity to cold. The field experiment revealed that clothing was not adjusted according to ambient temperature (−3 to −12°C), the clothing thermal insulation between torso and legs was imbalanced and clothing was often experienced wet mostly due to sweating. Wind lowered effective thermal insulation by 18–30%. Use and washes of the clothing decreased thermal insulation by about 20%. In conclusion, the cold protective clothing provided sufficient protection at the studied ambient conditions. On the other hand, improvement of lower body thermal protection, development of the adjustability of the clothing, and increased training of optimum protective methods are needed for improved protection in the Arctic mining. This study was financed by the European Union, Kolarctic ENPI CBC.

## Epidemiology of injury mortality by intent in Labrador and Newfoundland, 1993–2009

### Nathaniel Pollock^1^, Jennifer Woodrow^2^, Michael Jong^3^, Shree Mulay^2^ and James Valcour^2^
^1^Labrador Institute, Newfoundland and Labrador, Canada, nathaniel.pollock@med.mun.ca; ^2^Memorial University, St John’s, Happy Valley-Goose Bay, NL, Canada; ^3^Labrador Grenfell Regional Health Authority, Happy Valley-Goose Bay, NL, Canada

#### 

*Background.* Injuries, including suicide and accidents, are leading causes of death among Aboriginal children and youth in Canada, especially those in northern communities. Rural and northern populations appear to be at elevated risk for injuries associated with transportation, especially in off-road vehicles, and employment, in industries such as mining and fishing. Our objectives were to describe the epidemiology of injury fatalities by injury intent and identify regional differences. *Methods.* We examined the patterns of injury mortality in Labrador and Newfoundland, Canada by injury intent, region, and over time. We analysed provincial vital statistics from 1993 to 2009, which included data about the cause of death by ICD code, age, sex, place of residence and date of death. This dataset did not include Aboriginal identifiers, so we used “geozones” as proxies for Labrador’s three indigenous populations. For our preliminary analysis, we calculated proportionate mortality by injury intent, region, sex, age and season.*Findings*. The majority of injury deaths in the province were due to unintentional accidents, though Labrador has a much higher percentage of injury mortality (15.96%) than Newfoundland (4.11%). The majority of injury deaths in four of Labrador’s subregions were due to unintentional injuries such as motor vehicle collisions or fires. The exception was Nunatsiavut, the Inuit subregion, where the major cause was suicide. Overall, males accounted for 70% of all injury deaths, with a mean age of 47 years (SD 22.3). By contrast, females had an average age of 63 years (SD 27.3). There do not appear to be any seasonal trends. *Conclusion.* In this study, injury mortality varied by intent, region and demographic factors, though it appeared to disproportionately impact Labrador and specific subpopulations. Unintentional injuries and suicide were leading causes of injury fatalities that warrant targeted public health interventions.

## Injury rates: successes in closing the gap closed between Aboriginal and total population in British Columbia, Canada

### M. Anne George^1^, Mariana Brussoni^1^, Andrew Jin^2^, Chris Lalonde^3^ and Rod McCormick^4^
^1^University of British Columbia, Vancouver, Canada, ageorge@mail.ubc.ca; ^2^Epidemiology Consultant, Surrey, British Columbia, Canada; ^3^University of Victoria, British Columbia, Canada; ^4^Thompson Rivers University, Kamloops, Canada

#### 

*Background.* Gaps in injury rates exist between indigenous and non-indigenous people in many regions. We provide an overview of injury trends over 25 years for the Aboriginal and total populations for British Columbia (BC), Canada. The overall declining rates mask continuing high risk for some injury types and categories, and between communities. *Methods.* Hospitalization and primary care visits, and Workers Compensation records from 1986 to 2010 were obtained from the provincial database with universal healthcare coverage. Crude rates and standardized relative risks (SRR) health care due to injury among Aboriginal people, relative to the total population of BC, were calculated. Rates were standardized by age, gender and region. *Results*: Considerable improvement was found in risk of injury hospitalization, primary care visits and Workers Compensation claims for both the Aboriginal and total BC population. Disparity in overall hospitalization rates no longer exists by 2010. For some injury types, such as unintentional falls, declining rates resulted in a narrowing disparity gap. Within the Aboriginal population, women and older adults have benefited most. For children and youth under age 25 years, rates also declined but disparity continues because rates declined at proportionally similar rates for the two populations. During times of high employment years, risk of work injury increased among Aboriginal people, exceeding the general population; but declined for Aboriginal people to below total population during years of low employment. Between Aboriginal communities, we found profound diversity rates across the province. Our ecological community-based analyses found factors independently predicting increased injury risk as well as protective factors. *Interpretation.* In spite of success in declining overall injury risk, rates remain unacceptably high. Prevention initiatives should focus on specific injury types and categories, and on ecological factors.

**References**

1. George MA, Jin A, Brussoni M, Lalonde C. Injury inequalities: is the gap closing between the Aboriginal and general population of British Columbia? Health Reports. 2015;26:3–13.

2. Brussoni M, Jin A, George MA, Lalonde CE. Aboriginal community-level predictors of injury-related hospitalization in British Columbia, Canada. Prev Sci. 2014;16:560–7. doi: http://dx.doi.org/10.1007/s11121-014-0503-1

3. George MA, Hardy C. Addressing FASD in British Columbia, Canada: analysis of funding proposals. J Popul Ther Clin Pharmacol. 2014;21:e338–45.

## Young people and risk communication related to snowmobiling in northern Norway: a focus group study

### Grete Mehus, Sidsel Germeten and Henriksen Nils
University of Tromsø, Tromsø, Norway, grete.mehus@uit.no

#### 

This study aims to understand the communication of the risks of snowmobiling among northern Norwegian youths. A qualitative design with focus-group interviews was chosen. Interviews centred on safety precautions and estimation of risks related to snowmobiling and driving patterns. Eighty-one students (31 girls and 50 boys) aged between 16 and 23 years from eight high schools were interviewed in 17 focus-groups segregated by gender. Interview data were analysed using qualitative content analysis. Boys and girls communicated differently about risks. Peer-group conformity appeared stronger among boys than girls. Boys focused upon training, coping and balance between control and lack of control while driving. Girls talked about risks, were aware of risks and sought to avoid risky situations, in contrast to boys. Youths are familiar with accidents and knows how to prevent them. Boys’ risk communication in groups was about how to manage challenging situations and how to maintain control while simultaneously testing the limits. Three risk categories emerged: those who drive as they ought to (mostly girls), those who occasionally take some risks (boys and girls) and some boys who are extreme risk-takers. Perceptions of and communication about risk are related to gender, peer-group and familiarity with risk-taking when snowmobiling. Northern Norwegian boys’ driving behaviour highlights a specific need for risk reduction. According to international research there are some important safety recomandations for snowmobilers: 1) use safety equipment, helmet and well-insulated clothes, 2)do not drink and drive, 3) follow the trails and the rules-it is for your own safety, 4) avoid night driving, mountain climbing and showing off, 5) be aware of young boys – they are in the risk zone for accidents, 6) unfamiliar areas demand extra attention, 7) let someone know where you are going and when you will return, short trips can be long – be prepared, 8) small children are not allowed to drive.

**References**

1. Sy Mary, Corden TE. The perils of snowmobiling. WMJ. 2005;104:32–4.

2. Grete M, Sidsel G, Henriksen N. Youth, snowmobiling and the “snowmobile feeling. Tidsskrift for ungdomsforskning. 2010;10:39–56.

3. Douglas M. Risk acceptabillity according to the social sciences. New York: Russel Sage Foundation; 1985.

## Environmental Health

### Global association of cold spells and adverse health effects:a systematic review and meta-analysis

#### Niilo Ryti^1^, Yuming Guo^2^ and Jouni Jaakkola^1^
^1^University of Oulu, Oulu, Finland, niilo.ryti@oulu.fi; ^2^University of Queensland, St Lucia, Australia

##### 

*Background.* There is substantial evidence that mortality increases in low temperatures. Less is known about the role of prolonged cold periods denoted as cold spells. *Objective.* We conducted the first systematic review and meta-analysis to summarize the evidence on the adverse health effects of cold spells in varying climates. *Data sources and extractions*. Four databases [Ovid Medline, PubMed, Scopus, Web of Science] were searched for all years and languages available. Cold spell was defined as an event below a temperature threshold lasting for a minimum duration of 2 days. Of 1,527 identified articles, 26 satisfied our eligibility criteria. The articles were grouped by the three main study questions into 1) overall-effect group, 2) added-effect group and 3) temperature-change-effect group. *Data synthesis.* Based on random-effects models in the meta-analyses, cold spells were associated with increased mortality from all non-accidental causes (summary-effect estimate 1.101, 95% CI 1.035–1.172), cardiovascular diseases (1.111, 95% CI 1.033–1.194), and respiratory diseases (1.213, 95% CI 0.973–1.513). The increase in mortality was larger for males (1.08, 95% CI 1.002–1.165) than for females (1.067, 95% CI 0.992–1.147), and larger for people aged ≥65 (1.059, 95% CI 1.004–1.116) than for people aged 0–64 (1.013, 95% CI 0.998–1.029). Study-specific effect estimates from a limited number of studies suggested an increased morbidity related to cold spells, but it was not possible to quantitatively summarize the evidence. *Conclusions*. Cold spells are associated with increased mortality rates in populations around the world. People with cardiovascular and respiratory diseases, elderly and men are more susceptible to these effects.

## Diabetes and impaired glucose metabolism is associated with more cold-related cardiorespiratory symptoms: the National FINRISK Study

### Tiina Maria Ikäheimo^1,2^, Jari Jokelainen^3,4,5^, Juhani Hassi^1^, Liisa Hiltunen^6^, Sirkka Keinänen-Kiukaanniemi^4,5^, Tiina Laatikainen^7,8,9^, Pekka Jousilahti^7^, Markku Peltonen^7^, Leena Moilanen^10^, Juha Saltevo^11^ and Simo Näyhä^1^
^1^Center for Environmental and Respiratory Health Research, University of Oulu, Oulu, Finland, tiina.ikaheimo@oulu.fi; ^2^Medical Research Center, University of Oulu and University Hospital of Oulu, Oulu, Finland; ^3^Medical Faculty, University of Oulu, Oulu, Finland; ^4^Unit of General Practice, Oulu University Hospital, Oulu, Finland; ^5^Center for Life Course Epidemiology and Systems Research, University of Oulu, Oulu, Finland; ^6^Health Centre of Oulu, Oulu, Finland; ^7^National Institute for Health and Welfare, Helsinki, Finland; ^8^Institute of Public Health and Clinical Nutrition, University of Eastern Finland, Kuopio, Finland; ^9^North Karelia Central Hospital, Joensuu, Finland; ^10^Department of Medicine, Kuopio University Hospital, Kuopio, Finland; ^11^Department of Medicine, Central Finland Central Hospital, Jyväskylä, Finland

#### 

*Objective.* Diabetes and impaired glucose metabolism cause metabolic, neural and circulatory disturbances that may predispose to adverse cooling and related symptoms during the cold season. This study assessed the prevalence of cold-related cardiorespiratory symptoms in the general population according to glycaemic status. *Research Design and Methods.* The study population consisted of 2,436 men and 2,708 women aged 45–74 years who participated in the National FINRISK cold sub-studies in 2002 and 2007. A questionnaire assessed cold-related symptoms (respiratory, cardiac, peripheral circulation). Glycaemic status based on fasting blood glucose, oral glucose tolerance tests or reported diagnosis of diabetes mellitus was categorized into normal glucose metabolism, impaired fasting blood glucose, impaired glucose tolerance, screening-detected type 2 diabetes and type 2 diabetes. *Results:* Type 2 diabetes was associated with increased odds for cold-related dyspnoea [Adjusted OR 1.72 (95% CI, 1.28–2.30)], chest pain [2.10 (1.32–3.34)] and respiratory symptoms [1.85 (1.44–2.38)] compared with normal glucose metabolism. Screened type 2 diabetes showed increased odds ratios for cold-related dyspnoea [1.36 (1.04–1.77)], cough [1.41 (1.06–1.87)] and cardiac symptoms [1.51 (1.04–2.20)] compared with normal glucose metabolism. Worsening of glycaemic status was associated with increased reporting of cold-related dyspnoea (impaired fasting glucose vs. type 2 diabetes from 1.16 to 1.72, p=0.000), cough (1.02 to 1.27, p=0.032), chest pain (1.28 to 2.10, p=0.006), arrhythmias (0.87 to 1.74, p=0.020), cardiac (1.11 to 1.99, p=0.000), respiratory (1.14 to 1.84, p=0.000) and all symptoms (1.05 to 1.66, p=0.003). *Conclusions:* Subjects with diabetes and pre-diabetic disturbances experience more cold-related cardiorespiratory symptoms than those with no such conditions.

## Cold health impacts in northern Sweden

### Bodil Björ^1^, Lage Burström^1^, Ingrid Liljelind^1^, Ronnie Lundström^1^, Tohr Nilsson^2^ and Jens Wahlström^3^
^1^Occupational Medicine, Umeå University Hospital, Umeå, Sweden, bodil.bjor@envmed.umu.se; ^2^Occupational- and Environmental Medicine, Sundsvall Hospital, Sundsvall, Sweden; ^3^Department of Public Health and Clinical Medicine, Umea University, Umea, Sweden

#### 

Environmental conditions in the north are characterized by large variations in temperature with long, cold and dark winters; and short, mild and bright summers. The population is exposed to cold temperature in every day life during several months a year, an exposure that has been shown to lead to various negative effects on human performance and health. Exposure to cold can be a stressor, modifier or a trigger for certain diseases and can worsen the symptoms of prevailing chronic diseases. In order to determine the prevalence of cold related symptoms in northern Sweden, a health screening questionnaire study is about to be conducted. The area from which the respondents randomly will be drawn consists of the four most northern counties in Sweden, divided into 44 municipalities. Total population in the area is about 880,000 residents. Approximately 35,000 questionnaires will be sent to residents between the age of 18 and 70 at the beginning of February 2015. Answers from this questionnaire will create a baseline cohort on cold-related health outcomes in the northern part of Sweden. The results will also be used for forthcoming case-control studies. The main purpose of the questionnaire study is to identify cases with self-reported sensitivity to cold and to investigate exposure to cold and health outcomes such as cardiovascular diseases, respiratory problems, musculoskeletal disorders, arthritis and diabetes. Specific questions that will be addressed are to what extent people in the working population in the north consider themselves sensitive to cold, to retrieve a prevalence number on self-reported, cold-related health outcomes, to investigate if there is a gender difference in previous mentioned outcomes and to look for area differences, e.g. coastline versus inland area.At the conference, an overview of the results will be presented.

## Re-thinking access to health services in the era of rapid climatic and environmental change: perspectives from Arctic Russia and interior Alaska

### Philippe Amstislavski^1^, Leonid Zubov^2^, Anton Karpunov^3^ and Alexandr London^4^
^1^University of Alaska – Anchorage, AK, USA, pamstislavski@alaska.edu; ^2^Northern State Medical University, Russia; ^3^Nenets Regional Hospital, NAO, Russia; ^4^Council of Athabascan Tribal Governments, Alaska AK, USA

#### 

*Background.* Across the Arctic, climate change and variability, mounting external pressures of extraction industries, and related socio-economic changes have been negatively impacting access to health care and to traditional subsistence. These changes impact nutrition, mobility and lifestyles in many indigenous communities. In our previous study, we demonstrated that nomadic Nenets reindeer herders in Nenets Autonomous Okrug (NAO) became more medically isolated due later freeze-up and earlier break-up of water. Their surface access to health care in the village clinic decreased by 8 weeks. In Yukon Flats region of Alaska, surface access window to the health clinic, located in the largest village, also has decreased significantly. *Objectives.* We examined several models of health services provision in Arctic Russia and Alaska, with a goal to provide compare their responses to rapid climatic and socio-economic change. *Methods.* Longitudinal surface water and temperature data for both Arctic regions was compiled and analysed in respect to geographic access. We examined how different health services models respond to decreasing access to the hub clinics: Kaninskiy Krasny Chum, which moved medical visits to Nenets reindeer herder camps (360 nomadic reindeer-breeders were examined annually), the Medical Assistant Program (both in NAO), and the Community Health Aide Program in Yukon Flats Region of Alaska. *Conclusions.* In both regions surface access to “hub” clinics has decreased significantly and the indigenous communities became more medically isolated. Centralized, top-down approaches to public health services provision fail to respond to the rapidly changing social-ecological systems in the Arctic, its health needs and its many cultures. There is an urgent need for a broad, community-centered paradigm shift to local, more flexible health care models, tethered to the specialized services via telemed, and responsive to rapid climatic and social changes.

**References**

1. Amstislavski P, Ceccato P, Weedon J, Chen H, Zubov L. Surface water change and access to health care services by in the Russian Arctic. Int J Circumpolar Health. 2013;72.

2. Walker DA, Forbes BC, Leibman MO, Epstein HE, Bhatt US, Comiso JC, et al. Cumulative effects of rapid land-cover and land-use changes on the Yamal Peninsula, Russia. In: Gutman G, Reissel A, editors. Eurasian Arctic land cover and land use in a changing climate. New York: Springer; 2011. pp. 206–236.

3. Jean-François Pekel, Cottam A, Clerici M, Belward A, Dubois G, Bartholome E, et al. A global scale 30 m water surface detection optimized and validated for Landsat 8. Paper presented at the American Geophysical Union (AGU); December 17, 2014.

## Ethnic difference in the prevalence of angina pectoris in Sami and non-Sami populations: the SAMINOR study

### Bent-Martin Eliassen^1^, Sidsel Graff-Iversen^2^, Marita Melhus^1^, Maja-Lisa Løchen^3^ and Ann Ragnhild Broderstad^1^
^1^Centre for Sami Health Research, University of Tromso – The Arctic University of Norway, Tromsø, Norway, bent-martin.eliassen@uit.no; ^2^Norwegian Institute of Public Health, and Department of Community Medicine, University of Tromso - The Arctic University of Norway, Tromsø, Norway, ^3^Department of Community Medicine, University of Tromso – The Arctic University of Norway, Tromsø, Norway

#### 

*Objective.* To assess the population burden of angina pectoris symptoms (APS), self-reported angina and a combination of these, and explore potential ethnic disparity in their patterns. If differences in APS were found between Sami and non-Sami populations, we aimed at evaluating the role of established cardiovascular risk factors as mediating factors. *Design.* Cross-sectional population-based study. *Methods.* A health survey was conducted in 2003–2004 in areas with Sami and non-Sami populations (SAMINOR). The response rate was 60.9%. The total number for the subsequent analysis was 15,206 men and women aged 36–79 years (born 1925–1968). Information concerning lifestyle was collected by two self-administrated questionnaires, and clinical examinations provided data on waist circumference, blood pressure and lipid levels. *Results*. This study revealed an excess of APS, self-reported angina and a combination of these in Sami relative to non-Sami women and men. After controlling for age, the odds ratio (OR) for APS was 1.42 (p<0.001) in Sami women and 1.62 (p<0.001) for men. When including relevant biomarkers and conventional risk factors, little change was observed. When also controlling for moderate alcohol consumption and leisure-time physical activity, the OR in women was reduced to 1.24 (p=0.06). Little change was observed in men. *Conclusion.* This study revealed an excess of APS, self-reported angina and a combination of these in Sami women and men relative to non-Sami women and men. Established risk factors explained little or none of the ethnic variation in APS. In women, however, less moderate alcohol consumption and leisure-time physical activity in Sami may explain the entire ethnic difference.

**Reference**

1. Eliassen B, Graff-Iversen S, Melhus M, Løchen M, Broderstad A. Ethnic difference in the prevalence of angina pectoris in Sami and non-Sami populations: the SAMINOR study. Int J Circumpolar Health. 2014;73:21310, doi: http://dx.doi.org/10.3402/ijch.v73.21310

## Habitual wintertime cooling and blood pressure in hypertensive and normotensive men: an experimental study

### Heidi Hintsala^1^, Jouni JK Jaakkola^1^, Juhani Hassi^1^, Riitta Antikainen^2^ and Tiina Ikäheimo^1^
^1^Center for Environmental and Respiratory Health Research and MRC Oulu, University of Oulu, Oulu, Finland; ^2^Institute of Health Sciences, University of Oulu, Oulu, Finland

#### 

Short-term exposure to cold robustly increases blood pressure (BP) and cardiovascular strain (1,2), which could relate to the reported higher wintertime cardiovascular morbidity and mortality. The aim of our study was to examine brachial BP, heart rate (HR) and HR variability (HRV) among persons whose systolic BP level or response is high in cold. We conducted a population-based recruitment of 83 men (55–65 years) which included home BP measurement to distinguish hypertensive (n=51) and normotensive subjects. Electrocardiogram and brachial BP were recorded in control conditions (18°C, 15 min) and whole-body cold exposure restricted mainly to face (−10°C, winter clothes, 15 min). HRV was computed on low frequency (LF; 0.04–0.15 Hz) and high frequency (HF; 0.15–0.4 Hz) band. We compared the results between 90th percentile of systolic BP level and response to others. Cooling increased systolic BP above 200 mmHg in 13% of subjects (1 normotensive). Systolic BP increased more than 60 mmHg in 10% of both hypertensive and normotensive men. Home measured systolic BP (146±14 mmHg, vs. 133±14 mmHg, p<0.01) and control HF HRV [93 ms^2^ (47,222) vs. 54 ms^2^ (27,120), p<0.05] were higher in men whose BP increased above 200 mmHg compared to the others. Also, whose BP increased more than 60 mmHg had higher HF HRV [119 ms^2^ (69,204) vs. 54 ms^2^ (29,121), p<0.05] and lower HR [63 bpm (58, 69) vs. 79 bpm (70, 87), p<0.01] in control conditions than the others. Cold exposure corresponding to habitual winter circumstances, and restricted mainly to the face, increases systolic BP above 200 mmHg and more than 60 mmHg in middle aged men. Those with high BP in cold were more likely to have higher BP in home, cardiac vagal activity in control conditions, and increased cold-related BP response. Interestingly, an aggravated cold-induced increase in BP occurred in both hypertensive and normotensive men. Health care specialists should be aware of the remarkable rise in BP related to everyday winter conditions.

**References**

1. Hintsala H, Kandelberg A, Herzig KH, Rintamäki H, Mäntysaari M, Rantala A, et al. Central aortic blood pressure of hypertensive men during short-term cold exposure. Am J Hypertension. 2014;27:656–64.

2. Hintsala H, Kenttä TV, Tulppo M, Kiviniemi A, Huikuri HV, Mäntysaari M, et al. Cardiac repolarization and autonomic regulation during short-term cold exposure in hypertensive men: an experimental study. PLoS One. 2014;9:e99973. doi: http://dx.doi.org/10.1371/journal.pone.0099973

## Characterization of environmental health properties of a fabricated polymericfungi-based insulation material

### Philippe Amstislavski and Zhaohui (Joey) Yang
University of Alaska – Anchorage, AK, USA, pamstislavski@alaska.edu

#### 

*Significance.* Plastic polymers are commonly used for thermal insulation in infrastructure and housing in Circumpolar North. These materials may impact indoor air quality in buildings and are non-renewable. Their production and use involve substantial energy inputs and associated toxic wastes, presenting a well-documented global environmental problem. We found that when introduced into a nutrient-rich matrix, the mycelium of certain cold-resilient fungi bounds the matrix and produces a foam-like, lightweight, insulating composite. This renewable material has no chemical binders and may have excellent, but untested, applications as environmentally sound thermal insulation that could substantially reduce environmental health impacts of construction on permafrost. Using bench-top testing data, we will report on several environmental health aspects of using the new material. *Objectives.* Recognizing the growing demand for renewable, non-toxic thermal insulation materials, this study focuses on testing the key properties for fungi insulation material. Our objectives are to 1) test laboratory protocols for fungi inoculum and produce wood pulp matrices that afford rapid and sustained growth of the biologically active fungi inoculum; 2) produce composites using of cold-resilient fungi cultures; 3) test basic properties related to potential health implications of using the resultant fungi composites. *Methodology.* This study focuses on data from bench-top experiments. Key tasks include combining wood pulp, nutrient source, binding agent and the inoculum of white-rot fungi; and incubating it under controlled conditions; measuring thermal conductivity, fire rating, and VOCs of the resultant material. Full report documenting the findings will be provided. *Potential Impact*. This study will provide initial data on health aspects of using fungi-based insulation, needed for future development of environmentally-sound thermal insulations for Circumpolar regions.

**References**

1. Novotny C, Cajthaml T, Svobodova K, Susla M, Sasek V. Irpex lacteus, a white-rot fungus with biotechnological potential – review, Folia Microbiol. 2009;54(5):375–90.

2. Arnot JA, Armitage JM, McCarty LS, Wania F, Cousins IT, Toose-Reid L. Toward a consistent evaluative framework for POP risk characterization. Environ Sci Technol. 2011;45(1): 97–103.

3. Eben Bayer GM. Method for producing rapidly renewable chitinous material using fungal fruiting bodies and product made thereby, in The United States Patent and Trademark Office. In: T. U. S. P. a. T. Office, editor. U.S. Government; 2011.

## Women’s Health and Wellbeing

### Facilitators and barriers to positive birth experiences for indigenous women in Northwestern Ontario

#### Helle Møller^1^, Pamela Wakewich^2^, Martha Dowsley^3^, Kristin Burnett^4^ and Lisa Bishop^1^
^1^Department of Health Sciences, Lakehead University, Thunder Bay, Canada, hmoeller@lakeheadu.ca; ^2^Departments of Sociology and Women’s Studies, Lakehead University, Thunder Bay, Canada; ^3^Department of Geography and Anthropology, Lakehead University, Thunder Bay, Canada; ^4^Department of indigenous learning, Lakehead University, Thunder Bay, Canada

##### 

Quality of birth experiences and outcomes has historically been unfavourable for women from remote, rural and northern communities particularly indigenous women (IW) (1, 2). Contributing factors include reduced options for birthing care and medicalized framings of risk which largely ignore social determinants and cultural legacies (3). Project Objectives were to explore the quality of women’s birth experiences to better understand the benefits and drawbacks of physician and midwifery birthing models and facilitators and barriers to a positive birth experience for women from diverse cultural and ethnic and backgrounds. Using quota sampling, we conducted a qualitative study of birthing experiences of IW, ethnic minority women and Euro-Canadian women. Interviews explored birth stories, caregiver options and preferences, and the pre-, peri- and postnatal factors that facilitated or limited positive birth experiences. Here we report the experiences of indigenous women. Positive birth experiences were aided by: continuity of care, having personal knowledge/receiving information about what to expect; having the power to decide who was present during the birth and choose pain medication if desired. Barriers to a positive birth included: feeling pushed to not have pain medication/to breastfeed; experiences of prejudice/racism; not feeling respected/informed; lack of continuity of care; and not being able to choose who was present during birth. We conclude that birthing experiences of IW in this study are framed by: the type of knowledge they have and receive; whether their preferred birth assistant, care and continuity of care are available to them; and whether they feel respected as women, decision makers and recipients of care. To secure the best birthing care and outcomes for IW, midwives and physicians must engage in education/dialogue about anti-racism and IW’s birthing experiences and preferences.

**References**

1. Kornelsen Jude, Kotaska Andrew, Waterfall Pauline, Willie Louisa, Wilson Dawn. The geography of belonging: the experience of birthing at home for First Nations women. Health & Place. 2010;16:638–645.

2. Møller Helle, Dowsley Martha, Wakewich Pamela, Bishop Lisa, Burnett Kristin, Churchill Mackenzie. A qualitative assessment of factors in the uptake of midwifery of diverse populations in Thunder Bay, Ontario. Canadian Journal of Midwifery, Research and Practice (Submitted).

3. Hall, Canadian care providers’ and pregnant women’s approaches to managing birth: minimizing risk while maximizing integrity. Qual Health Res. 2012;22:575–86.

## Considering determinants of infant feeding practices: a community-based action approach

### Pertice Moffitt^1^ and Raissa Dickinson^2^
^1^Aurora College, Forth Smith, Yellowknife, Canada, pmoffitt@auroracollege.nt.ca; ^2^UBC, Vancouver, Canada

#### 

According to the results of ABP, normal and high normal blood pressure (World Health Organization, 2010) are found in 43.7% athletes, and high blood pressure (>140/90 mm Hg) is registered in 56.3%.

## Breast and cervical cancer screening: reaching women with disabilities in Alaska

### Virginia Miller and Karen Ward
University of Alaska, Anchorage, AK, USA, jenny.miller@uaa.alaska.edu

#### 

Health disparities in cancer morbidity and mortality are important public health problems. The National Cancer Institute reports that lower rates of cancer screening contribute to more advanced disease at diagnosis and higher cancer death rates. Approximately one in five women in the U.S. experience disabilities and access to cancer screening services may be very limited, especially among low-income women. In Alaska, the influence of climate, geography and public transportation intensify problems with access services. The purpose of this study was to learn if low-income women with disabilities living in the community have reduced access to and participation in breast and cervical cancer screening. The goal was to identify and understand barriers in order to improve access to services. Using a community-based, mixed methods approach, a structural barriers framework guided the retrospective cohort study. Specific aim: explore how socioeconomic status, ethnicity, living situation, location of health care providers, insurance status, disability type and severity affect access to services. Criterion-based, purposive sampling was used to conduct face-to-face interviews with an investigator-initiated instrument and a standardized Quality of Life tool. Recruitment strategies included enlisting community partners to distribute materials, implementing a Healthy Women Alaska curriculum, and providing incentives. Despite active recruitment, enrollment was inadequate to meet the target. A sample of 41 women was recruited with formal and informal activities: grocery/discount stores/malls (9); Healthy Women Alaska (10); study groups, clinics, snowball (10); community partners (12). Low-income women with disabilities, living in the community may be hidden from traditional recruitment activities. No single recruitment method will be successful for hard-to-reach women. Creative, flexible strategies are needed to recruit participants from this important group of women.

## Inuit women’s stories of strength: driving community-based HIV and STI prevention research forward in Nunavut

### Jenny Rand^1^ and Sherry Kadlun^2^
^1^Dalhousie University, Halifax, Canada, jrrand@dal.ca; ^2^Kugluktuk, Nunavut, Canada

#### 

*Background*. In Canada, Aboriginal People (First Nations, Métis, and Inuit) are over-represented in the HIV epidemic. Although Aboriginal people make up 3.8% of the Canadian population, they represented 8% of all prevalent HIV infections in 2008 (1). For Inuit, HIV prevention and education must reflect their unique cultural, geographic and linguistic characteristics (2). This presentation will discuss findings from a community-based participatory research (CBPR) project that took place in western Nunavut. Key national organizations such as the Canadian Aboriginal AIDS Network and Pauktuutit Inuit Women of Canada have contributed greatly within the field of Inuit HIV, however, there is still a dearth of literature to guide the development of Inuit community-based HIV and STI interventions. *Methods*. This study employed indigenous methodologies, by drawing from Inuit Qaujimajatuqangit (3) in a framework of Two-Eyed Seeing, and utilizing storytelling sessions to gather data. CBPR principles informed the design of the study, ensuring participants were involved in all stages of the project. Nine story-sharing sessions took place with 21 Inuit women ages 18–60. *Findings*. Five major themes emerged through participatory data analysis: the way it used to be, change, family, intimate relationships, and holistic strategies. These findings revealed key determinants of sexual health and ideas for innovative approaches that participants believed will work as prevention efforts within their community. *Implications*. Not only are the findings from this study useful for the development of future prevention programming, the research process has generated a group of community-researchers ready to take part in more research projects. Several participants have remained engaged with the principal researcher and are currently involved in another project. These women gained valuable skills and knowledge about CBPR processes and have committed to driving research forward in their community.

**References**

1. Centre for Communicable Diseases and Infection Control. HIV/AIDS among Aboriginal people in Canada. HIV/AIDS Epi Updates (Chapter 8). Ottawa; 2010.

2. Pauktuutit Inuit Women of Canada. Inuit – five year strategic plan on sexual health. Ottawa; 2010.

3. Tester, Frank James, Peter Irniq. Inuit Qaujimajatuqangit: social history, politics and the practice of resistance. Arctic. 2008;61(1):48–61.

## Gettin’ FOXY.: exploring the development of sexual self-efficacy among young women in northern Canada using an arts-based intervention

### Candice Lys
University of Toronto, Toronto, Canada, candice.lys@gmail.com

#### 

The sexual health of Northwest Territories (NWT) youth is a serious public health concern; thus, a social arts-based intervention that uses body mapping and drama techniques, named FOXY (Fostering Open eXpression among Youth) was developed for young women in the NWT. This doctoral research is grounded in social cognitive theory and social ecological theory and uses a community-based participatory research approach, developmental evaluation methodology, and the grounded theory method to develop a theory of how FOXY influences sexual behavior expectations among young women in the NWT, considering determinants that contextualize sexual health outcomes. The first aim of this study explores the intrapersonal and interpersonal contexts that influence the efficacy expectations and outcome expectations of female youth in the NWT. The second aim determines if and how a social arts-based intervention influences individual efficacy expectations regarding sexual behaviors among female youth in the NWT. Finally, the third aim determines if and how a social arts-based intervention influences individual outcome expectations regarding sexual behaviours among female youth in the NWT. In Phase I, pilot testing occurred with 6 female youth to improve design of the semi-structured interview guide. Phase II entailed semi-structured interviews with 41 female youth aged 13–18 years selected via purposive sampling (those who have completed the FOXY workshop within the previous three days). Data collection occurred until saturation of new themes was reached at six study locations across the NWT. Interview recordings are being transcribed verbatim and a multi-stage thematic analysis using memoing and coding using the grounded theory method will occur. Results are currently in process and will be presented at this conference. Front-line workers and researchers can use the results of this dissertation to inform arts-based intervention programs and research among other Arctic populations.

## Girls’ well-being in northern Finland: promoting and hindering factors

### Varpu Wiens, Helvi Kyngäs and Tarja Pölkki
University of Oulu, Oulu, Finland, varpu.wiens@gmail.com

#### 

*Background.* Results from previous studies indicate that gender and living conditions have an impact on health and well-being. Girls present different and to some extent more symptoms than boys, and seem to perceive their health as poorer than boys. Some aspects of the living conditions in northern Finland are demanding; for example, other studies have found that seasonal affective disorders (SAD) are more common in females than males. *Objectives.* The aim of this study was to describe the factors that promote or hinder girls’ well-being in northern Finland. This study is part of a research project entitled Special Issues of Child and Family Well-being of the University of Oulu, Institute of Health Sciences. *Ethical issues*. Approvals were granted by the Northern Ostrobothnia Hospital District Ethics Committee and written consent was obtained from the girls themselves and their parents. During the research, attention was paid to human dignity, which includes respondents’ consent, voluntariness and anonymity, as well as confidence in the ability to understand the study questions. *Design.* A qualitative descriptive study approach was chosen to obtain descriptions of the girls’ perceived factors promoting and hindering their well-being. The 117 girls who participated in this study were living in northern Finland and were aged between 13 and 16. Data were collected online with open-end questions which the girls answered during the school day by computer. Inductive content analysis was employed. *Results.* Factors hindering girls’ well-being were related to the experience of illness, negative social relationships and the girls’ own negative feelings and sensations about life and its circumstances. Well-being was promoted by social relationships that were perceived as supportive and by overall positive feelings towards life. More detailed results of the study will be reported at the conference.

**References**

1. Sourander A, Koskelainen M, Helenius H. Mood, latitude and seasonality among adolescents. J Am Acad Child Adolesc Psychiatry. 1999;38:1271–6.

2. Saarijärvi S, Lauerma H, Helenius H, Saarilehto S. Seasonal affective disorders among rural Finns and Lapps. Acta Psychiatr Scand. 1999;99:95–101. doi: http://dx.doi.org/10.1111/j.1600-0447.1999.tb07206.x

3. Cavallo F, et al. Girls growing through adolescence have a higher risk of poor health. Qual Life Res. 2006;15:1577–85.

## Community Driven Research

### Partnerships as an important tool for improving community health

#### André Corriveau, Debbie DeLancey and Sabrina Broadhead
GNWT Department of Health & Social Services, Canada, andre_corriveau@gov.nt.ca

##### 

The Department of Health and Social Services, Government of the Northwest Territories (GNWT-HSS), proposes to sponsor and facilitate a one-hour panel that will offer concrete examples of the critical role that partnerships can play in the quest for improving the health of populations. People are impacted by determinants of health in the communities where they live. For those who strive to understand and meet the fundamental need for healthier communities, it is important to involve these same communities in health promotion, both in the definition of the problem(s) to be addressed, as well as in the identification and implementation of solutions. This process leads us to move from individual actions to the power of shared ideas, resources and experiences that work to create those healthier communities. As the health of a community is often impacted by societal forces over which community members have little control, complex societal problems are best overcome through collaborative solutions that bring communities and institutions together as equal partners and build upon the assets, strengths and capacities of each member. In June 2013, the GNWT-HSS created its new Aboriginal Health and Community Wellness Division with an explicit mandate to address health disparities between the aboriginal and non-aboriginal populations of the Northwest Territories. An important focus of the new division has been the fostering and strengthening of intersectoral partnerships to address important determinants of health. Four examples of such partnerships will be described through the proposed panel. These examples will cover partnerships with aboriginal governments, with academia, across government departments and directly with communities or NGOs. Each of the four panelists would do a 10 minutes presentation on their particular initiative, which will then be followed by a question and answer period with the audience. The session could also be videotaped.

## IlikKuset-Ilingannet/Culture-Connect: promoting cultural-based youth mentorship programs to support mental health and resilience in Nunatsiavut, Labrador

### Ashlee Cunsolo Willox^1^, Inez Shiwak^1^, Michele Wood^1^; The IlikKuset-Ilingannet Team^2^, The Regulate Community Government^2^
^1^Cape Breton University, Sydney, Canada, ashlee_cunsolowillox@cbu.ca; ^2^Rigolet Inuit Community Government, Rigolet, Newfoundland & Labrador, Canada

#### 

Inuit populations are at the frontlines of climate chsange and, due to continued reliance on the land for sustenance and well-being, already-present health disparities, and difficulty accessing health-sustaining resources, are often susceptible to resulting impacts to physical and mental well-being. When combined with other stressors from changes in governance structures, economies, and social structures, as well as the intergenerational impacts of colonialism, there are further potential impacts on cultural continuity, knowledge exchange, and resilience. Understanding these needs, communities across the North are finding ways to maintain cultural values, foster livelihoods and promote resilience to change. Responding to these stressors and needs and building from previous research conducted in the region that found that youth and middle-aged adults are particularly susceptible to the mental health impacts of climate change, the Inuit Community Governments of Rigolet, Makkovik, Postville, Nunatsiavut, and Labrador designed and piloted the IlikKuset-Ilingannet!/Culture-Connect! Program. This program ran from October 2013 to March 2014 and united five youth with five adult mentors in each community to learn cultural skills – hunting, trapping, snowshoe-making, carving, art and sewing – in order to assist both youth and adults in connecting together in a positive and health-promoting environment dedicated to knowledge transmission and cultural skills development and preservation. Research conducted with 40 youth, mentors and key stakeholders in the region found that participating in the IlikKuset-Ilingannet! Program increased confidence and self-worth among the youth and mentors; created new and/or enhanced relationships between and among the youth and mentors; revitalized cultural pride among youth and mentors; supported skills training and development; and showed promise as a strategy for supporting youth and mentor resilience, mental health, and adaptive capacities.

## Together for healthier lifestyles: collaboration with multiple sectors in northern Canada linked to healthy eating, active living and health literacy

### Jody Butler Walker, Katelyn Friendship, Marilyn Van Bibber and Norma Kassi
Arctic Institute of Community-Based Research, Whitehorse, Canada, katelyn@aicbr.ca

#### 

The Arctic Institute of Community-Based Research (AICBR) is leading a four-year project *Working Together To Achieve Healthier Lifestyles in Yukon and Northwest Territories Communities*, funded by the Public Health Agency of Canada. The project targets children and youth, families, rural and remote, indigenous and non-indigenous communities in and across the two Territories. It focuses on the strengthening of partnerships and collaborations, in order to identify, plan, implement, and evaluate initiatives that focus on healthy lifestyles for northern families and communities. Our presentation will report on our approach and findings to date on efforts to enhance and strengthen collaboration and networking between and within non-government and government agencies, local businesses and communities in both YT and NWT, with a focus on fostering healthy lifestyles in communities, including healthy eating, active living and health literacy. As a part of this, we are looking to understand factors of sustainability and scalability of successful health interventions within a rural, remote and northern context; and through a community-based research lens. By improving the understanding of factors that influence the sustainability of programs and the initiative as a whole, we will be better positioned to identify how community-based partnerships can enhance facilitators and reduce barriers which may influence the success or failure of programs within a rural, remote, northern context. As lead and support for the project, AICBR is employing a collective impact approach working with those who share a common agenda, offer mutually reinforcing activities, and facilitating ongoing communication between partners. We anticipate outcomes from this project will shed new light on the importance of inter-sectoral collaboration for influencing sustainability and scalability of successful programs, and for contributing to long-term community health outcomes.

## Institutional ethnography as a decolonizing method of inquiry for applied health research

### Melody Morton Ninomiya
Memorial University, St John’S, Canada, melodym@mun.ca

#### 

I suggest that institutional ethnography (IE) can be used as a decolonizing method of inquiry. In the midst of an informal research moratorium in a rural indigenous community in eastern Canada, an exception was made for an applied health research study that had the potential to further stigmatize the community. Based on this applied and community-based health research study, I suggest that IE as a community-based research (CBR) study paired with appropriate knowledge translation (KT) can be used for decolonizing research and as a means to a decolonizing “end”. indigenous scholars have argued that by privileging the knowledge of those who are being researched (people subject to the effects of colonization) over those whose knowledge has been privileged (researchers), decolonizing health research can reduce inequalities and improve health outcomes.Widely accepted forms of mainstream qualitative and quantitative health research and knowledge translation (KT) practices have been criticized for (unknowingly) using colonialist ways of doing and knowing. Concepts and language of mainstream research and KT practices are rooted in methods and representations that are often incongruent with indigenous paradigms – paradigms were knowledge and action is intertwined and inseparable. IE is a method of inquiry born out of sociology that has been used to explicate the social organization of heath work – through, by and within health institutions. Rather than aim to test or generate theory, IE produces evidence-based research that “maps” how peoples’ activities are (invisibly) coordinated by institutional texts. To my knowledge, IE has rarely been used to do research explicitly with indigenous people. In this presentation, I suggest and illustrate how IE can be used as decolonizing method of inquiry when married with key CBR principles and appropriate indigenous KT.

## The Inuit Health Survey 2007–2008: a participatory and co-managed approach for this legacy study

### Hope Weiler^1^, Maureen Baikie^2^, Sharon Edmunds^3^ and Linnea Ingebrigtson^2^
^1^McGill University, Montréal, Canada, hope.weiler@mcgill.ca; ^2^Government of Nunavut, Iqaluit, Canada; ^3^Nunavut Tunngavik Incorporated, Iqaluit, Canada

#### 

The IHS 2007–2008 comprehensively assessed physical and mental health of Inuit and identified important modifiable factors behind health outcomes. In 2014, the Nunavut partners, McGill University, Nunavut Tunngavik Incorporated and Government of Nunavut, Department of Health co-established and signed a Research Partnership and Results Sharing Agreement to guide the use, access and dissemination of IHS data. The Nunavut partners are now implementing the Agreement and the research processes associated with it. To that end, the Nunavut Inuit Health Survey Steering Committee has developed a protocol to assess all requests related to the use of survey data, including mutually agreed upon terms for access to IHS data, including a process for review and feedback on reports relevant to Nunavut. As the Agreement provides a standardized process, including mechanisms for access, it will help students, researchers and other partners wishing to access and use data.The signing of the Agreement represents an opportunity for:

- communities to be involved in how their information is represented and reflected,
- new approaches for moving forward with partnership based research projects as related to the IHS and those of the future (of this nature and scale), and
- research to play a larger role in affecting positive change in our communities and Nunavut’s institutions of governance (i.e. with co-control over data, dissemination and messaging that is reflective of the context reflected in the data).

## Ten years of a community based participatory research program to address the high rate of Long QT syndrome in First Nations of northern BC

### Laura Arbour^1^, Julie Morrison^2^, Sarah McIntosh^1^, Beatrixe Whittome^1^, Fernando Polanco^1^ and Rosemary Rupps^1^
^1^UBC, Vancouver, Canada, larbour@uvic.ca; ^2^Gitxsan Health Society, Hazelton, Canada

#### 

In 2004, the representatives of the Gitxsan Nation in northern British Columbia (BC) requested that researches from the University of British Columbia (UBC) meet to discuss the high rate of Long QT syndrome (LQTS), an inherited disorder predisposing to sudden cardiac arrest. Discussions commenced, and a special advisory group consisting of local health care providers, community members and the Gitxsan Health Society began their work together with the researchers to address this health care priority. Research questions and methods were developed together, and the concept of “DNA on Loan” was agreed upon to ensure that genetic material was considered part of the participatory process. DNA was considered to be on loan to the researchers specifically for LQTS research, and could be returned to the participant if requested. In addition, all research steps, and changes in protocol would be discussed with the Gitxsan Health Society and as well, all manuscripts before publication would be reviewed. Participants would be updated with progress both by regular public presentations and newsletters. To date, more than 700 participants have enrolled. The causative genetic factor, the V205M mutation in KCNQ1 (known to cause LQTS) was discovered and confirmed to predispose to abnormal heart rhythm. More than 125 individuals have been found with the mutation, and clinical care has been put in place locally. Specific times in life have been determined as riskier than others, such as for women of childbearing years, but reassuring to see that children seem to have minimal effects. Further studies are underway to determine if minor genetic factors increase or decrease risk, and whether chronic disease influences outcomes. Ten years later, the on-going relationship between the community and the researchers remains strong. The merits of “continuous conversation” will be discussed and may be useful for others as they embark on community based participatory methodologies.

**References**

1. Arbour L, Cook D. DNA on loan: issues to consider when carrying out genetic research with Aboriginal families and communities. Community Genet. 2006;9:153–60. doi: http://dx.doi.org/10.1159/000092651

2. Arbour L, Rezazadeh S, Eldstrom J, Weget-Simms G, Rupps R, Dyer Z, et al. A KCNQ1 V205M missense mutation causes a high rate of long QT syndrome in a First Nations community of northern British Columbia: a community-based approach to understanding the impact. Genet Med. 2008;10(7):545–550.

## Correlation of maternal cotinine and neonatal NNAL levels:preliminary findings from the MAW study

### Christie Flanagan^1^, Kathryn Koller^1^, Timothy Thomas^1^, Abbie Willetto Wolfe^1^, Neal Benowitz^2^ and Christi Patten^3^
^1^Alaska Native Tribal Health Consortium, Anchorage, AK, USA, awolfe@anthc.org; ^2^University of California-San Francisco, San Francisco, USA; ^3^Mayo Clinic, Rochester, USA

#### 

*Background.* The prevalence of tobacco use during pregnancy is higher among Alaska Native (AN) women compared to other ethnic populations in the U.S. *Purpose*. In response to requests by tobacco-using AN women for specific information about fetal exposure to tobacco, we are developing and testing a novel intervention to motivate tobacco cessation in pregnant AN women. We present preliminary findings from Phase I, which examines biomarkers of exposure (maternal cotinine, a nicotine metabolite; and newborn NNAL, a carcinogen) in urine samples, obtained from postpartum AN women and their newborns. *Methods.* We are recruiting pregnant AN women, 18 years of age and older, receiving care at Southcentral Foundation in Anchorage, Alaska and planning to deliver at the Alaska Native Medical Center. The targeted enrollment is 165 women with 55 in each of three self-reported tobacco use categories: non-tobacco user, cigarette user and smokeless tobacco (ST) user. Urine is obtained from the mother and neonate within 24 hours of delivery. *Results.* In a preliminary analysis, 52 paired urine samples were analysed for maternal cotinine and infant NNAL levels. Maternal cotinine and infant NNAL correlation r value was 0.44 in smokers (n=36). In iqmik users (n=12) infant NNAL levels remained low regardless of maternal cotinine levels. Data for commercial ST users (n=4) is inadequate for correlational assessment. *Conclusion.* These preliminary findings demonstrate neonatal NNAL exposure positively correlates with maternal cotinine levels in AN women who smoke cigarettes during pregnancy. Infant NNAL levels appear to differ depending on product used in ST users; commercial versus iqmik. Phases II and III aim to work with AN women and their partners to develop and assess interventions using findings from Phase I.

**References**

1. Martin LT, McNamara M, Milot A, Bloch M, Hair EC, Halle T. Correlates of smoking before, during, and after pregnancy. Am J Health Behav. 2008;32(3):272–82.

2. Hecht SS. Human urinary carcinogen metabolites: biomarkers for investigating tobacco and cancer. Carcinogenesis. 2002;23:907–22.

3. Cnattingius S. The epidemiology of smoking during pregnancy: smoking prevalence, maternal characteristics and pregnancy outcomes. Nicotine Tob Res. 2004;6(Suppl 2):S125–140.

## Indigenous Community Food Security in Yukon Territory

### Norma Kassi, Jody Butler Walker, Katelyn Friendship and Marilyn Van Bibber
Arctic Institute of Community-Based Research, Whitehorse, Canada, katelyn@aicbr.ca

#### 

With changing climate and environmental conditions and increasing costs for food, food security is of increasing concern in Yukon Canada. Indeed, Yukon First Nations’ elders have been advising their communities for some time that hard times are coming and that it is time to plan for long-term changes related to food security. To that end, the Arctic Institute of Community-Based Research has been working in partnership with communities to develop locally based food security strategies. Our presentation will focus on the community-based approach we follow to engage with indigenous communities in a respectful and ethically responsible manner. Youth participation and capacity building, culturally appropriate research design and methods, elder participation, traditional knowledge, community research capacity development and community engagement are some examples of our methods. It is evident that for long term food sustainability and security, communities want clear plans that they can build from; which include being more self sufficient by increasing local food production, building community gardens, increasing animal husbandry, building micro enterprises, and returning to ancient methods of sharing and wildlife management. Following a community-based approach has resulted in tangible food security strategies tailored to meet the needs of each community circumstances and cultural heritage.

## Nature and Health

### Travel to Arctic regions – are travel health recommendations contemporary?

#### Anders Koch
Statens Serum Institut, Copenhagen, Denmark, ako@ssi.dk

##### 

Few people seek medical advice when travelling to Arctic regions. Yet, a number of vaccine preventable diseases are prevalent in Arctic areas and may pose risks to travellers. Such diseases include among others viral hepatitis type B and tuberculosis. In addition, other non-vaccine preventable infections such as sexually transmitted infections are likewise prevalent and may pose similar risks. Finally, a range of zoonotic infections may affect travelers. In spite of this, official travel health recommendations for Arctic areas are often absent or differ from each other regarding risk assessment. When existing, such travel health recommendations may include vaccination against hepatitis A, hepatitis B, tuberculosis, rabies and/or tetanus. Part of the reasons for the different or missing travel health recommendations may be due to the fact that Arctic areas for a large part are not individual countries, but rather areas or jurisdictions in countries where health risks for the most part are highly different from those of Arctic areas, e.g. the USA or Canada.

In this presentation, vaccine-preventable and non-preventable travel health risks in Arctic areas are assessed; existing travel vaccination programs are reviewed; and health considerations before and during travel to such areas are discussed.

## Impacts of winter weather conditions and slipperiness on tourists’ health in Finland

### Élise Lépy^1^ and Arja Rautio^2^
^1^Faculty of Humanities, University of Oulu, Oulu, Finland, elise.lepy@oulu.fi; ^2^Centre for Arctic Medicine, Thule Institute, University of Oulu, Oulu, Finland

#### 

Living conditions of Arctic and sub-Arctic communities are affected by the variability of climate and environmental changes, which all have impacts on health, subjective well-being and quality of life. The present study considers the impacts of the variability of winter weather conditions and slipperiness on the health and safety of people, especially tourists, practicing outdoor activities in northern Finland. A particular focus is made on Sotkamo area, one of the most visited places in Finland with a significantly growing tourism and boosted by Vuokatti resort centre. The main objective of this study is to analyse the impacts of the variability of weather conditions and therefore the quality of icy and snowy ground surface conditions on the health of tourists during the winter season in Sotkamo tourist area. We attempt to estimate the slipping hazard exposures in a specific context of space (touristic centre) and time (winter season) and we focus closely on the weather and other possible parameters (e.g. holidays, seasonal variations) which could be responsible of increased amounts of injuries and accidents. The methodology is based on statistics of weather data and patients’ health care visits and on questionnaire and interviews conducted with health care personnel. More precisely, we examine the correlation between meteorological factors and others with patients who got injuries to the shoulder and upper arm (S40–S49 according to the ICD-10 diagnosis codes), to the elbow and forearm (S50–59), to the wrist and hand (S60–69), to the hip and thigh (S70–79), to the knee and lower leg (S80–S89) and to the ankle and foot (S90–S99) from October to April of each year.

## Store Outside Your Door: hunt, fish, gather, grow

### Gary Ferguson^1^, Desiree Jackson^1^ and Tara Stiller^1^
^1^Alaska Native Tribal Health Consortium, Anchorage, AK, USA, gferguson@anthc.org

#### 

The Alaska Native Tribal Health Consortium (ANTHC) Store Outside Your Door (SOYD) Initiative focuses on the promotion of traditional and local foods by expanding on the concepts of Hunting, Fishing, Gathering, and Growing in Alaska. The program works with Alaska Native communities to highlight their local bounty in the “Store Outside.” Our rural communities are often considered “food deserts”, if just comparing what is available in the local store. The SOYD program has been working, over the past 6 years to educate and empower communities in the knowledge of how to live vibrantly off the bounty of the land around them. We highlight successful hunter, fisher, gatherers and help share elder wisdom that has helped our First People survive for thousands of years in the oftentimes harsh landscape that many of our communities are located. Through workshops, written materials, and mostly through the creation of “webisodes” (videos for web release) and social media, we are working with Alaska Native families so children can grow up with healthy, local foods. This addresses food security and its connections to chronic disease and also helps link traditional foods with reinforcing the wisdom in our many cultures, languages – thereby also promoting resilience. The SOYD program has created a well-received webisode series, highlighting the 7 main regions of Alaska with traditional food recipes and how to hunt, fish, gather, grow in that region. The SOYD initiative started with the USDA NIFA funded research project, “Helping Ourselves To Health.” Communities engaged through focus groups addressing food/nutrition security asked for more modern recipes utilizing traditional foods along with media that they could view on TV, Internet. Our current focus is on developing Maternal Child Health resources reinforcing traditional foods as first foods. We have our first webisode developed, “Ky’Woks” (Tshimshian language translated, “the food we give our infants for teething”).

**References**

1. www.youtube.com/anthcstoreoutside

2. www.facebook.com/storeoutside

3. www.alaskanplants.org

## Climate change, human health and well-being in Yakutia

### Tatiana Burtseva^1^, Victor Shadrin^1^, Sergei Avrusin^2^, Irina Solodkova^2^ and Vyacheslav Chasnyk^2^
^1^Yakut Research Centre for Complex Medical Problems SB RAMS, Yakutsk, Russian Federation; ^2^Saint-Petersburg State Pediatric Medical University, Saint-Petersburg, Russian Federation, chasnyk@gmail.com

#### 

*Introduction.* Though the pattern of average ambient temperature along the timeline of millions of years is known to have the downward trend, it is known also that it has fluctuations of incremental range (1). The global trend for the average temperature curve during the last 120–140 years is upward (2) and climate change-related exposures are likely to affect the health status of millions of people. *Objectives.* 1) to reveal climate-dependent problems in everyday activities of people living in rural areas of Yakutia, 2) to analyse the demographic and health status tendencies possibly associated with climate change. *Materials and methods.* The study was designed as a community survey conducted face-to-face, supplemented with analysis of the state reports. A total of 160 subjects living in eight rural settlements situated in the central and northern parts of Yakutia were reviewed: 15 inhabit Bulunsky, 15 Nizhnekolymsky and 130 Gorny uluses. The questionnaire consisted of 27 questions that the respondent had to answer in a set format picking an answer from a given number of three options: “yes”, “no”, don’t know. *Results.* The most impressive changes are registered for thinner ice on rivers, more muddy water and ice in rivers, greater number of sunny days, difficulties to predict good weather and beginning of spring, not so successful hunting, fishing, gathering mushrooms and berries and greater than it has been 5 years ago quantity of mosquitoes. Everyday activities of hunters, fishermen and herders have changed during the last 5 years due to climate change. People living in the central part of Yakutia are affected to a greater extent, which is associated with faster warming. *Conclusion.* The changes lead to problems with long-term and short-term weather forecast, difficulties to travel along tundra and forest, reindeer grazing, problems with fishing, hunting, gathering mushrooms and berries. Changes in morbidity can hardly be associated with climate change.

**References**

1. Lisiecki LE, Raymo ME. A Pliocene-Pleistocene stack of 57 globally distributed benthic 18O records, Paleocenography. 2005;20:18PA1003.

2. IPCC, 2007: Climate Change 2007: Synthesis Report. Contribution of Working Groups I, II and III to the Fourth Assessment Report of the Intergovernmental Panel on Climate Change, Intergovernmental Panel on Climate Change; 2007 [cited 2014 Jan 30]. Available from: http://www.ipcc.ch/graphics/syr/fig1-2.jpg

## A human-made flood: when the umbilical cord to water and life becomes inimical to health

### Myrle Ballard, Donna Martin, Shirley Thompson, Amanda Johnson, Janice Linton and Barry Lavallee
University of Manitoba, Winnipeg, Canada, myrle.ballard@umanitoba.ca

#### 

Situated on the shores of a freshwater lake once used for traditional and cultural activities, all members of Little Saskatchewan First Nation (LSFN), located 225 kilometres north of Winnipeg, experienced an emergency evacuation as a result of a human-made flood in 2011. To date, over 200 of the 700 on-reserve community members continue to reside in urban hotels or temporary housing in the City of Winnipeg. Residences in their home community have yet to be provided. Common themes emerged from displaced community members’ anecdotal stories about their health and well-being: premature deaths, suicides, miscarriages, mental health disorders, youth involvement in gangs, marital break-ups, along with worsening of chronic illnesses with no formal community health assessment. Few studies have examined, systematically documented, or digitally recorded the health outcomes of induced displacement as experienced by indigenous peoples. In this paper, we present the preliminary findings of health outcomes of induced displacement from the perspectives of several elders. With input from community leaders, indigenous and Non-indigenous researchers utilized a qualitative research method (participatory video) that honoured the “oral traditions.” This multidisciplinary research team was guided by an Advisory Council comprised of two elders from LSFN and the Band Council member for health. Data sources included surveys, individual interviews, field notes, focus groups, community-based workshops, evacuation and relocation policies and procedures, and participatory video. This study is significant in that foundational knowledge about the micro- and macro-construction of induced displacement among LSFN community members will emerge. Secondly, findings may be used to guide policy and program development and the evaluation of new service delivery models to indigenous peoples experiencing displacement. Thirdly, this study will provide a basis for future research.

## Backcountry travel emergencies in northern Canada:a case series of media-reported events

### Stephanie Young^1^, Taha Tabish^2^, Nathaniel Pollock^3^, Katie O’Beirne^1^ and Kue Young^4^
^1^Institute of Circumpolar Health Research, Yellowknife, Canada; ^2^Qaujigiartiit Health Research Centre, Iqaluit, Canada; ^3^Labrador Institute of Memorial University, Happy Valley-Goose Bay, Canada, nathaniel.pollock@med.mun.ca; ^4^University of Alberta, Edmonton, Canada

#### 

*Introduction.* Rural and indigenous populations in northern Canada regularly travel in backcountry areas where they have limited telecommunications and emergency access. This travel is necessary for employment, hunting, cultural practices, medical care and recreation, and often involves transport by snowmobile or boat. Travel in remote areas can pose risks for injuries and the media commonly reports on such incidents. In this study, we described the extent and characteristics of backcountry travel emergencies. *Methods.* We used a case series design to examine backcountry travel emergencies in the Northwest Territories (NWT) and Nunavut, Canada from 2004 to 2013. We identified cases by conducting an online search for articles from two media outlets, Northern News Services and Nunatsiaq News using the terms “rescue”, “missing” and “search.” We searched for cases related to travel that resulted in an emergency event such as a collision or missing person. We used descriptive statistics to examine demographic, environmental, and health-related trends. *Results.* Our analyses showed that backcountry travel emergencies are most frequent among males, between the ages of 20 and 39. In NWT, most travellers originated in Yellowknife and Inuvik, and events occurred most often in July and August. In Nunavut, travellers often originated in Iqaluit or Baker Lake, and events occurred mainly in November. The key factors that lead to emergencies were accidents in NWT and poor weather in Nunavut. In NWT and Nunavut, we found that 28% and 31% of events, respectively, resulted in the death of at least one group member. *Conclusion*. We showed that backcountry travel emergencies are frequent in northern Canada. They most often occurred among adult men, during the summer and fall, and were often fatal. Northern communities may benefit from improved emergency response infrastructure and public health interventions to increase access to safety gear and survival training.

## Subjective Evaluation of Health and Health-Related Quality of Life of Adolescents, Inhabitants of the Arctic Yamal

### Victor S. Rukavishnikov, Olga A. Diakovich and Marina P. Diakovich
East-Siberian Institution of Medical and Ecological Research, Irkutsk, Russia, marik914@rambler.ru

#### 

There are about 92% of natural gas and 10% of the Russian’s oil reserves in Yamal-Nenets autonomous area-one of the most northern regions of Russia. The investigation of health related quality of life (HRQoL) of the indigenous and the rooting population is important for resolving the issue of sustainable development of the region. Objective was to assess HRQoL and the health risk (HR) in adolescents living in the Yamal. Objects were adolescents from the district center at ages 14–17, 58 Nenets from a boarding school, 26 Russians from families. The assessment of the HR (from 0 to 1) was calculated with Bayes method. The PedsQL 4.0 generic core scheme self–report was used to assess HRQoL with age-appropriate components. The psychological (mood, communication, school), physical (actual health), total HRQoL components were identified. Share of adolescents with extremely high health risk (greater than 0, 95) were 31 and 46% among the Nenets and Russian. Nenets had lower risk of hypertension, functional disorders of the respiratory system, neurological disorders, borderline mental disorders. Regardless of ethnicity boys risk levels were lower, risks of digestive system and borderline mental disorders were most prevalent. HRQoL of Nenets was higher compared with Russian as a whole and on the role functioning scale that characterizes the communication. Gender differences in HRQoL were not identified. Nenets had psychological and physical components of HRQoL higher than the Russian. These results can be linked with less secrecy in the presentation of health complaints in adolescents living in families compared with the Nenets, was brought up in a boarding school. Further, subjective and objective study of health-related quality of life, social well-being and frustrations of adolescents living in the North, to search for the interdependence of these factors is necessary.Studies supported by the program fundamental researches of presidium of RAS, the project AZ RF-44P.

## Wednesday June 10th 2015

### Plenary Session

#### Västerbotten Intervention Programme – experiences and implications for population health

##### Margareta Norberg
Umeå University, Umeå, Sweden, margareta.norberg@umu.se

###### 

The Västerbotten Intervention Programme (VIP) was launched in 1985 as a response to the highest regional cardiovascular disease (CVD) mortality rates in Sweden (1). VIP was initially piloted in a small municipality, Norsjö, and then gradually introduced throughout the county, and it reached the entire middle-aged population by 1992. VIP combines population-based health promotion strategies with annual invitation to primary care for inhabitants turning 40, 50 or 60 years to attend a health assessment for systematic CVD risk factor screening and individual counselling by trained nurses to promote CVD prevention. Until 2013, 65,000 individuals have participated once and over 40,000 at least twice, generating data from over 150,000 health examinations including conventional cardiovascular risk factors and information about life style, socio-economic situation, quality of life, working conditions and psychosocial stress. The structure of VIP, the well-defined VIP population and data collection provide possibilities for research with cross-sectional studies (2), nested case-referent studies and cohort studies, and enables evaluations of prevalence, incidence, trends over time and longitudinal associations. Moreover, linkage with local, regional and national databases and registers provide opportunities for interdisciplinary research and assessment of demographic and socio-economic conditions in the population and subpopulations as well as evaluation of societal and health care interventions (3). VIP data are also used in international collaborations including several cohorts. In this presentation, some examples on results based on VIP data will be presented including effect of the VIP on total and CVD mortality in the target population in Västerbotten.

**References**

1. Norberg M, Wall S, Boman K, Weinehall L. The Vasterbotten Intervention Programme: background, design and implications. Glob Health Action. 2010;3:4643. doi: http://dx.doi.org/10.3402/gha.v3i0.4643

2. Long GH, Simmons RK, Norberg M, Wennberg P, Lindahl B, Rolandsson O, et al. Temporal shifts in cardiovascular risk factor distribution. Am J Prev Med. 2014;46(2):112–21.

3. Malmberg G, Nilsson LG, Weinehall, L. Longitudinal data for interdisciplinary ageing research. Design of the Linnaeus Database. Scand J Public Health. 2010;38(7):761–7.

## The Norwegian Mother and Child Cohort Study

### Ted Reichborn-Kjennerud
Norwegian Institute of Public Health, University of Oslo, Norway, Ted.Reichborn-Kjennerud@fhi.no

#### 

The Norwegian Mother and Child Cohort Study (MoBa) is currently the largest longitudinal birth cohort in the world, and includes more than 110,000 children, 95,000 mothers and 75,000 fathers as well as over 35,000 siblings. Data collection was done from 1999 to 2009 and was nationwide. Both mothers and fathers were recruited at ultrasound examinations around gestational week 17–18. Mothers have completed questionnaires during pregnancy and at regular intervals following birth, assessing mothers’ health, life style, dietary intake, socio-economic factors, child development and behaviour and other exposures relevant to child and parental health. The long-term goal intention is to follow the families across generations. Both parents have donated blood samples, and urine from the mother was collected to assess environmental toxins. Umbilical cord blood was drawn from the child at birth. MoBa data are routinely linked to data from the Medical Birth Registry of Norway. Follow-up of the cohort is also conducted through linkages to national health registries, including the Norwegian Patient Registry, the Norwegian Prescription Registry and the Sickness and Disability Registry. Additional data collection is conducted in case-control sub-studies focusing on specific disorders. The main aim of MoBa is to identify causes of diseases. There is good evidence that conditions during pregnancy and early childhood can have a major impact on a child’s later health, including both mental and somatic disorders. The strengths of MoBa include biological material, a prospective design which give more reliable data on both exposure and outcome, large sample size necessary to have sufficient statistical power to analyse rare disorders, paternal participation, allowing us to investigate paternal impact on child health and a large number of siblings, which permit us to control for common genetic and environmental risk factors.

## Northern Finland Birth Cohort Programme

### Marjo-Riitta Järvelin
Imperial College London, London, United Kingdom, m.jarvelin@imperial.ac.uk

#### 

The Northern Finland Birth Cohort study programme (NFBC) is the product of a project initiated in the 1960s to examine risk factors involved in preterm birth and intrauterine growth retardation, and the consequences of these early adverse outcomes on subsequent morbidity. The two cohorts of women and newborns were collected at 20-year intervals from the provinces of Oulu and Lapland: the younger cohort with an expected date of birth between 1 July 1985 and 30 June 1986, comprising 9,362 mothers and 9,479 children (NFBC1986), and the older with an expected date of birth in 1966, comprising of 12,068 mothers and 12,231 children (NFBC1966). Pregnancies were followed prospectively from the first antenatal contact (10–16th gestational week) and children at the ages of 6–12 months, 7–8 years (NFBC1985), 14–16 years, 24 years (subsample) (NFBC1966, 1986) and at the age of 31 years and 47 years (NFBC1966). At these time points, a wide range of phenotypic, lifestyle, demographic and other data were gathered using questionnaires and clinical examinations. The 16 years (in NFBC1986 for 6,700), 31 years and 47 years (NFBC1966 for up to 5,900) clinical outcome data include, for example, blood pressure, pulse, physical fitness (bicycle ergometry for NFBC1986), anthropometry, skin prick tests, lung function (spirometry), blood samples including DNA, fasting glucose, insulin, lipids, selected hormones, as well as metabonomic data (150 metabolites for NFBC1966 and 1986 by Nuclear Magnetic Resonance (NMR)) and stored serum, plasma and cells. GWAS data for 5,500 in NFBC1966, exome chip data for 1,500 in NFBC1966 and GWAS+exome chip for 4,000 in NFBC1986. Epigenetic methylation data have been generated for over 800 in NFBC1966 and for over 500 in NFBC1986. Linkage to national registries (hospitalization, deaths, education, medication, pensions) allows access to additional demographic and clinical data. Consequently, the NFBCs provide possibly the world’s largest data on early pregnancy measures with follow-up until the age of 50 years.

## Infectious Diseases III

### Rates of hospitalization with *Helicobacter pylori* and gastric cancer in American Indian and Alaska Native persons and in the U.S. population

#### Ian Plumb^1^, Marissa Person^1^, Robert Holman^1^ and Tom Hennessy^1^, Michael Bartholomew^2^, Claudia Steiner^3^ and Michael Bruce^1^ ^1^Centers for Disease Control and Prevention, Anchorage, Alaska, USA, iplumb@cdc.gov; ^2^Indian Health Service, Rockville, Maryland, USA; ^3^National Institutes of Health, Rockville Pike Bethesda, Maryland, USA

##### 

*Background*. *Helicobacter pylori* infects approximately 50% of the global population, and increases the risk for gastric cancer. Alaska Native populations have *H*. *pylori* antibody seroprevalence up to 75%, and high gastric cancer mortality. We estimated the rate of hospitalization associated with *H*. *pylori* infection and gastric cancer diagnosis in American Indians/Alaska Natives (AI/ANs), and in the general U.S. population. *Methods*. We analysed two hospital discharge data sets for *H*. *pylori* or gastric cancer diagnoses during 2006 through 2011: the Indian Health Service (IHS) Direct and Contract Care Inpatient for AI/ANs, and the Nationwide Inpatient Sample for the general U.S. population. Average annual age-specific and age-adjusted hospitalization rates were calculated, for 2006–2008 and 2009–2011, for AI/ANs and for the general U.S. population. *Results*. From 2006–2008 to 2009–2011, average annual age-adjusted hospitalization rates/100,000 declined in the IHS AI/AN population for *H*. *pylori* by 35.0% (33.4–21.7), and for gastric cancer by 32.5% (12.6–8.5); respective rates for the Alaska region declined by 29.8% (44.7–31.4) and 14.8% (29.0–24.7). In the general U.S. population, rates were estimated to decline by 22.1% for *H*. *pylori* from 18.1 (95% confidence interval [CI]17.8–18.5) to 14.1 (CI 13.8–14.4); and remain stable for gastric cancer: 14.3 (CI 13.8–14.7) compared with 14.0 (CI 13.6–14.5). In 2009–2011, for both outcomes, rates were highest in AI/AN men aged ≥65 years residing in Alaska. *Discussion*. The hospitalization rates for *H*. *pylori* and gastric cancer declined from 2006–2008 to 2009–2011 in AI/ANs, while rates for *H*. *pylori* declined and gastric cancer appeared to have plateaued in the general U.S. population. In 2009–2011, both *H*. *pylori* and gastric cancer hospitalization rates in the Alaska region were higher than in the other IHS regions and in the general U.S. population, especially among older males.

**References**

1. Demma LJ, Holman RC, Sobel J, Yorita KL, Hennessy TW, Paisano EL, et al. Epidemiology of hospitalizations associated with ulcers, gastric cancers, and *Helicobacter pylori* infection among American Indian and Alaska Native persons. Am J Trop Med Hyg. 2008;78(5):811–8.

2. Feinstein LB, Holman RC, Yorita Christensen KL, Steiner CA, Swerdlow DL. Trends in hospitalizations for peptic ulcer disease, United States, 1998–2005. Emerg Infect Dis. 2010;16(9):1410–8.

3. Leontiadis GI, Nyrén O. Epidemiology of *Helicobacter pylori* infection, peptic ulcer disease and gastric cancer. In: Talley NJ, editor. GI epidemiology: diseases and clinical methodology. 2nd ed. New York: Wiley; 2014. p. 135–57.

## Serotype and genotypes of penicillin-nonsusceptible invasive pneumococcal isolates in the pneumococcal conjugate vaccine era in Alaska

### Karen Rudolph, Michael Bruce, Lisa Bulkow, Tammy Zulz, Alisa Reasonover, Marcella Harker-Jones, Debby Hurlburt and Thomas Hennessy
Arctic Investigations Program, Centers for Disease Control and Prevention, Atlanta, GA, USA, krudolph@cdc.gov

#### 

*Background*. Introduction of pneumococcal conjugate vaccines (PCV7 – 2001; PCV13 – April 2010) in Alaska resulted in reduced rates of vaccine-type invasive pneumococcal disease (IPD). We evaluated the impact of conjugate vaccines on the serotype and genetic composition of penicillin-nonsusceptible (PNS) IPD isolates from 2001 to 2013. *Methods*. IPD isolates collected from 2001 to 2013 through statewide laboratory surveillance were confirmed and serotyped by standard methods. Antimicrobial susceptibility testing was performed by broth microdilution; isolates with an MIC of ≥0.12 µg/mL were classified as PNS. *Results*. Of 1,448 IPD isolates recovered from 2001 to 2013, 248 (17.1%) were PNS. The rate of PNS IPD did not change (2.89–2.57/100,000/yr; p=0.412) between the pre- (2001–March 2010) and post-PCV13 (April 2010–2013) periods. However, the rate of PNS PCV13-type IPD declined significantly (2.5/100,000–1.0/100,000; p<0.001) and the rate of PNS non-vaccine type IPD increased (0.41–1.58/100,000/yr; p<0.001). PCV13 serotypes represented 86% (153/178) and 39% (27/70) of total for pre- and post-vaccination periods, respectively. The most common non-vaccine serotypes (6C, 15A, 23A, 35B) made up 53% of the isolates. Forty-eight different sequence types (STs) were found; six STs (172, 199, 338, 320, 558, 63) represented the majority (63%) of isolates. Forty STs were associated with the pre-vaccination period (27 exclusive) and 21 (8 exclusive) with the post-vaccine period; 13 STs were identified in both periods. *Conclusions*. The proportion of PNS IPD isolates has changed little since introduction of conjugate vaccines. However, PNS IPD due to vaccine types has declined while PNS IPD due to non-vaccine types has increased. Continued surveillance is warranted to monitor changes in antimicrobial resistance and serotypes causing IPD in the conjugate vaccine era.

## Surveillance of invasive non-typeable *Haemophilus influenzae* in Alaska, 1980–2013

### Tammy Zulz, Karen Rudolph and Debby Hurlburt, Marcella Harker-Jones, Alisa Reasonover and Michael Bruce
Centers for Disease Control and Prevention, Arctic Investigations Program, Atlanta, GA, USA, tsc3@cdc.gov

#### 

*Background*. CDC’s Arctic Investigations Program (AIP) has conducted surveillance of invasive *Haemophilus influenza* (Hi) disease in Alaska since 1980. Historically, Hi type b (Hib) caused most disease; after introduction of Hib vaccine in 1991, other Hi types have caused most invasive disease. We report on non-typeable Hi (NT-Hi). *Methods*. We defined invasive Hi as an isolate from a normally sterile site in a surveillance region resident. Isolates forwarded to AIP were confirmed, serotyped and tested for antimicrobial susceptibility. Serotyping was performed by slide agglutination; NT-Hi were identified by a negative test for serotypes a–f. Data reported are for years 1980–2013. *Results*. Among 1,254 Hi cases reported, 1,045 isolates were serotyped; of those, 185 (17%) were NT-Hi. The proportion of total Hi comprised by NT-Hi has increased significantly (p<0.0001) over time; from 4.5% (1980–1992) to 45% (2003–2013). Overall rates of NT-Hi invasive disease from 1980 to 2013 were 0.96/100,000 (range 0–1.9/100,000) compared to 0.99/100,000 for the U.S. (1999–2008). Disease rates in Alaska Native people were higher (1.9/100,000) than in non-Native people (0.8/100,000) (p<0.0001). Fifty-one percent of cases were male. Age ranged from newborn to 93 years (median 51.3 years). Pneumonia with bacteraemia was the most common clinical presentation (47%). Since 1999, 46% of NT-Hi pneumonia cases had chronic lung disease; 95% of these occurred in adults >35 years old. Thirty-six deaths (case-fatality ratio 21% vs. 17% for the U.S. [p=0.29]) occurred; median age of fatal cases was 61.6 years (range newborn to 93 years). Isolates (n=160) were susceptible to ceftriaxone (100%), chloramphenicol (99%), ampicillin (75%) and trimethoprim sulfa (70%). *Conclusions*. Rates of invasive NT-Hi are higher in Alaska Native people compared to non-Native people in Alaska. Chronic lung disease is commonly associated with cases presenting with bacteraemic pneumonia. The high case-fatality ratio warrants further study.

## *Haemophilus influenzae* type a infections causing invasive disease in Utah children

### Hillary Crandall^1^, Jarrett Killpack^1^, Beth D. Knackstedt^1^, Andrew T. Pavia^1^, Krow Ampofo^1^, Anne J. Blaschke^1^, Mandy Dickey^2^ and Judy A. Daly^2^
^1^University of Utah, Salt Lake City, UT, USA, hillary.crandall@hsc.utah.edu; ^2^Primary Children’s Hospital, Salt Lake City, UT, USA

#### 

*Haemophilus influenzae* type b (Hib) has long been known to cause severe invasive disease in non-immune infants and children. The incidence of Hib has dramatically decreased in many areas with the widespread use of conjugate vaccines, however there is concern that serotype replacement could occur. In fact, several clusters of *H*. *influenzae* type a (Hia) infections with clinical features that resemble Hib have been reported in certain geographic areas. In Utah, we continue to have Hia causing invasive disease in children, with an incidence of 1.6 per 100,000 children per year in children less than the age of 5 years. Here we report on 29 cases occurring over a 7-year period from 2007 to 2014. The median age of patients was 9 months (range: 1–162 months). Sixteen of twenty-nine children had meningitis, eight had bacteraemia with an upper or lower respiratory source, two had bone or joint infections, while one had bacteraemia with purpura fulminans and died. Invasive Hia disease occurred at all times of the year, however about 75% of cases occurred in winter months. We collected all available isolates (23/29) and performed whole genome sequencing and evaluated the relatedness of isolates by determining the multilocus sequence type from assembled genomes. The majority (80%) of cases of invasive Hia in our population were caused by three distinct sequence types (56, 62 and 576). We describe a persistence of severe disease caused by Hia that resembles the disease caused by Hib, indicating that Hia has the potential to emerge as a significant pathogen. Further understanding of *H*. *influenzae* epidemiology and virulence, as well as vaccine development, will be important in future disease prevention and treatment.

## Immune recognition of Mycobacterium tuberculosis antigens and the risk of tuberculosis in Greenland

### Sascha Wilk Michelsen^1^, Bolette Soborg^1^, Else Marie Agger^1^, Lars Jorge Diaz^1^, Soren Tetens Hoff^1^, Anders Koch^1^, Hans Christian Florian Sorensen^2^, Karen Bjorn-Mortensen^1^, Peter Lawætz Andersen^1^, Jan Wohlfahrt^1^ and Mads Melbye^1^
^1^Statens Serum Institut, Denmark, swm@ssi.dk; ^2^Tasiilaq Hospital, Greenland

#### 

*Background*. *Mycobacterium tuberculosis* (*Mtb*) antigens expressed during latent tuberculosis infection (LTBI) may potentially distinguish individuals who successfully control LTBI from those who progress to develop tuberculosis (TB) disease. LTBI antigens may, therefore, be of use in future TB diagnostics and post-exposure TB vaccines. This study aims to evaluate LTBI antigen recognition in a Greenlandic population who, due to the Arctic environment, are virtually free of exposure to cross-reacting non-tuberculous mycobacteria. Furthermore, the study aims to determine if LTBI antigen recognition is associated with reduced incidence of TB. *Methods*. In 2012, East Greenlanders who aged 5–31 years were included in a cohort study and followed through 2014. A personal identifier given to all Greenlanders at birth identified the study participates and allowed individual level follow-up in the TB notification system. Immune recognition of LTBI antigens (Rv2660, Rv1284, Rv2659a-c) was assessed by whole blood antigen stimulation and subsequent measurement of interferon gamma (IFNy) by enzyme-linked immunosorbent assay (ELISA). Hazard ratios (HRs) of TB notification by antigen recognition were estimated using Cox regression. *Results*. A total of 978 participants were included of which, 67 had been diagnosed with TB previous to study entry. The proportion of participants recognizing at least one LTBI antigen was 448 (46%); 4% (Rv2660), 11% (Rv1284) and 34% (Rv2659b). Among 911 participants with no prior TB history, 31 (3%) were notified with TB during follow-up. Recognition of selected LTBI antigens was not associated with decreased risk of a subsequent TB diagnosis; Rv2660 HR 1.65 (95% CI 0.39–6.90), Rv1284 HR 0.99 (95% CI 0.30–3.25), Rv2659 HR 1.12 (95% CI 0.55–2.26). *Conclusions*. LTBI antigens were recognized among almost half of the study participants. This study does not support that immune recognition of the selected LTBI antigens is strongly associated with reduced incidence of TB.

## Ten years of tuberculosis intervention in Greenland – has it prevented cases of childhood tuberculosis?

### Emilie Birch^1^, Mikael Andersson^1^, Anders Koch^1^, Flemming Stenz^2^ and Bolette Søborg^1^
^1^Statens Serum Institut, Denmark, embi@ssi.dk; ^2^The National Board of Health, Greenland

#### 

*Background*. The incidence of tuberculosis (TB) disease in Greenland doubled in the 1990s. To combat the increase, national TB interventions were initiated in 2000 and strengthened in 2007. *Objective*. To determine whether the effect of interventions could be detected, we estimated the TB disease risk among children <16 years of age before and after interventions were implemented. *Design*. For a study cohort, we recruited all children <16 years of age included in the Greenlandic Civil Registration System (CRS) from 1990 to 2010. The CRS identifier was used to link cohort participants with TB cases identified based on the Greenlandic National TB registry. Bacille Calmette Guerin (BCG) vaccination status was identified through year of birth, as BCG was offered to newborns born either before 1991 or after 1996. Years with interventions were defined as 2000–2006 (primary interventions) and 2007–2010 (intensified interventions). Risk of TB was estimated using Poisson regression. *Results*. The study included 35,858 children, of whom 209 had TB disease. The TB disease incidence decreased after interventions were implemented (2007–2010: IRR [incidence rate ratios] 0.62, 95% CI 0.39–0.95, p=0.03, compared with the 1995–1999 period). The TB disease risk was inversely associated with BCG vaccination (IRR: 0.54, 95% CI 0.41–0.72, p<0.001). *Conclusions*. Years with national TB interventions in Greenland, including neonate BCG vaccination, are associated with a lower TB disease incidence among children <16 years of age.

## Organization of the bacteriological diagnosis and treatment of tuberculosis in Yakutia

### Alexander F. Kravchenko, Galina I. Alekseeva and Maria K. Vinokurova
Research & Practice Center for Tuberculosis of the Sakha Republic (Yakutia), Russia, alex220560@yandex.ru

#### 

*Introduction*. Yakutia is the largest region of Russia (3.2 million km^2^, with 40% lying beyond the Arctic Circle), known for long (50–3,000 km) distances between administrative districts and Yakutsk, the capital, low average population density (0.3–1 per km^2^, and a strongly continental climate). Vastness and extreme environment present challenges to bacteriologic service organization in Yakutia. We developed a model of bacteriologic service, consisting of the Central Laboratory in Yakutsk and laboratories in the district tuberculosis (TB) dispensaries (early treatment clinics). Diagnosis is done in two steps. Step 1 is performed by primary network clinics or TB dispensaries. Step 2 is performed strictly by laboratories in TB dispensaries. The percentages of new pulmonary cases positive for *Mycobacterium tuberculosis* (MTB) in 2012, 2011 and 2010 were 51.2%, 55.6% and 54.1%, respectively. Since the introduction of BACTEC MGIT-960 and GeneXpert Dx systems, final results are ready in 21.1 days on average, compared to 64.6 days using classic methods. Cryogenic preservation using natural frost did not influence MTB detection rate, using BACTEC and GeneXpert systems. Also, real-time PCR laboratory started to function in 2013. Patients with established diagnoses of TB undergo their treatment in the TB center, which offers special departments for surgical treatment of pulmonary and extra-pulmonary TB, pediatric TB and MDR-TB. The effectiveness of chemotherapy was nearly 85% in the absence of MDR, and more than 65% in MDR cases, but was significantly lower in relapsed and chronic TB. *Conclusions*. TB service in Yakutia is well equipped for diagnosis and treatment of TB. Use of up-to-date technologies shortens the time to diagnosis and start of adequate chemotherapy, and the use of new treatment methods for TB and concurrent diseases. Establishment of the TB center in the challenging conditions of a circumpolar region allows sufficient TB surveillance.

## Determination of NAT2 acetylation status in the Greenlandic population – an enzyme related to tuberculosis therapy

### Frank Geller, Bolette Soborg, Anders Koch, Sascha Wilk Michelsen, Bjarke Feenstra and Mads Melbye
Statens Serum Institut, Denmark, ako@ssi.dk

#### 

N-acetyltransferase 2 (NAT2) is a well-studied phase II xenobiotic metabolizing enzyme relevant in drug metabolism and cancerogenesis. NAT2 activity is largely determined by genetic polymorphisms in the coding region of the corresponding gene. We investigated NAT2 acetylation status in 1,556 individuals from Greenland based on four different single nucleotide polymorphism (SNP) panels and the tagging SNP rs1495741. There was a good concordance between the NAT2 status inferred by the different SNP combinations. Overall, the fraction of slow acetylators was low with 17.5% and varied depending on the degree of Inuit ancestry; in individuals with less than 50% Inuit ancestry, we observed more than 25% slow acetylators reflecting European ancestry. Greenland has a high incidence of tuberculosis and individual dosing of isoniazid according to NAT2 status has been shown to improve treatment and reduce side effects. Our findings could be a first step in pharmacogenetics-based tuberculosis therapy in Greenland.

## Tuberculosis

### Zagoruiko Galina
ICC Chukotka, Anadyr, Russia, sweetheart17@yandex.ru

#### 

In my presentation, I will be covering the following: Overview of the work of the district antituberculosis (ATB) service; the organizational structure of the ATB department; a review of the epidemiological situation in the district; prevention of tuberculosis; the organization of preventive measures of early tuberculosis in children and adolescents; tuberculosis treatment: key principles, prevention and tuberculosis rehabilitation in children and adolescents.

## Educational Programs

### Indigenizing the Canadian physician workforce: a perspective from Nunavut

#### Madeleine Cole
Qikiqtani General Hospital, Canada, mcole@gov.nu.ca

##### 

The very low numbers of indigenous physicians in Canada make it clear that despite some efforts on the part of medical schools to recruit indigenous students, there is a long way to go. While the legal profession has worked to build an indigenous legal community, medicine is woefully behind. In addition to creating a physician workforce that is more reflective of Canada’s population, there is the challenge of training non-indigenous medical learners and practicing physicians to provide better informed and more culturally safe care to indigenous patients. If you are from one of Canada’s three northern territories, there are numerous barriers to pursuing a medical education. Social capital (meaning the role-modelling, connections and opportunities that are almost pre-requisites to admission to medical schools) is so much harder to come by. Financial barriers are also immense when one considers the cost of a medical education and the poverty faced by many northern indigenous families. The number of Inuit physicians in Canada can be counted on one hand while a significantly higher number of Greenlandic Inuit are practicing medicine. Canada remains the only polar nation without a university in the arctic which creates an additional geographic hurdle in the path to medical education. This poster describes challenges that young people (particularly First Nations, Inuit and Metis) in Canada’s north experience in accessing health careers. A circumpolar lens is used to collect success stories and reflect on how the medical profession, can support, and encourage action by government and indigenous leadership to move forward positively such that becoming a physician is a wanted and achievable career path for Inuit and other indigenous Canadians.

## Towards an orientation of non-violence – exploring prevention of violence as a challenge of education, participation and sustainable change

### Suvi Pihkala, Mervi Heikkinen and Vappu Sunnari
University of Oulu, Oulu, Finland, suvi.pihkala@oulu.fi

#### 

Violence in intimate partnership is a specific and largely silenced problem for health, security and human rights and is so also for the Arctic. Violence and its prevention are challenged by historical, cultural, social and psychological factors that influence how people conceptualize violence and how it is silently accepted (1). Participation in and the construction of networks and measures for violence prevention and support is hindered by the vast distances in the Arctic. Systematic multisectoral and multidisciplinary education specializing in violence and abuse is not available in Finland, and the situation is not much better in the EU or globally. There is a need to provide and co-construct knowledge on the multiple forms of violence, their consequences and interventions against violence, and to build structures to support work against violence in professional practices and in personal lives (2). In order to respond to the problem, Women’s and Gender Studies in the University of Oulu constructed and is coordinating a multisectoral and multidisciplinary 25 European Credits Transfer System (ECTS) e-learning programmes specializing in these issues together with several national and international partners. In this presentation, we discuss the challenges and possibilities for supporting an orientation of non-violence in the context of the e-learning programme. We illustrate the discussion with a case study of one of the students in our e-learning programme. We are interested in what and how the student we chose for our case study writes and tells about violence and what and how she writes and tells about herself as an active agent in her life. In our preliminary analysis, we emphasize the conceptualizations of the “self” in the context of feeling, belonging, authenticity and interdependency; participation and sustainability – paying attention to the orientation of non-violence as a constantly evolving long-term matter.

**References**

1. Arctic Human Development Report II. Regional Processes and Global Linkages (eds. Larsen JN and Fondahl G). Tema Nord, ISSN0908-6692; 2014:567.

2. Sosiaali – Ja terveysministeriö. Naisiin kohdistuvan väkivallan vähentämisen ohjelma. Sosiaali – a terveysministeriön julkaisuja; 2010 [cited 2015 Jan 30]: [p. 5]. Available from: http://urn.fi/URN:ISBN:978-952-00-3031-5

## Specifics of providing medical aid in hard-to-reach northern regions

### Galina Degteva^1^, Nadezhda Dubinina^1^, Leonid Zubov^1^, Yana Korneeva^1^, Svetlana Malyavskaya^1^ and Natalia Simonova^1,2^
^1^Northern State Medical University, Russia; ^2^Northern (Arctic) Federal University named after MV Lomonosov, Russia, n23117@mail.ru

#### 

When organizing medical aid in northern regions, it is necessary to take into account the following: the remoteness and hard accessibility of the territories, the specifics of the lifestyle, the specifics of the “northern” physiology and pathology, ethnic features and current climate changes. Among the regional features affecting the health of the indigenous minorities of the North and complicating the organization of medical aid are harsh climate and environmental conditions, vast territories; low social and cultural development level, nomadic life; uneven development of the network of health care facilities due to geographic and demographic conditions. One of successful organizational forms for resolving medical and social problems of nomadic reindeer-breeders’ families was project “Kaninsky Krasny Chum.” Main tasks of the project were moving medical and cultural services closer to team camps, to study people’s health, their social and hosehold problems. Another area for improving medical aid provided to nomadic reindeer-breeders’ families can be the system of training health and medical assistants for reindeer-breeding teams. This will enable to enhance the nomadic people’s medical and sanitary-hygienic knowledge and to carry out preventive work in teams, and will provide a considerable economic benefit by eliminating the need to call an air ambulance in case of a mild illness that can be treated directly on site. The quality of medical aid rendered to the population residing in hard-to-reach territories of the Far North can also be improved using modern telemedicine technologies that provide vast opportunities for receiving consulting medical assistance by general practitioners, thus saving money for salaries to a large staff of highly specialized doctors.

## Assessment and enhancement of community development competencies in Canada’s northern public health workforce: a pan-territorial approach

### Kerry Lynn Durnford, Pertice Moffitt and Marnie Bell
Aurora College, Fort Smith, Canada, marnie.bell@northwestel.net

#### 

Community development is a key competency for public health and social service workers around the world. The purpose of this presentation is to share the results of a public health initiative and learning needs assessment of a circumpolar public health workforce. An environmental scan in 2009 indicated that community development training and orientation was needed for northern nurses and community health workers in Canada’s Northwest Territories (NWT). In addition, the northern health care service struggles with a chronic issue of recruitment and retention of community health care providers, establishing a need for professional development. Aurora College received funding from the Public Health Agency of Canada for a pan-territorial (Yukon College, Aurora College, Nunavut Arctic College) project to assess community development competencies for public health workers across Canada’s North. Since the inception of the 3-year project in 2012, community development competencies were adapted and an in-depth survey launched to assess learning needs of the public health workforce in the Yukon and NWT. Partnership with territorial governments at all stages of the project ensured a community-driven approach. Results of the survey indicated strengths of public health workers, including building community capacity, establishing relationships, appreciating diversity and varied cultural practices. Learning needs were identified by workers and employers in the areas of utilizing problem solving and conflict resolution skills, creating collaborative methods and capacity building. An online learning module was developed in 2014 by the pan-territorial team and piloted with public health providers in the three territories. The academic and political collaboration in three circumpolar regions provides valuable learning for the future of effective and sustainable public health care in the North.

## Developing higher education in the circumpolar north – experiences from the MCH master’s programme

### Sanna-Mari Ahonen, Ulla Timlin and Arja Rautio
University of Oulu, Oulu, Finland, sanna-mari.ahonen@oulu.fi

#### 

*International Master’s Degree Programme in Health and Wellbeing in the Circumpolar Area (MCH)* is one of the University of the Arctic’s 2-year master’s programmes and a part of the Barents Cross Border University. Since 2009, MCH has educated circumpolar-oriented professionals for different positions within health care, well-being and research. Master’s in health sciences degree is awarded by the University of Oulu. With international collaboration as a guiding principle, MCH has been established and implemented by an international partner university network, in order to bring together expertise in special features and challenges related to health and well-being in the circumpolar area. In addition to academic content development, the curriculum has been constantly developed in terms of learning and teaching methods, aiming to meet the guidelines set for higher education programmes, and the needs of students. Programme quality has been developed on the grounds of student feedback and self-evaluation. In this presentation, the central experiences of programme development are discussed and highlighted in order to facilitate good practices within circumpolar higher education.

## Quality nursing education through decentralization: establishing a circumpolar northern and indigenous workforce

### Carol Bullin^1^, Jill Bally^1^, Jill Butler^1^, Heather Exner-Pirot^1^, Emmy Neuls^1^, Bente Norbye^2^, Mari Wolff Skaalvik^2^ and Mark Tomtene^1^
^1^University of Saskatchewan, Saskatoon, Canada, carol.bullin@usask.ca; ^2^University of Tromso – The Arctic University of Norway, Tromso, Norway

#### 

*Background*. As the future workforce, nursing students can act as knowledge brokers, transforming the face of the health care system in the circumpolar north. Today, more students across the circumpolar north are graduating from high school, providing an opportunity to change the long-term outlook that suggests the unemployment rate will be four times higher in the north than in the south. *Purpose*. To provide opportunities for better engagement and inclusion of indigenous communities in both educational programming and research in the circumpolar north. *Goals*. Global collaboration will provide a comparative perspective for adapting distributed/decentralized programming to the north to contribute to improved health education and service delivery. Building capacity with health human resources educated with this technology, positions the circumpolar north to lead in creating a high quality, knowledge intensive environment for recruitment and retention of health professionals to rural and remote northern areas. A second goal is to establish whether the delivery of the Bachelor of Nursing Program to northern regions using this technology, is more cost effective, and ultimately, more sustainable. *Methods*. 1) Review the pedagogy of teaching and learning methods used in distributed/decentralized nursing education in northern regions, 2) conduct an integrative review of the literature to explore the methods presently used for outreach and community engagement in providing nursing education, 3) document lessons learned from the educational models that can be used for further development and delivery of skills training to build professional capacity in northern communities and regions and 4) conduct global faculty exchanges. *Outcome*. Linking quality of education, socio-economic well-being and health within a distributed/decentralized initiative will provide compelling evidence to assist policy and decision makers in determining the best long-term, sustainable investment.

## Developing a public health course on adverse childhood experiences (ACEs) and promoting resilience in the North

### Linda Chamberlain
Alaska Family Violence Prevention Project, Homer, Alaska USA, linda.chamberlain@alaska.gov

#### 

The University of Alaska at Anchorage will launch the first public health course on adverse childhood experiences (ACEs) and resiliency in the north. This distance learning course, designed to attract students throughout the circumpolar world, will embrace technology and the concept of socialstructing to facilitate access to resources, data and networking (1,2). As described on the Centers for Disease Control and Prevention website, the ACE Study has led to international awareness of the long-term impact of early trauma on health. An expanding body of research has examined the connection between ACEs and leading health concerns such as obesity, substance abuse and suicide in circumpolar nations (3). This graduate level course will explore how communities are using these data to become more trauma informed and how their experiences can be translated to circumpolar communities and populations. This workshop will engage in a dialogue about barriers, potential harms and culturally appropriate strategies for sharing this sensitive information in ways that instill hope and promote resilience among diverse northern populations that may be struggling with the intergenerational transmission of ACEs. Lessons learned from Alaskan youth/peer educators who are reframing the ACEs information within a resiliency framework will be discussed. The course syllabus which includes topics such the neuroscience of trauma, addressing vicarious trauma as the first step of becoming trauma informed and the culture of resiliency will be reviewed. Discussion will solicit input from participants to identity protective factors, strengths and considerations related to culture, lifestyle and northern traditions that should be highlighted as well as suggestions to enhance global participation from the circumpolar world. Workshop participants will provide invaluable insights that will shape this course.

**References**

1. Centers for Disease Control and Prevention. Injury prevention & control: division of violence prevention. [cited 2015 Jan 9]. Available from: http://www.cdc.gov/violenceprevention/acestudy/

2. Marina Gorbis. The nature of the future: dispatches from a socialstructed world. New York: Free Press; 2013.

3. Halonen J, Vahtera J, Kivimaki M, Pentti J, Kawachi J, Subramanian SV. Adverse experiences in childhood, adulthood neighborhood disadvantage and health behaviors. J Epidemiol Community Health. 2014;68(8):741–6.

## Development of Health Care I

### Ilagiinut. An ethnography of a community based participatory research project for families of Nunavik

#### Sarah Fraser^1^, Jennifer Hunter^2^ and Raymond Mickpegak^2^
^1^Université de Montreal, Montreal, Canada, sarah.fraser.1@umontreal.ca; ^2^Makivik Corporation, Saint-Laurent, Canada

##### 

In 2007 and later in 2010, the commission of the rights of the child determined that over 30% of Inuit children of Nunavik were being reported to child welfare agencies (Sirois & Montminy, 2010). Faced with these important social issues and the lack of adequate services,the Regional Partnership Committee (RPC), a committee composed of Inuit leaders of Nunavik, proposed the development of community prevention programs for young children and their families. The ultimate objective of the community mobilization would be to reduce the number of reports and placement under child welfare services.The project is based on core values of action research. The objective of this talk is to describe the process of community mobilization in this specific community. A variety of methods were used including detailed field notes, recording of meetings, formal and informal interviews. Two emerging topics will be discussed: 1) processes of empowerment and disempowerment in community development in a context of sociopolitical marginalization, 2) the complexities of organizational development in a culturally appropriate fashion. We rely on concepts and theories such as community readiness, insider/outsider and empowerment research to reflect upon our experiences with community mobilization in this Inuit community. We conclude that despite the presence of numerous best practice guidelines on community development, the steps towards community mobilization are emotionally and psychologically demanding for community members and include moments of disempowerment that can only be understood in a sociopolitical, historical and cultural contexts. Any best practice programs must first and foremost promote and enhance culturally and socially safe environments where community members can slowly learn to work together. This process requires reflections related to the role of outsiders in community development and the nature of relationships between outsiders in community development.

## Policy and practice options for equitable access to primary health care for indigenous peoples in British Columbia and Norway

### Josée Lavoie
University of Manitoba, Winnipeg, Canada, josee.lavoie@umanitoba.ca

#### 

Over the past three decades, policy reforms have been geared towards improving quality of care, responsiveness and equitable access to health care services for all social groups in general, and individuals living in marginalizing circumstances in particular. The purpose of this study was to document how primary health care (PHC) services are provided in Norway and British Columbia to meet the needs of indigenous peoples and use this knowledge to critically explore policy alternatives that inform the delivery of PHC for vulnerable populations. Findings show that in British Columbia, indigenous-specific PHC services have been the preferred mechanism to ensure better care. This is not the case in Norway, where Sámi-centric services exist only in mental health and only in Finnmark.

**References**

1. Lavoie JG. Policy and practice options for equitable access to primary health care for indigenous peoples in British Columbia and Norway. Int Indig Policy J. 2014;5(1):1–17.

2. Browne AJ, Varcoe CM, Wong ST, Smye VL, Lavoie J, Littlejohn D, et al. Closing the health equity gap: evidence-based strategies for primary health care organizations. Int J Equity Health. 2012;11:59.

3. Lavoie JG. Policy silences: why Canada needs a National First Nations, Inuit and Metis health policy. Int J Circumpolar Health. 2013;72:22690. doi: http://dx.doi.org/10.3402/ijch.v72i0.22690

## “Health care kalaallisut” – development of health care in Greenland through involvement of patients’ cultural perspectives on health practice

### Tine Aagaard
Ilisimatusarfik/University of Greenland, Nuuk, Greenland, tiaa@pi.uni.gl

#### 

*Background*. In the West, patient involvement in health care has become a major issue. It is grounded in considerations about, for example, human rights and ethical demands. Likewise, current political health strategies in Greenland emphasize patient involvement. Although to date no systematic initiatives to create the necessary conditions have been taken, such developments are imminent. *Aim*. The aim is to show that the involvement of patients’ specific cultural and historic conditions for living their everyday lives with illness, how they handle it, and their rationales is essential in order to develop health practice so that it is more relevant and useful for patients. *Theoretical–methodological approach and material*. In governmental strategies, health care contributions are mainly defined as purely professional. Other approaches recognize the patients’ perspectives as providing specific knowledge about individual patients’ everyday lives and how they themselves understand their situation. The research reported here is in accordance with the latter. The analyses are based on empirical material from my Ph.D. dissertation (1). A number of patients were followed for 3 years involving interviews and participant observations during hospitalization and in daily life in Nuuk, towns and settlements. *Results*. Neglect of patients’ opportunities and problems in everyday life often leads to a reduced effect of skilful, well-intentioned and costly professional initiatives. The conditions often impede the professionals’ ability to involve their patients’ perspectives. In addition, professional knowledge is more valued than is patients’ knowledge of their own lives. The implementation of strategies for user involvement must include conditions that can facilitate patients’ knowledge about their everyday lives with illness, their values and wishes in order to develop health practice in a culturally sensitive way.

**Reference**

1. Aagaard T. “Everyday life with illness – patients’ cultural perspectives on health practice in Greenland. [PhD dissertation]. Nuuk: University of Greenland; 2014.

## The expected future of primary health care centre from customers point of view – mixed method research

### Leena Paasivaara and Hanna Tiirinki
Univeristy of Oulu, Oulu, Finland, hanna.tiirinki@oulu.fi

#### 

Finnish primary health care covers primary medical care and public health. Health care is changing and customer orientation has growed dynamically. In Finland, social and health care system is going on one of the most massive structural reform. The research task was to investigate the cultural meanings associated with primary health care centres from customers’ point of view. The goal was to understand the expectations of future health care centres from the point of view of customers. In the theoretical frame of the study, new public service theory and a cultural model to conceptualize cultural meanings related to health care centres was utilized. A mixed methods approach was used in this study, utilizing both qualitative and quantitative data. The first phase of the empirical part involved investigating cultural meanings from the customer’s viewpoint with the aid of documents (N=621). The second phase consisted of virtual anthropological material (N=250). The third phase comprised a survey (Northern Finland birth cohort 1966) (N=200 and N=3,237). Qualitative material (phases I, II and parts of phase III of the empirical part) were analysed using inductive and deductive content and text analysis. Quantitative material (parts of phase III of the empirical part) were analysed statistically. In the study, the expected future model described expect for the health care centres future from the customers point of view. Cultural meanings related to expectations concerning the future of the health care centre, describing a generative and functional local health care centre with a set of values based on holisticity. It is important to examine the future-oriented cultural meanings associated with primary health care centres from customers point of view when developing and reforming the national primary health care system. The study generated new knowledge that can be utilized in improving public health care.

**References**

1. Tucker CM, Arthur TM, Roncoroni M, Wall W, Sanchez J. Patient-centered, culturally sensitive health care. Am J Lifestyle Med. 2013. doi: http://dx.doi.org/10.1177/155982761349806

2. Mauri AG, Muccio S. The public management reform: from theory to practice. The role of cultural factors. Int J Adv Manage Sci. 2013;1(3):47–56.

3. Fielding NG. Triangulation and mixed method design: data integration with new research technologies. J Mix Methods Res. 2012;6(2):124–36.

## Effect of the 13-valent pneumococcal conjugate vaccine on nasopharyngeal carriage by respiratory pathogens among Greenlandic children

### Johan Navne^1^, Malene Børresen^1^, Hans-Christian Slotved^1^, Mads Melbye^1^, Karin Ladefoged^2^ and Anders Koch^1^
^1^Statens Serum Institut, Copenhagen, Denmark, mlb@ssi.dk; ^2^Dronning Ingrids Hospital, Nuuk, Greenland

#### 

*Background*. In November 2010, Greenland introduced the 13-valent pneumococcal conjugate vaccine (Prevnar 13®; Pfizer/Wyeth – PCV-13) in the children vaccination program. Recent studies indicate changes in rates of nasopharyngeal bacterial carriage post-PCV introduction. We aimed to evaluate the impact of the PCV-13 on nasopharyngeal carriage by four clinically relevant bacteria frequently associated with respiratory infections in children. *Method*. In 2013, a cross-sectional population-based study was conducted among Greenlandic children aged 0–6 years and data compared with a previous carriage study from 2011. PCV-13 status was obtained through nationwide registries. Nasopharyngeal samples tested for *Streptococcus pneumoniae*, Non-typeable *Haemophilus influenzae* (NTHi), *Moraxella catarrhalis* and *Staphylococcus aureus* among others. Pneumococcal serotyping was performed by Quellung reaction and serotype-specific antisera. Statistical analysis included logistic regression models, adjusting for known risk factors. *Result*. A total of 377 nasopharyngeal samples were collected. Overall carriage rate of *S*. *pneumoniae* remained unchanged from 2011 to 2013 (51 and 56%, p=0.13), but significant serotype shifts were observed both among vaccinated and unvaccinated. Carriage rate of *S*. *aureus* significantly declined among vaccinated (aOR 0.48, 95% CI 0.25–0.91) whereas that of *M*. *catarrhalis* increased (aOR 1.52, 95% CI 0.99–2.33). *Conclusion*. PCV-13 introduction in Greenland has likely led to shifts in nasopharyngeal pneumococcal serotype distribution as well as reductions in *S*. *aureus* carriage and increasing carriage rates of the important otopathogen *M*. *catarrhalis* among vaccinated children. Continued surveillance is warranted to clarify if these changes are temporary or persistent, and if these changes may have an impact on the pattern of respiratory and invasive diseases in Greenland.

## Prenatal health information for expecting Inuit dads in Nunavut

### Amanda J. Sheppard
The Hospital for Sick Children, Toronto, Canada, amanda.sheppard@sickkids.ca

#### 

*Background*. It is imposed on the majority of pregnant women in Nunavut to leave their community 2 weeks before their due date. This colonial influence has excluded the expecting dad from much of his partner’s and baby’s care and delivery. Ultimately, this has changed his role, experience and knowledge relating to the pregnancy and birth. *Methods*. Collaboration with the Inuit Health Matters Advisory Board *Results and conclusion*. An online space and materials have been prepared for the expecting dad towards empowering him to choose actions that will encourage a healthy and well pregnancy for him and his growing family.

## Development of a trauma screening and brief intervention process for Alaska Native people in a primary care setting

### Denise Dillard^1^, Vanessa Hiratsuka^1^, Lisa Dirks^1^, Jaedon Avey^1^, Laurie Moore^2^, Barbara Beach^3^ and Douglas Novins^2^
^1^Southcentral Foundation, Anchorage, AK, USA, vhiratsuka@scf.cc; ^2^University of Colorado Denver, Denver, CO, USA; ^3^Cherokee Nation, Tahlequah, OK, USA

#### 

*Background*. Behavioural health disorders in primary care are common yet are not often recognized and, accordingly, are left untreated. Trauma-related health histories are not regularly completed in primary care settings despite the fact that many people with trauma seek physical, emotional and behavioural health care through primary care. Within this project, we used a community-based participatory research (CBPR) approach to develop a screening, brief intervention and referral process for trauma among Alaska Native/American Indian (AN/AI) adults in two primary care settings, Southcentral Foundation in Anchorage, AK and Cherokee Nation Heath Services in Tahlequah, OK. In this paper, we will describe the formative research process and findings used to develop a screening and brief intervention for trauma in primary care. *Methods*. A cross-site steering committee was formed to guide the research process. To inform future development of a pilot screening and brief intervention pilot project, we conducted iterative semi-structured interviews and focus groups with customer-owners, clinical providers and administrative leaders in two tribally owned and operated health systems. Data collected processes elicited participants’ views on trauma screening, brief intervention processes and referral for treatment. Data were transcribed and analysed for key themes. *Results*. The most common traumatic experiences mentioned were trauma related to physical or sexual abuse. Respondents reported that trauma services should be provided in a safe, timely, culturally appropriate and community-oriented manner. Preferences for screening for trauma differed by location. Process recommended for brief intervention differed based on available clinical and human resources. *Conclusion*. The process to conduct screening and brief intervention differed at each location due to available resources, particularly immediate access to behavioural health providers.

## Mental Well-being in the North I

### Adversity and adolescents’ mental health in Greenland – the role of resilience

#### Cecilia Petrine Pedersen^1^ and Peter Bjerregaard^2^
^1^Aarhus University, Aarhus, Denmark, cepp@edu.au.dk; ^2^University of Southern Denmark, Odense, Denmark

##### 

*Background*. In order to reduce the high prevalence of social vulnerability, suicidal behaviour and poor well-being among adolescents in Greenland, it is important to strengthen the knowledge of factors affecting adolescent’s mental health. Resilience refers to an individual’s healthy development despite exposure to adversity. The aim of the study is to examine how resilience mediates the association between adverse life events and mental health among adolescents in Greenland. *Material and methods*. The analyses are based on a cross-sectional survey, Well-being among Youth in Greenland 2011, among 15–16-year-old pupils from schools in seven towns. Resilience was measured by the Resilience Scale for Adolescents (READ) and mental health was measured by the Strengths and Disability Questionnaire (SDQ). The data contain information on adverse life events such as exposure to sexual and physical abuse and household dysfunction factors. Logistic regression analyses with indicators of adverse life events as exposure and self-reported mental health as outcome measure will be performed in relation to degree of resilience. *Results*. The response rate was 82% with a study population of 481 students, representing approximately 40% of all pupils in this age group in primary schools in Greenland. Preliminary results showed a high prevalence of adverse life events; 32% girls and 9% boys have been exposed to sexual abuse from peers or adults, 12% boys and 17% girls have been exposed to serious violence from parents, 10% have witnessed serious violence against mother and 20% have been exposed to parental alcohol abuse. Further analysis will provide knowledge on the association of adverse life events and adolescents’ mental health and how resilience mediates this association. *Implications*. Better understanding of the role of resilience in mental health will have important implications for interventions and policies and in strengthening the areas where adolescents’ conditions are vulnerable.

## “We’re like lemmings”: making sense of the cultural meaning(s) of suicide among the indigenous Sámi

### Jon Petter Stoor
Sámi Norwegian National Advisory Board on Mental Health and Substance Abuse, Bjurholm, Norway, jon.petter.anders.stoor@finnmarkssykehuset.no

#### 

*Background*. Suicide is a widespread problem in the circumpolar Arctic, and a major public health issue among the indigenous Sámi in Scandinavia. To adapt suicide prevention strategies that are culturally attuned, one must understand how suicide is understood within context, that is, the cultural meaning(s) of suicide. *Objective*. To explore and make sense of the cultural meaning(s) of suicide among Sámi in Sweden. *Design*. Five open-ended focus group discussions (FGDs) on “suicide among Sámi” were carried out with, in total, 22 Sámi informants in Sweden. FGDs were recorded, transcribed verbatim and analysed with content analysis. *Results*. Five themes emerged, including: “The Sámi world is close knit and multiplex,” “We are like lemmings fighting for our culture and the herders are in the middle of the fight,” “Suicide is a consequence of Sámi losing (or having lost) their identity,” “Suicide is almost contagious!” and “There is no help to get.” *Conclusions*. Findings indicate that suicide among Sámi in Sweden is framed by other Sámi within a threatened (Sámi) cultural context where suicide is an act that takes place, and makes sense to others, when a Sámi no longer has the means or power to maintain a Sámi identity.

## Entanglements of loneliness and depression experienced by young adults born in Northern Finland

### Anna Reetta Rönkä, Vappu Sunnari, Arja Rautio and Anja Taanila
University of Oulu, Oulu, Finland, anna.r.ronka@oulu.fi

#### 

Loneliness is multidimensional, subjective and negative experience affected by socioemotional, cultural and contextual factors. For some, it might become chronic condition with serious consequences to one’s health and well-being. Loneliness has been associated with psychosocial and mental health problems including low self-esteem, neuroticism, social anxiety and social phobia. Moreover, numerous quantitative studies have demonstrated correlations between loneliness and depression in adolescents and adults (1). Loneliness and depression are distinct phenomena, even though they share common causes and loneliness may play a causative role in the development of depression and vice versa (2). Little qualitative research has been conducted on these topics. Qualitative approach may capture more fully the socioemotional and situational factors in relation to these live experiences from the point of view of the person experiencing them (3). We examined the experiences of loneliness associated with depression in Northern Finland Birth Cohort 1986 data (N=9,432). Semi-structured interviews were conducted among 39 cohort members (age 27–28 years), who had experienced loneliness throughout their lives. The interviews were analysed with qualitative content analysis. The preliminary analysis show that as many as 24 (62%) of interviewees told about their depression or depressive mood and 15 of them were diagnosed for depression. The interviewees described the entanglements, causes and consequences between these negative experiences and the difficult emotions associated with them. Loneliness was strongly linked with depression. Their co-occurrence is alarming, since young people experiencing loneliness and depression may be at great risk for developing major depressive disorder, which eventually may cause inability to successfully integrate into society and may lead to social exclusion, disability or even morbidity. The possible prevention measures for loneliness will be discussed.

**References**

1. Heinrich LM, Gullone E. The clinical significance of loneliness: a literature review. Clin Psychol Rev. 2006;26(6):695–718. [cited 2015 Jan 25]. doi: http://dx.doi.org/10.1016/j.cpr.2006.04.002

2. Witvliet M, Brendgen M, van Lier PA, Koot HM, Vitaro F. Early adolescent depressive symptoms: prediction from clique isolation, loneliness, and perceived social acceptance. J Abnorm Child Psychol. 2010;38(8):1045–56. [cited 2015 Jan 25]. doi: http://dx.doi.org/10.1007/s10802-010-9426-x

3. Barg FK, Huss-Ashmore R, Wittink MN, Murray GF, Bogner HR, Gallo JJ. A mixed-methods approach to understanding loneliness and depression in older adults. J Gerontol B Psychol Sci Soc Sci. 2006;61(6):329–39. [cited 2015 Jan 25]. Available from: http://www.pubmedcentral.nih.gov/articlerender.fcgi?artid=2782769&tool=pmcentrez&rendertype=abstract

## Youth-identified protective factors for mental health and well-being in a changing climate: perspectives from Inuit youth in Nunatsiavut, Labrador, Canada

### Joanna Petrasek MacDonald^1^, Ashlee Cunsolo Willox^2^, James Ford^1^, Inez Shiwak^3^, Michele Wood^4^; IMHACC Team^5^
^1^McGill University, Montreal, Canada, macdonald08@gmail.com; ^2^Cape Breton University, Sydney, Canada; ^3^Word’ Storytelling and Digital Media Lab, Rigolet, Canada; ^4^Department of Health & Social Development, Nunatsiavut Government, Nain, Canada; ^5^Nain Inuit Community Government, Hopedale Inuit Community Government, Postville Inuit Community Government, Makkovik Inuit Community Government, Rigolet Inuit Community Government, Nain, Canada

#### 

Similar to other circumpolar regions, the Canadian Arctic is experiencing rapid changes in climatic conditions, with implications for Canadian Inuit communities widely documented. Inuit youth have been identified as an at-risk population, with likely impacts on mental health and well-being. This study identifies and characterizes youth-specific protective factors that enhance well-being in light of a rapidly changing climate, and examines how climatic and environmental change challenges these protective factors within Northern Labrador, Canada. This research was led by the Rigolet Inuit community government, in partnership with the other Nunatsiavut Inuit community governments of Nain, Hopedale, Postville and Makkovik and the Department of Health and Social Development. In-depth conversational interviews were conducted by local research coordinators with youth aged 15–25 years from the five communities. Five key protective factors were identified as enhancing their mental health and well-being: being on the land; connecting to Inuit culture; strong communities; relationships with family and friends and staying busy. Changing sea ice and weather conditions were widely reported to be compromising these protective factors by reducing access to the land, and increasing the danger of land-based activities. This work contributes to existing scholarship on Northern climate change health adaptation by identifying factors that may enhance youth resilience and, if incorporated into adaptation strategies, may contribute to creating successful and effective health adaptation responses and to fostering adaptive capacities.

## Exposure to maternal smoking during pregnancy and cognitive performance in early adulthood in the Northern Finland Birth Cohort 1986

### Hugh Ramsay^1^, Jennifer H. Barnett^2^, Jouko Miettunen^1^ and Juha Veijola^1^
^1^University of Oulu, Oulu, Finland, drhughramsay@gmail.com; ^2^University of Cambridge, Cambridge, United Kingdom

#### 

*Background*. While there is evidence that maternal smoking of tobacco in pregnancy is associated with neurobehavioural and cognitive deficits in children, there is little research examining longer term effects into adulthood. Significant confounders, including smoking in later life, further complicate studies of these associations. *Methods*. Using a nested case-control study design with group matching and significant exclusion criteria from the Northern Finland Birth Cohort 1986, this study examines the effect of maternal smoking in pregnancy on cognition in early adulthood. Univariate analysis with t-tests and chi-squared tests were followed by multivariate linear regression where differences were found on univariate testing. *Results*. Among those exposed to maternal smoking in pregnancy, a large number were also exposed in adolesence, adulthood or both (128/214), with 49/214 exposed at all three timepoints. Maternal smoking in pregnancy was borderline associated with lower level of education (p=0.064) and with poorer vocabulary score (p=0.079) by early adulthood but not other cognitive scores. Stratified by gender, maternal smoking in pregnancy was associated with vocabulary (p=0.025) and matrix reasoning (p=0.028) in males but not females. Associations with lower level of education and poorer cognitive performance were stronger for adolescent smoking and smoking in young adulthood. *Discussion*. Prenatal smoking may be associated with mild adverse cognitive performance and academic achievement in early adulthood, particularly in males, though this requires larger samples for confirmation. Adult smoking has the largest association with cognition and academic performance. These results suggest that smoking-reduction campaigns are beneficial across age groups.

## Growing Up Tobacco-Free (GUTF) in Alaska: a study to implement tobacco system changes into a Head Start programme in rural Alaska

### Karen Doster, Gary Ferguson and Crystal Meade
Alaska Native Tribal Health Consortium, Anchorage, AK, USA, gferguson@anthc.org

#### 

The Growing Up Tobacco-Free (GUTF) in Alaska Project addresses the high level of tobacco use within Head Start families to influence and increase healthy outcomes and educational achievement. The goal of GUTF is to build capacity for the Head Start system to address tobacco or any health risk a family may be facing, while ultimately helping children in Alaska grow up tobacco free. Using tobacco is the single greatest cause of preventable disease and death in the United States and Alaska. Tobacco use in Alaska contributes to more deaths than all other preventable causes combined. State of Alaska BRFSS statistics show that 38% of low SES non-natives use tobacco, 20% of adults between the ages of 18 and 29 years use tobacco and 36% of Alaska Native people use tobacco, all numbers that represent many of the families that are served by Head Start programs in Alaska. ANTHC worked with the RurAL CAP Head Start programme during a 3-year pilot period to establish a system to address tobacco use through education, resources and training in order to reduce the high prevalence of tobacco use. A model was created that addressed tobacco use through culturally appropriate education and training, evidence-based approaches to tobacco control, provision of easily accessible tobacco cessation services and evaluation to be implemented into RurAL CAP Head Start sites. At the end of the pilot period, 24 RurAL CAP Head Start sites were part of the project. A total of 589 families were surveyed (5,474 individuals). Of the individuals surveyed, there were 227 quit attempts and 63 successful quits. The project proved to be a promising approach to educate Head Start staff and families about the use of tobacco and implement a system within the RurAL CAP Head Start programme to address tobacco use among Head Start families. This approach can be tailored to address all behaviours and health and socio-economic issues in order to change social norms and improve the life of children growing up in Alaska.

## Functional mapping of dynamic happy and fearful facial expressions in young adults with familial risk for psychosis (FR)

### Johannes Pulkkinen, Juha Nikkinen and Juha Veijola
University of Oulu, Oulu, Finland, johannes.pulkkinen@oulu.fi

#### 

*Background*. Psychotic disorders and their prodromal states have been connected to impaired social functioning. We compared the brain activity between young adults with familial risk (FR) for psychosis and matched controls during visual exposure to emotional facial expression. Methods. In total, 51 FR and 52 control subjects were drawn from the Northern Finland 1986 Birth Cohort (Oulu Brain and Mind Study). Participants underwent functional MRI (fMRI) using visual presentation of dynamic happy and fearful facial expressions. FMRI data were processed to produce maps of blood oxygen level dependent (BOLD) responses for happy and fearful facial expressions, which were then compared between groups. *Results*. FR subjects had increased BOLD response in the superior frontal gyrus and supplementary motor area and reduced negative BOLD response in the paracingulate cortex during happy facial expressions. The FR group also showed a statistically significant linear correlation between mean amygdala BOLD response and facial expression recognition. PPI showed that there was a significant negative interaction between the amygdala and the dorsolateral prefrontal cortex (dlPFC) and superior temporal gyrus in FR subjects. *Conclusions*. Our results indicate abnormal function of PFC in FR subjects. This was also suggested by PPI, as the dlPFC showed decreased functional connectivity with the amygdala in the FR group. This may indicate that in FR subjects the amygdala have to take a greater role in emotion recognition and social functioning. This inference was supported by our discovery of statistically significant correlations between the amygdala BOLD response and emotion recognition in the FR group but not in controls.

## Indigenous Health IV: “Food Insecurity, Obesity and Diabetes”

### Establishment of the Study of Diabetes among Inuit Populations in the Arctic Network (ESDIPAN)

#### Torben Hansen^1^ and Marit Eika Jørgensen^2^
^1^University of Copenhagen, Copenhagen, Denmark; ^2^Steno Diabetes Center, Southern Denmark University, Odense, Denmark, maej@steno.dk

##### 

*Background*. The Danish Diabetes Academy has funded an international Arctic diabetes network. Diabetes is common among Inuit with 10% of adults being affected, and additionally 20% identified with pre-diabetes (1,2). Recently a genetic variant was discovered in TBC1D4 that increases the risk of diabetes with an odds ratio of 10.3 among 5,000 Greenland Inuit (3). The observed effect sizes are several times larger than any previous findings, and the gene variant explains about 15% of all diabetes in Greenland. Among Arctic Inuit, both information about the social transition and the disease pattern is exceptionally good, and it is therefore ideal to study gene-environment interactions on diabetes and for the study of epigenetics. An established collaboration already exists between research groups from Alaska, Canada and Denmark on methods standardization of cohort protocols for the circumpolar epidemiological study “Inuit Health in Transition.” *Methods*. The network will build on existing collaborations in the Arctic and will be complemented with relevant researchers to facilitate international co-operation with a potential long-term inclusion of studies of Indians and other populations in the North. *Milestones*. The planned activities of the ESDIPAN are as follows: June 2015: First satellite symposium will be held in connection with the ICCH16, in Oulu, Finland. Otober 2015: First network meeting will be held in Nuuk in connection with a PhD course. September 2016: Second network meeting will be held in Nuuk in connection with the NUNAMED conference. *Perspectives*. With ESDIPAN we hope to 1) build a strong network among diabetes researchers in the Arctic; 2) collaborate across research groups with focus on diabetes epidemiology, genetic and epigenetic studies and studies of implementation of novel technologies for complication screening; 3) set the standard for best practice diabetes research in the Arctic; and 4) educate younger diabetes researchers from Arctic countries.

**References**

1. Jørgensen ME, Bjeregaard P, Borch-Johnsen K. Diabetes and impaired glucose tolerance among the Inuit population of Greenland. Diabetes Care. 2002;25(10):1766–71.

2. Jørgensen ME, Borch-Johnsen K, Witte DR, Bjerregaard P. Diabetes in Greenland and its relationship with urbanization. Diabetes Med. 2012;29:755–60.

3. Moltke I, Grarup N, Jørgensen ME, Bjerregaard P, Treebak JT, Fumagalli M, et al. A common Greenlandic TBC1D4 variant confers insulin resistance and type 2 diabetes. Nature. 2014;512(7513):190–3. doi: http://dx.doi.org/10.1038/nature13425

## Living well with diabetes in Alaska

### Meera Narayanan, Cynthia Schraer and Judy Thompson
Alaska Native Tribal Health Consortium (ANTHC), Anchorage, AK, USA, mnarayanan@anthc.org

#### 

Clinicians recognize that some individuals, when diagnosed with diabetes, live many years with few, if any, complications. Others face a much shortened life span fraught with a great deal of morbidity. The Alaska Native Diabetes Program has begun an analysis based on a registry that was started in 1986 to elucidate factors that are associated with living at least 20 years after the diagnosis of diabetes without experiencing major morbidity. As of the end of 2012, 360 Alaska Native people were living in Alaska with diabetes, and had been diagnosed at least 20 years previously. Since this group was older than those with less than 20 years of diabetes, we identified for analysis the group of persons of age at least 65 years as of 31 December 2012. The group alive on 31 December 2012 and age 65 years or greater, included 249 people with ≥20 years of diabetes (the 20+ group) and 1,322 people with <20 years of diabetes (the 20− group). The 20+ group was somewhat older (more likely to be >75 years old), more likely to be Indian as opposed to Aleut or Yupik/Inupiat, and more likely to be female than those in the 20− group. Among those of age ≥75, the same pattern was apparent. Compared to the general Native population age ≥65 in Alaska, the 20− group had a similar proportion of women to men, while the 20+ group had a greater proportion of women. In preliminary analysis, we found that among those in the 20+ group, 50 (20%) had experienced at least one of the complications of myocardial infarction, stroke, lower extremity amputation or end stage renal failure, while 199 (80%) had experienced none of these major complications. In summary, a substantial number of Alaska Native people with diabetes have lived at least 20 years post diagnosis, without suffering any of four major complications. Future analysis of the 20+ group will explore indicators of well-being such as community living versus institutionalization, presence or absence of functional impairments, age at diagnosis and biologic parameters such as lipids, Hgb A1C, blood pressure and BMI, and treatment modality. We plan to perform survival analyses on both 20− and 20+ groups to examine time until complication or death for those who died prior to 1 January 2013.

## DNA methylation patterns are associated with n-3 fatty acid intake in Yup’ik people

### Stella Aslibekyan^1^, Howard Wiener^1^, Peter Havel^2^, Kimber Stanhope^2^, Diane O’Brien^3^, Scarlett Hopkins^3^, Devin Absher^4^, Hemant Tiwari^1^ and Bert Boyer^3^
^1^University of Alabama at Birmingham, Birmingham, AL, USA; ^2^University of California at Davis, Davis, CA, USA; ^3^University of Alaska at Fairbanks, Fairbanks, AK, USA, bert.boyer@gmail.com; ^4^Hudson Alpha Institute for Biotechnology, Huntsville, AL, USA

#### 

A large body of evidence links a high dietary intake of n-3 (ω-3) polyunsaturated fatty acids (PUFAs) with improved cardiometabolic outcomes. Recent studies suggested that the biologic processes underlying the observed associations may involve epigenetic changes, specifically DNA methylation. To evaluate changes in methylation associated with n-3 PUFA intake, we conducted an epigenome-wide methylation association study of long-chain n-3 PUFA intake and tested associations between the diabetes- and cardiovascular disease-related traits. We assessed DNA methylation at ~470,000 cytosine–phosphate–guanine (CpG) sites in a cross-sectional study of 185 Yup’ik Alaska Native individuals representing the top and bottom deciles of PUFA intake. Linear regression models were used to test for the associations of interest, adjusting for age, sex and community group. We identified 27 differentially methylated CpG sites at biologically relevant regions that reached epigenome-wide significance (P<1×10^−7^). Specifically, regions on chromosomes 3 (helicase-like transcription factor), 10 (actin α-2 smooth muscle/Fas cell surface death receptor) and 16 (protease serine 36/C16 open reading frame 67) each harbored two significant correlates of n-3 PUFA intake. In conclusion, we present promising evidence of association between several biologically relevant epigenetic markers and long-term intake of marine-derived n-3 PUFAs.

## Self-assessed food poverty among school children – a comparison between Greenland and northern Canada

### Birgit Niclasen^1^, Colleen Davison^2^, Nathan King^3^ and William L Pickett^2^
^1^National Institute of Public Health, Nuuk, Denmark/Greenland, bivn@nanoq.gl; ^2^Department of Public health Sciences, Queen’s University and Kingston General Hospital Research Institute, Kingston, Canada; ^3^Department of Public health Sciences, Queen’s University, Kingston, Canada

#### 

Despite the fact that populations in northern Canada and Greenland face similar challenges related to contamination and reduction of country foods and high dependence on imported products, issues of food security or food poverty in children in these regions have not been directly compared. Limitations of earlier studies include the lack of comparability due to the mix of measures and definitions used and dependence on information from caregivers rather than the young people themselves. In this study, self-assessed data derived from the same question on food poverty are directly compared between children in Greenland and northern Canada. The study uses data from the Health Behaviour in School-aged Children study, a WHO collaborative initiative. In Greenland, 13% in 2010 of 11–17-year-old school children reported to go school or to bed hungry “always” or “often” due to lack of food in the home while 19% experience it “sometimes.” The same figures were among northern Canadian school children 6% and 31%, respectively. The distribution of food poverty is compared in relation to community size and area-level affluence, family composition, parental employment and associated to the use of country food, family connectedness and family wealth. Implications for policy intervention are significant.

## Participatory food costing in a northern indigenous context

### Tiff-Annie Kenny^1^, Sonia Wesche^2^, Myriam Fillion^1^, Jullian MacLean^3^, O’Hara Shannon^3^ and Laurie H.M. Chan^1^
^1^Centre for Advanced Research in Environmental Genomics, Ottawa, Canada, Tiff-Annie.Kenny@uOttawa.ca; ^2^University of Ottawa, Ottawa, Canada; ^3^Inuvialuit Regional Corporation, Inuvik, Canada

#### 

Residents of Canada’s north often cite the high price of nutritious market foods as an obstacle to healthy eating and food security. As part of our broader food security work in the western Canadian Arctic, we report on an ongoing participatory food costing study in six communities of the Inuvialuit Settlement Region (ISR). The ISR, home to the Inuvialuit people, includes one road-accessible community and five plane-accessible communities. The 2007–2008 Inuit Health Survey (IHS) found that 33% of households in the ISR were moderately food insecure, with 13% experiencing severe food insecurity. Despite access in five of six communities to a federally administered food subsidy programme, there is concern within the region that food is unjustly expensive. While it is mandatory for food retailers to publish quarterly food price reports in remote communities, residents have expressed concern that these reports lack transparency and that the “voice” of communities is neither being heard, nor heeded, on the issue of food prices. This project was developed in a participatory manner with the ISR Food Security Working Group (FSWG), which includes representatives from community organizations, and the health, education and wildlife sectors. Participatory food costing is an established methodology for gathering food prices. It yields independent, internally generated food price data at the community level and empowers community members to contribute directly to the resolution of an important local food system issue. Data were collected in late 2014 and early 2015. The list of food items included in the costing study reflects the availability of healthy foods in remote/northern communities and the dietary habits of Inuit as reported in the IHS. This project will advance the development of a culturally appropriate northern food costing methodology. A strategy for knowledge translation and results dissemination is being developed in collaboration with the community research assistants.

## Ethnic differences in anthropometric measures and abdominal fat distribution: a comparative study among Inuit, Africans and Europeans

### Pernille Falberg Rønn^1^, Gregers Stig Andersen^1^, Torsten Lauritzen^2^, Dirk Lund Christensen^3,4^, Mette Aadahl^3,5^ and Bendix Carstensen^1^, Marit Eika Jørgensen^1^
^1^Steno Diabetes Center, Gentofte, Denmark, prfr@steno.dk; ^2^Aarhus University, Aarhus, Denmark; ^3^University of Copenhagen, Copenhagen, Denmark; ^4^University of Cambridge, Cambridge, United Kingdom; ^5^Research Centre for Prevention and Health, Glostrup, Denmark

#### 

*Background*. Evidence suggests that differences in obesity and cardiometabolic risk between populations may be explained by differences in abdominal fat distribution, particularly excess visceral fat. Ethnic differences in body composition have especially been identified as a limitation to the use of BMI. The relations between anthropometry and abdominal fat distribution are not clearly established across different populations. We aimed to examine how BMI and waist circumference (WC) are related to visceral adipose tissue (VAT) and subcutaneous adipose tissue (SAT) in three ethnic groups comprising an Inuit, African and European population. *Methods*. We combined data from four cross-sectional studies using similar methodology. The studies were conducted between 2005 and 2011 in Greenland (The Inuit Health in Transition Study), Kenya (The Kenya Diabetes Study) and Denmark (Helbred2008 and The ADDITION-Pro Study). A total of 7,118 individuals (3,102 Inuit, 1,139 Kenyans and 2,877 Danes) aged 17–95 years had measures of anthropometry, and VAT and SAT assessed by ultrasonography. Linear models were performed to analyse VAT and SAT as functions of BMI and WC controlled for age. The models were analysed separately for sex and ethnic group. *Results*. Africans had significantly lower mean values of BMI, WC, VAT and SAT compared to Inuit and Europeans, whereas the Inuit had the highest mean SAT values. The European women had higher levels of VAT, where a difference of 1 kg/m^2^ in BMI corresponded a 4.1% (95% CI: 3.9–4.4) higher VAT compared to Inuit women (3.6%, 95% CI: 3.4–3.8) and African women (2.8%, 95% CI: 2.3–3.2). In contrast, African women had significantly higher values of SAT for a given increase in BMI or WC, while the European had the lowest. Overall, the same pattern was seen for men. *Conclusions*. Differences in the relationship between anthropometry and abdominal fat distribution may contribute to explain differences in obesity-associated cardiometabolic risk across ethnic groups.

## Cannabis use in relation to obesity and insulin resistance in the Inuit population

### Michel Lucas^1^, Gerard Ngueta^1^, Richard Bélanger^1^ and Elhadji A. Laouan-Sidi^2^
^1^Laval University, Quebec City, Canada, michel.lucas@crchuq.ulaval.ca; ^2^Population Health and Optimal Health Practices Research Unit, Centre hospitalier universitaire de Québec (CHUQ) Research Centre, Quebec, Canada

#### 

*Objective*. To ascertain the relationship between cannabis use, obesity and insulin resistance. *Methods*. We analysed data on 786 Inuit adults from the Nunavik Inuit Health Survey (2004). Information on cannabis use was obtained from a self-completed, confidential questionnaire. Fasting blood glucose and insulin, and homeostasis model assessment of insulin resistance (HOMA-IR) served as surrogate markers of insulin resistance. Analysis of covariance and multivariate logistic regression ascertained relationships between cannabis use and outcomes. *Results*. Cannabis use was highly prevalent in the study population (57.4%) and was statistically associated with lower body mass index (BMI) (p<0.001), lower percentage fat mass (p<0.001), lower fasting insulin (p=0.04) and HOMA-IR (p=0.01), after adjusting for numerous confounding variables. Further adjustment for BMI rendered fasting insulin and HOMA-IR differences statistically non-significant between past year cannabis users and non-users. Mediation analysis showed that the effect of cannabis use on insulin resistance was indirect, through BMI. In multivariate analysis, past year cannabis use was associated with 0.56 lower likelihood of obesity (95% CI 0.37–0.84). *Conclusions*. Cannabis use was associated with lower BMI, and such an association did not occur through the glucose metabolic process or related inflammatory markers. The association between cannabis use and insulin resistance was mediated through its influence on weight.

## The commercialization of country food and food security: The case of Greenland and what Nunavut can learn

### Joanna Petrasek MacDonald^1^, James Ford^1^, Catherine Huet^1^, MacRury Allison^2^ and Sara Statham^2^
^1^McGill University, Montreal, Canada, joannamacdonald08@gmail.com; ^2^Government of Nunavut, Iqaluit, Canada

#### 

Food security exists “when all people, at all times, have physical, social and economic access to sufficient, safe and nutritious food to meet their dietary needs and food preferences for an active and healthy life” (1). Access to adequate food has been identified as a major challenge in the Canadian Arctic, particularly for Inuit communities, where levels of food insecurity are consistently higher compared to Southern Canada (2). In Greenland, preliminary studies indicate relatively secure food systems in communities of comparable size to those in Nunavut, albeit with emerging stresses in-light of climatic and socio-economic change. Qualitative work has pointed to the potential of country food markets in providing relatively affordable sources of traditional food in communities (3). Herein, open air traditional food markets (kalaaliiaraq) are common in Greenland in communities of diverse sizes, providing both a source of economic returns for hunters and secure access to food for those in waged employment or who do not have access to networks through which food is shared. In-light of the relative security of food systems in Greenland, the project presented here investigates the role of commercial country food markets in enhancing food security within Greenland, and explores whether or not it is feasible to develop and promote similar markets in a Nunavut context. The project used a systematic literature review alongside semi-structured, in-depth interviews with decision makers, civil society organizations, the Kalaallit Nunaani Aalisartut Piniartullu Katuffiat (Organization of Hunters and Fishermen in Greenland), researchers and representatives of Inuit organizations (e.g. ICC) in Nuuk, Copenhagen and Iqaluit. The operation of commercial country food markets in Greenland, their impact on food security and transferable lessons from the Greenland experience will be discussed.

**References**

1. FAO. Declaration of the World Summit on Food Security. Proceedings of World Summit on Food Security, 2009; Rome, Italy; 2009.

2. Huet C, Rosol R, Egeland GM. The prevalence of food insecurity is high and the diet quality poor in Inuit communities. J Nutr. 2012;142(3):541–7.

3. Ford JD, Goldhar C. Climate change vulnerability and adaptation in resource dependent communities: a case study from West Greenland. Clim Res. 2012;54(2):181–96.

## Body mass index of First Nations children and youth on first entering Canadian Prairie residential schools – 1919 to 1953

### Paul Hackett, Sylvia Abonyi and Roland Dyck
University of Saskatchewan, Saskatoon, Canada, sylvia.abonyi@usask.ca

#### 

Historical research documenting nutrition experiments performed on First Nations (FN) residential school children in Canada from the 1940s indicates that students experienced hunger and malnutrition and suffered from overall poor health (1). Using cross-sectional residential school entrance examination data collected between 1919–1953, we report here on the BMIs of 1,767 Canadian Prairie FN children. This is significant because it reflects conditions in their home communities rather than in the schools. Examinations captured height and weight data as well as general qualitative observations on the health of the child. Following Cole et al (2, 3) age-specific BMIs were calculated and categorized as underweight, normal weight and overweight/obese by age, sex, time period and residential school site. Height and weight quartiles were compared with a 1953 Canadian survey and BMIs with current WHO growth charts for Canadian children. On admission to residential school, FN children were more likely to have normal BMIs than Canadian children today, and to have lower rates of overweight/obesity and higher rates of underweight. FN children tended to be slightly taller than non-FN Canadian children from the 1953 survey, but shorter than Canadian children today. Most weights for older FN children were within the 25th and 75th percentiles of non-FN Canadian children from the 1953 survey. A general trend for diminishing levels of underweight and increasing levels of overweight/obesity over time was observed. Highest rates of underweight occurred before the depression. Those attending residential schools in the southern prairies were more likely to be underweight. These findings are consistent with reports of malnutrition in some communities during the study period. Overall, however, results suggest that many FN children were not leaving their home communities in a malnourished state, unlike the poor health reported for children in attendance at the schools.

**References**

1. Mosby I. Administering colonial science: nutrition research and human biomedical experimentation in Aboriginal communities and residential schools, 1942–1952. Histoire Sociale/Soc Hist. 2013;46(91)145–72.

2. Cole TJ, Bellizzi MC, Flegal KM, Dietz WH. Establishing a standard definition for child overweight and obesity worldwide: international survey. BMJ. 2000;320:11240–3.

3. Cole TJ, Flegal KM, Nicholls D, Jackson AA. Body mass index cut offs to define thinness in children and adolescents: international survey. BMJ. 2007;335:194–201.

## Indigenous Health V: “Health systems”

### Geographic and social inequalities in blood pressure among Inuit in Greenland: a multilevel study

#### Mylene Riva^1^, Christina V.L. Larsen^2,3^ and Peter Bjerregaard^2,3^
^1^Centre de recherche du CHU de Quebec, Universite Laval, Quebec City, Canada, mylene.riva@crchuq.ulaval.ca; ^2^National Institute of Public Health, University of Southern Denmark, Odense, Denmark; ^3^Greenland Center for Health Research, University of Greenland, Nuuk, Greenland and Denmark

##### 

*Background*. The distribution of the social determinants of health is multilevel. This requires considering both the characteristics of people, including socio-economic characteristics in addition to behavioural risk factors, and the conditions of communities in the explanation of health variations. With the goal of documenting the social determinants of cardiovascular health among Inuit, this multilevel study examines the influence of community-level socio-economic conditions and individuals’ socio-economic position on blood pressure (BP) in Greenland. *Methods*. Data on 3,108 Inuit aged 18 years and older are from the Inuit Health in Transition – Greenland Survey (2005–2010). Systolic and diastolic BP were measured using an automatic measuring device. Respondents lived in 22 communities categorized by settlement type (towns vs. villages) and tertiles of material deprivation. Individual socio-economic position was defined by household wealth. Data were analysed using multilevel models, further adjusted for selected sociodemographic characteristics, clinical and behavioural risk factors. *Results*. In fully-adjusted models, BP of people living in communities categorized in the middle tertile of deprivation was significantly higher compared to BP of people living in communities in the most deprived tertile. BP did not vary by settlement type. With regards to individuals’ socio-economic position, BP decreased with increasing household wealth. Sex-stratified analyses further demonstrated the salience of community socio-economic conditions in influencing BP among women. *Conclusion*. Findings indicate geographical and social variation in BP in Greenland. This suggests that public health and social policies, programs and interventions targeting communities and living conditions might yield population-wide health benefits.

## Health and living conditions in northern Norway, the SAMINOR survey

### Ann Ragnhild Broderstad and Marita Melhus
Centre for Sami Health Research, University of Tromso – The Arctic University of Norway, Tromso, Norway, ann.ragnhild.broderstad@uit.no

#### 

*Background*. The first SAMINOR study, was conducted in 2003/2004 by Centre for Sami Health Research, in collaboration with the Norwegian Institute of Public Health. This was due to the insufficient information about the health and living conditions among the Sami in Norway. The data collection was carried out within a cross-sectional epidemiological design where all inhabitants in 24 selected municipalities were invited. In total, 16,968 persons participated. In 2012, the Centre initiated a follow up survey, SAMINOR 2, approximately 8 years after the first survey was completed. The data collection was done in two parts. Part 1, a questionnaire study in the same areas where SAMINOR1 was carried out, was conducted in 2012. The clinical part (part 2) was done in the period of September 2012–June 2014. *Objectives*. The main objectives to do these comprehensive surveys are to assess associations between lifestyle factors and risk factors for disease in relation to the different ethnic groups in the north. *Some results*. SAMINOR 1 and 2 give a unique knowledge about the health situation in the Sami population, but also provide health information about the non-Sami population in the same areas. We will present the study design for SAMINOR 2 and some preliminary results from the clinical analyses with regard to obesity, metabolic syndrome and type 2 diabetes mellitus. In addition, some other health outcomes will be highlighted in relation to ethnicity, gender and age. In general, this survey demonstrates a high prevalence of overweight and obesity in this population. Obesity and central obesity was most pronounced in Sami women. Preliminary results indicate a high prevalence of T2DM among the SAMINOR 2 population. *Conclusions*. The results from our analyses clearly demonstrate high prevalence of overweight and obesity in this population. Further analyses of living conditions and diseases will be done in the future.

## Bridging dichotomies in circumpolar health research: Findings from a systematic realist review

### Jennifer Jones^1^, Ashlee Cunsolo Willox^2^ and Sherilee Harper^1^
^1^University of Guelph, Guelph, Canada, jjones14@uoguelph.ca; ^2^Cape Breton University, Sydney, Canada

#### 

Indigenous populations in the circumpolar north continue to experience social and health inequalities that challenge policy makers, community leaders and health workers to rely on available research. Yet, the current health disparities and the heterogeneity of populations in the North requires an examination of and discussions around the tensions between large-scale population health-based research that aim to produce replicable data, and community-situated and often-small sample size approaches. If the aim of those working in research, health care, policy or programming is to support health and well-being in circumpolar regions, it is imperative to consider how findings using a multiplicity of approaches can work together. This issue is particularly germane given that communities have charged traditional epidemiological approaches of not being able to respond to complexities and nuanced issues that inform health and well-being. However, findings from both large empirical approaches and smaller community-driven qualitative research can support Northern communities in addressing systematic health disparities. Building from a strengths-based approach, and recognizing that health research in the circumpolar north is committed to responding to community level concerns, this presentation communicates findings from a systematic realistic review. Results are shared in effort to stimulate conversation to bridge perceived dichotomies of quantitative/qualitative, Western/indigenous and empirical-/community-driven research approaches as well as underlying assumptions that frame health research. Findings from this review seek to offer ground where researchers, communities, health professionals and decision makers using these multiple approaches can communicate findings resulting in customized, locally-appropriate responses to health and wellness issues in the North.

## From theory to practice: indigenous research ethics in action

### Julie Bull
NunatuKavut Community Council, Toronto, Canada, julierbull@gmail.com

#### 

As an indigenous researcher, I have spent the last decade working in partnership with my home community of NunatuKavut, Canada. This subarctic, geographically dispersed “community” is well positioned to be an example of how to meaningfully engage and conduct research involving indigenous people. In 2010, in partnership with Memorial University of Newfoundland, NunatuKavut revised its research ethics process and have implemented a rigorous community-based review system. Part of this oversight is also to have more access and control of the research being done with NunatuKavut lands or people. To address this need, we developed a research database that includes information of the past and current projects that are being undertaken in that region. This presentation will focus the best practices in community-driven research by emphasizing the role that indigenous communities themselves have in the research process and in the review of research in their communities. By drawing on this decade long partnership with NunatuKavut, I will highlight the importance of community-driven research and give examples of how such research actually works in practice.

## Oral health-related quality of life among an Alaska Native/American Indian urban outpatient pediatric population

### Vanessa Hiratsuka
Southcentral Foundation, Anchorage, AK, USA, vhiratsuka@scf.cc

#### 

*Introduction*. The early childhood caries (ECC) prevalence rate among American Indian/Alaska Native (AI/AN) children is five times the rate in the U.S. population. ECC impacts early life including a child’s ability to eat and speak, learn, have positive self-esteem and have an overall positive quality of life. Neither oral hygiene, oral health beliefs, nor oral health-related quality of life (OHRQL) have been well described among AI/AN people yet are necessary to understand in the shaping of oral health interventions for AI/AN ECC prevention. *Methods*. A cross-sectional survey which included the Oral Hygiene Scale, Oral Health Belief Questionnaire, and Early Childhood Oral Health Impact Scale (ECOHIS) was administered to parents of AI/AN children aged 0–6 years attending outpatient pediatric primary care appointments in an urban Alaskan health care setting. A total of 100 self-report surveys were collected. Bivariate analyses using Pearson chi-square were conducted comparing each behaviour, belief and OHRQL variable to each demographic variable to assess confounders of caregiver age, income, gender, education and race. *Results*. Univariate analysis of the ECOHIS found 49% of children had experienced pain in the teeth, mouth or jaws. AI/AN children with ECC were reported to have a statistically significantly higher prevalence of pain (p=0.001), difficulty drinking (p<0.001), difficulty eating (p=0.002), difficulty pronouncing words (p=0.007), missing school or daycare (p=0.005), trouble sleeping (p=0.004), feeling irritable (p<0.001), avoiding smiling (p=0.006) and avoiding talking due to ECC (p=0.064). Parents of a child with ECC reported family members with upset feelings (p<0.001), guilty feelings (p<0.001), having to take time off work (p<0.001) and having a financial impact due to ECC (p=0.005). *Conclusions*. These findings indicate that quality of life of AI/AN children and their families is diminished due to ECC. AI/AN children with ECC had higher mean ECOHIS scores.

## A research methodology for informing a dialogue on health systems improvements in circumpolar regions using indigenous knowledge

### Susan Chatwood
ICHR, Yellowknife, Canada, susan.chatwood@ichr.ca

#### 

The health services’ challenges experienced by indigenous peoples in circumpolar nations are complex and engage a broad and inter-related range of sectors, including health, environment, education, justice and traditional institutions outside government departments. Declarations such as the United Nations Declaration on the Rights of Indigenous Peoples have recognized the rights of indigenous peoples to maintain access to their traditional medicines and health practices, including the conservation of vital medicinal plants, animals and minerals. The UN declaration also calls for the right to access, without discrimination, all social and health services. Circumpolar nations have agreed to the terms of these declarations, but have recognized that these rights will require a stewardship model informed not only by currently valued evidence but also by an appreciation of diverse value systems and different sources of evidence. There has been a call to expand the research agenda across sectors responsible for health and well-being and recognize academic and indigenous knowledge bases. Such a research approach requires systematic understandings and comparisons in order to gain insight into health-systems strengths and adaptations applicable in the circumpolar setting. This paper describes current understandings of methodological approaches and presents a novel approach of a mixed methods methodology to meet this challenge.

## Disparities in infectious disease hospitalizations among Alaska Native persons compared to non-Alaska Native persons in Alaska, USA

### Prabhu Gounder^1^, Robert Holman^1^, Sara Seeman^1^, Jason Mehal^1^, Alice Rarig^2^, Mary McEwen^2^, Claudia Steiner^3^, Michael L. Bartholomew^4^ and Thomas W. Hennessy^1^
^1^Centers for Disease Control and Prevention, Atlanta, GA, USA, iym4@cdc.gov; ^2^Division of Public Health, Alaska Department of Health and Social Services, Juneau, AK, USA; ^3^ Health care Cost and Utilization Project, Center for Delivery, Organization and Markets, Agency for Health care and Research and Quality, Rockville, MD, USA; ^4^Indian Health Service, Rockville, MD, USA

#### 

*Background*. For the first time, a comprehensive data set exists with both Alaska Native (AN) and non-AN persons hospitalized in Alaska, USA. This study aims to estimate of the rate of infectious disease (ID) hospitalizations among AN and non-AN people living in Alaska, and provide comparisons between the two groups. *Methods*. Hospital discharge data for IDs from Alaska’s Indian Health Service and the Alaska State Inpatient Database (includes all but one non-tribally-operated acute care community hospital) were combined and analysed for 2010?2011. The ID hospitalizations were defined as records with a first-listed *International Classification of Diseases, 9th Revision, Clinical Modification code* for IDs. Age-adjusted and age-specific hospitalization rates/100,000 persons were calculated using corresponding annual population data from the Alaska Department of Labor and Workforce Development; rates were directly adjusted using the 2000 U.S. population as the standard. Rates were compared using the z-test and Poisson regression. *Results*. Among AN people, 19% (6,531) of hospitalizations were for ID versus 12% (7,752) among non-AN people in Alaska. The age-adjusted annual ID hospitalization rate in AN persons (3,004) was higher than in non-AN persons (898; rate ratio [RR]=3.3, p<0.05). The ID hospitalization rate disparity between AN and non-AN people was greatest for children aged <1 year (RR=5.4, p<0.05) and 1?4 years (RR=4.2, p<0.05). The lower respiratory tract infections (LRTI) ID category accounted for 38% of ID hospitalizations in AN persons; the age-adjusted rate was higher for AN compared to non-AN persons (RR=4.6, p<0.05). *Conclusions*. The AN people experience a disproportionate burden of ID hospitalizations compared with non-AN people in Alaska. The disparity in the rate for ID hospitalizations among AN persons compared with non-AN persons was greatest for children aged <5 years. LRTI hospitalizations contributed the greatest to the burden of ID hospitalizations.

## Population Health I

### Assessment of oral health in elderly in circumpolar territory of Russia

#### Karina Kunavina^1,2^, Andrey Soloviev^1^, Alexander Opravin^1^ and Arja Rautio^2^
^1^Northern State Medical University, Arkhangelsk, Russia, kunavina.karina@mail.ru; ^2^University of Oulu, Oulu, Finland

##### 

Modern world social and health tendencies largely dictate necessity of development of ageing population problem. Ageing is a natural phenomenon and an inevitable process but when number of old-aged people exceeds the number of working-age population, it needs serious modifications in medical care, economics, social support and so forth. Sociodemographic situation in the Arkhangelsk region is characterized by increase in the number of retirement age people. Severe climate conditions affect human health and well-being. In this regard, organs and tissues of the mouth are not an exception and elderly have serious problems with their oral health too. The aim of our study was to evaluate changes in the oral health of elderly and also influence of North conditions and possible unhealthy habits on the health of oral cavity. Research focuses on elderly people older than 60 years, who live in nursing home (Arkhangelsk, Russia). Examination includes objective, standardized dental tests and indices recommended by WHO (2013) (2). During the study, determination of dental, periodontal, salivary, lingual and immune status of oral health in elderly patients and comparison these indicators with norm were performed. We observed 100% prevalence of caries and Decayed, Missed, Filled Teeth (DMFT)=24 with predominance of missing teeth, frequent attrition, low level of hygiene, and poor condition of dentures. There was high prevalence of periodontal diseases, oral mucosa lesions, low salivary flow rate (0.08 ml/min) and pH (6.0), high saliva viscosity and unfavorable changes of the oral immunity indicators (sIgA, IgG, TNF-α, IL-8, cortisol). This study will allow formulating recommendations for patients, social workers and superior management bodies for improving oral well-being of this group of people. Data received during this study can be beneficial for other countries of Barents region in two common positions: dental features of ageing population and influence of North living conditions on oral health.

**References**

1. Lantto A, Lundqvist R, Wårdh I. Oral rehabilitation in older people and functionally impaired individuals. 22nd Nordic Congress of Gerontology, Gothenburg. 2014 May 25–28. Abstracts.

2. World Health Organization. Oral health surveys: basic methods. 5th ed. [cited 2015 Jan 4]. Available from: http://apps.who.int/iris/bitstream/10665/97035/1/9789241548649_eng.pdf?ua=1

3. Somsak K, Kaewplung O. The effects of the number of natural teeth and posterior occluding pairs on the oral health-related quality of life in elderly dental patients. Gerodontology. 2016;33:52–60.

## Is coronary artery disease more severe in the North compared to the South of Tyumen region?

### Vadim Kuznetsov, Grigoriy Kolunin, Dmitriy Krinochkin, Elena Gorbatenko, Georgiy Pushkarev and Luiza Marinskikh
Tyumen Cardiology Center, Tyumen city, Russia, kuznets@cardio.tmn.ru

#### 

*Aim*. The aim of this study was to test the hypothesis about more severe coronary artery disease in patients living in the circumpolar areas compared to the south areas of Tyumen region of Western Siberia. *Methods*. A total of 9,869 symptomatic consecutive subjects underwent clinical investigation, echocardiography and coronary angiography. 6,350 patients with coronary angiographic atherosclerosis (>50% of lumen) or with a history of myocardial infarction were selected: 3,715 from the south and 2,635 from the circumpolar north areas of Tyumen region. Groups were matched for age and gender. *Results*. Higher rates of smoking (59.9 vs. 51.2%, *p*<0.001), obesity (63.3 vs. 57.0%, *p*<0.001), diabetes mellitus (15.7 vs. 11.6%, *p*<0.001), alcohol drinking (29.3 vs. 22.1%, *p*<0.001), history of myocardial infarction (73.0 vs. 66.4%, *p*<0.001), thyroid disorders (14.6 vs. 8.4%, *p*<0.001) were found in the north compared to the south patients as well as higher level of blood glucose (5.75±1.76 vs. 5.58±1.59 mmol/L, *p*<0.001). Most of patients in the north and the south had arterial hypertension (81.7 and 81.0%, *p*=0.67). No difference in heart failure severity was detected between the north and the south patients. The prevalence of angina pectoris was higher in the south (94.6 vs. 86.7%, *p*<0.001) as well as the rate of III–IV Canadian classes of angina (52.0 vs. 48.3, *p*=0.01). Echocardiographic measurements and angiographic characteristics were not significantly different between the groups. *Conclusion*. We revealed more severe risk-factor profile and higher prevalence of prior myocardial infarction in the north coronary patients, however more severe classes of angina pectoris occurred in the south patients of Tyumen region.

## Collaboration in action: the Yukon CANRISK Diabetes Screening Project

### Brendan Hanley^1^, Ying Jiang^2^ and Gail Peekeekoot^3^
^1^Chief Medical Officer of Health, Whitehorse, Canada, brendan.hanley@gov.yk.ca; ^2^Public Health Agency of Canada, Ottawa, Canada; ^3^Department of Health and Social Services, Government of Yukon, Whitehorse, Canada

#### 

*Introduction*. The Public Health Agency of Canada (PHAC) CANRISK initiative seeks to promote the CANRISK diabetes screening tool in order to identify people at high risk of diabetes, as well as to increase awareness among Canadians about pre-diabetes, Type 2 diabetes and its risk factors. A collaborative project between PHAC, Government of Yukon, the Office of the Chief Medical Officer of Health (CMOH), the Council of Yukon First Nations (CYFN) and Kwanlin Dun First Nation was set up as part of an effort to validate the CANRISK screening tool for young Canadian First Nation adults. *Methods*. A total of 303 Yukon adults aged 20–39 years and without known diabetes were recruited into the study between January and June 2014. Eighty percent of the participants self-identified as First Nations. Participants were measured for waist circumference and BMI, and were asked to complete a questionnaire on demographic information and risk factors for diabetes. Blood samples were taken for fasting plasma glucose (FPG), oral glucose tolerance test (OGTT) and glycated hemoglobin test (HbA1C). Risk factor assessments were analysed against blood results. *Results*. Only 2 (1%) of the participants were found to have undiagnosed diabetes, while 30 (10%) were found to have pre-diabetes. Significant numbers of participants had high waist circumference and/or BMI’s in the obese range. Follow-up to discuss results and lifestyle considerations was offered to all participants. *Conclusion*. The Yukon CANRISK project demonstrated a successful inter-governmental collaboration and helped to raise awareness of diabetes amongst young Yukon First Nations adults. Pre-diabetes was found to be a significant issue for this population. Relatively high BMI and waist circumference rates support the need to connect people to more active lifestyles and healthy eating patterns, while recognizing the complex lives that young First Nations adults often lead in a post-residential school context. Both individual and community-based follow-up projects are being planned.

## Hip geometry and cortical index in Greenland hip fracture patients and the possible influence on hip fracture occurrence

### Inuuteq Fleischer^1^, Mogens Berg Laursern^2^ and Stig Andersen^1^
^1^Arctic Health Reserach Centre, Deptartment of Clinical Medicine, Aalborg University Hospital, Aalborg, Denmark, inuuflei@rm.dk; ^2^Department of Ortopedics, Aalborg University Hospital, Aalborg, Denmark

#### 

*Background*. Osteoporosis is a debilitating disease associated with fractures, pain, disability, premature death and marked costs on society. Osteoporotic fractures occur at forearm, spine and hip with predominance in the 6th, 7th and 8th decades, respectively. Increasing life span among Arctic inhabitants calls for attention to the occurrence of fragility fractures. Inuit is a distinct ethnic group that differs in body build and BMI. Thus, the research questions are: Do differences in body build carried through to bones? If so, does this influence the risk of osteoporotic fractures? *Methods*. We retrieved radiographs of 200 patients admitted to the Orthopaedic Department at Queen Ingrids Hospital, Nuuk, Greenland for a hip fracture. We evaluated collodiaphysial angle, femur neck length, outer and inner diameter of femur, cortical thickness and cortical thickness index at 2 and 5 cm below the lesser trochanter. *Results*. We here include results from the first 37 evaluations. Median height/weight was 165 cm/68 kg in men and 154 cm/56 kg in women. Mean age was 67/74 years in men/women. Collodiaphysial angle was 137.5°/136.1° and femur neck length was 38.5/34.9 mm in men/women (p=0.012). At 5 cm below the lesser trochanter outer/inner femur diameter was 29/15 mm in men and 25/15 mm in women with cortical thickness of 6.9 and 5.3 mm in men and women, respectively (gender difference, p<0.001). The cortical thickness index in men/women was 0.49/0.36 (p=0.005) at 5 cm below the lesser trochanter. Cortical thickness index decreased with age (r −0.09, p0.05). *Conclusion*. Greenland patients were younger and smaller compared to those in Europe. Hip geometry in Greenland was in keeping with reference values for Caucasians. Gender differences were marked. Cortical thickness index was comparable to Scandinavians and decreased with age. This heralds increasing hip fracture frequency in the ageing Arctic populations.

## The structure of congenital heart defects (CHD) among newborn in the Republic of Sakha (Yakutia)

### Tuyara Nelunova^1^, Vyacheslav Chasnyk^1^ and Tatyana Burtseva^2^
^1^Saint-Petersburg State Pediatric Medical University, St. Petersburg, Russia, nelunova-ti@mail.ru; ^2^Yakut Scientific Center, Yakutsk, Russia

#### 

*Aim of research*. To study the structure of congenital heart defects (CHDs) among newborn in the Republic of Sakha (Yakutia) on ethnic criteria. *Data and methods of research*. The research was conducted on the basis of the Perinatal Center State Budgetary Institution of the Republic of Sakha (Yakutia) Republican Hospital N^o^1 among live-born newborns for the period from 2011 to 2015. CHDs were registered according to the nomenclature headings Q20–Q28 (ICD10). Nosological diagnoses CHDs were confirmed with data of echocardiography and Doppler sonography of heart and vessels, electrocardiograms, roentgenograms, computer tomograms in angio regime, angiographic researches. The indicator of frequency was counted in an amount of 1.000 live-born children. Proportion of the Yakuts in ethnic composition of population was 49.9%, the Russians 37.8%, the Ukranians 2.2%, the Evenks 2.2%, the Evens 1.6%, the Tatars 0.9% (according to the data of All-Russia census in 2010). *Results and discussion*. According to our data, there were registered 899 cases of CHDs among live-born newborns for the period from 2011 to 2013. In ethnic composition, the proportion of the Yakuts was 72.08% (648 newborn children), the Russians 16.70% (150), the Evenks 4.89% (44), the Evens 1.33% (12), the Yukagirs 0.11% (1), the Dolgans 0.11% (1), the Chukchis 0.11% (1), others 4.67% (42) among all the cases of CHDs (899). The indigenous minorities of the North amounted to 6.56% (59 newborns) in total among all the diagnosed cases of CHDs. *Conclusions*. 1) The absolute prevalence of the Yakut children with CHDs was elicited: 72.08% (648 cases) of all the diagnosed cases of CHDs. 2) The Russians are on the second place in frequency – 16.70% (150 cases). Indigenous minorities of the North amounted to 6.56% (59 cases) which made the third place in frequency. 3) The given data correspond to proportions in ethnic composition according to statistics (on the results of All-Russia census in 2010).

## Controversies in newborn screening and alternative approaches to prevention of hypoglycemia for the common Arctic variant (P479L) in CPT1A

### Laura Arbour^1^, Cheryl Greenberg^2^, Graham Sinclair^1^, Sharon Edmunds-Potvin^3^, Charmaine Enns^4^, Hilary Vallance^1^, Sorcha Collins^1^, Bob Thompson^5^ and Jessica Hartley^2^
^1^UBC, Vancouver, Canada, larbour@uvic.ca; ^2^University of Manitoba, Winnipeg, Canada; ^3^Nunavut Tunngavik Incorporated, Iqaluit, Canada; ^4^Island Health, Victoria, Canada; ^5^Cadham Provincial Laboratory, Winnipeg, Canada

#### 

CPT1a is an enzyme required in the use of fats for energy during fasting. Severe CPT1a deficiency, with little or no enzyme activity leads to hypoglycemia, seizures and death if not treated. In contrast, the P479L variant of CPT1A has reduced but not severely deficient activity but may still lead to hypoglycaemia. The P479L variant is highly prevalent in Inuit, Inuvialuit, Alaska Native and Vancouver Island First Nations populations but not seen in non-indigenous populations. There is evidence the variant conferred a strong historical advantage during early migration, allowing survival in harsh environments on a high fat, low carbohydrate diet. However, in all areas with a high prevalence of P479L homozygosity, there is also an associated increase in infant mortality (IM). Significant association of the P479L allele and IM caused by Sudden death in infancy (SUDI), Sudden infant dealth syndrome (SIDS) and/or infectious disease has been established in all populations studied, but the underlying mechanism is unknown, and causation has not been established. There is substantial controversy as to whether newborn screening should be carried out, leading to significant variability in practice. Given the high prevalence of the variant in First Nations of Vancouver Island, but insufficient evidence to support newborn screening, Medical Guidelines to Prevent Hypoglycemia in First Nations Infants have been developed to raise the awareness of the possible deleterious effects. Similarly, Manitoba medical specialists covering the Kivalliq region of Nunavut (>70% homozygosity) have also focused on increasing awareness through dissemination of information and education of local health professionals. All Kivalliq newborns born in Winnipeg are screened for hypoglycemia and select genotyping is performed. Although more research is urgently needed to address or refute the increasing evidence that the variant has a negative impact on infant and child health, these interim approaches to care aim to inform and mitigate potential risk.

**References**

1. Greenberg CR, Dilling LA, Thompson GR, Seargeant LE, Haworth JC, Phillips S, et al. The paradox of the carnitine palmitoyltransferase type Ia P479L variant in Canadian Aboriginal populations. Mol Genet Metab. 2009;96:201–7.

2. Collins SA, Sinclair G, McIntosh S, Bamforth F, Thompson R, Sobol I, et al. Carnitine palmitoyltransferase 1A (CPT1A) P479L prevalence in live newborns in Yukon, Northwest Territories, and Nunavut. Mol Genet Metab. 2010;101:200–4.

3. Sinclair GB, Collins S, Popescu O, McFadden D, Arbour L, Vallance HD. Carnitine palmitoyltransferase I and sudden unexpected infant death in British Columbia First Nations. Pediatrics. 2012;130:1162–9.

## The Yukon CANRISK project: lessons learned in collaborative research

### Brendan Hanley^1^, Ying Jiang^2^ and Gail Peekeekoot^3^
^1^Chief Medical Officer of Health, Whitehorse, Canada, brendan.hanley@gov.yk.ca; ^2^Public Health Agency of Canada, Ottawa, Canada; ^3^Department of Health and Social Services, Government of Yukon, Whitehorse, Canada

#### 

*Introduction*. Collaboration between Public Health Agency of Canada (PHAC), Government of Yukon, the Office of the Chief Medical Officer of Health (CMOH), the Council of Yukon First Nations (CYFN), Kwanlin Dun First Nation and several rural Yukon communities supported a research project to validate the CANRISK screening tool for young Canadian First Nation adults (20–39 years). Six other Yukon First Nations played supported and facilitated the research. In follow-up to the research, a “lessons learned” report was compiled by the study author. The lessons learned are particularly relevant for a multi-stakeholder collaboration which saw a project completed within a relatively short time, in a jurisdiction with little formal research infrastructure. Discussion of research lessons learned may be of value to other northern jurisdictions considering research projects. *Methods*. In a separate report to the research results, a “lessons learned” document was prepared by the project coordinator. The report in draft was discussed at a post-project wrap-up meeting where considerable discussion of the project’s successes and shortcomings were addressed. The “lessons learned” document was finalized and distributed after this meeting. *Results*. Several recommendations to strengthen future collaborative research projects were made and will be discussed. Specific areas of interest include:

- Administrative requirements: financial coordination, technological and communications infrastructure and logistical support.
- Recruitment: finding the best way to reach a young adult, primarily First Nation audience.
- Agreements: establishing written contribution agreements between collaborating bodies.
- Consent: mechanisms to ensure adequate informed consent for participants.
- Follow-up: mechanisms to ensure adequate follow-up is provided for all participants.
- Next steps: the lessons learned will be applied to future research and collaborative projects in Yukon.

##### Population Health II

###### On the anti-inflammatory effect of vitamin D in the traditional Inuit diet in Greenland

####### Louise Schæbel^1,2^, Henrik Vestergaard^3^, Peter Laurberg^4^, Eva Bonefeld-Jørgensen^1^ and Stig Andersen^2^
^1^Centre for Arctic Health, Department of Public Health, Aarhus University, Aarhu, Denmark, lohos@rn.dk; ^2^Arctic Health Research Centre, Department of Clinical Medicine, Aalborg University Hospital, Aalborg, Denmark; ^3^The Novo Nordisk Foundation Center for Basic Metabolic Research, Section of Metabolic Genetics, Faculty of Health and Medical Sciences, University of Copenhagen, Copenhagen, Denmark; ^4^Endocrine Research Unit, Department of Clinical Medicine, Aalborg University Hospital, Aalborg, Denmark

######## 

*Purpose*. The marine diet is an important source for vitamin D in Greenland and high levels of vitamin D is found in the traditional Greenlandic diet that consists mainly of fish and marine mammals. Vitamin D may dampen inflammation. Yet, the influence of vitamin D from the traditional Inuit diet on inflammation needs to be elucidated. *Methods*. Blood samples were drawn and interview-based food frequency questionnaires were conducted in a population-based survey in Nuuk and Ammassalik district in West and East Greenland, respectively. Vitamin D and the markers of inflammation YKL-40 and hsCRP were measured in serum. Participants were divided into three groups based on dietary habits: intake of mainly traditional Inuit diet versus mixed versus mainly imported food items. *Results*. The population consisted of 535 men and women aged 50–69 years. They were 434 Inuit and 101 non-Inuit. Vitamin D levels in serum varied with intake of traditional Inuit food items: Inuit diet/mixed diet/imported foods: 74.2/69.8/52.9 nM (p<0.001). Parallel differences were seen for hsCRP (1.6/1.4/1.3 mg/L; p=0.002) and for YKL-40 (130/95/61 micro g/L; p<0.001). YKL-40 level decreased with increasing vitamin D level for the participants living on traditional Inuit diet (p=0.014), this was however not seen in participants living on imported foods (p=0.87). There was no significant difference in hsCRP with increasing vitamin D for either of the diet groups. YKL-40 decreased with higher intake of vitamin D after adjusting for other factors known to influence inflammation (p<0.001). This was not seen for hsCRP. *Conclusion*. Vitamin D and markers of inflammation vary in parallel with the intake of traditional Inuit diet. This does not conform to a positive influence of vitamin D on the inflammatory process unless other factors are involved. This may be speculated to be the case.

##### Trends in vitamin D status in Greenland from 1987–2010 and the association with type 2 diabetes

###### Nina Odgaard Nielsen, Peter Bjerregaard and Marit Eika Jørgensen
University of Southern Denmark, Odense, Denmark, noni@niph.dk

####### 

*Background*. Low vitamin D status may be pronounced in Arctic populations due to limited sun exposure and decreasing intake of traditional food. Low vitamin D status has been associated with type 2 diabetes in other populations. *Objective*. To investigate serum 25(OH)D3 as a measure of vitamin D status among adult Inuit in Greenland and the trend from 1987 to 2005–2010 and the association with glucose homeostasis and type 2 diabetes. *Design*. A total of 2,877 randomly selected Inuit (≥18 years) from the Inuit Health in Transition study 2005–2010 were included. A subsample (n=330) donated a blood sample in 1987 allowing assessment of time trends in vitamin D status. Association between vitamin D status and glucose homeostasis and type 2 diabetes was assessed at a cross-sectional level. *Results*. The geometric mean serum 25(OH)D3 in 2005–2010 was lowest among the 18–29-year-old individuals (30.7 nmol/L; 95% CI: 29.7; 31.7) and increased with age. In all age-groups it decreased from 1987 to 2005–2010 (32–58%). Low 25(OH)D3 concentrations (<50 nmol/L) were present in 77% of the 18–29 year old and decreased with age. Contrary to the hypothesis, preliminary analyses show an increase in fasting plasma glucose (5.4–5.9 mmol/L, p<0.001) and 2-h plasma glucose (5.3–6.7 mmol/L, p<0.001) across quintiles of serum 25(OH)D3 (quintile 1 lowest; quintile 5 highest), whereas fasting insulin decreased from quintile 1 to 5 (47.8–40.1 pmol/L, p<0.001). Two-hour insulin was equal in quintiles. The prevalence of impaired fasting glucose, impaired glucose tolerance and type 2 diabetes increased across quintiles (p<0.001 for all three). *Conclusion*. We identified a remarkable decrease in vitamin D status from 1987 to 2005–2010 and a presently low vitamin D status among Inuit in Greenland. A change away from a traditional diet may well explain the observed decline. Our study can, however, not provide evidence for an association between low vitamin D status and type 2 diabetes in Greenland.

## Creatinine excretion is lower in Inuit than in non-Inuit and the influences on iodine nutrition classification

### Stig Andersen and Peter Laurberg
Aalborg University Hospital, Aalborg, Denmark, stiga@dadlnet.dk

#### 

*Background*. Human contamination and iodine excretion is commonly assessed by the analysis of urine. A 24-h urine sample is ideal but it is inconvenient, inaccurate and unreliable. Thus, spot urine sampling with creatinine to adjust for differences in void volume is widely used. Still, the importance of ethnicity and the timing of spot urine samples need to be settled. *Methods*. We collected 104 spot and 24-h urine samples from Greenland Inuit and non-Inuit. We measured creatinine using the Jaffe method, iodine using the Sandell-Kolthoff reaction and para-amino benzoic acid (PABA) by the high pressure liquid chromatography (HPLC) method for estimation of completeness of sampling. *Results*. Population-based recruitment was done from the capital city, a major town and a settlement (n=36/48/20). Participants were 78 Inuit and 26 non-Inuit aged 30 through 69 years. Inuit were smaller than non-Inuit (weight, 71 vs. 84 kg; p<0.001). Urinary creatinine excretion was lower in Inuit than in non-Inuit (men, 1,344/1,807 mg/24 h; women 894/1,259 mg/24 h; p=0.002, 0.02). Creatinine excretion was influenced by age (p<0.001), gender (p<0.001), weight (p<0.001) and ethnicity (p=0.047) while unaffected by Inuit diet. Data suggest a similar influence of age on creatinine excretion in Inuit and non-Inuit. Iodine excretion in Inuit/non-Inuit 24-h urine (median, 25–75 percentiles) was 153, 97–251 µg/102, 73–138 µg (p=0.026) when compensated for incomplete sampling (40% of samples). It increased with rising intake of traditional Inuit foods (p=0.005). Iodine was lower in morning spot urine than in 24-h urine samples (p<0.001). This difference associated with iodine intake levels (p<0.001) and led to misclassification of iodine excretion when iodine nutrition was above the recommended level. In conclusion, creatinine excretion was lower with Inuit ethnicity. This influences estimates of 24-h excretions from spot urine samples. The nutrition level was misclassified from morning spot urine samples with higher levels of iodine.

## Genetic signals of adaptation to climate in Siberian populations

### Vadim Stepanov, Vladimir Kharkov, Anton Markov, Ksenia Vagaitseva, Anastasia Cherednichenko and Ekaterina Trifonova
Institute for Medical Genetics, Tomsk, Russia, vadim.stepanov@medgenetics.ru

#### 

Adaptation to new environment during early human migrations from tropical to moderate and Arctic climate played the substantial role in shaping the genetic structure of modern human populations. We have investigated the distribution of genome-wide single nucleotide polymorphisms (SNPs), correlated with climatic and geographic parameters, in native Siberians comparing to worldwide human populations. Our data on genome-wide SNPs frequencies in five native Siberian populations (Buriat, Yakut, Tuva, Khants, Kets) were pooled with data on worldwide populations and analysed by means of positional search of association of allele frequencies with climatic and geographic parameters and search for signals of natural selection. Top signals were subjected to bioinformatic analysis of disease associations, biological processes, pathways and molecular functions of corresponding genes. About 1,000 SNPs were found very significantly correlated with key climatic parameters and absolute latitude. These SNPs are located in regions of about 450 genes. Major clinical phenotypes associated with these regions are metabolic traits, immune and infectious diseases, neuro-psychiatric disorders, behavioural traits, response to xenobiotics and chemo-dependency traits. Some top genomic regions were found under positive or balancing selection according to haplotype homozygozity (EHH, XP-EHH) and Tajima tests. Examples of regions subjected to selective pressure in Siberian natives include UDP-Glucuronosyltransferase-2B7 (UGT2B7, metabolism of xenobiotics), uncoupling proteins (UCP2-3, thermoregulation) and interferon lambda (IFNL4, modulation of immune response) genes. We suppose that genetic diversity in the substantial part of the human genome in native Siberian populations were shaped by adaptation to cold climate. The adaptive pattern of genetic diversity may contribute to common diseases, and may be explained by the hypothesis of decanalization of genotype-environment relationships under the pressure of natural selection.

## Seasonality of clinical symptoms among high-risk families for bipolar disorders in the Arctic

### Sami Pirkola^1^, Heidi Eriksen^1^, Tiina Paunio^2^, Tuula Kieseppä^2^, Timo Partonen^2^, Juha Veijola^3^, Erika Jääskeläinen^3^ and Eeva-Maija Mylläri-Figuerola^4^
^1^University of Tampere, Tampere, Finland, heidi.eriksen@utsjoki.fi; ^2^The National Institute of Health and Welfare, Helsinki, Finland; ^3^University of Oulu, Oulu, Finland; ^4^Hospital District of Lapland, Rovaniemi, Finland

#### 

*Background*. Bipolar disorder (BD) is characterized by periods of manic and depressive behaviour, often precipitated by stressful life-events, substance abuse and sleep-cycle disturbances. Regional variation as well as particularly high penetrance families in Finland exist. These are likely to relate both to genetic and specific environmental factors. Seasonality in bipolarity symptoms is common and circadian, and seasonal rhythms are often disturbed. We explored the clinical characteristics of subjects living in latitudes 68–70, with extreme annual variation in daylight. Three groups were studied: (1) 15 subjects with a bipolar spectrum disorder from known high-prevalence pedigrees, (2) 16 healthy family members and (3) 18 healthy non-related controls from the same geographical area. Possible seasonal fluctuation in mood, distress, sleep, social activity and alcohol consumption, were followed up at the four most demarcated photoperiodic time points of a year. Groups 1 and 2 represented the indigenous population on the northern latitudes, the Sámi people, who have settled the area for 8,000–10,000 years. *Data*. The diagnostic Structural Clinical Interview for DSM disorders (SCID) interview and the data from traumatic experiences (TSQ), lifetime manic behaviour (MDQ) and self-reported seasonality factors (SPAQ1 and SPAQ2) were collected in the baseline, and self-reported depressive symptoms (BDI), psychic distress (GHQ-12), sleep duration, alcohol consumption (g/wk) and vitamin D levels at the four time points. *Results*. The baseline: The affected had the highest MDQ, SPQ1 and TSQ sums. The follow up: There was a variation in measures both within and between the groups. In individual statistical significance testing, the affected scored higher than healthy relatives in winter and spring GHQ12 (4.00 vs. 1.25 and 3.78 vs. 1.08) and BDI (11.88 vs. 4.50 and 11.20 vs. 5.17), but not in summer and autumn. Summer vitamin D levels were the highest among the affected subjects. *Discussion*. The baseline differences validate the study groups, and support seasonal patterns in bipolarity. Seasonal variation in follow-up was observed in affective and distress symptoms although the sample is relatively small for statistical significances. Individual findings will be explored in further genetic analyses.

**References**

1. Craddock N, Sklar P. Genetics of bipolar disorder. Lancet. 2013;381(9878):1654–62.

2. Wang B, Chen D. Evidence for seasonal mania: a review. J Psychiatr Pract. 2013;19(4):301–8.

3. Akhter A, Fiedorowicz JG, Zhang T, Potash JB, Cavanaugh J, Solomon DA, et al. Seasonal variation of manic and depressive symptoms in bipolar disorder. Bipolar Disord. 2013;15(4):377–84.

## Environmental Contaminants III

### Project “The impacts of hazardous substances on human health and communities in the Barents Region” as an environment health policy tool in the region

#### Elena Golubeva and Natalia Kukarenko
Northern Arctic Federal University, Arkhangelsk, Russia, e.golubeva@narfu.ru

##### 

*Introduction*. The documents adopted by the Russian national legislation, the Arctic Council and WHO stated priority of preventive measures to stabilize the demographic processes, preserving and strengthening the health of the most vulnerable groups, including indigenous. To harmful factors specific to the Arctic regions, the intensity of exposure which can be reduced or offset by preventive methods include: climatic, anthropogenic, pathogenic (1). The people in the Arctic are exposed to contaminants through the food (2) and the surrounding environment, from both distant and local sources. Arkhangelsk region is industrialized regions, where the largest pulp and paper and wood processing industry, energy sector. Emissions from industrial facilities have a high content of sulfur dioxide (50.0%), and various kinds of dust (16.5%), carbon monoxide (10.6%), hydrocarbons (12.6%). Over the past 5 years in the cities of Arkhangelsk region, there is a tendency to an increase in the level of air pollution with nitrogen dioxide (3). *The aim of research*. To investigate the impact of contaminants on food and water safety, human health and communities in Russian part of the Barents Region. *Materials and methods*. The interview guide and subsequently a questionnaire form are used for the comparative and synthesis studies (Novodvinsk [Arkhangelsk region]), N=375, and interviews with qualitative, including interviews with ordinary people, experts and policy makers, analyses of newspapers and other media, as well as policy document studies were done. *Results and discussion*. We supported the development of tools for local and regional risk management for Arctic communities, assessing relationships between pollution control policies, health, culture, social condition and environment, as well as strategies for risk communication, based on how expert and local traditional knowledge is generated and perceived.

**References**

1. Chashchin V. et al. Characteristics of the main risk factors for the population’s health living in areas of active environmental management in the Arctic. Hum Ecol. 2014;1:3–12.

2. Donaldson et al. Sci. Total Environ. 2010; 408(3):5165–234.

3. Baidakova E. et al. The asthma morbidity of population in the Arkhangelsk Region. Hum Ecol. 2011;12:8–13.

## Levels and trends of contaminants in Arctic populations

### Jennifer Gibson^1^, Bryan Adlard^1^, Kristín Olafsdottir^2^ and Torkjel Sandanger^3^
^1^Health Canada, Ottawa, Canada, Jennifer.gibson@hc-sc.gc.ca; ^2^University of Iceland, Reykjavík, Iceland; ^3^University of Tromso – The Arctic University of Norway, Tromso, Norway

#### 

The Arctic Monitoring and Assessment Programme (AMAP) is one of six working groups (WG) established under the Arctic Council. AMAP is tasked with monitoring the levels of contaminants present in the Arctic environment and people as well as assessing their effects on a continuous basis, and reporting these results regularly. This presentation provides an overview of the human biomonitoring data reported in the 2015 Human Health Assessment Report from all eight Arctic countries. Levels of contaminants are declining in the monitored Arctic populations, but not consistently across the Arctic. Certain populations are experiencing more rapid declines than others, and certain populations have concentrations that are remaining stable or are still increasing. Most Arctic populations described in this chapter continue to experience elevated levels of these contaminants compared to other populations monitored worldwide, for example, mercury, where 7–85% of Inuit women 18–39 years of age in Arctic Canada and Greenland exceed the Canadian provisional blood guidance value of 8 ug/L established for children and women of childbearing age. There are certain contaminants, like perfluorinated compounds (PFCs) and polybrominated diphenyl ethers (PBDEs) which are still increasing in Arctic populations, and require more investigation to find the predominant and important sources of exposure. Most of these data have been collected over the last 20 years and are from all eight circumpolar countries. Coordinated, international biomonitoring must continue in the future to determine if levels of these contaminants, and others, are changing in Arctic populations. This is especially important for those populations still showing elevated levels.

## Greenlandic pregnant women: diet and serum persistent organic pollutants

### Eva C. Bonefeld-Jørgensen^1^, Ane-Kersti S. Knudsen^1^, Henning S. Pedersen^2^ and Manhai Long^1^
^1^Centre for Arctic Health & Unit of Cellular and Molecular Toxicology, Department of Public Health, Aarhus University, Aarhus, Denmark, ml@ph.au.dk; ^2^Primary Health Care Center, Nuuk, Greenland

#### 

The Greenlandic Inuit have high blood concentrations of environmental persistent organic pollutants (POPs), reported to be associated with age, smoking and intake of marine food. Studies have indicated that exposure to POPs during pregnancy can have adverse effect on fetal and child developmental health. The study aimed to assess geographical differences in exposure to POPs of pregnant women in Greenland. The pregnant women in this ACCEPT sub-study were enrolled during 2010–2011 and few from 2013. Questionnaire data and blood samples were collected from 207 pregnant women living in five Greenlandic regions (North, Disco Bay, Mid-West, South, East). Blood samples were analysed for 11 organochlorine pesticides (OCPs), 14 polychlorinated biphenyls (PCBs), 5 polybrominated diphenyl ethers (PBDEs), 15 perfluoroalkylated substances (PFAS) and 63 metals. The trend of higher marine food intake in the East and North was supported by a higher plasma n-3/n-6 fatty acids ratio. Significant regional differences were found for blood concentrations of PCBs, OCPs, PFAS and mercury, with significantly higher levels in the North and East regions. Most of the POPs were moderately associated to the n-3/n-6 ratio. The PFAS were significantly associated with the PCBs and OCPs in most regions. Moreover, in the North region, the PFAS were associated with both selenium and mercury. No significant regional difference was observed for PBDEs. The regional difference of POPs and mercury levels were related to marine food intake and plasma n-3/n-6 ratio. Compared to earlier reports, decreased levels of legacy POPs and perfluorooctane sulfonate (PFOS), perfluorooctanoic acid (PFOA) were observed. However, for other congeners such as perfluorohexane sulfonate (PFHxS), perfluorononanoic acid (PFNA), the level sustained. The detection of relatively high level of POPs and heavy metals in the maternal blood indicate that the foetus is exposed to these compounds that might influence the foetus development.

## Polymorphisms in Phase I and Phase II genes and breast cancer risk and relations to persistent organic pollutant exposure: an Inuit case-control study

### Mandana Ghisari^1^, Hans Eiberg^2^, Manhai Long^1^ and Eva C. Bonefeld-Jørgensen^1^
^1^Center for Arctic Health, Aarhus University, Aarhus, Denmark, mg@mil.au.dk; ^2^Department of Cellular and Molecular Medicine, University of Copenhagen, Copenhagen, Denmark

#### 

*Background*. The incidence of breast cancer (BC) among Inuit in Greenland has considerably increased from a very low level to approximately 60% of the incidence in Denmark. Previously, we reported that persistent organic pollutants (POPs) such as perfluorinated compounds (PFCs) and polychlorinated biphenyls (PCBs) are risk factors in BC development in Greenlandic Inuit women. Genetic polymorphisms in genes involved in xenobiotic metabolism and in oestrogen biosynthesis and metabolism might modulate the individual susceptibility to environmental carcinogens in relation to developing BC. *Aim and methods*. The present case-control study aimed to investigate the effect of polymorphisms in the genes CYP1A1, CYP1B1, COMT and CYP17, CYP19 and the BRCA1 founder mutation in relation to BC risk and to explore possible interactions between the gene polymorphisms and serum POP levels on BC risk in Greenlandic Inuit women. The study population consisted of 31 BC cases and 115 matched controls, with information on serum levels of PFCs, PCBs and organochlorine pesticides (OCPs). *Results*. We found an independent association of CYP1A1 (Val) and CYP17 (A1) with BC risk. An increased BC risk was observed for women with high serum levels of PFOS and PFOA and carriers of at least: one CYP1A1 variant Val allele; one variant COMT Met allele or the common CYP17 A1 allele. The risk of BC was not significantly associated with exposure to PCBs and OCPs, regardless of genotype for all investigated single nucleotide polymorphisms (SNPs). The frequency of the Greenlandic founder mutation in BRCA1 was as expected higher in cases than in controls. *Conclusion*. The BRCA1 founder mutation and genetic polymorphisms in CYP1A1 (Val) and CYP17 (A1) can increase the BC risk among Inuit women and the risk increase with higher serum levels of PFOS and PFOA. Serum PFC levels were a consistent risk factor of BC, but inter-individual polymorphic differences might cause variations in sensitivity to the PFC/POP exposure.

## Thursday June 11th 2015

### Plenary Session

#### Human development and well-being in the New Arctic: a discussion of key findings and major trends in the AHDR-II

##### Joan Nymand Larsen^1^ and Gail Fondahl^2^
^1^Stefansson Arctic Institute, Akureyri, Iceland, jnl@unak.is; ^2^University of Northern British Columbia, Prince George, Canada

###### 

This presentation provides an overview and discussion of the key findings and major trends in the recently published Arctic Human Development Report: Regional Processes and Global Linkages (1). The AHDR-II presents an update to the first AHDR (2004) in terms of an assessment of the state of Arctic human development. The report highlights the major trends and changes unfolding related to the various issues and thematic areas of human development in the Arctic over the past decade, and it identifies policy relevant conclusions and key gaps in knowledge, and new and emerging Arctic success stories. The state of human development is assessed in terms of arctic populations and migration; cultures and identities; economic systems; governance in the arctic: political systems and geopolitics; legal systems; resource governance; human health and well-being; education and human capital; globalization; and community viability and adaptation. The AHDR-II addresses critical issues and emerging challenges in Arctic living conditions, quality of life in the North, global change impacts and adaptation, and indigenous livelihoods. Among cross-cutting trends is the continued combination of rapid and stressful changes highlighted in the first AHDR (2004), amplified in rate and magnitude. Societal and environmental changes confront Arctic residents, local communities, and socioeconomic sectors and challenge their well-being. Gaps in development and human well-being persist between different groups, genders, levels and geographical locations (1). This presentation highlights and discusses the AHDR-II key findings, major trends and policy relevant conclusions on Arctic human development and living conditions.

**Reference**

1. Arctic Human Development Report: Regional Processes and Global Linkages (AHDR-II). Joan Nymand Larsen and Gail Fondahl (Eds). TemaNord 2014:567. Copenhagen: Nordic Council of Ministers; 2014.

## Paradoxes of alcohol consumption in the north as reflection of social contradictions

### Andrey Soloviev^1^, Andrey Paramonov^1^ and Irina Golchikova^2^
^1^Northern State Medical University, Arkhangelsk, Russia, ASoloviev@nsmu.ru; ^2^Arkhangelsk Regional Mental Hospital, Arkhangelsk, Russia

#### 

Alcohol consumption, including regional consumption patterns, continues to be one of the major social problems. In some of European countries, alcohol consumption is reduced, but the other half of the population began to drink more. North of Russia, unfortunately, belong to the second.

The aim of this work was to identify the paradoxes of alcohol consumption in the North as a reflection of contemporary social contradictions. Changing demographics and alcohol consumption at the population level often go hand in hand. With increasing intensity of alcohol abuse, a life expectancy reduced, growing number of deaths from unnatural causes, especially among men of working age. Social drinking traditions are important in the northern territories relating, in particular, with a harsh climate. They are distinguished by high levels of alcohol consumption on the “northern style”: reception mainly spirits, in large doses and with high frequency. The situation in the North of Russia exacerbates extensive development in the timber industry – it is important for the social development of the region, but at the same time associated with the production of technical ethyl alcohol (hydrolysis, sulfite and others.), having a very low cost and greater frequency of consumption by the population. Paradoxical changes in alcohol policy in Russia (e.g. anti-alcohol campaign under Mikhail Gorbachev) that relate to regional restrictions of time alcohol sales, periodic attempts to ban the sale of low-alcohol cocktails, the sudden decrease in prices for spirits and empowerment of alcohol advertising. Social instability, high rates of growth in food prices over the rising price of alcohol, poor quality anti-alcohol propaganda, setting the mass consciousness of the population north of the usefulness and necessity of reception of alcoholic beverages leads to the central paradox: the modern social system first creates powerful incentives for consumption, and then agonizingly long time to find the way how to deal with them!. A real alternative to alcohol abuse in the population of the northern territories is the creation of sound economic, environmental and spiritual conditions of life and learning experience of border areas of prevention of alcohol consumption.

## Improved Health Knowledge in the Arctic I

### Re-thinking “data gaps” in Canada’s north: understanding statistical information in a social field context

#### Chris Andersen
University of Alberta, Edmonton, Canada, chris.andersen@ualberta.ca

##### 

Despite the growing strategic importance of Canada’s north with respect to resource-extraction industries, comparatively little data exists with respect to the kinds of “quality of life” indicators that are taken for granted in Canada’s more southerly regions. Indeed, commentators have pointed out enormous and troubling data gaps in various northern communities, a problem exacerbated by the Canadian federal government’s recent switch from its long-standing census to a new National Household Survey (implemented for the first time in 2011). Beginning with the assumption that data collection is in fact part of a broader “statistical cycle” that involves, in addition, data creation, interpretation and dissemination. Bearing this in mind, the presentation offers three major “gaps” to the existing statistical field (with specific respect to indigenous populations) in Canada’s north: 1) the impact of the new National Household Survey on the ability of statistical actors to collect data on small communities; 2) the relative lack of “statistical literacy” among indigenous organizations to analyse existing data in ways that make sense to them; and 3) the manner in which “the north” has been essentialized by geographical understandings, leading to a lack of discussion about the number of indigenous individuals from/still connected to the north who actually live in “the south.”

## Ethnicity, statistics and health in Sweden – ways forward

### Per Axelsson
Umeå University, Umeå, Sweden, praksel@gmail.com

#### 

Today a majority of the world’s countries identify ethnic groups in their official statistics (1). Still there are countries with excellent population registers that do not make use of this possibility. Sweden is one of them. Even if Sweden has one of the world’s most extensive population registers allowing for excellent research in most fields where demographic data are needed, the country has prohibited the use of ethnic statistics in official statistics. This paper will examine the policy development around ethnic statistics in Sweden and suggest ways forward.

**Reference**

1. Morning AJ. Ethnic classification in global perspective: a cross-national survey of the 2000 Census Round. Popul Res Pol Rev. 2008;27:239–72. doi: http://dx.doi.org/10.1007/s11113-007-9062-5

## What kind of Sámi figures? Dealing with deficient Sámi ethnicity data in Norway

### Torunn Pettersen
Sámi allaskuvla/Sámi University College, Kautokeino, Norway, torunn.pettersen@samiskhs.no

#### 

In contemporary Norway, the use of Sámi ethnicity as a variable in studies aiming at quantitative knowledge on health and living conditions at the population level is challenged by insufficient Sámi-demographic data and blurred Sámi-ethnic boundaries. This situation can be related to, in particular, two factors. Firstly, in post-war Norway, only the 1970 national census has recorded ethnicity information about the indigenous Sámi, however restricted to selected areas in the north. Secondly, the effects of a long-standing assimilation policy have gradually lost ground during the recent decades in favour of upcoming Sámi revitalization. Taking as a starting point the adoption of a Sámi Act in 1987 and based on the premise that the Sámi people’s knowledge about themselves as a people should include meaningful statistical narratives, the talk will focus on ways of dealing with deficient Sámi ethnicity data in Norway. This will include the presentation of some numerical consequences of applying principles derived from Norway’s Sámi Act as a foundation for formalized inclusion criteria in population-based studies involving Sámi health and living conditions. It will be highlighted that it might not be possible to find an unambiguous solution regarding the operationalization of Sámi ethnicity.

**Reference**

1. Pettersen T, Magritt B. Which Sámi? Sámi inclusion criteria in population-based studies of Sámi health and living conditions in Norway – an exploratory study exemplified with data from the SAMINOR study. Int J Circumpolar Health. 2013;72:21813. doi: http://dx.doi.org/10.3402/ijch.v72i0.21813

## A call for improving health strategies regarding HLA-B27 carriers in the Arctic, considering traditional and scientific knowledge

### Lena Maria Nilsson
Umeå University, Umeå, Sweden, lena.nilsson@umu.se

#### 

Sometime around the year 1885, a relative of mine threw a holy stone (seijte) into the lake Keppejaur in Northern Sweden, to demonstrate himself as a good Christian (Manker 1957). Since that day, our family has been afflicted with eye disease and visual impairness. When medical experts lacked meaningful lifestyle advice regarding my rheumatic eye disease, this story became the base for a scientific review rendering the opposite. HLA-B27 carriers are common among Arctic peoples (1/6 Nordic north, 1/4 Sami, 1/3 Inuit, 2/5 Chukotka natives and 1/2 Haida) and less common elsewhere (1/12 Caucasians) (1). Arctic people may benefit from health advise based on their colonial experiences and traditional lifestyles, such as a diet rich in n3 fatty acids and spiritual stress reduction (2). Further, since HLA-B27 carriers are common in the North, this tissue sub-type should seriously be taken into consideration as a potential effect modifier in Arctic epidemiological research. It has already been shown that HLA-B27 carriers have an increased risk of rheumatic and intestinal disorders and a decreased risk of malaria, Hepatitis C and AIDS (3). Risk associations may similarly differ for a number of other diseases, not least those with an Arctic under- or over-representation, such as nephropathia epidemica.

**References**

1. Peschken CA, Esdaile JM. Rheumatic diseases in North America’s indigenous peoples. Semin Arthritis Rheum. 1999;28:368–91.

2. Feldtkeller E, Lind-Albrecht G, Rudwaleit M. Core set of recommendations for patients with ankylosing spondylitis concerning behaviour and environmental adaptations. Rheumatol Int. 2013;33:2343–9. doi: http://dx.doi.org/10.1007/s00296-013-2727-y

3. Mathieu A, Paladini F, Vacca A, Cauli A, Fiorillo MT, Sorrentino M. The interplay between the geographic distribution of HLA-B27 alleles and their role in infectious and autoimmune diseases: a unifying hypothesis. Autoimmun Rev. 2009;8:420–5. doi: http://dx.doi.org/10.1016/j.autrev.2009.01.003

## Ethnicity, health indicators and circumpolar comparisons: the case of Greenland

### Peter Bjerregaard^1,2^
^1^University of Southern Denmark, Odense, Denmark; ^2^University of Greenland, Nuuk, Greenland, pb@niph.dk

#### 

In Greenland, ethnicity is not recorded in the official registries. Instead place of birth is used as a proxy. Place of birth is (still) a reasonably valid indicator of ethnicity for adults currently living in Greenland but decreasingly so for children and not at all for Greenlanders living in Denmark. It must, however, be noted that in Greenland the proportion of Greenlanders amounts to approximately 90% so the bias of taking national figures to represent Inuit health is small. Approximately 10,000 ethnic Greenlanders live in Denmark and while place of birth is clearly an irrelevant proxy, a few attempts have been made at identifying these within the general population of Denmark. In health interview surveys, ethnicity has been estimated by questions about self-perceived ethnicity, language and grandparents’ ethnicity. Most such studies concentrate on Greenlanders, and a pragmatic way of identifying a person as a Greenlander is to let the interviewer and interviewee agree on a case by case basis. As part of the development of the Public Health Program for Greenland a set of 45 indicators of various aspects of health has been adopted. These combine indicators from registries and regular health surveys among adults and children. Although most indicators have been chosen based on their availability, there are a number of proposed indicators for which metrics are not available as well as indicators that need further development. Issues like marginalization and discrimination have not been included due to the status of Greenland as a semi-autonomous country but might be reconsidered as relevant for the health of regional minorities within the country. Some of the classic indicators such as infant mortality, smoking and obesity are comparable across circumpolar regions, while a major task lies ahead to agree on and develop a set of circumpolar health indicators for topics like for instance social inequity, quality of life, alcohol use patterns and diet.

## Improved Health Knowledge in the Arctic II

### Breaking the silence – suicide prevention through storytelling among indigenous Sámi

#### Per Henrik Bergkvist^1^, Lars Jacobsson^2^, Sofia Kling^3^, Anne Silviken^4^, Peter Sköld^2^ and Jon Petter Stoor^4^
^1^Jovnevaerie Sámi Community, Offerdal, Sweden, per-henrik.edqvist@igp.uu.se; ^2^Umeå University, Umeå, Sweden; ^3^Jämtland County Council, Östersund, Sweden; ^4^Sámi Norwegian National Advisory Board on Mental Health and Substance Abuse, Karasjok, Norway

##### 

The suicide epidemics among the indigenous peoples of the Arctic has been a great concern for several decades. Research initiatives have been undertaken, and a main issue for those efforts has been to identify best practices for interventions to promote youth resilience and mental health among indigenous youth. Lessons learned so far includes that best practice interventions must integrate elements of local, indigenous culture and community participation. In this conference, we would like to showcase an intervention that embodies these elements and might prove pivotal in designing future directions in circumpolar suicide prevention. The “Breaking the Silence” intervention for suicide prevention among Sámi has been initiated and carried out by Sámi reindeerherder Per Henrik Bergkvist of Jovnevaerie Sámi community (South Sámi area, Jämtland county, Sweden). He utilises the great potential in addressing the issue from an inside perspective. Through sharing his life′s story, he addresses mental health issues growing up as a Sámi reindeer herder, including talking openly of his struggles with suicidal ideation, loosing relatives to suicide and finally deciding that enough is enough; that he must “break the silence” and try to make a difference. And indeed he has. Per Henrik has been working together with indigenous community leaders, journalists, clinicians and academic researchers to get his message out. The intervention has been implemented through community participation and in that context proven to enable young indigenous men to talk openly (in culturally safe environments, of course) about experiences that are usually considered taboo (such as mental pain, romantic relationships and suicidal ideation) and in doing so; opening up a possibility for healing through sharing. Per Henrik’s presentation will be of interest to all those residing or working in indigenous circumpolar contexts or with health in general.

## What′s missing? Understanding and integrating indigenous perspectives on suicide and suicidal behavior among indigenous Sámi youth

### Jon Petter Stoor
Sámi Norwegian National Advisory Board on Mental Health and Substance Abuse, Bjurholm, Norway, jon.petter.anders.stoor@finnmarkssykehuset.no

#### 

The suicide epidemics among the indigenous peoples of the Arctic has been a great concern already for several decades. Research initiatives have been undertaken and a main issue for those efforts has been to identify best practices for interventions to promote resilience and mental health among indigenous youth (1). Lessons learned so far includes best practice interventions should build on elements of local indigenous culture and community participation. Further expanding the knowledge base in regard to these topics therefore carries potential to strengthen suicide prevention among indigenous peoples in the Arctic. In this conference, key-note presenter and reindeer herding Sámi Per Henrik Bergkvist showcases an intervention that embodies these elements and has proven culturally relevant in Sámi and several other indigenous Arctic contexts: the “Breaking the Silence” intervention for suicide prevention among Sámi youth. In this presentation, it is argued that indigenous perspectives, such as culture specific intervention programmes, have been non-existent in governmental suicide preventive efforts among Sámi in Sweden. Building on this lack of initiatives, Per Henrik′s intervention and other Sámi “from within” perspectives, including art work and a music video by renowned artist Sofia Jannok (2) are utilized to examine how suicide is understood within context. Such knowledge could be useful when trying to adapt suicide prevention efforts to fit the Sámi needs. Also, when integrating these “missing data” with perspectives on mental health and suicidology, new prevention and research opportunities might arise. It is argued that the examined Sámi perspectives on suicide emphasize the importance of cultural context and lack of political power, and that suicide preventive effort and research among the Sámi must address such issues. The presentation will be of interest for researchers, clinicians and policymakers in the field(s) of indigenous mental health and suicide prevention.

**References**

1. Council A. The evidence-base for promoting mental wellness and resilience to address suicide in circumpolar communities; 2013.

2. Jannok S, Östergren O. Áhpi (Wide as oceans) 2013 [cited 2015 Feb 18]. Available from: https://www.youtube.com/watch?v=hr13WV7UkgA.

## Mental Well-being in the North II

### The accumulation and interaction of factors related to deficits in health and risk of marginalisation in young adults in the Northern Finland

#### Tuula Hurtig^1^, Anja Taanila^1^, Hanna Ebeling^1^, Heli Koivumaa-Honkanen^2^, Pentti Kuronen^1^ and Kai Parkkola^3^
^1^University of Oulu, Oulu, Finland, tuula.hurtig@oulu.fi; ^2^University of Eastern Finland, Joensuu, Finland; ^3^Finnish Defence Forces, Helsinki, Finland

##### 

*Aim*. Marginalization due to deficits in individual resources is a health problem in many western societies, especially among young people. This research investigates the accumulation and interaction of individual deficits in mental health and well-being as potential risk factors of marginalization in young adults. It is hypothesized that especially depression, neuropsychiatric symptoms, and social and communication deficits are related to the risk of marginalization through failure in social or academic achievements, as well as rejection from military service. *Method.* The multidisciplinary study utilizes two large community-based samples in the provinces of Oulu and Lapland in Finland. First one is a 1-year military call-up sample including 4,500 men born in 1996. The call-up sample comprises all men in our target area attending obligatory military call-up examination in the year 2014 before entering military service in the next year. The other one is the Northern Finland Birth Cohort 1986 sample including 4,872 men born in 1985/1986. Internationally validated questionnaires measuring health, well-being, and educational and occupational achievements were completed in both study populations. In addition, information from national health registers are used. Both cross-sectional and longitudinal designs are obtained, as well as time-trend comparisons between the samples. *Results.* The data collection in the military call-up sample ended in December 2014 with a response rate of 65%. Preliminary results show that the symptoms or diagnosis of depression were common and were related to rejection from military service. *Discussion.* The research provides new information in identifying individuals and groups at risk of marginalization because of unfavorable conditions, health deficits or insufficient personal resources.

## Socioeconomic inequality in past year suicidal behavior among Greenland Inuit

### Christina V L Larsen^1,2^ and Peter Bjerregaard^1,2^
^1^National Institute of Public Health, University of Southern Denmark, Odense, Denmark, cll@niph.dk; ^2^Greenland Center for Health Research, University of Greenland, Nuuk, Greenland

#### 

*Background.* Suicide rates in Greenland are among the highest in the world and represent a major challenge for public health. Time trends in suicides and suicidal thoughts indicate a recent regional shift towards an increased marginalization between towns on the central west coast, villages, and East and North Greenland. This study aims to gain a better understanding of this shift in relation to socioeconomic differences at the individual level. *Material and methods.* Data on 2,612 Greenland Inuit aged 18–64 from the Inuit Health in Transition – Greenland Survey. Questions regarding past year suicidal thoughts and attempts were included in a self-administered questionnaire. Information regarding socioeconomic status was obtained through an interviewer-based questionnaire. Socioeconomic characteristics included household wealth, occupational status, educational level and level of engagement in the on going social transition. Data were analysed using multiple logistic regression models. *Results.* The prevalence of past year suicidal attempts was 9.6% for men and 12.8% for women. The prevalence of suicidal attempts was 3.6 and 4.4% for men and women respectively. Past year suicidal attempts and thoughts were associated with household wealth, occupational status as well as the level of involvement in social transition. In general, the OR increased with a decrease in occupational status and household wealth. Gender differences applied. *Conclusion.* The results indicate that socioeconomic characteristics at the individual level are important factors in understanding the distribution of suicidal behaviour in Greenland. Following this observation, the recent shift in regional distribution of suicidal thoughts is likely to be linked to the substantial differences in living conditions between the regions. Preventive strategies and interventions should be targeted according to the social inequality in suicidal behaviour.

## Mental health rehabilitation and responding to people with mental illness in the context of criminal courts in remote, mainly Inuit Arctic communities

### Priscilla Ferrazzi
Queen’s University, Kingston Ontario, ferrazzi@ualberta.ca

#### 

Criminal court mental health initiatives to reduce the number of people with mental illness in the criminal justice system do not exist in the Canadian Arctic territory of Nunavut despite their proliferation internationally. These initiatives belong to a family of “problem-solving” courts that address the root cause of criminal behaviour. Researchers have recently suggested using the problem-solving court principles that guide criminal court mental health diversion initiatives to deliver their rehabilitative objectives outside of well-resourced, specialized urban courts. But while the problem-solving court principles include the need for an effective “rehabilitative response,” a contemporary understanding of this response from mental health rehabilitation research – including current thinking about the role of culture in mental health recovery – is not well-explored. This study examined the potential for delivering the objectives of criminal court mental health diversion in Nunavut using problem-solving court principles. A qualitative multiple-case study involving 55 semi-structured interviews and focus groups was used to gather and analyse the experiences of justice and health professionals, representatives of community organizations and other community members in three communities. The study showed that the confounding effects of Inuit culture comprised a dominant issue in conversations about the three most-discussed objectives of problem-solving principles: identifying mental illness, approaches to treatment and multidisciplinary collaboration. Themes revealed in conversations about culture showed a clear resonance with culturally responsive concepts from mental health research known as “protective factors” in the mental health of Inuit and other indigenous peoples. This result suggests a consideration of protective factors may be essential for problem-solving court thinking for people with mental illness in the context of mainly Inuit Arctic communities.

**Reference**

1. Ferrazzi P. Therapeutic jurisprudence, rehabilitation, and responding to mental illness in the context of criminal courts in remote, mainly Inuit Arctic Communities. [PhD dissertation], Kingston, Canada: Queen’s University, 2015.

## Adverse Childhood Experiences (ACEs) in Alaska: new data fuels a statewide initiative

### Linda Chamberlain
Alaska Family Violence Prevention Project, Homer, USA, linda.chamberlain@alaska.gov

#### 

The landmark Adverse Childhood Experiences (ACEs) study, a retrospective case control design with over 17,000 adult participants demonstrated that early trauma including child abuse and neglect, exposure to domestic violence, growing up in a household with substance abuse or mental illness are common experiences that often cluster (1). ACEs were predictive, in a dose response relationship, of leading public health problems including suicide, substance abuse, obesity and depression. Worldwide, ACEs are recognized as leading social determinants of health. More than 20 states have collected population-based data through their participation in the United State’s Behavioral Risk Factor Surveillance System (BRFSS), the largest telephone survey in the world. The State of Alaska added the ACEs module to their BRFSS survey in 2013. While slightly more than one-third (35.6%) of adult Alaskans disclosed zero ACEs, more than one out of four (27.4%) indicated that they experienced three or more ACEs before the age of 18 (2). Comparison of BRFSS data for Alaska to a study combining BRFSS data for five other states indicated higher rates of several childhood adversities (3). Population attributable risk fractions indicated that ACEs are responsible for significant proportions of leading Alaskan public health concerns including heavy and binge drinking, smoking tobacco, poor physical health and asthma. These data suggest a downward trend in the prevalence of ACEs among older Alaskans compared to young adults. Data from the 2011–2012 National Survey of Children’s Health on the prevalence of ACEs among Alaskan children will also be discussed. This workshop will examine the data on the prevalence of ACEs in Alaska within the context of a statewide initiative to educate service providers and communities about ACEs.

**References**

1. Felitti VJ, Anda RF, Nordenberg D, Williamson DF, Spitz AM, Edwards V, et al. The relationship of childhood abuse and household dysfunction to many of the leading causes of death in adults: the Adverse Childhood Experiences (ACE) Study, Am J Prev Med. 1998;14:245–258.

2. Alaska Department of Health and Social Services, Section of Chronic Disease Prevention and Health Promotion, Adverse Childhood Experiences in Alaska: The Costs and Opportunities, [cited 2015 January 6], Available from: http://dhss.alaska.gov/abada/ace-ak/Documents/2013-BRFSS-ACEdatat20140915.pdf

3. Centers for Disease Control and Prevention, Adverse Childhood Experiences Reported by Adults – Five States, 2009, [cited 2015 January 5], Available from: http://www.cdc.gov/MMWR/preview/mmwrhtml/mm5949a1.htm

## Sex differences in microstructure of white matter tracts in a birth cohort sample of young adults

### Juha Nikkinen^1^, Vesa Kiviniemi^1^, Pirjo Mäki^1,2^, Tanja Nordström^2^, Solja Niemelä^2,3^, Zdenka Pausova^4^, Tomá Paus^5,6,7^, Juha Veijola^2^ and Lassi Björnholm^1,2^
^1^Oulu University Hospital, Oulu, Finland, Lassi.Bjornholm@oulu.fi; ^2^University of Oulu, Oulu, Finland; ^3^Lapland Hospital District, Rovaniemi, Finland; ^4^University of Toronto, Toronto, Canada; ^5^Rotman Research Institute, Baycrest, Toronto, Canada; ^6^Department of Psychology, University of Toronto, Toronto, Canada; ^7^Department of Psychiatry, University of Toronto, Toronto, Canada

#### 

*Introduction.* Sexual dimorphism of the brain is seen in differences in neural connectivity in white matter (WM)(1). A major motivation for studying brain sex differences arises from sex related vulnerabilities to developmental and psychiatric disorders. Studies using diffusion tensor imaging (DTI) have recently shown mainly more coherent diffusion, as higher fractional anisotropy (FA), and lower myelin or higher axonal caliber, as lower magnetization transfer ratios (MTR), in male versus female WM (2). In this study, two modalities of magnetic resonance imaging – DTI and magnetization transfer imaging (MTI) – were used to collect data for the analyses of WM tracts of 450 participants from the Northern Finland 1986 Birth Cohort (NFBC 1986). *Methods.* Of the 9,259 cohort members, 1,344 were invited and 468 (34.8%) participated in the field study. DTI, MTI and structural T1 images were produced of 191 male and 259 female participants. Group comparisons were made between male and female DTI-based WM skeletons and MTR maps using a voxel-wise permutation method. *Results.* Male FA was shown to be significantly higher than female FA throughout the WM (p<0.01). Also the comparison of the MTR maps showed significantly higher male MTR’s in most WM regions. *Conclusions.* The results show large differences in comparison to most other studies, in the sense of high male versus female FA and MTR. One can argue that the finding arises from our relatively large sample size and high quality of data collection.

**References**

1. Paus T. Sex differences in the human brain: a developmental perspective. In: Ivanka Savic, editor. Progress in brain research. Academic Press; 2010. p. 13–28.

2. Lenroot RK, Giedd JN. Sex differences in the adolescent brain. Brain Cogn. 2010;72:46–55.

## Frontline workers’ community response to intimate partner violence in the Northwest Territories, Canada

### Pertice Moffitt, Heather Fikowski, Elizabeth Thompson and Kyla Cherwaty
Aurora College, Canada, pmoffitt@auroracollege.nt.ca

#### 

Intimate partner violence (IPV) is deleteriously affecting the lives of women and their families in the Northwest Territories. The statistics show a staggering rate of reported violence in many scattered communities where resources are minimal. The purpose of this presentation is to address the findings to date of a 5-year Social Sciences Health Research Council funded project and to share implications which further our understanding of IPV in the Arctic and Subarctic communities of Canada. Utilizing IPV statistics and an environmental scan in the first year of the project, geographical information system maps spatially portrayed the picture of IPV and resources available. These maps helped to design and target communities in years 2 and 3 data collection processes. In year 2, frontline workers (n=31) were interviewed and data analysed using the constant comparative method of grounded theory. A central phenomenon of the symptom of hands are tied was identified with social processes of put-up, shut-up, and get on with life. This year, the model is shared with two communities being profiled in the north. The communities were selected based on the criteria of north/south and resourced/minimally resourced. Narratives are being created in the community profiling using focus groups, document appraisals, and purposeful interviews. It is anticipated that through the knowledge generated interventions can be identified to assist health planners develop policies to create and sustain non-violent communities.

## Use of life stories as memory training (reminiscence) in elderly care

### Janne Isaksen Engnes
University of Tromso – The Arctic University of Norway, Campus Hammerfest, Norway, janne.i.engnes@uit.no

#### 

*Introduction*. Elderly, older than 75 years, whom was born and raised in Finnmark was in Oct 1944 deported by the Germans, in The World War II. They were deported from their homes and experienced loss of livelihoods, slaughtering of animals, all while houses, buildings and boats all burnt. The people from Finnmark were relocated in the south of Norway to live as refugees in their own country. The elderly of Finnmark need to tell their life stories so that nurses can understand them better and address their care of needs. *Method.* The material is based on memory work within two groups of elderly women (n=9). One group: in day ward at a nursing home (n=6), the other: a focus group where elderly women living at home (n=3) was writing their own life stories. The communication was interactive, a moderator helped keeping on track. Both groups started as pilots.The purpose of the memory work was to have meaningful memory work, avoid stagnation and stress connected to their experiences and maintain memory and language in older age. *Results.* How deportation influenced their lifes was a recurring theme. It became clear that there is a need to have targeted conversations about the deportation. Many of the elderly still have trouble talking about the evacuation, and after they returned much of their story was suppressed and forgotten. *Discussion.* Elderly care in Finnmark is influenced by the fact that nursing home residents have a story that should be both told and understood. The deportation of Finnmark left a strong mark on the people it affected. Autentic movies and written accounts tell us that the deportation was both traumatising and brutal. Finnmark is rural and remote, and characterised by its small communities. Its nursing homes are also small, and if their staff have local and cultural knowledge, this can create a good opportunity to promote the idea that each resident’s story can be used for meaningful conversations and person-centred care. Old voices must be heard.

**References**

1. Immonen I. Nursing during the World War II: Finnmark County, Nothern Norway. Int J Circumpolar Health. 2013;72:20278, doi: http://dx.doi.org/10.3402/ijch.v72i0.20278

2. Serrani Azcurra DJ. A reminiscence program intervention to improve the quality of life-long term care residents with Alzheimer’s disease. A randomized controlled trial. Rev Bras Psiquiatr 2012;34, doi: http://dx.doi.org/10.1016/j.rpb.2012.05.008

3. Ivor F. Goodson. Narrative pedagogy. New York: Peter Lang; 2011.

## Mental Well-being in the North III

### Harmful drinking in Greenland in relation to socioeconomic position

#### Inger Katrine Dahl-Petersen, Christina Viskum Lytken Larsen and Peter Bjerregaard
National Institute of Public Health, University of Southern Denmark, Odense, Denmark, idp@niph.dk

##### 

*Background.* A high alcohol intake might have enormous consequences not only for the health and well-being of those afflicted, but also for their families and the community as a whole. Harmful drinking has been highlighted as an important risk factor for public health in Greenland. Understanding the socioeconomic patterns of harmful drinking is important for public health policy planning. The aim of this study was to examine harmful drinking in Greenland in relation to socioeconomic position. *Material and methods.* Cross-sectional population-based health survey among adults in 2014. Information on alcohol was obtained by a self-administered questionnaire (N=1795). A modified Cage test assessed harmful use of alcohol during past year. Socio-demographic information was collected by interview. Logistic regression models were used to examine the association between gender, age, occupational status, place of residence and a harmful drinking pattern. *Results.* Preliminary results show that aproximately one fourth of the participants were categorized as past year harmful drinkers (28% of men and 20% of women). Adjusted for age, the odds ratio of harmful drinking was highest for men (OR: 1.35; CI: 1.07–1.72) and lower by increasing age.The OR of harmful drinking was higher among participants living in smaller settlements and remote districts compared with towns on the Central West Coast (OR: 1.53; CI: 1.18–2.0) and regional differences were found. Harmful drinking was highest among the unemployed compared with those reporting occupational activity (OR: 2.24; CI: 1.45–3.46). *Conclusions.* The prevalence of harmful drinking is high in Greenland. Preliminary analyses suggest important regional variation and socioeconomic differences in the distribution of harmful drinking. This information could be useful in order to target future interventions and preventive strategies. Further analyses will be done to investigate social patterns in harmful drinking and changes over time.

## Suicide prevention in circumpolar regions

### Susan Chatwood^1^, Nathaniel Pollock^2^, Anne Silviken^3^, Petter Stoor^4^, Christina Larsen^5^, Michael Jong^2^, Gwen Healey^6^, Kue Young^7^ and Peter Bjerregaard^5^
^1^Institute for Circumpolar Health Research, Yellowknife, Canada, susan.chatwood@ichr.ca; ^2^Memorial University, St. John’s, Canada; ^3^University of Tromso – The Arctic University of Norway, Tromsø, Norway; ^4^SANKS, Sweden; ^5^University Southern Denmark, Odense, Denmark; ^6^QHRC, Nunavut, Canada; ^7^University of Alberta, Edmonton, Canada

#### 

During Canada’s chairmanship, the Arctic Council has prioritized joint initiatives to promote wellness and prevent suicide in circumpolar regions. With support from the CIHR – Institute of Aboriginal People’s Health, we have established a network of indigenous community leaders, researchers, clinicians and policy makers for our project, “Mental Well-Being and Suicide Prevention in Circumpolar Regions: Developing the Evidence Base and Identifying Promising Practices.” For this symposium, we will bring our team together to present findings from a series of projects focused on suicide prevention in Sweden, Greenland and Canada. Our team’s objectives are to (1) synthesize the state of knowledge about mental well-being and suicide prevention in circumpolar regions and indigenous communities; (2) create a statistical summary of suicide patterns and their determinants in circumpolar populations; and (3) identify best practices in youth suicide prevention related to the integration of culture, programme evaluation and scale-up. Overall, we are interested in developing a framework for suicide prevention that will support innovative, effective, scalable and locally adaptable interventions to promote youth resilience and mental health. Our fundamental aims are to improve the mental health of northern populations, reduce the burden of suicide and to contribute to the evidence-base for community and policy interventions in circumpolar contexts.

## Substance use, mental health challenges and systemic disadvantages: a northern Cree perspective

### Henri Pallard^1^, Carol Kauppi^1^, Jessica Hein^2^ and Amanda McLeod^1^
^1^Laurentian University, Greater Sudbury, Canada, hpallard@laurentian.ca; ^2^University of Toronto Scarborough, Toronto, Canada

#### 

Northern Cree communities have been faced with varied issues that have led to a “suicide pandemic” (1). This paper explores how the social context and systemic disadvantages of life in a remote northern Cree community contributed to mental health challenges, suicide attempts and persistent substance abuse for a Cree man from the western James Bay in northern Canada. Historical factors such as the colonial history of the James Bay, the consequences of residential schooling and child welfare practices, as well as experiences of racism and social exclusion led to an extended period of chronic substance use, homelessness and struggles with mental health. The 4-year research process utilized narrative methods which are compatible with oral traditions of northern Cree people and provide for a culturally sensitive approach to research. The presentation focuses on interconnections between the social context, traumatic stress, struggles with mental health, pathways into and out of institutionalization, homelessness and chronic use of substances. First, the narrative study explores some of the systemic causes from an indigenous perspective. Second, the in-depth data reveal some of the factors that work and do not work in facilitating transitions out of chronic substance use and homelessness for an indigenous person struggling with mental health effects of trauma. Third, the study enables non-indigenous peoples to have a better understanding of how systemic disadvantages contribute to individuals falling into substance use. Finally, the findings show how the formal service system did not work, why new perspectives and service responses are needed, and what changes are required to facilitate recovery from trauma and severe addiction for indigenous peoples.

**Reference**

1. Mushkegowuk Council. The Mushkegowuk Inquiry into Our Suicide Pandemic. Mushkegowuk Council, ON, Canada; 2014. [cited 2015 January 9]. Available from: http://www.mushkegowuk.com/?p=3438

## Suicide in the Northwest Territories (1999–2013): a review of the Coroner’s data

### John Omura^1^, Cathy Menard^2^ and Kami Kandola^3^
^1^University of British Columbia, Vancouver, Canada; ^2^Department of Justice, Government of Northwest Territories, Ottawa, Canada; ^3^Department of Health & Social Service, Government of the Northwest Territories, Ottawa, Canada, kami_kandola@gov.nt.ca

#### 

*Background.* Suicide is a common cause of mortality in Canada and has reached epidemic levels in regions of the North especially among Inuit populations. This study assesses suicide trends within the Northwest Territories (NWT) and also identifies risk factors associated with suicide as well as opportunities for enhanced surveillance and prevention. *Methods.* From 1999 to 2013, data were collected on all suicide deaths in the NWT by the Office of the Chief Coroner using the NWT Coroner Service Suicide Form. After compiling all data into a database, rates were calculated and descriptive analyses were performed. *Results.* A total of 125 suicide cases were identified during the study period. Age-standardized suicide rates were 8.0 per 100,000 person-years (PY) at risk for females (95% confidence interval [CI] 4.9, 11.2), 30.0 for males (95% CI 23.8, 36.2) and 19.4 for males and females combined (95% CI 15.9, 22.9). Rates were highest in males aged 14 to 44. While rates remain higher in males than females, the male rate appears to be declining. In terms of seasons, 74 deaths by suicide occurred in the spring and summer compared to 45 in the fall and winter seasons. Ethnic variation was identified, with highest rates among the Inuit population (58.2 suicides per 100,000 PY; 95% CI 40.6, 75.8). Frequently identified risk factors included: single status; unemployment; alcohol and drug use; depressed; emotionally distressed; issuing a statement of suicide intent; family breakup or separation; relationship breakup; death of a friend or relative; and extended separation from family due to school, medical, other. *Discussion.* Suicide rates in the NWT are high, particularly among males aged 14–44. Specific high risk populations include the Beaufort-Delta region and the Inuit. These findings, along with several identified risk factors, highlight opportunities for prevention strategies to mitigate the profound impact of suicide in the NWT.

**References**

1. Statistics Canada Leading causes of death in Canada – 2009; 2009. Available from: http://www.statcan.gc.ca/pub/84-215-x/2012001/tbl/T001-eng.pdf

2. Kirmayer LJ, Brass GM, Holton T, Paul K, Simpson C, Tait C. Suicide among Aboriginal people in Canada. Ottawa: The Aboriginal Healing Foundation; 2007.

3. Health Canada. First Nations and Inuit health: suicide prevention; 2013. Available from: http://www.hc-sc.gc.ca/fniah-spnia/promotion/suicide/index-eng.php

## Inuktitut Mental Health Glossary for clinicians: an online tool and mobile app

### Alex Drossos
McMaster University, Hamilton, Canada, drossos@mcmaster.ca

#### 

*Introduction.* Previous efforts to develop a general medical terminology translation glossary and electronic tool from English to Inuktitut (“MedInuktitut”) sparked a particular interest to expand and enhance the section on mental health terminology. This prior work, together with active clinical training in the field of psychiatry, informs the current project. The goals were to: a) learn about and better understand the use of terminology regarding mental health in Inuktitut, b) publish and disseminate the glossary through a simple to use online tool and mobile app for clinicians at the point of care and c) test the use of the terminology and make ongoing additions and changes to reflect more appropriate and locally understandable language. *Methods*. While initiating the current project, it became clear that such “mental health” terminology doesn’t exist in the fullest (and Western) sense in Inuktitut, at least in the same way that it does in English. As a result, ethnographic and linguistic reports and other documents were researched and reviewed concurrently with reports prepared by the Government of Nunavut, Canada which has ongoing efforts to generate and standardize medical and health terms. A subset of terms that were deemed the most important and likely to be used by mental health clinicians were then selected to be used in the online tool and mobile app. The design and platform of the electronic media (e.g. the online tool and mobile app) existed from previous efforts as described above, thus the newly generated Mental Health Glossary was simply added to the previous database. *Conclusions*. Use testing of the Mental Health Glossary is ongoing and will take some time given the small numbers of mental health clinicians able to test the electronic tools in the field. It is hoped that the continued efforts and updates to the glossary will improve clinician-patient communication and do so in a more culturally appropriate and safe manner.

**References**

1. Kirmayer L, Fletcher C, Corin E, Boothroyd L. Inuit concepts of mental health and illness: an ethnographic study. Montreal: Department of Psychiatry, McGill University; 1997.

2. Evic L, Nesbitt G, Douglas C (eds.). Inuktitut for health professionals: a phrasebook. Iqaluit, Canada: Pirurvik Press; 2011.

Penney C. Medical Glossary. Iqaluit, Canada: Nunavut Arctic College; 1995.

## Disease Profiles in the Changing Climate

### Climate change: the next challenge for circumpolar mental health?

#### Ashlee Cunsolo Willox^1^, Inez Shiwak^2^ and Michele Wood^3^, The IMHACC Team^2^, The Rigolet Inuit Community Government^2^
^1^Cape Breton University, NSW, Canada, ashlee_cunsolowillox@cbu.ca; ^2^Rigolet Inuit Community Government, Rigolet, Canada; ^3^Nunatsiavut Department of Health & Social Development, Nain, Canada

##### 

Indigenous peoples in the Circumpolar North are at the frontlines of climate change, experiencing rapid shifts in weather patterns, snow and ice conditions, and animal and plant behaviour. Since many indigenous peoples in this region continue to rely on the natural environment for livelihoods, cultural continuity, identity and well-being, even subtle alterations in climate and environment can have effects on health, including psychological well-being. Although knowledge is still limited about the connections between climate change and mental health, evidence is indicating that impacts may be felt at both the individual and community levels and are expected to be widespread, cumulative and unequally distributed. Recognizing this, the five Inuit Community Governments of Nunatsiavut, Labrador, Canada conducted a community-based and community-led regional study on the impacts of climate change on mental health. Data were gathered through 120 in-depth interviews from men and women of all ages in all five Inuit communities in the region. Through this research, participants reported: intense emotional reactions associated with loss of activities, identity and sense of place, including grief, anxiety, stress and distress; real and potential increases in addictions; potential aggravation of acute anxiety disorders and major depression; and potential increases in suicide ideation. When combined with other stressors from changes in governance structures, economies and social structures in the North, as well as the intergenerational impacts of colonialism, and greater disparities in health outcomes, there are further potential climate-related impacts to individual and community well-being. These findings contribute to the emerging research on the psychosocial dimensions of climate change, and provide a baseline of potential pathways through which climate change may impact on mental health in the North and globally.

## Health effects of climate change in Alaska: findings from a multi-year community-based Sentinel surveillance system

### David Driscoll, Rebecca Barker, Janet Johnston, Erica Mitchell and Susan Renes
University of Alaska, Anchorage, AK, USA, DDriscoll@uaa.alaska.edu

#### 

In order to develop effective adaptations to environmental effects of climate change, community-level assessment of health outcomes related to these effects are needed. Sentinel surveillance systems were implemented in a cross-section of Alaskan communities widely distributed across northwestern, central and southeast Alaska (8 communities in round 1 in 2011/12; 10 in round 2 in 2013/14). On average, 42 residents in both cohorts provided monthly observations of local weather, hunting and harvesting experiences, food and water safety, and health. Open-ended text fields for each theme and for general observations were included. Injury as a result of unusual weather was more likely during months in which the weather was unusual for that time of year (OR=2.8, 95% CI 1.6 – 4.9) and more likely in months when community members changed travel plans due to unusual weather (OR=4.5, 95% CI 2.9 – 6.9). Other cold-related injuries were only more likely in months when travel plans changed (hypothermia OR=3.5, 95% CI 1.7 – 7.0; frostbite OR=3.5 95% CI 2.2 – 5.6; and mortality as a result of unusual weather OR=3.7 95%, CI 1.9 – 7.3). Community residents were also 1.5 times as likely to report allergic asthma or pollen allergies during unusually warm environmental conditions. In open-ended responses, residents reported that air-pollution sources, including increasing exposures to pollen and wildfire smoke, were causally associated with respiratory health problems in warmer weather. We found significant associations between unusual weather conditions and cold-related morbidity and mortality in two rounds of surveillance data collection across the state. Additional adverse health outcomes were associated with increasing prevalence of icy conditions in winter, and unusually warm and dry conditions in summer. Further, discussions of the surveillance data with local residents provided insights into prospective causal relationships between health outcomes and climate change.

## Cardiovascular risk factors in patients with diabetes mellitus and coronary artery disease in Western Siberia: north-south difference

### Vadim Kuznetsov, Elena Yaroslavskaya, Grigoriy Kolunin, Georgiy Pushkarev, Elena Gorbatenko and Luiza Marinskikh
Tyumen Cardiology Center, Tyumen, Russia, kuznets@cardio.tmn.ru

#### 

*Background*. The north-south gradient of cardiovascular risk factors exists in many countries. We hypothesized that the north-south gradient could be observed in patients with coronary artery disease (CAD) and diabetes mellitus (DM) in Western Siberia. *Aim*. To compare cardiovascular risk factors in patients with CAD and DM in the North and the South of Tyumen region (Western Siberia). *Methods*. The study included 512 patients with type 2 DM selected from 8,875 consecutive patients with angiographically proven CAD (>50% stenosis). 384 patients were permanent residents of the North of Tyumen region (north of latitude 64 degrees N) (group 1), and 128 patients were permanent residents of the South of Tyumen region (south of latitude 57 degrees N) (group 2). Cases of acute coronary syndrome were excluded. *Results*. Patients of group 1 were significantly younger compared to group 2 (53.9±6.7 vs. 58.1±7.2 years, p<0.001). Smoking and alcohol consumption were higher in group 1 (58.9 vs. 30.0%, p<0.001 and 29.3 vs. 10.5%, p=0.001, respectively). Most of the patients in both groups had arterial hypertension (90.4 and 96.1%) and obesity (85.9 and 80.4%). The level of plasma total cholesterol did not differ significantly between groups (5.5±1.5 and 5.6±1.5 mmol/l). The rate of patients with three CAD risk factors or more was significantly higher in patients of group 1 (34.3 vs. 19.0%, p<0.001). The clinical manifestations of CAD were not significantly different between the groups. Multivariate analysis showed the independent associations of young age and smoking with residence in high latitudes. *Conclusion*. The north-south gradient of cardiovascular risk factors was observed in patients with DM and CAD in Western Siberia.

## Tularemia mapping in northernmost Sweden: seroprevalence and a case-control study of risk factors

### Maria Furberg and Johansson Anders
Umea University, Umeå, Sweden, maria.furberg@climi.umu.se

#### 

*Background.* Sweden has one of the highest incidences of tularemia type B in the world, with major local variations. Our recent study has shown a 10-fold increase in Sweden between the periods of 1984–1998 and 1999–2012 (1), with the highest incidences in the north. What proportion of the population in Sweden that has had tularemia is unknown, and it is still uncertain what life activities are associated with high risk of being infected. Tularemia creates lifelong immunity, and antibodies are detectable decades after the infection. No modern studies of tularemia seroprevalence exists in Sweden and the true number of cases may be larger than what is suggested by the disease notifications. Tularemia is considered a climate sensitive infection, and it is important to establish baseline data for monitoring changes over time. *Method.* We are analysing 1,613 serums from a random population sample from Norrbotten (BD) and Västerbotten (AC) counties. The serum donors have answered questions about suspected tularemia contraction risk factors. We will also investigate if it is possible to correlate seroprevalence in different areas with incidence figures calculated using disease surveillance data (1). In addition, we will analyse risk factors of tularemia using a case-control study design with questionnaires sent out after an outbreak in 2012 in BD and AC counties. Potential risk factors investigated include time spent on various outdoor activities, housing and the residence distance to water. This study includes 195 unique cases and controls. *Results.* The results will describe which areas and what activities in northern Sweden that carries the highest risk of contracting tularemia and also give an idea about to what extent people get sick without seeking medical care. This study is part of a larger project aiming at creating a tularemia early warning system. The data has been collected and is at present being analysed. Preliminary results will be presented at the conference.

**Reference**

1. Desvars A, Furberg M, Hjertqvist M, Vidman L, Sjöstedt A, Rydén P, et al. Epidemiology and ecology of tularemia in Sweden, 1984–2012. Emerg Infect Dis. doi: http://dx.doi.org/10.3201/eid2101.140916

## Arterial hypertension resistance in the North depending on the adaptive type of the organism adaptive reserves mobilization

### Vyacheslav Hasnulin^1,2^, Mikhail Voevoda^1^, Olga Artamonova^1^ and Pavel Hasnulin^1^
^1^FSBI Research Institute of Therapy and Preventive Medicine SB RAMS, Novosibirsk, Russia; ^2^Siberian Institute of Management, branch of Russian Presidential Academy of National Economy and Public Administration, Novosibirsk, Russia, hasnulin@ngs.ru

#### 

It is found that non-indigenous inhabitants of the North with the hyporeactive adaptive type of an organism adaptive reserves efficient mobilization in the chronic northern stress conditions (“stayers”), showing a higher resistance to emotional stress, desynchronosis in unusual photoperiodism, high functional possibilities of the liver, exhibit lower risks of arterial hypertension as well as associated pathologies development and progression while living in the extreme conditions. Hyperreactive persons (“sprinters”) proved to be less resistant to the northern stress and, respectively, to the arterial hypertension formation; they also have a higher level of psychoemotional stress, a higher degree of dysadaptation manifestations, reliable decrease in recovery potential, mental capacity reduction and an increase in desynchronoses and hepatocellular dysfunction. The notion that the individuals with “stayer” adaptive type have gene- and phenotypically fixed mechanisms for more effective regulation of the circulatory system in extreme climatic conditions in the North, which reduce the risk of arterial hypertension, is now making its appearance. There possibility of stimulation of recovery processes and blood pressure correction and desynchronoses in the Arctic inhabitants with the arterial hypertension at the health resorts in middle latitudes is considered.

## Population Health III

### Aboriginal engagement in healthy living and social determinants of health in the Northwest Territories

#### Vee Faria^1^, Kami Kandola^1^, Karen Blondin-Hall^1^, Donna Manca^2^, Carolina Aguilar^2^ and Nicolette Sopcak^2^
^1^Department of Health and Social Services, Government of the Northwest Territories, Ottawa, Canada, kami_kandola@gov.nt.ca; ^2^University of Alberta, Edmonton, Canada

##### 

Health outcome disparities in the Northwest Territories (NWT) have been linked to community variations in income, education, housing and other social determinant issues. Due to our ageing population, there is an increase in the burden of chronic diseases, particularly cancer, cardiovascular disease and diabetes. Resources to support improvement are concentrated in the regional centers and the capital Yellowknife. An additional approach beyond the clinical setting was required for the most vulnerable populations. While the Building on Existing Tools to Improve Chronic Disease Prevention and Screening in Primary Care (BETTER 2) programme was designed for primary care practitioners, the aboriginal engagement component enhanced the primary care support model by directly targeting the communities. One of the methods was in promoting well-recognized aboriginal champions from various regions in NWT who best exemplified healthy living practices by sharing their stories in five motivational and inspirational videos focusing on the following themes: physical activity, healthy eating, tobacco cessation, alcohol management and healthy relationships. Facilitator workshops, direct engagement with community representatives and aboriginal organizations on training related to chronic disease prevention and screening occurred. Preliminary results of the impact of training will be available at the time of the conference.

**References**

1. Campbell-Scherer D, Rogers J, Manca D, Lang-Robertson K, Bell S, Salvalaggio G, et al. Guideline harmonization and implementation plan for the BETTER trial (Building on Existing Tools to Improve Chronic Disease Prevention and Screening in Family Practice). Canadian Medical Association Journal Open;2014:2:E1–10.

2. Grunfeld E, Manca D, Moineddin R, Thorpe KE, Hoch JS, Campbell-Scherer D, et al. Improving chronic disease prevention and screening in primary care: results of the BETTER pragmatic cluster randomized controlled trial. BMC Fam Pract. 2013;14:175.

3. Manca DP, Aubrey-Bassler K, Kandola K, Aguilar C, Campbell-Scherer D, Sopcak N, et al. Implementing and evaluating a program to facilitate chronic disease prevention and screening in primary care: a mixed methods program evaluation. Implement Sci 2014;9:135.

## Changes in health effects of substance use in a population of chronically homeless people after moving into a Housing First facility in Alaska

### David Driscoll, Sarah Shimer, Rebecca Barker, Travis Hedwig and Janet Johnston
University of Alaska, Anchorage, AK, USA, DDriscoll@uaa.alaska.edu

#### 

*Background*. The Housing First (HF) model is an evidence-based approach to provide permanent supported housing and reducing substance use and mental health disorders in chronically homeless populations. Harm reduction is one of the central tenets of HF with an emphasis on pragmatically reducing the adverse consequences of substance abuse by providing housing without preconditions of sobriety or treatment. Beginning in December 2011, two project-based HF facilities opened in Anchorage and Fairbanks, Alaska to house the most vulnerable urban homeless chronic alcoholics. *Methods*. A total of 94 tenants at the two facilities participated in a pre-post evaluation to assess the utility of the HF model in Alaska. Residents completed semi-structured interviews and quantitative health surveys when they moved in and again a year later. Interviews addressed social connections, engagement with services, health status, housing and employment history and alcohol consumption. Staff members were also interviewed about their experiences with tenants and working at HF. *Results*. Tenants reported significant declines in both quantity and frequency of alcohol consumption 1 year after moving into HF. Tenants further reported higher levels of social connectedness with family and friends, fewer depressive symptoms and less pain. While tenants reported improved mental welfare, they also presented with persistent medical ailments and struggled with grief and alcohol. Case management and attentive staff had an influence on tenant participation in services and recreation other than alcohol consumption. *Conclusions*. This evaluation adds to the evidence that living in a project-based HF facility provides significant health benefits over living in camps and shelters, although challenges still remain. Successful implementation of HF in Alaska provides added support to policymakers considering project-based HF facilities in other urban centers in the circumpolar north.

## A community-based design process for reaffirming cultural heritage

### Amanda McLeod^1^, Carol Kauppi^1^, Jessica Hein^2^ and Henri Pallard^1^
^1^Laurentian University, Greater Sudbury, Canada, homeless@laurentian.ca; ^2^University of Toronto Scarborough, Toranto, Canada

#### 

A design charrette was conducted with northern indigenous peoples with a focus on their housing needs as related to their cultural heritage. The research activities were conducted in 2010 and 2011 with Cree people from several First Nation communities on the western James Bay in northern Ontario, Canada. The results of the workshops and the architectural designs that emerged were presented at a conference held in a James Bay community in 2012 as a way to continue the dialogue with Cree people. This community-based project reflects a set of activities that supported cultural affirmation through the use of focus groups combined with a design charrette activity. Using a wide range of materials and drawing, modelling and sculpting techniques, the Cree participants designed their ideal housing. The research process encouraged discussion about memories of their traditional patterns of life, cultural heritage and living circumstances. Participants also discussed the housing issues and challenges they were facing at the time of the study. This poster presentation summarizes the research process and results from the focus groups and from the design charrette which reveal the strengths of utilizing community driven research methods. The poster presentation includes images of materials used, the housing models created by the participants and the architectural designs emerging from this project. The traditional forms found within the snowshoe, the canoe, the tipi and the shaapuhtuwaan inspired the creative process and the design of culturally appropriate housing. The designs centred on housing as a response to Cree material culture, social structure and ways of experiencing the land. The research and design process investigate the myriad ways in which architecture can act as a cultural tool that reaffirms a sense of place and responds to living patterns and the northern climate.

## Indigenous Health VI: “Health status overview (2)”

### Anthropometric parameters predict metabolite biomarker concentrations among school-aged Nunavik Inuit children

#### Michel Lucas^1^, Salma Meziou^1^, Audray St-Jean^1^, Cynthia Roy^1^, Marc Medehouenou^2^, Pierre Ayotte^1^ and Gina Muckle^1^
^1^Laval University, Quebec, Canada, michel.lucas@crchuq.ulaval.ca; ^2^Population Health and Optimal Health Practices Research Unit, CHU de Québec Research Centre, Quebec, Canada

##### 

Branched-chain amino acids (BCAAs) and their metabolites (acylcarnitines), as well as aromatic amino acids (AAAs) concentrations are elevated in humans with diet-induced obesity and are actually viewed as the best predictor of type 2 diabetes (T2D). In Canada, obesity and T2D prevalence are higher in indigenous compare to non-indigenous people. This study aims to understand the association between anthropometric parameters and metabolite biomarkers in Nunavik Inuit children. Data were collected in 2005–2010 among 250 school-aged children (8–14 years; 51% girls) from the Nunavik Child Development Study (NCDS). Concentrations of metabolites (BCAAs, AAAs and other amino acids, and acylcarnitines) were considered as dependant variables. Independent variables were anthropometric parameters [body mass index (BMI), triceps skinfold thickness (TST) and subscapular skinfold thickness (SST)]. Participants were classified as normal weight, overweight and obese according to International Obesity Task Force (IOTF) classification systems. Analysis of variance was used to assess associations between weight status categories and metabolites. Multivariate linear regression was used to assess relationship between one-standard deviation increase of anthropometric and metabolite concentrations. Prevalence of overweight was 26.9%, with 6.6% obesity. Differences between metabolite concentration means were only statistically significant for valine (BCAA) and tyrosine (AAA). We noted that increase of anthropometric measurements (BMI, TST and SST) were associated with higher valine concentration, whereas isoleucine (BCAA) and tyrosine were only raised with increase of BMI. In this school-aged children population, we noted that plasma concentrations of BCAA and AAA were raised with increase of anthropometric measurements. Longitudinal studies are deserved to investigate if excess adiposity and higher metabolite biomarkers allow early identification of individuals at high risk of future T2D.

## A comparative cohort study of seizures in Greenlandic and Danish children

### Jacqueline Møller Mistry^1^, Sascha Wilk Michelsen^1^, Bolette Søborg^1^, Anders Koch^1^, Maria Miranda^2^ and Malene Landbo Børresen^1^
^1^Statens Serum Institut, Copenhagen, Denmark, mlb@ssi.dk; ^2^Neuropædiatrics, Herlev Hospital, Herlev, Denmark

#### 

*Background.* Only little research on epilepsy in Greenlandic children exists, with inconsistent results. The aim of this study was to identify the incidence of seizures in both Greenland and Denmark for comparison. *Methods.* A register-based cohort study of all children in Greenland and Denmark aged 0–15 in the period from 1987 through 2011 was conducted. Using the greenlandic patient register (GLPR) and the Danish National Patient Register, cases were identified and coupled to demographics through the Civil Registration System. Incidence rates (IRs) per 100,000 person years (pyrs) and incidence rate ratios (IRRs) for comparison were calculated. *Results.* The study showed an epilepsy IR of 204.0/100,000 pyrs (189.9–219.2) in Greenland and 170.5/100,000 pyrs (168.9–172.1) in Denmark. Moreover, Greenlanders in Denmark had a higher IR of epilepsy than Greenlanders in Greenland, with an IRR of 1.53. For febrile seizures, the IR was 175.5/100,000 pyrs (162.4–189.6) in Greenland and 263.3/100,000 pyrs (261.4–265.3) in Denmark. However, the IRR for Greenlanders in Denmark compared with Greenlanders in Greenland was 1.60. The IRR of febrile seizures for children living in settlements compared with Nuuk was 0.40/100,000 pyrs (0.29–0.55). The risk of epilepsy subsequent to a diagnosis of febrile seizures was doubled in Greenland compared with Denmark. *Discussion.* The results pointed towards under-reporting of epilepsy in Greenland, and ethnicity was found a likely risk-factor. Furthermore, febrile seizures must be under-reported in settlements in Greenland. Additionally, the higher IR of epilepsy in Greenland compared with Denmark might be due to genetics, perinatal factors, CNS infections etc. However, further investigation and validation of GLPR concerning seizure diagnoses is needed.

## Has social equality in health increased or decreased in Greenland since 1992?

### Peter Bjerregaard, Inger K Dahl-Petersen and Christina VL Larsen
National Institute of Public Health, Copenhagen, Denmark, pb@niph.dk

#### 

In 1992, the Greenland Government took responsibility for health care and thereby for creating a more equal distribution of health in the country. The purpose of the presentation is to analyse the development of health equality in Greenland during the last 25 years. With a Gini-coefficient of more than 34 (in 2013) which is higher than the EU average of 30 and much higher than that of other Nordic countries, economic inequality is prominent in Greenland. This is a major risk factor for inequality in health. Inequality in health exists at several dimensions, for example, between Greenland and Denmark, regionally between towns and villages and between West and East Greenland, and at the individual level according to social position. For the period 1990–2014, we have analysed inequality in health for selected indicators of physical and mental health. Infant mortality and suicides were analysed using the mortality registries for Greenland and Denmark; suicidal thoughts, self-rated health and smoking were analysed using data from four population health surveys during the period. As examples, suicide rates in East Greenland remained three times higher than in the rest of the country throughout the period of study while the prevalence ratio of smoking among the least wealthy relative to the wealthiest Greenlanders increased from 1.2 to 1.8. Although there may be exceptions equality in health has not generally increased in Greenland during the last 25 years.

## Social determinants of Inuit health

### Anna Fowler^1^, Elizabeth Ford^1^ and Sharon Edmunds-Potvin^2^
^1^Inuit Tapiriit Kanatami, Ottawa, Canada; ^2^Nunavut Tunngavik Incorporated, Ottawa, Canada, sedmunds-potvin@tunngavik.com

#### 

Inuit continue to face significant health disparities compared to non-Inuit Canadians including comparatively lower life expectancies, high rates of infant mortality and the highest suicide rates of any population group in the country. Effective solutions will involve addressing the underlying determinants and focusing on a holistic view of health. In 2014, Inuit Tapiriit Kanatami (ITK) developed a report on the Social Determinants of Inuit Health in Canada. Drawing from current data sources and consultation with Inuit organizations, agencies and governments, this paper highlights the key social determinants of health that are relevant to Inuit in Canada including: quality of early childhood development, culture and language, livelihoods, income distribution, housing, personal safety and security, education, food security, availability of health services, mental wellness and the environment. While summarizing the key challenges that exist for each of these areas, the report also highlights practices that have resulted in positive outcomes. This Social Determinants of Inuit Health in Canada Report is an Inuit-specific resource designed to support public health activities across the Inuit regions in Canada and to function as a reference for organizations and governments working within the Canadian health and social services sector. While progress is being made, substantial work is still required to address the conditions that lead to poor health outcomes for Inuit.

## Addressing historical legacies in the assessment of resource development: why it matters for indigenous well-being

### Ben Bradshaw and Jennifer Jones
University of Guelph, Guelph, Canada, jjones14@uoguelph.ca

#### 

Environmental Assessment has long struggled to effectively identify and mitigate community and human health impacts associated with resource development, especially in Northern, largely indigenous, jurisdictions. This is changing with routine use of assessment mechanisms like Health Impact Assessments (HIAs), supplemental permitting rules and Impact and Benefit Agreements (IBAs) between indigenous groups and mine developers. Coincident with this shift in practice is a growth in research that recognizes diverse concepts and complex drivers of indigenous well-being; as a result, it is increasingly common for researchers to speak of the “good life” and to recognize health disparities that are based in experiences with poverty, stress, trauma, cultural erosion and environmental dispossession. Unfortunately, little of this research has come to influence contemporary HIA practices, supplemental permitting rules and the content of IBAs. Hence, missing from these governance and assessment mechanisms is the recognition that indigenous well-being and resource development are complicated by legacies of colonialism and assimilation policies. What matters to indigenous communities, and what is captured in an HIA, supplemental permitting rules or an IBA, seldom coincide. Drawing on empirical research that documented indigenous participation in an HIA and the negotiation and implementation of IBAs, this paper calls for the refinement of frameworks governing northern development in order to better understand and respond to the complexities that inform indigenous well-being. Ideas for moving forward current research and practice will be shared and discussed. Environmental Assessment has long struggled to effectively identify and mitigate community and human health impacts associated with resource development, especially in Northern, largely indigenous, jurisdictions. This is changing with routine use of assessment mechanisms like HIAs, supplemental permitting rules and Impact and Benefit Agreements (IBAs) between indigenous groups and mine developers. Coincident with this shift in practice is a growth in research that recognizes diverse concepts and complex drivers of indigenous well-being; as a result, it is increasingly common for researchers to speak of the “good life” and to recognize health disparities that are based in experiences with poverty, stress, trauma, cultural erosion and environmental dispossession. Unfortunately, little of this research has come to influence contemporary HIA practices, supplemental permitting rules and the content of IBAs. Hence, missing from these governance and assessment mechanisms is the recognition that indigenous well-being and resource development are complicated by legacies of colonialism and assimilation policies. What matters to indigenous communities, and what is captured in an HIA, supplemental permitting rules or an IBA, seldom coincide. Drawing on empirical research that documented indigenous participation in an HIA and the negotiation and implementation of IBAs, this paper calls for the refinement of frameworks governing northern development in order to better understand and respond to the complexities that inform indigenous well-being. Ideas for moving forward current research and practice will be shared and discussed.

## Midway evaluation of Greenland’s tuberculosis programme

### Thomas Rendal, Flemming Stenz and Turid Skifte
National Board of Health, Nuuk, Greenland, thre@nanoq.gl

#### 

Despite massive strategic efforts, high prevalence, high incidence and high rates of infection still characterize the tuberculosis situation in Greenland. Following a major incidence-peak in 2010, the process of developing a new TB programme was set in motion involving, among other things, a country visit by a WHO Expert Group. The programme was implemented in autumn of 2011. In 2014, The National Board of Health conducted a thorough review of the programme in cooperation with the WHO Regional Office for Europe and other outside experts. Although the evaluation found the Greenlandic TB programme to be sound, several key areas of improvement were pinpointed: 1) The use of aggressive interventions, when localized epidemics are detected, should be strengthened. 2) Efforts should be made to lower the current high rates of “loss to follow-up” and “not evaluated” case outcomes, and existing data should be used to identify these patient groups. 3) Greenland should expand the use of DOT using available modern technology, and cease the use of Rifampicin in standalone tablet form. 4) Diagnostic capabilities should be strengthened at the lowest possible setting. 5) Supervision from central level should be expanded.

## Establishment of oral health surveillance in Alaska; use of electronic dental records

### Timothy Thomas^1^, Dane Lenaker^2^, Dana Bruden^3^, Richard Baum^3^ and Tom Hennessy^3^
^1^Alaska Native Tribal Health Consortium, Anchorage, AK, USA, tkthomas@anthc.org; ^2^Yukon Kuskokwim Health Corporation, Bethel, AK, USA; ^3^Arctic Investigation Program, Centers for Disease Control and Prevention, Anchorage, AK, USA

#### 

*Background.* For many American Indian and Alaska Native (AI/AN) communities, the prevalence of dental cavities among children is the highest in the US 2008 oral health survey in five rural Alaska communities, which showed 91% of children aged 4–15 had cavities. Conducting comprehensive oral surveys is costly and labor intensive. AI/AN dental care in south-western Alaska is provided through the regional tribal health corporation. We explored the use of electronic dental records for surveillance purposes. *Methods.* The regional dental unit maintains three databases that capture the existing condition (e.g. decayed, missing teeth) and treatment provided on- and off-site (e.g. fillings, crowns). We merged data for all children age ≤ 6 years seen by dental providers between 2003 and 2011 and determined the accumulated decayed, missing, filled teeth (dmft) score for each child. We provide an average dmft score for children aged 6 years in 2009, 2010 and 2011 seen by the dental system within 2 years and compare dmft scores for communities with/without in-home piped water or a dental health aid therapist (DHAT). *Results.* Between 2009 and 2011, the proportion of children seen by the dental system increased from 58 to 83%; however there was no change in dmft score (9.6 in 2009 and 10.9 in 2011). The 2011 dmft scores in communities with and without in-home piped water were 10.3 and 12.0 respectively (p<0.01). In the 20 DHAT communities, 99% of the children were seen compared to 73% in the 29 non-DHAT communities. Dmft scores were lower in DHAT (10.3) versus non-DHAT villages (11.2), (p=0.05). *Conclusions.* Using the electronic dental record, we were able to establish dmft scores for a representative portion of the region’s population of 6 years olds and compare by characteristics of community of residence. Continued surveillance will allow monitoring of trends and evaluation of interventions.

## Is self-rated health associated with clinical measures of health among the Inuit of Greenland and Nunavik?

### Inger Katrine Dahl-Petersen^1^, Marie Baron^2^, Christina Viskum Lytken Larsen^1^, Mylène Riva^2^ and Peter Bjerregaard^1^
^1^National Institute of Public Health, University of Southern Denmark, Odense, Denmark, idp@niph.dk; ^2^Universite Laval, Centre de recherche du CHU de Quebec, Ville de Québec, Canada

#### 

*Background*. Self-rated health (SRH) has been found to be an indicator of mortality and morbidity in different populations worldwide, and it is widely used as a measure of health status in population health surveys. However, few studies have examined SRH in relation to objectives health measures among indigenous populations in the circumpolar area. The objective of this study is to assess if SRH is associated with objective health measures in Nunavik and Greenland and to identify whether associations differ between men and women. *Methods*. Cross-sectional comparable data among adults (18+ years) from the Nunavik Inuit Health Survey (2004) and Inuit Health in Transition Greenland Study (2005–2010) were analysed. SRH was dichotomized into good health (excellent, very good or good health) versus poor health (fair and poor health). Age-adjusted logistic regression models were used to examine associations between SRH and several clinical health measures: waist circumference, diabetes (impaired fasting glucose), blood pressure and cholesterol. Analyses were stratified by sex and conducted separately for the two surveys. *Results*. In overall regional sample, poor SRH was associated only with impaired fasting glucose among participants from Greenland and with higher waist circumference among participants from Nunavik. In sex-specific analyses, poor SRH was associated with impaired fasting glucose and with higher waist circumference among women in Greenland, but not among men. In Nunavik, poor SRH was associated with higher waist circumference among men only. *Conclusions*. Associations between SRH and objective health measures provided mixed and sex-specific results. More in depth analyses are needed to understand associations between SRH and other health measures with relevance for clinical practice and preventive strategies, and to assess the strength of SRH in predicting mortality among Inuit populations.

## Smart Technology (workshop)

### Tips on how to use telehealth to lead resuscitation remotely

#### Michael Jong
Memorial University, Newfoundland and Labrador, Canada, mjong@warp.nfld.net

##### 

Telehealth has been used for many purposes from chronic disease management to acute care. It has been used successfully for leading resuscitation in remote communities since 2006. This is occurring in at least two countries, Canada and Australia. This small group workshop will provide tips on how to set up videoconferencing on either a 3G cell network or a 256 kb/sec internet. Participants will be able to use the technology via a laptop or I-pad to check out how they may be able to provide this service in their remote settings.

**Reference**

1. Jong M. Resuscitation by video in northern communities. Int J Circumpolar Health. 2010;69:519–27.

## Sexually Transmitted Infections (STIs)

### Incidence of syphilis in Greenland 2010–2014

#### Nadja Albertsen, Michael Lynge Pedersen and Gert Mulvad
Queen Ingrid Health Care Centre, Nuuk, Greenland, naal@peqqik.gl

##### 

*Background*. Sexually transmitted diseases (STDs) have been common in Greenland for decades, with gonorrhea and chlamydia being the most frequent. Also, syphilis was previously a common disease in Greenland with two major epidemics in the 1970s and 1980s. They were followed by a steady decline in incidence and until recently less than one case was reported annually. However, in 2012 and 2013, small epidemics were observed in Western Greenland and the actual incidence and management of syphilis in Greenland is unknown. *Objective.* The aim of this study was to estimate the incidence of syphilis in Greenland from 2010 to 2014 and evaluate sexual contact tracing. *Study design*. This study was performed as an observational retrospective cross-sectional study based on register information and review of the electronic medical record. *Method.* Patients with syphilis were identified from the chief medical officers national register, where all cases of syphilis in Greenland has been registered mandatorily since 2012. In addition, patients treated with either benzathine penicillin, doxycycline or tetracycline between 2010 and 2014 were identified electronically by searching the electronic medical record system. The medical records were reviewed with focus on diagnosis, treatment, contact tracing and follow-up. The population in Greenland as by January 1st 2014 was used as background population. *Results.* Preliminary results indicate an increase in the overall number of cases of syphilis in Greenland from 2010 to 2014. Sexual contact tracing was widely used in the management of syphilis in Greenland and approximately 25% of all new cases were identified this way. *Conclusion.* Further details and conclusions will be presented at the conference.

## Sexually transmitted infections in Greenland – epidemiology and interventions

### Mila Broby Johansen, Bolette Søborg, Anders Koch and Jan Wohlfahrt
Statens Serum Institut, Copenhagen, Denmark, mibj@ssi.dk

#### 

*Objectives.* Greenland carries the highest incidence rates (IRs) of the sexually transmitted infections (STIs) Chlamydia trachomatis and Neisseria gonorrhoea in the Arctic region. This study aims to describe changes in IR for chlamydia and gonorrhoea in Greenland in the period 1990–2012 and to describe if change in surveillance system activities, changes in diagnostic procedures or implementation of national STIs interventions correlates with changes in IR. *Methods.* Data from the national Greenlandic STI surveillance system were used. For 1990–2008, IR was estimated using STI case-data notified as weekly number of cases and population size from Statistics Greenland. For 2009–2012, IR was estimated using individually notified STI cases and individual follow-up from the Civil Registration System. Log-linear Poisson regression was used to estimate IR ratios. Analyses were stratified in three periods (1990–1995, 1996–2005 and 2006–2012) and further divided by sex and age groups. *Results.* Overall, IR for both gonorrhoea and chlamydia increased over the period to reach 2,555 per 100,000 person years (PYs) for gonorrhoea and 6,403 per 100,000 PYs for chlamydia in 2012. The highest IR were observed among young adults aged 15–24 for both diseases; for the period 2006–2012, the IR of this group were approximately 8,000 per 100,000 PYs for gonorrhoea and 22,000 per 100,000 PYs for chlamydia. Different STI interventions have been launched, but only one targeted screening appeared to correlate with decrease in IR. A change in diagnostic test for chlamydia seems to have increased IR, but changes in surveillance system were not correlated with increased IR. *Conclusion.* IR of chlamydia and gonorrhoea increased in Greenland from 1990 to 2012 and are now among the highest in the world. Primary interventions launched have shown little correlation with change in IR, whereas targeted screening and change in diagnostic test appear to correlate with changes in IR.

## Human papillomavirus genotypes among Alaska Native women attending a colposcopy clinic in Anchorage, Alaska, 2009–2011

### Neil Murphy^1^, Tom Hennessey^2^, Lisa Bulkow^2^, Lauri Markowitz^3^, Eileen Dunne^3^, Elizabeth Unger^4^, Elissa Meites^3^ and Martin Steinau^5^
^1^Southcentral Foundation, Anchorage, AK, USA, njmurphy@scf.cc; ^2^Arctic Investigation Program, Anchorage, USA; ^3^Division of STD Prevention, CDC, Atlanta, GA, USA; ^4^HPV Laboratory, CDC, Atlanta GA, USA; ^5^Division of High Consequence Pathogens and Pathology, National Center for Emerging and Zoonotic Infectious Diseases, CDC, Atlanta, GA, USA

#### 

*Background and objective*. Vaccines against HPV genotypes 16 and 18, which may cause 70% of cervical cancer, have been in use since 2006 (1). Recently, the CDC Advisory Committee on Immunization Practices recommended use of a 9-valent HPV vaccine (9vHPV) which protects against HPV genotypes 31, 33, 45, 52 and 58 in addition to 16 and 18 (2). We determined the HPV genotypes infecting women seeking evaluation for abnormal cervical cytology soon after HPV vaccine introduction. *Design*. We began a prospective programme to detect HPV genotypes in Alaska Native (AN) women undergoing colposcopy at the Alaska Native Medical Center. We obtained a cervical brush biopsy tested by polymerase chain reaction using linear array hybridization for 37 genotypes. *Results*. After obtaining informed consent, specimens from 488 AN women were collected. The mean age of participants was 29.7 years. Cytology indications for colposcopy were: high grade squamous intraepithelial lesion 46 (9.4%), low grade squamous intraepithelial lesion 162 (33.2%), atypical squamous cells cannot exclude high-grade squamous intraepithelial lesion 25 (5.1%) and atypical squamous cells of undetermined significance 231 (47.3%). Diagnoses were: cervical intraepithelial neoplasia grade three (CIN 3): 21 (4.3%), CIN 2: 46 (9.4%), CIN 1: 177 (36.3%), metaplasia: 96 (19.7%) and inflammation 42 (8.6%). The mean number of HPV genotypes was 2.5 per person. One HPV genotype was identified in 126 (26%), two HPV genotypes in 126 (26%), three HPV genotypes in 94 (19%), four HPV genotypes reported in 55 (11%) and five HPV genotypes in 31 (6%). HPV 16 was the most commonly identified HPV genotype in 127 (29%), HPV 31 in 64 (13%), HPV 58 in 59 (12%), HPV 52 in 58 (12%), HPV 39 in 52 (11%), HPV 59 in 51 (10%), HPV 45 in 40 (8%) and HPV 18 in 39 (8%). Genotypes 16 or 18 were associated with 17 (77%) of the 22 cases of CIN 3, 24 (53%) of the 45 cases of CIN 2, and 67 (36%) of the 187 cases of CIN 1 (p<0.001). The 9vHPV genotypes were associated with 21 (95%) of CIN 3, 37 (82%) of the cases of CIN 2, and 119 (65%) of the cases of CIN 1 (p<0.001). *Conclusion*. Many AN women will benefit from vaccination with vaccines against genotypes 16/18. More AN women are likely to benefit from use of 9vHPV.These data indicate that use of 9vHPV offers better coverage of the genotypes causing pre-cancerous cervical lesions among AN women.

**References**

1. Quadrivalent Human Papillomavirus Vaccine. Recommendations of the Advisory Committee on Immunization Practices (ACIP). MMWR 2007;56:RR-2.

2. Petrosky E, Bocchini JA Jr., Hariri S, Chesson H, Curtis CR, Saraiya M, et al. Use of 9-valent human papillomavirus (HPV) vaccine: updated HPV vaccination recommendations of the advisory committee on immunization practices. MMWR. 2015;64(11):300–4.

## NATIVE It’s Your Game: adaptation of a computer-based HIV, STD, teen pregnancy prevention programme for American Indian and Alaska Native Youth

### Cornelia Jessen^1^, Jennifer Torres^2^, Christine Markham^2^, Stephanie Craig Rushing^3^, Travis Lane^4^, Amanda Gaston^3^, Gwenda Gorman^4^, Ross Shegog^2^, Taija Revels^1^ and Jennifer Williamson^1^
^1^Alaska Native Tribal Health Consortium, Anchorage, AK, USA, cmjessen@anthc.org; ^2^University of Texas Prevention Research Center, Houston, USA; ^3^Northwest Portland Area Indian Health Board, Portland, OR, USA; ^4^Inter Tribal Council of Arizona, Phoenix, AZ, USA

#### 

Persistent disparities in birth and sexually transmitted infection (STI) rates between American Indian/Alaska Native (AI/AN) youth and other US teens indicate a need for effective HIV/STI/pregnancy prevention for this population. The Alaska Native Tribal Health Consortium HIV/STD Prevention Program partnered with the University of Texas Prevention Research Center, the Northwest Portland Area Indian Health Board and the Inter Tribal Council of Arizona to develop an Internet-accessible middle school sexual health education curriculum for AI/AN youth and to evaluate its acceptability, feasibility and impact. NATIVE It’s Your Game (N-IYG) is comprised of 13 Internet-accessible lessons adapted from an existing urban curriculum, “It’s Your Game Tech.” Pre-adaptation usability testing was conducted with AI/AN youth (n=80) and adult stakeholders (n=18) and post-adaptation usability testing with AI/AN youth (n=45) and adult stakeholders (n=25) within a larger randomized control trial. Parameters for informing cultural adaptations included satisfaction, ease of use, credibility, understandability and motivational appeal which were quantitatively and qualitatively assessed. Percentages below range from the lesson with the lowest rating to the lesson with the highest rating for each parameter. Youth rated N-IYG as culturally acceptable with positive agreement for easy to use (79–100%), paced just right (58–100%), correct and trustworthy (77–100%) and understandable (74–100%). The majority of students liked the lessons (68–94%) and indicated that the lessons would help them make better choices (73–100%). Adult stakeholders rated N-IYG as appropriate and relevant for AI/AN youth. A systematic community-based approach to surface and deep cultural adaptations has the potential to make a preexisting evidence-based sexual health programme more salient for AI/AN youth. N-IYG may help to alleviate the persistent disparities between traditionally underserved AI/AN youth and other US teens.

## Safe in the village: developing a sexual health video program for American Indian/Alaska Native youth in Alaska

### Cornelia Jessen^1^, Taija Revels^1^, David Driscoll^2^, Janet Johnston^2^ and Sarah Shimer^2^
^1^Alaska Native Tribal Health Consortium, Anchorage, AK, USA, cmjessen@anthc.org; ^2^University of Alaska Anchorage, Anchorage, AK, USA

#### 

American Indian/Alaska Native (AI/AN) youth ages 15–24 in rural Alaska communities experience high rates of sexually transmitted infections (STIs) and have limited access to sexual health programmes, disparities that they share with indigenous youth across the circumpolar north. The Alaska Native Tribal Health Consortium HIV/STD Prevention Program partnered with the University of Alaska Anchorage Institute for Circumpolar Health Studies to address the need for culturally and age appropriate sexual health programmes for this population. The outcome is safe in the village (SITV), a new healthy relationships and safe behaviours video programme. SITV was developed through formative data collection with AI/AN youth (n=97) in rural Alaska communities (n=5) using in-depth interviews and Likert scale surveys to understand perceptions, attitudes and knowledge of HIV/STIs and healthy relationships and associated risk and protective behaviours and factors. In-depth interviews were transcribed and analysed using a grounded theory approach to identify common themes. Surveys were developed based on a cultural consensus approach to ascertain the degree of agreement among participants on key themes. Researchers actively engaged communities by obtaining community support and tribal approvals, employing local site coordinators and involving a community advisory committee to provide guidance and input during the programme development. Sexual health and healthy relationship messages targeted at AI/AN youth need to incorporate traditional values and culturally appropriate conflict resolution while emphasizing protective factors and framing STIs and sex in the context of alcohol abuse and intimate partner violence. A qualitative approach grounded in active community engagement is crucial to developing culturally appropriate and relevant sexual health programmes for AI/AN youth residing in remote Native communities. The SITV programme may also have relevance in circumpolar communities outside Alaska.

## HPV variants in Inuit women from Nunavik, Quebec

### Barbara Gauthier^1^, Francois Coutlée^2^, Eduardo Franco^3^ and Paul Brassard^1^
^1^Department of Epidemiology, Biostatistics, and Occupational Health, McGill University, Montreal, Canada, paul.brassard@mcgill.ca; ^2^Département de Microbiologie et Infectiologie, Centre Hospitalier de l’Université de Montréal, Montreal, Canada; ^3^Division of Cancer Epidemiology, McGill University, Montreal, Canada

#### 

*Background*. Inuit communities in Northern Quebec have been shown to have high rates of human papillomavirus (HPV) infection, cervical cancer and cervical cancer-related mortality as compared to the Canadian population. High risk (HR) HPV can be further classified as intratypic variants which may differ in risk of cervical lesions or neoplasia. There is limited information on which variants are found in circumpolar areas. *Methods*. 629 Inuit women (aged 15–69) from Nunavik had HPV DNA collected along with Pap smear samples between 2002 and 2010 and sequenced to determine the intratypic variant. The different variants and lineages present were described and compared to similar circumpolar populations. *Results*. There were 174 women who were positive for HPV – 16, 18, 31, 33, 35, 45, 52, 56 or 58 during follow-up. There were five different variants, all of which were of European lineage, amongst the 57 women positive for HPV-16. The majority (n=31, 54%) were prototypic. There were eight different variants HPV-18 present. All were non-prototypic and of European lineage (n=21). There was one woman who tested positive for a prototypic lineage A variant of HPV-31, and 52 women (96%) were from lineage B and one from lineage C (2%). Only one prototypic, lineage A1, HPV-45 variant was present. The rest (n=16) were from lineage B. All women HPV-52 variants (n=23) were in lineage A and 39% were prototypic. One prototype sample was seen for HPV-56 with the majority in lineage A (n=14, 82%). All HPV-58 samples were in lineage A (n=40), and none were prototypic. *Conclusion*. These trends are similar to what was seen in other circumpolar regions of Canada, although there appears to be less diversity as no non-European variants of HPV-16 or HPV-18 were present. This study shows that most variants were clustered in one lineage for each HPV type. We are currently exploring associations of HR-HPV variant with infection persistence.

## Rapid change in the ciprofloxacin-resistance pattern among *Neisseria gonorrhoeae* strains in Nuuk, Greenland

### Anne Rolskov^1^, Karen Bjørn-Mortensen^2^, Gert Mulvad^3^, Peter Poulsen^4^, Jørn Skov Jensen^5^ and Michael Lynge Pedersen^3^
^1^Department of Gynaecology and Obstetrics, Hillerød Hospital, Hillerød, Denmark, asrolskov@gmail.com; ^2^Department of Epidemiology Research, Statens Serum Institut, Copenhagen, Denmark; ^3^Queen Ingrid Health Care Center and Greenland Center for Health Research, Institute of Nursing and Health, University of Greenland, Nuuk, Greenland; ^4^The Central Laboratory, Queen Ingrid Hospital, Nuuk, Greenland; ^5^Microbiology and Infection Control, Statens Serum Institut, Copenhagen, Denmark

#### 

*Objectives*. Sexually transmitted infections (STIs), including infections with *Neisseria gonorrhoeae* (GC), are highly incident in Greenland. Since January 2011, GC testing has been performed on urine with nucleic acid amplification tests (NAATs) by stand displacement amplification (Becton Dickinson ProbeTec). Monitoring of GC antibiotic susceptibility by culture was introduced in Nuuk in 2012. Until 2014, no cases of ciprofloxacin-resistant GC strains were reported. In this paper, we report the finding of ciprofloxacin-resistant GC and describe the most recent incidence of GC infections in Greenland. *Methods*. Men in Nuuk with a positive urine test (NAAT) for GC have been encouraged to provide a urethral swab for culture and susceptibility testing. The number of urine NAATs and culture positive swabs from January to October 2014 were obtained from the Central Laboratory at Queens Ingrid’s Hospital in Nuuk and stratified on gender, place and period of testing. Incidence rates were estimated as number of urine NAAT * (12/10) per 100,000 inhabitants. *Results*. From January to October 2014, a total of 5,436 urine GC NAATs were performed in Nuuk and 9,031 in the rest of Greenland. Of these, 422 (8%) and 820 (9%) were positive, respectively. From January to August, 6 (15%) cultures from Nuuk were ciprofloxacin resistant while in September and October 26 (59%) were resistant (p<0.01). In total, 35 (40%) of 88 culture positive isolates showed ciprofloxacin resistance. GC incidence in Nuuk was 3,017 per 100,000 inhabitants per year, while in the rest of Greenland 2,491 per 100,000 inhabitants/year.

*Conclusion*. Within a short period, a rapid and dramatic change in ciprofloxacin susceptibility among GC strains isolated in Nuuk was documented and recommendation for first line treatments has changed. Continued monitoring and re-thinking of primary and secondary preventive initiatives is highly recommended in this high GC incidence setting.

**References**

1. Pedersen ML, Clausen-Dichow P, Poulsen P, Nyborg H, Jensen JS. Low prevalence of ciprofloxacin-resistant Neisseria gonorrhoeae in Nuuk, Greenland. Sex Transm Dis. 2013;8:639–40.

2. Statistics Greenland. [cited 2014 Nov 13]. Available at http://www.stat.gl/

3. Bignell C, Unemo M. European STI Guidelines Editorial Board. Int J STD AIDS. 2013;2:85–92.

## Friday July 12th 2015

### Plenary Session

#### Challenges for health and well-being in the Arctic: local capacity building strengthens the Arctic collaboration

##### Gert Mulvad
University of Greenland, Nuuk, Greenland, gm@peqqik.gl

###### 

Polar Scientist, Knud Rasmussen travelled by dogsled from Greenland via Hudson Bay Canada to the Bering Strait in Alaska and Siberia. “Give me winter, give me dogs – you can keep the rest.” He was an important player in a study of diet and metabolism of Eskimos at the Arctic Station in Greenland in 1908 led by the Nobel Prize winner August Krogh. The past open our eyes for future challenges in the Arctic. There is an increasing demand for health services in all the areas, especially in mental health. The social determinants of health (e.g. housing, education, and employment) have the most significant impact on health outcomes. These challenges will require more emphasis on research and education in order to set proper priorities. I will focus on some statement and lessons learnt from the Arctic collaboration and the local capacity building. The most important ones are the Arctic Health Declaration (Health ministerial meeting in Nuuk 2013), which emphasizes the important tools of proactive health promotion and capacity building in addressing challenges in the Arctic communities, and the ICC Declaration which recognizes that too few Inuits are working as MDs and nurses and the importance of a new project to strengthen the local perspective.

## Development of Health Care II

### Family care where a family member is chronically ill with Parkinson’s disease – a qualitative study

#### Lise Hounsgaard^1,2^
^1^Institute of Nursing and Health Science, University of Greenland, Nuuk, Greenland; ^2^OPEN, Institute of Clinical Research, University of Southern Denmark, Odense, Denmark, Lhounsgaard@health.sdu.dk

##### 

*Background*. A recent health care reform in Greenland demands quality health care improvements in health promotion and family nursing. The project investigates how the effects of this reform are revealed in the everyday life of families in which a member has Parkinson’s disease. Family members are greatly involved in informal care-giving, because the disease progression has impacts on motor and cognitive functioning. *Objectives*. The aim was to raise awareness of the relatives’ situation and to create new nursing knowledge about care-giving and support to families that include a chronically ill family member. *Materials and methods*. A phenomenological hermeneutic approach was taken. The study comprises individual and focus group interviews with relatives living in the capital, Nuuk. Individual interviews were undertaken in 2014 with partners and grown-up children. In the spring of 2015, a further focus group interviews will be carried out. A critical interpretation using phenomenological-hermeneutic textual analysis is ongoing. *Results*. Preliminary results provide insight into the fact that relatives lack knowledge of the development of the illness and the steadily increasing burden to which the disease progression gives rise in everyday life. The results are likely to help to identify what information relatives should be offered to broaden their knowledge and enable them to take charge of their everyday lives. Broadly, the project is expected to contribute to raising awareness of the situation of relatives in health promotion and prevention work.

**References**

1. Hounsgaard L, Pedersen BD, Wagner L. The daily living for informal caregivers with a partner with Parkinson’s disease – an interview study of women′s experiences of care decisions and selfmanagement. J Nurs Healthc Chronic Illn. 2011;3:504–12.

2. Hounsgaard L, Jensen AB, Præst Wilche J. The nature of nursing practice in rural and remote areas of Greenland. Int J Circumpolar Health. 2013;72:20964.

## Follow-up of cancer incidence among Finnish Sami 1979–2010

### Leena Soininen^1^ and Eero Pukkala^2^
^1^University of Helsinki, Helsinki, Finland, leena.soininen@fimnet.fi; ^2^Finnish Cancer Registry, Helsinki, Finland

#### 

The Sami are regarded as indigenous people with traditional dwelling zones in northern parts of Norway, Sweden, Finland and northwest Russia. In Finland, there are three Sami subgroups: North Sami, Inari Sami and Skolt Sami. All persons living in the two northern municipalities of Finland (Inari and Utsjoki) on 31 December 1978 were identified from the National Population Register (N Sami = 2,651). The Sami cohort was extracted from that and grouped to the subgroups of Sami. Follow-up for cancer through the Finnish Cancer Registry for periods 1979–1998 and 1999–2010 was done, standardized incidence ratios (SIRs), using the entire Finnish population as reference. The SIRs for all sites cancer for the Finnish Sami during the two time periods were 0.64 (95% confidence interval (CI) 0.53–0.76) and 0.76 (95% CI 0.62–0.93), respectively. During the first time period, the SIR for North Sami was 0.60 (0.44–0.80), for the Inari Sami 0.48 (0.32–0.70) and for the Skolt Sami 1.01 (0.68–1.46). The corresponding SIRs during the second time period were 0.77 (0.57–1.02), 0.71 (0.46–1.05) and 0.88 (0.53–1.38). The SIR of stomach cancer among Skolt Sami in 1979–1998 was high (4.00, 1.61–8.24), but decreased to 1.67 (0.04–9.20) in the latter periods. Common cancers among the Finnish main population such as cancers of the prostate, breast and skin are still rarer among the Finnish Sami. For example, the SIRs of cancer of prostate for the two time periods for the Sami were 0.25 (0.08–0.59) and 0.38 (0.17–0.72). Among the non-Sami on the same area, the incidence of prostate cancer was about the same as among the general population of Finland. The SIRs for the breast cancer for all Sami groups were 0.37 (0.15–0.76) and 0.40 (0.16–0.81). It seems that incidence of most cancers among the Finnish Sami is approaching that of the general population.

**References**

1. Soininen L, Järvinen S, Pukkala E. Cancer incidence among Sami in Northern Finland, 1979–1998. Int J Cancer. 2002;100:342–6.

2. Kurttio P, Pukkala E, Ilus T, Rahola T, Auvinen A. Radiation doses from global fallout and cancer incidence among reindeer herders and Sami in Northern Finland. Occup Environ Med. 2010;67:737–43.

## The burden of cancer on Alaska Native people: comprehensive cancer planning and programs within the Alaska Tribal Health System

### Stacy Kelley, Christine DeCourtney, Judith Muller and Karen Morgan
Alaska Native Tribal Health Consortium, Anchorage, AK, USA, sfkelley@anthc.org

#### 

Cancer is the leading cause of death in Alaska Native people. Cancer tumor registry data is available from 1969. Prior to developing the first comprehensive cancer program, few programs and services, other than treatment, were available to patients diagnosed with cancer. The word cancer was feared and misunderstood by Alaska Native people. Aside from tobacco control, there was little focus and few resources devoted to prevention and screening. There were no survivorship or palliative care programs. Development of a community and provider inclusive cancer plan included 150 people and 18 months of hard work. In a state with few resources, developing a positive, ongoing relationship with the State of Alaska cancer program and other partners was important. Finding funders to support plan implementation projects was critical for success. Bringing awareness about continuum of care gaps was needed to improve capacity as well as community and provider education in all parts of the cancer continuum. Since the implementation of the first comprehensive cancer plan in 2004, colorectal cancer screening has improved significantly through improved capacity, partnerships and additional funding sources. Traditional foods nutrition materials for adults and children have been very successful and used throughout the state. Palliative care programs have been developed to allow people to remain at home and enhanced through telehealth. Unique survivorship resources help address survivorship care gaps. Children who have lost a loved one to cancer can attend a special camp. Over 100 peer-reviewed manuscripts were published. A 5-year plan progress report found successes and challenges. There is still much work to be done to reduce the cancer burden in our efforts to make the Alaska Native people the healthiest people in the world. The 2011–2017 Alaska Tribal Health System Cancer Plan update addresses the continuing challenges.

## Developing a community based research agenda with lesbian, gay, bisexual, transgender and queer youth in the Northwest Territories, Canada

### Carmen Logie and Candice Lys
University of Toronto, Toronto, Canada, carmen.logie@utoronto.ca

#### 

*Background*. Mental and sexual health disparities among youth in Canada’s North are a pressing concern. Stigma and discrimination targeting lesbian, gay, bisexual, transgender and queer (LGBTQ) youth are pervasive global phenomena that have devastating impacts on health and well-being. Yet there is little understanding of how stigma is experienced among LGBTQ youth in the Northwest Territories (NWT). *Objectives*. Our primary objective was to explore and identify LGBTQ youth health research priorities in the NWT. The goal was to develop a LGBTQ youth focused community based program of research in the NWT. *Methods*. We conducted formative research to define our research agenda. We held a meeting with college faculty, students and community agencies; a focus group with LGBTQ youth (n = 10) during NWT Pride; consultations with an arts-based youth sexual health program; and a key stakeholder meeting (n = 15). *Findings*. Findings revealed interest and willingness of health, education and social programs to address LGBTQ issues in the NWT. LGBTQ youth discussed community norms that devalued same sex identities, and stigma surrounding LGBTQ specific services and agencies. Yet there were few instances of violence and enacted stigma due to close knit and small communities. Stigma was exacerbated for LGBTQ youth in secondary school and gender non-conforming youth. While recent activism (e.g. NWT Pride) generated media and community discussion of LGBTQ issues, many youth discussed heteronormativity and invisibility in school and health care. Service providers discussed the importance of integrating LGBT issues in youth programs to enhance accessibility, particularly in rural regions. *Conclusions*. Findings reveal opportunities to provide LGBTQ specific services, but also the need to integrate sexual and gender diversity into health, education and social programs. Future research should explore the intersections of sexuality, ethnicity, gender and rural/urban identities in the NWT.

## Alaska Native breast cancer patients:a care coordination quality improvement project

### Stacy Kelley, Gretchen Day, Christine DeCourtney and Karen Morgan
Alaska Native Tribal Health Consortium, Anchorage, AK, USA, sfkelley@anthc.org

#### 

Breast cancer is the leading cancer among Alaska Native women. When an Alaska Native woman in a village is suspected of having breast cancer, a long journey begins. She must travel by airplane for a screening mammogram, and then again for further tests, diagnosis and treatment. Travel co-ordination, tribal policies, treatment guidelines, cultural barriers and provider communication create a potential for delays and gaps in the cancer care continuum and all impact diagnosis and timely treatment. To address breast cancer disparities within the Alaska Tribal Healthcare System (ATHS) a collaborative quality improvement study was conducted to identify variances and gaps in the cancer care continuum. We analysed SEER Tumor Registry Data for Alaska Native women at point of breast cancer diagnosis to treatment for years 2009-2014 (N=284) by residence, age, stage of cancer diagnosis and treatment type. We calculated adjusted proportions with logistic regression to examine diagnosis and treatment within program benchmarks (<60 days). We also identified data measures and gaps within the Electronic Health Record and variances within clinical protocols and processes throughout the ATHS through in depth provider and administrator interviews. Median diagnosis date to time of initial treatment for breast cancer was 26 days. No significant rural versus urban differences were found. Additional findings related to the breast cancer data analysis will be presented. Qualitative results of interviews as well as process evaluation results will be presented. This retrospective population based data analysis suggests that most breast cancer patients are receiving timely care regardless of home community, age and stage of cancer. Treatment for breast cancer patients is well within the established US National core performance indicators and standards. Strengths and challenges of the ATHS will provide recommendations for different models for coordinating care.

## Smart Technology

### Narrowing the gap of health care inequality in the north by using remote presence robotic technology

#### Ivar Mendez^1^, Tanya Holt^1^, Veronica McKinney^1^ and Debra Keays-White^2^
^1^University of Saskatchewan, Saskatoon, Canada, ivar.mendez@usask.ca; ^2^Health Canada, Halifax, Canada

##### 

Disparities in the provision of health care services to Canadians who live in the north have a major negative impact on their health and life expectancy. Aboriginal communities in the North have an average life expectancy significantly lower than the rest of Canadians. Barriers of distance, lack of adequate health care infrastructure and expertise limit the provision of health care to vulnerable populations such as children and the elderly. The expansion in telecommunication technology has opened the door for solutions that may help address these challenges. We have pioneered the use of remote presence robotic technology in the Canadian North by using robots and portable devices capable of real-time connectivity using available cell phone networks or Wi-Fi link. After the first deployment of a telepresence robot “Rosie” in Nain, Labrador, the program has expanded with a second generation robot to the northern Saskatchewan Aboriginal community of Pelican Narrows. The first priority in Pelican Narrows has been to provide pediatric care as it was deemed to be an urgent need by the community. Our approach to implementation of this technology has been inclusive and multidisciplinary. It involved the active participation of the Aboriginal community, their leaders and the local health care workers before the robot was deployed. Specialists in pediatric emergency, intensive care and surgery provide remote presence medical service to Pelican Narrows. Our experience in managing pediatric emergencies, triaging of patients, providing elective and follow up care has been so far positive. The impact of this technology on health outcomes, patient transportation, nurse’s support and patient satisfaction will be discussed. This project strongly suggests that the use of remote presence robotic technology using a multidisciplinary and culturally inclusive approach has the potential to empower remote Aboriginal communities to narrow the gap of inequality in health care delivery.

**References**

1. Mendez I, et al. The use of remote presence for health care delivery in a Northern Inuit community: a feasibility study Int J Circumpolar Health. 2013;72:21112.

2. Mendez I, Van den Hof M. Mobile remote-presence devices for point-of-care health care delivery. CMAJ. 2013;185:120223.

3. Mendez I, et al. Point of-care programming for neuromodulation: a feasibility study using remote presence. Neurosurgery. 2013;72:99–108.

## Yukon Baby: a community-based mobile app for pregnant women, their partners and families

### Shannon Ryan^1^, Christopher Naylor^2^, Wes Wilson^3^, Kate Swales^4^ and Kathleen Cranfield^5^
^1^Yukon Government, Whitehorse, Canada, shannon.ryan@gov.yk.ca; ^2^Crocus Maternity Clinic, Whitehorse, Canada; ^3^University of British Columbia, British Columbia, Canada; ^4^Yukon College, Whitehorse, Canada; ^5^Community Midwives Association Yukon, Whitehorse, Canada

#### 

Thirty six percent of Yukon’s preschool children are vulnerable in one or more areas of early development (1). The prenatal, post-natal and early childhood stages present an opportunity to significantly impact future health and development. However, providing access to consistent and locally relevant health information is difficult in Yukon due to the geography, diversity of providers, frequent staff turnover and changing health information. In addition, young women are frequent users of Internet technology; they want short, quick answers to their pregnancy and child rearing concerns and prefer information readily accessible on mobile devices (2, p. 6). The Yukon Baby smartphone app will engage women, men and their families and support them during pre-pregnancy, pregnancy and early childhood periods. Although many pregnancy and childhood-based apps exist, none are locally relevant and tailored to our northern communities. The app will increase awareness of evidence-based healthy practices such as folic acid intake and vaccination visits to ultimately improve pregnancy and childhood outcomes in Yukon, which in turn will reduce health system costs. The app will provide easy access to local resources, relevant health information and tools to support health. Tools will include a pregnancy timeline, community events calendar, frequently asked questions, and ways to track the health of their pregnancy, newborn and young children (immunizations, height/weight, milestones, depression screen and others). Additional components will add to their experience including a kick counter, contraction timer, birth plan and “bump” photos. The project is being designed so that the content could easily be customized for use in other communities in Canada and internationally.

**References**

1. Canadian Institute for Health Information (CIHI). Children vulnerable in areas of early development details for Yukon. [cited 2015 Jan 23]. Available from: http://yourhealthsystem.cihi.ca/indepth?lang=en#/indicator/013/2/C99003/

2. Hearn L, Miller M, Lester L. Reaching perinatal women online: the Healthy You, Healthy Baby website and app. J Obes. 2014;2014:1–9. doi: http://dx.doi.org/10.1155/2014/573928

## Inuit youth as health change advocates: engaging community through the use of technology and new media

### Taha Tabish^1^ and Shirley Tagalik^2^
^1^Qaujigiartiit Health Research Centre, Iqaluit, Canada; ^2^Arviat Wellness Centre, Kugaaruk, Canada, inukpaujaq@gmail.com

#### 

Inuit jurisdiction demographics indicate a large youth population that is experiencing several health and well-being challenges (1,2). In Arviat, through community-initiated strategies of engaging youth in the development of healthier messaging around key issues, youth are becoming the advocates for improved life outcomes. Using Inuit Qaujimajatuqangit (traditional knowledge) strength-building processes such as pilimmaksarniq/mastering skills, youth in Arviat are becoming effective researchers, knowledge interpreters, media designers and message carriers to their community and beyond. In this presentation, we will highlight the path this work has taken over a period of 5 years, with a specific look at the Atii, Let’s Do It! project for healthy nutrition and active living. Through this project, we will identify the linkages to community expertise, resources and supports, the impacts the work has had on youth engagement and leadership, community cohesion and health awareness. This work has benefitted from partnerships with several Qaujigiartiit Health Research Centre initiatives, so that collaboratively, using the foundation of piliriqatigiingniq/working for a common purpose, youth generated messaging and programs are reaching a broad audience of Inuit youth. This work is critically supported by training opportunities in design and technologies. The presentation will feature youth-produced examples of video messaging, music, storybooks, game app development and productions for television as a way of illustrating the impacts of youth advocacy.

**References**

1. NTI. 2010–11 Annual report on the state of Inuit culture & society: the status of Inuit children and youth in Nunavut. Iqaluit, NU: Nunavut Tunngavik Incorporated; 2011.

2. Egeland GM. Qanuippitali?: The International Polar Year Nunavut Inuit Child. Ottawa, Canada: Health Canada; 2009.

3. Health Survey, 2007–2008. Ste-Anne-de-Bellevue, QC: the Canadian Federal Program for International Polar Year; 2009.

## Creating a comprehensive picture of research in the circumpolar Arctic

### Christy Garrett
Alaska Medical Library, University of Alaska, Anchorage, AK, USA, cgarrett@uaa.alaska.edu

#### 

Research is a key component of good evidence based public health; knowledge of what research is being done can make for a more informed process. Toward this end the National Library of Medicine (NLM), in conjunction with the Alaska Medical Library has begun a project to ultimately establish data sharing partnerships, secure depositories and create a comprehensive record of research in Alaska and eventually throughout the circumpolar Arctic. As part of the project NLM will be seeking collaborations with organizations, researchers and community representatives in order to create an easily accessible database where researchers, public health professionals and others can go to see what type of research is being done, by whom and where, to facilitate wider sharing and collaboration, eventually creating a more comprehensive picture of the health issues being studied in the Arctic. Additionally, part of this profile will refer where the data and reports are held and whether access might be possible. It is hoped that these collaborations can eventually lead to discussion about data depositories for projects dealing with circumpolar Arctic human health issues with a central research record that is as comprehensive as possible while still respecting the populations involved. Ultimately, the hope is to create a forum for the discussion of where data resides, how it’s stored and if it can be accessed, by whom, and whether a centralized depository for the data from projects around the Arctic might be possible. In year 1, the focus is on researchers and research conducted in Alaska. In future years, the projects will expand to the circumpolar Arctic research community. The researcher profiles will be made available through the Arctic Health Website, a project of NLM and the Alaska Medical Library. This presentation will introduce the project, setting the stage for the future goal of collaborations with researchers throughout the Arctic.

## Are we there yet? What’s freely available on the internet for circumpolar health researchers?

### Janice Linton
University of Manitoba, Winnipeg, Canada, janice.linton@umanitoba.ca

#### 

Circumpolar health research is international, interdisciplinary, distinctive and often based on collaborative partnerships between community, government and academic stakeholders. A search of the interdisciplinary scholarly or peer-reviewed literature was carried out on the topic of human health research in the circumpolar regions, 2004–2014. Analysis was carried out to identify publishing trends for research outputs, with a focus on determining the accessibility of the research to stakeholders outside the academic community. In recent years, funding agencies in several countries have established requirements for researchers to publish results in open access venues freely accessible on the Internet. This study was carried out to see how well this community has been doing in knowledge dissemination. The result of this examination of the literature confirms that the circumpolar research community is meeting the challenge of disseminating research findings accessible to stakeholders and communities as well as to other communities who can benefit from the leading-edge research being carried out and the dissemination of indigenous knowledge. The results of this study can be used by seasoned researchers along with practitioners, policy makers, and those new to Arctic research, to identify where to access published research for free, efficiently and effectively, and which journals provide the most fruitful knowledge dissemination. A guide to circumpolar health research is available at http://libguides.lib.umanitoba.ca/circumpolar.

## Living in the Arctic

### Population ageing in the Arctic: intra-regional variations and the differences with national rates

#### Anastasia Emelyanova and Arja Rautio
University of Oulu, Oulu, Finland, anastasia.emelyanova@oulu.fi

##### 

The health profile of Arctic populations (morbidity, life-style behaviors, health care arrangements, etc.) affects, in many ways, demographic variables and vice versa. It is therefore important to access the well-being of populations in the Arctic from a point of view of significant demographic changes, where regional demographic face alters, apparently “ageing.” Population ageing is the subject of analysis in my interdisciplinary doctoral thesis. This process in the Arctic inhabited with a lot of small populated communities has the power to transform those across all layers and can profoundly distort the age composition of regional populations. We argue in the study that population ageing observed within remote Arctic territories differs, sometimes significantly, from the “mainland” (e.g. Greenland vs. Denmark, Nunavut vs. Canada). It is therefore important producing evidence on ageing at a sub-national level defining ageing variations in the context of demographic, social, economic and political history of each Arctic territory. With this, population related policies can be adjusted and perhaps modified from general national courses. The methods incorporate traditional United Nations promoted indicators on ageing as well as recently invented “prospective” measures (Sanderson & Scherbov 2008). The latter take into account changes in people’s characteristics beyond standard chronological age, for example, changes in remaining life expectancy, health and morbidity, disability, and cognitive functioning (looking in our study predominantly at “prospective” age based on remaining life expectancies). During the ICCH16 Congress, I would like to present the results of my doctoral dissertation, comparing the ageing development over recent decades between Arctic territories from the North-Atlantic, North-American, North-Russian, and the Barents Euro-Arctic regions, in comparison to each other and national averages of the countries to which these territories belong.

**References**

1. Emelyanova A, Rautio A. Ageing population in the Barents Euro-Arctic region. J Eur Geriatr Med. 2012;3:167–73.

2. Emelyanova A, Rautio A. Perspectives for population ageing in the Russian North. J Popul Ageing. 2013;6:161–87.

3. Sanderson W, Scherbov S. Rethinking age and ageing. Popul Bull. 2008;63:1–20.

## More than shelter: housing as a determinant of health in two northern Canadian Dene communities

### Linda Larcombe^1^, Matthew Singer^1^, Lancelot Coar^1^, Lizette Denechezhe^2^, Evan Yassie^3^, Kathi Avery-Kinew^4^ and Pamela Orr^1^
^1^University of Manitoba, Winnipeg, Canada, porr@hsc.mb.ca; ^2^Northlands Denesuline First Nation, Lac Brochet, Canada; ^3^Sayisi Dene First Nation, northern Manitoba, Canada; ^4^Assembly of Manitoba Chiefs, Winnipeg, Canada

#### 

This study was undertaken as part of a multi-year community based research partnership exploring the biologic and social determinants of health in two Dene First Nations northern Canadian communities. These communities have experienced high rates of tuberculosis for many years. We report the results that focus on the relationship between housing and health, and discuss the implications within the context of indigenous rights. There are no Dene words to say “there is no room” in any place of shelter, reflecting traditional practices of sharing. However, an initial survey identified significant deficiencies in the quantity (leading to crowding), as well as in the quality of current housing – particularly inadequate ventilation, inadequate outdoor water drainage systems, excess indoor humidity and overgrowth of mold (fungi). Community workshops and in depth interviews with youth and adults of all ages also identified the need for culturally appropriate housing design. In particular, community members expressed the need for the following: specific areas within houses to process and store country food; wood stoves as a “back-up” to more recently installed heating furnaces; a mix of private and public space within houses that promotes the mental health of family members; accommodation of disabled individuals. The research findings have been used to advocate with government agencies for improved housing. The project is currently exploring culturally appropriate housing design through a youth exchange program between the Dene and academic communities.

## Health and housing among indigenous people in northern Canada

### Carol Kauppi^1^, Jessica Hein^2^, Amanda McLeod^1^, Henri Pallard^1^
^1^Laurentian University, Sudbury, Canada, ckauppi@laurentian.ca; ^2^University of Toronto Scarborough, Toronto, Canada

#### 

This paper focuses on the health impacts of housing within remote indigenous communities in a subarctic region of Canada—the Western James Bay lowlands. According to UN Special Rapporteur on the Rights of Indigenous Peoples James Anaya (1), the housing circumstances of Inuit and First Nations communities constitute a crisis. He notes that their housing situation contributes to health challenges, particularly in northern communities. This project utilized multiple methods to study housing and health in two Cree communities. The comparison of two remote indigenous communities in the Canadian north make this study unique. In the town of Moosonee, where 85% of the residents are indigenous, a community survey was conducted in 2012 people, as well as a digital storytelling project in which participants narrated issues relating to housing and health. In Fort Albany First Nation, a remote fly-in community on the James Bay coast, a photovoice project was conducted in 2012 in which residents took photographs of their housing situation and explained the impacts on their health. In the results, we identify (i) common themes from the survey data and from photographs and narratives, allowing for a better understanding of the negative impacts of poor housing on physical and mental health; and (ii) common issues such as “couch surfing” and overcrowding, sleeping rough and substandard or inadequate housing. Furthermore, (iii) project’s design enables us to compare the housing circumstances of Cree people living in James Bay communities with those who have migrated from the James Bay to an urban centre in the near north. The findings from this project are similar to those from medical studies on the effects of poverty and housing challenges on health. The implications of the findings will be discussed in terms of the health and housing policies of the Canadian federal government, responsible for funding housing and health in First Nations.

**Reference**

1. UN Human Rights Council.Report of the Special Rapporteur on the Rights Indigenous Peoples, James Anaya. Addendum: The Situation of Indigenous Peoples in Canada; 2014, A/HRC/27/52/Add.2. [cited 2015 Jan 9]. Available from: http://daccess-dds-ny.un.org/doc/UNDOC/GEN/G14/075/08/PDF/G1407508.pdf

## Housing interventions in the Arctic: baseline results of a study assessing the impacts of moving to a new house for Inuit health and well-being

### Mylene Riva
Centre de recherche du CHU de Quebec, Universite Laval, Quebec City, Canada, mylene.riva@crchuq.ulaval.ca

#### 

Across the circumpolar north, a large proportion of Inuit households live in overcrowded conditions. This situation is compromising people’s health and communities’ capacity for social and economic development. Studies have shown that moving to a new house improves health directly and indirectly through psychosocial pathways. To date few studies have assessed the impacts of housing interventions for health and well-being in the Arctic. Set in Nunavik and Nunavut, this project aims to examine whether moving to a new house – by reducing exposure to overcrowding and improving housing quality – is associated with improved health, and to assess the mediating role of psychosocial factors. Adults in single-person and family households on the waitlist for social housing were recruited to the study. Baseline data were collected 4–6 weeks before people moved into a new house in six communities in Nunavik (October–November 2014), and in three communities in Nunavut (May 2015). Follow-up data collection will be conducted 18 months after moving house. Results of associations between housing conditions (objective and perceived crowding, housing quality), physical and mental health and psychosocial factors (control, identity, satisfaction, privacy, safety) will be presented for a sample of about 130 participants in Nunavik. Baseline results will provide novel evidence pertaining to health and psychosocial factors associated with housing conditions in the Arctic. Overall, results produced by this project have the potential to be integrated in the formulation of housing strategies in Nunavik and Nunavut. Partners: Kativik Municipal Housing Bureau; Nunavik Regional Board of Health and Social Services; Kativik Regional Government; Société d’Habitation du Québec; Nunavut Housing Corporation; Government of Nunavut Departement of Health; Nunavut Tunngavik Inc.

## Flood hazard in Kashechewan First Nation: an environmental justice perspective

### Jessica Hein^1^, Carol Kauppi^2^, Arshi Shaikh^3^, Henri Pallard^2^ and Amanda McLeod^2^
^1^University of Toronto Scarborough, Toronto, Canada; ^2^Laurentian University, Sudbury, Canada, phm_admin@laurentian.ca; ^3^Renison University College, Waterloo, Canada

#### 

A key aspect of environmental health for many indigenous communities of the Canadian north pertains to floods and water damage to homes and communities. Floods are costly in terms of damage to property and infrastructure as well as adverse social, psychological and health impacts. Many First Nations face annual flooding and evacuations due to the geographic location of their communities. Kashechewan First Nation, situated in the James Bay lowlands in northern Ontario, has experienced annual flooding over the past several years. This presentation explores the intersecting issues of flooding and displacement in Kashechewan from an environmental justice perspective and considers sustainable, viable long-term policy options to break the annual cycle of flood hazard and its consequences. The environmental justice framework remains largely underutilized for policy development in Canada and this project addressed this knowledge gap. We explored the historical, colonial processes associated with the flooding, the magnitude of the flood hazard, current flood hazard management policies and programs operating in Kashechewan and policy solutions devised by different jurisdictions within Canada. The environmental justice lens revealed an undercurrent of ongoing environmental colonialism, oppression, and racism embedded in the floods encountered by Kashechewan First Nation. In examining contextual factors and policy solutions, we developed viable long-term policy alternatives for flood hazard mitigation by utilizing the environmental justice framework. We recommend flood hazard mitigation policy initiatives that can be integrated with civic engagement and community capacity building, and potentially with broader regional development in the western James Bay area. Although the paper focuses on the localized issue of floods, evacuation and displacement for Kashechewan, the recommendations may offer solutions for other First Nations facing similar issues.

## Food security in Alaska: definitions of “urban” and “rural” make a difference

### Tracey Burke
University of Alaska Anchorage, Anchorage, AK, USA, tkburke@uaa.alaska.edu

#### 

Research on food security provides a case study of the ambiguity of the terms rural and urban when referring to some areas of Alaska and Canada. This presentation draws on a qualitative study conducted with urban and rural users of Alaskan food pantries to illustrate how different types of communities are associated with different discourses of food security and possible interventions. The project is part of a long-standing collaboration between the principal investigator (PI) and the state’s largest hunger-assistance NGO. Academic and colloquial references to “rural Alaska” often refer to the very-remote, mostly Alaska Native communities found off the road and ferry systems. Yet, Alaska has numerous communities on the road and ferry systems, the residents of which are typically non-Native, and very few of which are conventionally “urban.” There is no sanctioned typology of Alaskan communities; to conduct our study, we developed our own. It is based on the U.S. Department of Agriculture’s “frontier and remote” codes (1) and uses two dimensions – population size and order of services available and remoteness. Alaska’s three discourses of food security map onto the typology easily. The remoteness dimension reflects favored local foods. The discourse of food security in remote Alaska focuses on subsistence hunting/fishing. In more accessible communities, the discourse of “locally grown” produce gains prominence. The size/services dimension captures the variation in the response to poverty-related food insecurity. Relatively larger communities, even when remote, are more likely to have more social and health services than small ones. Anticipating how the dominant discourse of food security and the capacity to respond to poverty will vary with the type of community, facilitates discussions of food security. The policy and services implications of recognizing nuanced meanings of rural and urban will be discussed.

**Reference**

1. Cromartie J, Nulph D, Hart G. Mapping frontier and remote areas in the US; 2012. [cited 2013 Aug 1]. Available from: http://www.ers.usda.gov/amber-waves/2012-december/data-feature-mapping-frontier-and-remote-areas-in-the-us.aspx#.UfrDj43CZ8H

## Helicobacter pylori

### Community-driven research of *Helicobacter pylori* infection in Arctic Canada: update from the Canadian North *Helicobacter pylori* Working Group

#### Katharine Fagan-Garcia^1^, Emily V. Hastings^1^, Laura McAlpine^1^, Andre Corriveau^2^, Brendan Hanley^3^, Sander JO Veldhuyzen van Zanten^1^, Monika Keelan^1^, Karen Goodman^1^, Hsiu-Ju Chang^1^; The CANHelp Working Group
^1^University of Alberta, Edmonton, Canada, aplin@ualberta.ca; ^2^NWT Health and Social Services, Yellowknife, Canada; ^3^Office of the Chief Medical Officer of Health, Edmonton, Canada

##### 

*Helicobacter pylori* infection occurs with increased frequency in the circumpolar north. The CANHelp Working Group formed to address community concerns about *H. pylori* describe the associated disease burden and inform health policy. At the request of community leaders, we initiated community projects in Aklavik, Tuktoyaktuk, and Fort McPherson, NT, and Old Crow, YT, during 2007–2012. Each project is guided by a local planning committee comprising community leaders, health care providers and research staff. Project components include: urea breath test (UBT) screening for *H. pylori*, structured interviews, endoscopy with gastric biopsy for pathology and microbiology, and a treatment trial. We use collected data to describe the disease burden and estimate effects of socio-environmental exposures on health outcomes. We develop knowledge exchange activities to share findings with community members and other stakeholders. In all communities combined, 921 participants have enrolled: 856 screened by UBT; 749 providing data on health history; 656 on individual socio-environmental exposures with household-level data for 412 households; 329 completing endoscopy; 267 enrolled in a treatment trial. Overall *H. pylori* prevalence was 61% (95% CI 57–64). Of the 231 *H. pylori*-positive persons with pathology results, 48% (95% CI 41–54) had severe gastritis and 43% (95% CI 37–50) had gastric atrophy. Of 205 *H. pylori* isolates, 43% (95% CI 36–50) were resistant to at least one antibiotic tested. Of trial participants with post-treatment status, treatment was successful in: 59% (95% CI 44–73) on triple therapy; 72% (95% CI 61–81) on sequential therapy; 95% (95% CI 83–99) on quadruple therapy. CANHelp community projects show a high prevalence of *H. pylori* infection, often accompanied by severe gastritis, which indicates elevated risk of gastric cancer. These findings show that community concern is warranted. This research will inform the development of regional *H. pylori* clinical and public health strategies.

## Investigating exposure to antibiotics among participants of community *Helicobacter pylori* projects in Arctic Canada

### Kathleen Williams^1^, Emily Hastings^1^, Katharine Fagan-Garcia^1^, Hsiu-Ju Chang^1^, Rachel Munday^2^, Karen Goodman^1^; CANHelp Working Group^1^
^1^University of Alberta, Edmonton, Canada, kfwillia@ualberta.ca; ^2^Susie Husky Health Centre, Aklavik, Canada

#### 

Chronic *Helicobacter pylori* infection increases the risk of peptic ulcers and gastric cancer. Residents of Canadian Arctic communities are disproportionately affected by *H. pylori*–associated diseases. Standard treatment to eliminate *H. pylori* has poor effectiveness in this region, due in part to antibiotic resistance of local *H. pylori* strains. As a preliminary step in investigating the influence of exposure to antibiotics on the prevalence of antibiotic-resistant strains, this analysis describes the antibiotic dispensation rate in Canadian Arctic communities and compares it to that of the Edmonton outpatient population. This analysis used data from projects conducted in 2007–2012 by the CANHelp Working Group in Aklavik, Tuktoyaktuk, Fort McPherson, NT, and Old Crow, YT. Project staff collected data from participants’ medical charts on the frequency and type of antibiotic prescriptions for the 5 years preceding project enrolment. Antibiotic dispensation rates in Edmonton for 2010–2013 were estimated from the Alberta Government Interactive Health Data Application. Chart reviews were completed for 297 participants from the four communities. The average number of antibiotic prescriptions per participant for the 5-year period was 4.6 (95% CI 4.1–5.1) and this average was higher among females (5.6; 95% CI 4.8–6.4) compared to males (3.4; 95% CI 2.9–3.9). The antibiotic prescription dispensation rate across communities was 0.94/person-year (95% CI 0.89–1.0). In contrast, the dispensation rate in Edmonton was 0.562/person-year (95% CI 0.561–0.563). The rate difference between the northern communities and Edmonton was 0.38/person-year (95% CI 0.33–0.43). This analysis estimates a notably higher antibiotic dispensation rate among residents of Arctic communities relative to the Edmonton outpatient population. This may be due in part to the limited availability of diagnostic technology in Arctic communities, leading to more frequent dispensation of antibiotics prior to confirmed diagnosis.

## Epidemiology of *Helicobacter pylori* infection among peoples of the Arctic

### Michael Bruce^1^, Karen Goodman^2^, Anders Koch^3^, Flemming Stenz^4^ and Vladislav Tsukanov^5^
^1^Centers for Disease Control and Prevention, Atlanta, GA, USA, zwa8@cdc.gov; ^2^University of Alberta, Edmonton, Canada; ^3^Statens Serum Institute, Copenhagen, Denmark; ^4^Office of the Chief Medical Officer, Nuuk, Greenland; ^5^Siberian Division of Russian Academy of Medical Sciences, Krasnoyarsk, Russia

#### 

*Introduction*. *Helicobacter pylori* infection is common among indigenous peoples of the Arctic, is a major cause of stomach ulcers and is associated with an increased risk of stomach cancer. *Objectives*. Review recent studies on *H. pylori* infection among peoples of the Arctic. *Methods*. Review of published data from studies on prevalence, antimicrobial resistance, treatment failure, peptic ulcer disease and gastric cancer in Arctic populations. *Results*. Seroprevalence is high among indigenous persons in Alaska (63–85%), northern Canada (51–95%), Greenland (47–58%), Northern Finland (52%) and Russia (77–90%) compared with persons living at lower latitudes in those countries. Data on antimicrobial susceptibility from Alaska from 2000 to 2013 demonstrate that 30% of *H. pylori* isolates from Alaska Native persons (AN) were resistant to clarithromycin, 45% to metronidazole and 14% to levofloxacin. Recent data from a northern Canadian First Nations community show that 31% of the isolates were resistant to one antibiotic and 20% were resistant to more than one. In a study performed in Greenland, 68% of children failed initial metronidazole-based treatment and in a recent treatment outcome study in northern Canada, 50% failed initial treatment with clarithromycin-based triple therapy. In a study from Alaska, 72% of persons with a clarithromycin-resistant isolate failed treatment with clarithromycin-based triple therapy. In Alaska, rates of 2-year reinfection were high at 16%. Rates of peptic ulcer disease are elevated in Alaska (299/100,000) and northern Canada (394/100,000). Gastric cancer incidence rates among indigenous people in Alaska, northern Canada, Greenland and Sweden are 2–4 times higher than those of non-indigenous people in the same countries. Indigenous Arctic residents have a high prevalence of *H. pylori* infection. In Alaska, there is a high rate of reinfection. Future studies looking at elevated rates of gastric cancer and *H. pylori* infection in the Arctic are warranted.

## Community-based research on *Helicobacter pylori* infection in Arctic Canada: determinants of the high prevalence of severe gastritis

### Emily Hastings, Safwat Girgis, Karen J. Goodman; The CANHelp Working Group
University of Alberta, Edmonton, Canada, evhastin@ualberta.ca

#### 

Residents of Arctic Aboriginal communities have a high prevalence of *Helicobacter pylori* infection. In response to community concerns about cancer risk from this infection, the CANHelp Working Group established community projects to investigate the disease burden and improve disease control strategies. We present preliminary analysis of the distribution and determinants of severe gastritis in Aklavik, Northwest Territories and Old Crow, Yukon, where community screening estimated *H. pylori* prevalence at 58% (192/332) and 68% (126/186), respectively. In 2008 in Aklavik and 2011 in Old Crow, 257 participants underwent upper endoscopy with gastric biopsy; 5 biopsies per person were assessed for gastritis severity by a single pathologist using the Sydney classification. Data on potential risk factors came from structured interviews. ORs and 95% CIs for the effect of exposures of interest on severe gastritis among *H. pylori*-positive (HP + ) participants were estimated by logistic regression. Of HP+ persons 97% had gastritis, which was mild in <5%, so gastritis severity was dichotomized as severe versus mild/moderate. The prevalence of severe gastritis was high (48%, CI 41–56%). As a potential risk factor of community interest, we estimated the effect of untreated river water consumption on severe gastritis prevalence. Among 160 HP+ persons with complete data, the estimated OR for this effect was 1.8 (CI 0.86–3.8) adjusting for age, ethnicity, education, alcohol, smoking, NSAID use and community. Given the potential for variation in water quality by community, this effect was also estimated by community (Aklavik 2.8, CI 1.1–7.2; Old Crow 0.85, CI 0.19–3.9). Our results show high frequencies of severe gastritis among HP+ individuals and a higher prevalence of severe gastritis among participants who consume untreated river water in Aklavik. Further analysis will include data from other communities to estimate effects of additional dietary factors.

## Rates of hospitalization with *Helicobacter pylori* and gastric cancer in American Indian and Alaska Native persons and in the United States population

### Ian Plumb^1^, Marissa Person^1^, Robert Holman^1^, Tom Hennessy^1^, Michael Bartholomew^2^, Claudia Steiner^3^ and Michael Bruce^1^
^1^Centers for Disease Control and Prevention, Atlanta, GA, USA, iplumb@cdc.gov; ^2^Indian Health Service, Rockville, MD, USA; ^3^National Institutes of Health, Bethesda, MD, USA

#### 

*Background*. *H. pylori* infects approximately 50% of the global population, and increases the risk for gastric cancer. Alaska Native populations have *H. pylori* antibody seroprevalence up to 75%, and high gastric cancer mortality. We estimated the rate of hospitalization associated with *H. pylori* infection and gastric cancer diagnosis in American Indians/Alaska Natives (AI/ANs) and in the general US population. *Methods*. We analysed two hospital discharge datasets for *H. pylori* or gastric cancer diagnoses during 2006 through 2011: the Indian Health Service (IHS) Direct and Contract Care Inpatient for AI/ANs, and the Nationwide Inpatient Sample for the general US population. Average annual age-specific and age-adjusted hospitalization rates were calculated, for 2006–2008 and 2009–2011, for AI/ANs and for the general US population. *Results*. From 2006–2008 to 2009–2011, average annual age-adjusted hospitalization rates/100 000 declined in the IHS AI/AN population for *H. pylori* by 35.0% (33.4–21.7), and for gastric cancer by 32.5% (12.6–8.5); respective rates for the Alaska region declined by 29.8% (44.7–31.4) and 14.8% (29.0–24.7). In the general US population, rates were estimated to decline by 22.1% for *H. pylori* from 18.1 (95% confidence interval [CI] 17.8–18.5) to 14.1 (CI 13.8–14.4); and remain stable for gastric cancer: 14.3 (CI 13.8–14.7) compared with 14.0 (CI 13.6–14.5). In 2009–2011, for both outcomes, rates were highest in AI/AN men aged ≥65 years residing in Alaska. *Discussion*. The hospitalization rates for *H. pylori* and for gastric cancer declined from 2006–2008 to 2009–2011 in AI/ANs, while rates for *H. pylori* declined and gastric cancer appeared to have plateaued in the general US population. In 2009–2011 both *H. pylori* and gastric cancer hospitalization rates in the Alaska region were higher than in the other IHS regions and in the general US population, especially among older males.

**References**

1. Demma LJ, Holman RC, Sobel J, Yorita KL, Hennessy TW, Paisano EL, et al. Epidemiology of hospitalizations associated with ulcers, gastric cancers, and *Helicobacter pylori* infection among American Indian and Alaska Native persons. Am J Trop Med Hyg. 2008;78:811–8.

2. Feinstein LB, Holman RC, Yorita Christensen KL, Steiner CA, Swerdlow DL. Trends in hospitalizations for peptic ulcer disease, United States, 1998–2005. Emerg Infect Diseases. 2010;16:1410–8.

3. Leontiadis G. I. NO. Epidemiology of *Helicobacter pylori* infection, peptic ulcer disease and gastric cancer. In: Talley NJ, editor. GI Epidemiology: diseases and clinical methodology, 2nd Edition. John Wiley and Sons; 2014. p. 135–57.

## The prevalence of iceA *Helicobacter pylori* gene in eastern Siberia (Sakha Republic)

### Nyurgun Gotovtsev^1^, Nikolay Barashkov^2^, Maria Pak^3^, Vera Pshennikova^1^, Adyum Rafailov^4^, Sargylana Lekhanova^5^, Savvinova Kyunnei^4^ and Fedorova Sardana^1^
^1^Laboratory of Molecular Biology, Institute of Natural Sciences, MK Ammosov North-Eastern Federal University, Yakutsk, Russia, Donzcrew@mail.ru; ^2^Yakut Scientific Center of Complex Medical Problems, Yakutsk, Russia; ^3^Department of Endoscopy, Republic Hospital No1 – National Center of Medicine, Yakutsk, Russia; ^4^Department of Biology, Institute of Natural Sciences, MK Ammosov North-Eastern Federal University, Yakutsk, Russia; ^5^Department of Normal and Pathological Anatomy, Operative Surgery with Topographic Anatomy and Forensic Medicine, MK Ammosov North-Eastern Federal University, Yakutsk, Russia

#### 

*Introduction*. *Helicobacter pylori* is a proven etiological agent associated with gastroduodenal diseases (1). Main factors of virulence and pathogenicity of *H. pylori* are considered to be the proteins CagA, VacA, BabA and IceA (2). iceA are most associated with peptic ulcer and chronic gastritis (CG). Recently, a study was undertaken on a meta-analysis of clinical outcomes in carriers of different variants of the gene iceA, in more than 5,000 people, which showed that the majority of patients with iceA1 allele are associated with peptic ulcer disease (3). In Yakutia, similar researches have not been conducted. Therefore, the aim of this study was to examine the prevalence of iceA *H. pylori* in eastern Siberia. *Material and methods*. The studied group consisted of DNA samples of *H. pylori*, which were isolated from biopsies of 42 Yakut patients with confirmed diagnosis of CG by histological and cytological study. *Results and discussion*. Among the investigated 42 clinical isolates obtained from biopsies of Yakut patients with CG, iceA1 was identified in 33 patients and iceA2 was detected in 9 patients. The frequency of iceA1 in Yakutia was 78.5% (CI 0.64–0.88), which was significantly higher than the average values recorded in Europe (29.5% CI 0.34–0.44) and the US (46.5% CI 0.31–0.41), but different from the average values recorded in Asia (58.9% CI 0.58–0.64). The frequency of iceA2 in Yakutia was 20.9% (CI 0.11–0.36) in the US (55.4% CI 0.50–0.60) and Europe (38.8% CI 0.38–0.48), but not different from Asia (26.3% CI 0.22–0.28). Comparing the received data from other countries studied, we concluded that the frequencies of iceA1/A2 in Yakutia correspond to frequencies of the iceA prevalent in Asia. *Conclusion*. The frequencies of iceA1/A2 in our study were significantly different from the frequencies in Europe and the US (p < 0.05), but were close to the frequencies in Asian populations (p > 0.05). The work was supported by the project No6.656.2014/K.

**References**

1. Suerbaum S, Michetti P. *Helicobacter pylori* infection. N Engl J Med. 2002;347:1175–86.

2. Atherton JC, Cao P, Peek RM Jr., et al. Mosaicism in vacuolating cytotoxin alleles of *Helicobacter pylori*. Association of specific vacA types with cytotoxin production and peptic ulceration. J Biol Chem. 1995;270:17771–7.

3. Shiota S, Watada M, Osamu M, Iwatani S, Suzuki R, Yamaoka Y. *Helicobacter pylori* iceA, clinical outcomes, and correlation with cagA: a meta-analysis. PLoS One. 2012;7:e30354.

## POSTER SESSIONS Tuesday June 9th 2015

### A. Infectious Diseases

#### Preliminary association of hepatitis B-specific, immune-mediated inflammatory responses with chronic hepatitis B disease activity

##### Brenna Simons-Petrusa^1^, Minjun Apodaca^2^, Syn Lim^3^, Tammy Choromanski^1^, Danielle Pratt^1^, Susan Negus^1^, Mary Snowball^1^, Chriss Homan^1^, Chihiro Morishima^2^, Cindy Knall^3^ and Brian J. McMahon^1^
^1^Alaska Native Tribal Health Consortium, Anchorage, AK, USA, bcsimons@anthc.org; ^2^University of Washington, Seattle, WA, USA; ^3^University of Alaska Anchorage, Anchorage, AK, USA

###### 

High prevalence of chronic hepatitis B (CHB) infection disproportionately affects indigenous Arctic populations. During CHB infection, the hepatitis B virus (HBV) can activate immune-mediated inflammation and liver damage. Some chronic HBV carriers experience active disease with high-HBV DNA levels and liver inflammation, some of whom may benefit by antiviral treatment, while others experience inactive disease with low HBV DNA levels and no liver inflammation. Clinical management of chronic HBV can be challenging, often requiring ultrasound or liver biopsy, procedures that may not be available in many rural Arctic communities. Biomarkers of CHB disease status that can easily be measured from a small blood draw are needed. The role of adaptive immunity in CHB infection and whether disease status can be inferred from immune biomarkers is incompletely understood. In this study, we compared HBV-specific immune responses between persons with inactive (n=9) and active (n=9) CHB. Peripheral blood mononuclear cells (PBMCs) were assessed by flow cytometry for the frequency and phenotype of immune cells. Ex vivo cultures were established to measure HBV-specific IFNγ cytokine response by flow cytometry and ELISpot. A trend towards an increase in regulatory T cells (Treg) amongst inactive carriers was identified. CD45RO+CD4+FoxP3+ Treg cell frequency was significantly higher (p=0.019) amongst active versus inactive carriers. Pro-inflammatory, HBV-specific IFNγ responses were significantly increased (p=0.05), while anti-inflammatory HBV-specific IL-10 responses were significantly decreased (p=0.028), amongst active carriers. Collectively, these data suggest increased immune-mediated inflammatory responses amongst active carriers. Recent improvements in laboratory methods may allow for the expansion of cellular immune testing in remote settings, paving the way for needed population-based immune studies of CHB infection in the Arctic.

## Use of Diaskintest for selection of children and adolescents from tuberculosis risk groups for preventive therapy

### Larisa I. Mordovskaya, Natal’ya M. Oshchepkova, Evgeniya N. Il’ina, Maria K. Vinokurova and Alexander F. Kravchenko
Research & Practice Center for Tuberculosis of the Sakha Republic (Yakutia), Yakutsk, Russia, limordovskaya@mail.ru

#### 

*Background*. Extreme environment, low population density, and troublesome road pattern are characteristic features of the Sakha Republic (Yakutia), associated with challenges in the organization of public health care. Tuberculosis (TB) is considered an endemic disease in Yakutia and is one of the urgent social and health care problems. Incidence of TB among paediatric population remains high. Starting in 2010, a novel test for TB infection, Diaskintest (DST) based on recombinant allergen derived from ESAT-6 and CFP-10 proteins, has been included to compulsory examination of children and adolescents. *Material and methods*. We examined 783 children and adolescents aged 1–17, residents of the Tulagino village, having the following diagnoses: postvaccinal complications (PVC) (n=318), early phase of primary TB infection (EPPTBI) (n=195), growing tuberculin sensitivity (n=259) and condition after localized forms of TB (n=11). All patients received two tests at a time (Mantoux test with 2 TU PPD-L and DST); tests were administered and interpreted in compliance with practice guidelines. *Results*. In 195 children with EPPTBI, DST results were negative (162; 83%), doubtful (23; 12%) and positive (9; 4.6%). DST results in patients with growing tuberculin sensitivity were negative (195; 75.3%), doubtful (34; 13.1%), positive (28; 10.8%) and hyperergic (2; 0.8%). DST results in patients with PVC were negative (99%) or doubtful (3; 1%). Patients with a condition after localized forms of TB (n=6) had the following responses to DST: hyperergic (5; 83.3%), markedly positive (1; 16.7%) and positive (5). *Conclusions*. DST allows accurate identification of children and adolescents at high risk for TB, to select patients for preventive therapy.

## Major genotypes of *Mycobacterium tuberculosis* in new cases of tuberculosis in the High North of Russia

### Maria K. Vinokurova^1^, Nadezhda E. Evdokimova^1^, Galina I. Alekseeva^1^, Oleg B. Ogarkov^2^, Svetlana N. Zhdanova^2^ and Alexander F. Kravchenko^1^
^1^Research & Practice Center for Tuberculosis of the Sakha Republic (Yakutia), Yakutsk, Russia, mkvin61@mail.ru; ^2^Institute of Epidemiology and Microbiology, Irkutsk, Russia

#### 

With growing incidence of multidrug resistance (MDR) and extensive drug resistance (XDR) of *Mycobacterium tuberculosis* (MTB), the study of the population structure of MTB circulating in areas with unfavourable epidemiological situation for tuberculosis (TB), such as Yakutia, is becoming especially important. MIRU-VNTR genotyping was performed for MTB isolates from 129 new cases of TB, for the period from 2010 to 2012. Drug resistance was determined using classic culture test and BACTEC-960 system. Patient data were analysed by gender, age, ethnicity and clinical presentation of pulmonary TB. All patients were HIV-negative, mostly male (62.0%). Patients aged 18 to 29 made 36.4%; 34.1% of patients were older than 50. Urban residents account for 80.6%, and the rest were residents of remote rural settlements. 58.1% were aboriginal peoples of the North. The most prevalent clinical form was infiltrative TB (73.6%) with destructions in the lungs. Drug-resistant MTB were found in 56.6%. Among 56 drug-resistant cases, 12.5% were monoresistant, 25.0% were polyresistant, 59.0% were MDR and 3.5% were XDR. Strains belonged to the following families: Beijing (33.3%), Orphan (19.4%), S (13.2%), T/H (8.5%), Ural (8.5%), Haarlem (7.8%), LAM (4.7%), Uganda (3.9%) and Cameroon (1 case). MDR was determined only in four families: Beijing (48.5%), S (39.4%), Orphan (9.1%) and LAM (3.0%). XDR was detected in S 256 and Orphan 805 families (1 case each). Resistance profile of 75.8% of cases of MDR TB was a combination of isoniazid+rifampicin+streptomycin+etambutol. Two XDR cases were additionally resistant to kanamycin+fluoroquinolone+capreomycin. This is the first study on genotyping of MTB in Yakutia, and it showed the predominance of strains from Beijing, Orphan and S families. The same families were the most prevalent in MDR TB. More advanced molecular epidemiologic studies are needed in the future, to improve epidemiologic surveillance for TB infection in the High North environment.

## The problem of increase in multidrug-resistant tuberculosis cases based on the results of regional-level monitoring in the Sakha Republic (Yakutia)

### Alexander F. Kravchenko, Maria K. Vinokurova, Semyon N. Kondakov and Oksana E. Dogorova
Research & Practice Center for Tuberculosis of the Sakha Republic (Yakutia), Yakutsk, Russia, mkvin61@mail.ru

#### 

*Aim*. To study the results of detection and chemotherapy effectiveness monitoring in newly identified patients with pulmonary tuberculosis (PTB). *Material and methods*. Annual cohorts of patients with PTB in the Sakha Republic (Yakutia) for the period from 2005 to 2013. *Results and discussion*. Starting in 2005, monitoring programme is being carried out in Yakutia, adapted to the WHO standards. BACTEC-960 system has been introduced in 2009, to improve detection of tuberculosis (TB). From 2005 to 2013, quarterly coverage with culture tests in newly identified PTB increased from 76.3 to 98.1%. Rate of timely drug sensitivity tests performed increased from 66.2 to 95.0%. Incidence of primary multidrug resistance (MDR) detected during registration of new cases increased from 2.1 to 16.6%. In 2006, cases resistant to isoniazid and rifampicin were resistant also to streptomycin (93.2%), kanamycin (41.3%), etambutol (18.1%), ethionamide (12.1%), capreomycin (9.6%), PAS (8.9%) and fluoroquinolones (6.4%). In 2013, resistance spectrum changed a bit: streptomycin (98.7%), kanamycin (44.0%), etambutol (26.0%), fluoroquinolones (24.3%), capreomycin (17.0%), ethionamide (9.2%), PAS (5.5%) and cycloserine (9.2%). Over the last 4 years, the effectiveness of 12-month chemotherapy was 68.9–71.5%. Treatment failures (15.6–10.0%) were observed in 80–92% of cases due to MDR. 5.2–6.4% of patients died, in 2.3–3.2% TB was the cause of death. 2.8–3.2% defaulted, and 1.6–5.7% were transferred out from the republic. There were only solitary cases of removal from the register of TB patients (0.7–1.0%). In the cohort of MDR TB patients, 67.4–68.7% had been effectively treated, and surgical treatment had been used in almost half of the cases. *Conclusion*. Monitoring results showed improved detection of TB and chemotherapy effectiveness in Yakutia. We observed an alarming growing trend of MDR TB cases among new cases of TB and an increasing drug resistance trend.

## Organization of the bacteriological diagnosis and treatment of tuberculosis in Yakutia

### Alexander F. Kravchenko, Galina I. Alekseeva and Maria K. Vinokurova
Research & Practice Center for Tuberculosis of the Sakha Republic (Yakutia), Yakutsk, Russia, alex220560@yandex.ru

#### 

*Introduction*. Yakutia is the largest region of Russia (3.2 million km^2^, with 40% lying beyond the Arctic Circle), known for long (50–3,000 km) distances between administrative districts and Yakutsk, the capital with low average population density (0.3–1 per 1 km^2^) and a strongly continental climate. Vastness and extreme environment present challenges to bacteriologic service organization in Yakutia. We developed a model of bacteriologic service, consisting of the Central Laboratory in Yakutsk and laboratories in the District TB Dispensaries (early treatment clinics). Diagnosis is done in two steps. Step 1 is performed by primary network clinics or TB Dispensaries. Step 2 is performed strictly by laboratories in TB Dispensaries. The percentages of new pulmonary cases positive for *Mycobacterium tuberculosis* (MTB) in 2012, 2011 and 2010 were 51.2%, 55.6% and 54.1%, respectively. Since the introduction of BACTEC MGIT-960 and GeneXpert Dx systems, final results are ready in 21.1 days on average, compared to 64.6 days using classic methods. Cryogenic preservation using natural frost did not influence MTB detection rate, using BACTEC and GeneXpert systems. Also, real-time PCR laboratory started to function in 2013. Patients with established diagnoses of TB undergo their treatment in the TB Center, which offers special departments for surgical treatment of pulmonary and extra-pulmonary TB, paediatric TB and MDR-TB. The effectiveness of chemotherapy was nearly 85% in the absence of MDR, and more than 65% in MDR cases, but was significantly lower in relapsed and chronic TB. *Conclusion*. TB service in Yakutia is well equipped for diagnosis and treatment of TB. Use of up-to-date technologies shortens the time to diagnosis and start of adequate chemotherapy, and the use of new treatment methods for TB and concurrent diseases. Establishment of the TB Centre in the challenging conditions of a circumpolar region allows sufficient TB surveillance.

## Determination of NAT2 acetylation status in the Greenlandic population – an enzyme related to tuberculosis therapy

### Frank Geller, Bolette Soborg, Anders Koch, Sascha Wilk Michelsen, Bjarke Feenstra and Mads Melbye
Statens Serum Institut, Copenhagen, Denmark, ako@ssi.dk

#### 

N-acetyltransferase 2 (NAT2) is a well-studied phase II xenobiotic metabolizing enzyme relevant in drug metabolism and cancerogenesis. NAT2 activity is largely determined by genetic polymorphisms in the coding region of the corresponding gene. We investigated NAT2 acetylation status in 1,556 individuals from Greenland based on four different single nucleotide polymorphism (SNP) panels and the tagging SNP rs1495741. There was good concordance between the NAT2 status inferred by the different SNP combinations. Overall, the fraction of slow acetylators was low with 17.5% and varied depending on the degree of Inuit ancestry; in individuals with less than 50% Inuit ancestry, we observed more than 25% slow acetylators reflecting European ancestry. Greenland has a high incidence of tuberculosis, and individual dosing of isoniazid, according to NAT2 status, has been shown to improve treatment and reduce side effects. Our findings could be a first step in pharmacogenetics-based tuberculosis therapy in Greenland.

## Rates of hospitalization with *Helicobacter pylori* and gastric cancer in American Indian and Alaska Native persons and in the United States population

### Ian Plumb^1^, Marissa Person^1^, Robert Holman^1^, Tom Hennessy^1^, Michael Bartholomew^2^, Claudia Steiner^3^ and Michael Bruce^1^
^1^Centers for Disease Control and Prevention, Atlanta, GA, United States, iplumb@cdc.gov; ^2^Indian Health Service, Rockville, MD, United States; ^3^National Institutes of Health, Bethesda, MD, United States

#### 

*Background*. *Helicobacter pylori* infects approximately 50% of the global population and increases the risk for gastric cancer. Alaska Native populations have *H. pylori* antibody seroprevalence up to 75% and high gastric cancer mortality. We estimated the rate of hospitalization associated with *H. pylori* infection and gastric cancer diagnosis in American Indians/Alaska Natives (AI/ANs) and in the general US population. *Methods*. We analysed two hospital discharge data sets for *H. pylori* or gastric cancer diagnoses during 2006 through 2011: the Indian Health Service (IHS) Direct and Contract Care Inpatient for AI/ANs, and the Nationwide Inpatient Sample for the general US population. Average annual age-specific and age-adjusted hospitalization rates were calculated, for 2006–2008 and 2009–2011, for AI/ANs and for the general US population. *Results*. From 2006–2008 to 2009–2011, the average annual age-adjusted hospitalization rates per 100,000 declined in the IHS AI/AN population for *H. pylori* by 35.0% (33.4 to 21.7), and for gastric cancer by 32.5% (12.6 to 8.5); respective rates for the Alaska region declined by 29.8% (44.7 to 31.4) and 14.8% (29.0 to 24.7). In the general US population, rates were estimated to decline by 22.1% for *H. pylori* from 18.1 (95% confidence interval [CI] 17.8–18.5) to 14.1 (CI 13.8–14.4) and remain stable for gastric cancer: 14.3 (CI 13.8–14.7) compared with 14.0 (CI 13.6–14.5). In 2009–2011, for both outcomes, rates were highest in AI/AN men aged ≥65 years residing in Alaska. *Discussion*. The hospitalization rates for *H. pylori* and for gastric cancer declined from 2006–2008 to 2009–2011 in AI/ANs, while rates for *H. pylori* declined and gastric cancer appeared to have plateaued in the general US population. In 2009–2011, both *H. pylori* and gastric cancer hospitalization rates in the Alaska region were higher than in the other IHS regions and in the general US population, especially among older males.

**References**

1. Demma LJ, Holman RC, Sobel J, Yorita KL, Hennessy TW, Paisano EL, et al. Epidemiology of hospitalizations associated with ulcers, gastric cancers, and *Helicobacter pylori* infection among American Indian and Alaska Native persons. Am J Trop Med Hyg. 2008;78:811–18.

2. Feinstein LB, Holman RC, Yorita Christensen KL, Steiner CA, Swerdlow DL. Trends in hospitalizations for peptic ulcer disease, United States, 1998–2005. Emerg Infect Dis. 2010;16:1410–18.

3. Leontiadis GI, Nyren O. Epidemiology of *Helicobacter pylori* infection, peptic ulcer disease and gastric cancer. In: Talley NJ, editor. GI epidemiology: diseases and clinical methodology, Second Edition, New York, NY: John Wiley and Sons; 2014. p. 135–57.

## The prevalence of iceA *Helicobacter pylori* gene in eastern Siberia (Sakha Republic)

### Nyurgun Gotovtsev^1^, Nikolay Barashkov^2^, Maria Pak^3^, Vera Pshennikova^1^, Adyum Rafailov^4^, Sargylana Lekhanova^5^, Savvinova Kyunnei^4^ and Fedorova Sardana^1^
^1^Laboratory of Molecular Biology, Institute of Natural Sciences, MK Ammosov North-Eastern Federal University, Yakutsk, Russia, Donzcrew@mail.ru; ^2^Yakut Scientific Center of Complex Medical Problems, Yakutsk, Russia; ^3^Department of Endoscopy, Republic hospital No1 – National Center of Medicine, Yakutsk, Russia; ^4^Department of Biology, Institute of Natural Sciences, MK Ammosov North-Eastern Federal University, Yakutsk, Russia; ^5^Department of Normal and Pathological Anatomy, Operative Surgery with Topographic Anatomy and Forensic Medicine, MK Ammosov North-Eastern Federal University, Yakutsk, Russia

#### 

*Introduction*. *Helicobacter pylori* is a proven etiological agent associated with gastroduodenal diseases (1). The proteins CagA, VacA, BabA and iceA are considered to be the main factors of virulence and pathogenicity of *H. pylori* (2). iceA are most associated with peptic ulcer and chronic gastritis (CG). Recently, a study undertaken on a meta-analysis of clinical outcomes in carriers of different variants of the gene iceA, which has been studied by more than 5,000 people, showed that the majority of patients’ iceA1 allele is associated with peptic ulcer disease (3). In Yakutia, similar researches have not been conducted. Therefore, the aim of this study is to examine the prevalence of iceA *H. pylori* in eastern Siberia. *Material and methods*. The studied group consisted DNA samples of *H. pylori*, which were isolated from biopsies of 42 Yakut patients with confirmed diagnosis of CG by histological and cytological study. *Results and discussion*. Among the investigated 42 clinical isolates obtained from biopsies of Yakut patients, CG iceA1 was identified in 33 patients and iceA2 was detected in 9 patients. Frequency of iceA1 in Yakutia was 78.5% (CI=0.64–0.88), which was significantly higher than the average values recorded in Europe (29.5%, CI=0.34–0.44) and America (46.5% CI=0.31–0.41), but differs from the average values recorded in Asia (58.9%, CI=0.58–0.64). Frequency of iceA2 in Yakutia was 20.9% (CI=0.11–0.36), in America (55.4%, CI=0.50–0.60) and in Europe (38.8%, CI=0.38–0.48), but not different from Asia (26.3%, CI=0.22–0.28). When comparing the data received from other countries’ studies, we concluded that the frequencies of iceA1/A2 in Yakutia correspond to the frequencies of the iceA circulating in Asia. *Conclusion*. The frequencies of iceA1/A2 in our study differ significantly from the frequencies in Europe and America (p<0.05), but were close to the values of the frequencies in Asian populations (p>0.05). The work was supported by the project no. 6.656.2014/K.

**References**

1. Suerbaum S, Michetti P. *Helicobacter pylori* infection. N Engl J Med. 2002;347:1175–86.

2. Atherton JC, Cao P, Peek RM Jr, Tummuru MK, Blaser MJ, Cover TL. Mosaicism in vacuolating cytotoxin alleles of *Helicobacter pylori*. Association of specific vacA types with cytotoxin production and peptic ulceration. J Biol Chem. 1995;270:17771–7.

3. Shiota S, Watada M, Osamu M, Iwatani S, Suzuki R, Yamaoka Y. *Helicobacter pylori* iceA, clinical outcomes, and correlation with cagA: a meta-analysis. PLoS One. 2012;7:e30354.

## GJB2 mutation spectrum in patients with congenital hearing loss in Yakutia (eastern Siberia)

### Pshennikova Vera^1^, Barashkov Nikolay^2^, Teryutin Fedor^2^, Solovyev Aisen^1^, Klarov Leonid^3^ and Romanov Georgii^1^
^1^Institute of Natural Sciences, M.K. Ammosov North-Eastern Federal University, Yakutsk, Russian Federation, psennikovavera@mail.ru; ^2^Yakut Scientific Center of Complex Medical Problems, Siberian Branch of the Russian Academy of Medical Sciences, Yakutsks, Russian Federation; ^3^Republican Hospital # 2 – Center of Emergency Medicine, Yakutsks, Russian Federation

#### 

Mutations in the GJB2 gene, encoding the protein connexin 26 (Cx26), are recognized as a major cause of congenital hearing loss. More than 150 pathogenic mutations are identified in the GJB2 gene, where spectrum and frequency vary considerably among different ethnic groups (1, 2). Until now, the spectrum and the frequency of mutations in the GJB2 gene (exon 1 and exon 2) were not fully described in the Yakutia (eastern Siberia). The complete resequencing of promoter and coding region of the GJB2 gene was performed for the first time in 580 persons in Yakutia, of which 393 patients were having congenital hearing impairment (360 unrelated) and 187 were normal hearing individuals. In the total sample (n=580), 13 allelic variants of GJB2 gene were revealed, of which 8 were recessive mutations. Twenty-one different GJB2 genotypes were shown in 393 patients, of which 10 genotypes had double recessive mutations. The contribution of GJB2 gene mutations in hearing loss in the population of Yakutia (45.55%) is the highest among all previously studied regions in Asia. In the investigated sample of patients, three most frequent mutations, c.-23+1G>A, c.35delG and c.109G>A, were identified. For Yakut patients, the most common mutation was c.-23+1G>A (94.17% of all mutant chromosomes), and for Russian patients c.35delG (73.07% of all mutant chromosomes). Carrier frequency mutations of c.-23+1G>A and c.109G>A in Yakut population were 11.21 and 2.80%, respectively. The carrier frequency mutation of c.35delG in Russians was 2.5%. Thus, the search algorithm mutations in the GJB2 gene in the routine DNA diagnosis in patients in Yakutia may be limited to three frequent mutations c.-23+1G>A, c.35delG and c.109G>A. The study was supported by Project no. 6.656.2014/к of Ministry of Education and Science of Russia, RFBR (#14-04-01741_A, 14-04-9010_Bel_A, 15-44-05106-r_vostok_a), SBRAS Integration project #92, Sakha Republic President grant for Young Researchers for 2015 and RAS Program (#30 for 2013–2015).

## B. Environmental Contaminants

### Organic anion transporter 4 modifies placental transfer of perfluorinated alkyl substances PFOS and PFOA

#### Elina Sieppi, Arja Rautio, Päivi Myllynen and Maria Kummu
University of Oulu, Oulu, Finland, maria.kummu@oulu.fi

##### 

Perfluorinated alkyl substances (PFAS) are widely used in industry and consumer products. Many of these compounds are found widely in the nature and humans. For instance, perfluorooctane sulphonate (PFOS) and perfluorooctanoate (PFOA) are found in human blood and they are known to affect human health (1). The main route for exposure is dietary, but their almost ubiquitous presence in the environment leads to continuous exposure through various routes. The presence of PFAS in umbilical cord blood also suggests foetal exposure to these compounds (2). Transporter proteins are widely expressed in barrier tissues of human body. They have a role in both absorption and excretion of physiological and xenobiotic compounds. Placenta plays a key role in foetal exposure to xenobiotics. In human placenta, both organic anion transporter (OAT4) and ATP-binding cassette transporter G2 (ABCG2) transporter proteins are highly expressed. ABCG2 has been shown to protect foetus from xenobiotics, and also OAT4 is suspected to have a similar function based on localization and function (3). In this project, the placental kinetics of PFOS and PFOA was studied ex vivo using dual recirculating human placental perfusion. PFC concentrations were analysed using LC-MS/MS. Placental OAT4 and ABCG2 transporter expressions were studied using immunoblotting, and transporter protein expressions were correlated with transfer indexes (TI%) of PFAS. Both PFOS and PFOA crossed placenta during 4-hour perfusions with average foetal to maternal concentration ratios of 0.26 and 0.20, respectively. OAT4 and ABCG2 were expressed in all of the studied placentas, and as expected, the expression levels showed person-to-person variation. Interestingly, OAT4 expression correlated significantly with TI% of PFOA and PFOS at 120 min (12.9±1.5%, R2=0.92, p=0.043 and 14.4±3.9%, R2=0.99, p=0.007, respectively). ABCG2 expression did not correlate with PFAS transfer.

**References**

1. Giesy JP, Kannan K. Global distribution of perfluorooctane sulfonate in wildlife. Environ Sci Technol. 2001;35:1339–42.

2. Gützkow KB, Haug LS, Thomsen C, Sabaredzovic A, Becher G, Brunborg G. Placental transfer of perfluorinated compounds is selective–a Norwegian mother and child sub-cohort study. Int J Hyg Environ Health. 2012;215:216–19. doi: http://dx.doi.org/10.1016/j.ijheh.2011.08.011

3. Vähäkangas K, Myllynen P. Drug transporters in the human blood-placental barrier. Br J Pharmacol. 2009;158:665–78. doi: http://dx.doi.org/10.1111/j.1476-5381.2009.00336.x

## The xeno-oestrogens, bisphenol A and para-nonylphenol, decrease the expression of ABCG2 transporter protein in human term placenta

### Elina Sieppi^1^, Kirsi Vähäkangas^2^, Arja Rautio^3^, Luana Ricci Paulesu^4^ and Päivi Myllynen^5^
^1^Department of Pharmacology and Toxicology, Institute of Biomedicine, University of Oulu, Oulu, Finland, elina.sieppi@oulu.fi; ^2^Faculty of Health Sciences, School of Pharmacy/Toxicology, University of Eastern Finland, Kuopio, Finland; ^3^Centre for Arctic Medicine, Thule Institute, University of Oulu, Oulu, Finland; ^4^Department of Life Sciences, University of Siena, Siena, Italy; ^5^Nordlab Oulu, Oulu, Finland

#### 

Nowadays, environment is widely contaminated by different chemicals including mainly industry-produced xeno-oestrogens. Xeno-oestrogens are compounds mimicking natural oestrogens and affecting many endocrinological systems. Xeno-oestrogens, like bisphenol A (BPA) and p-nonylphenol (p-NP), are an increasing concern for people and especially for pregnant women who experience important endocrinological changes (1). Developing foetus is sensitive for xeno-oestrogens, because maintenance of placenta and normal foetal development is highly regulated by endocrine system. Placenta is also an endocrinological organ being maintained hormonally but also producing hormones itself to maintain pregnancy. Human placenta connects mother and foetus enabling pregnancy. Placenta expresses several ABC transporters that facilitate the transport of both endogenous compounds and xenobiotics. Efflux transporters, like ABCG2 and ABCB1/4, have been shown to participate in foetal protection against xenobiotics. Regulation of transporters is still under study, but hormones and xenobiotics are known to participate (2,3). The effects of xeno-oestrogens (BPA, p-NP) on ABC transporter (ABCG2, ABCB1) expression were studied by human placental chorionic villous explants. Estradiol was used as a positive control for hormonal response. The effect of gestational age of placenta was studied using both first trimester and term placentas. The role of oestrogen receptors in the effects of chemicals on ABCG2 was studied using ER-α antagonist. Results showed that ABCG2 expression decreases with advancing gestation. All studied chemicals decreased the expression of ABCG2 protein in term placentas. Also, ABCB1 was decreased by xeno-oestrogens. The antagonist and chemicals used interacted differently implying different mechanisms between chemicals. In conclusion, environmental xeno-oestrogens affect the expression of ABC transporters and hence may affect the human placental transport of endogenous and xenobiotic compounds.

**References**

1. Vähäkangas K, Myllynen P. Drug transporters in the human blood-placental barrier. Br J Pharmacol. 2009;158:665–78.

2. Ni Z, Mao Q. ATP-binding cassette efflux transporters in human placenta. Curr Pharm Biotechnol. 2011;12:674–85.

3. Robins JC, Marsit CJ, Padbury JF, Sharma SS. Endocrine disruptors, environmental oxygen, epigenetics and pregnancy. Front Biosci. 2011;3:690–700.

## C. Indigenous Health

### Disparities in infant mortality, stillbirth and preterm birth among First Nations, Inuit and Métis populations in Canada

#### Amanda J. Sheppard^1^, Seungmi Yang^2^, Tracey Bushnik^3^, Mourad Dahhou^2^, Serenity Perry^4^, Jay S. Kaufman^2^, Russell Wilkins^5^ and Michael Kramer^2^
^1^The Hospital for Sick Children, Toronto, Canada, amanda.sheppard@sickkids.ca; ^2^McGill University, Montréal, Canada; ^3^Statistics Canada, Fort William First Nation, Canada; ^4^Ontario Native Women’s Association, Fort William First Nation, Canada; ^5^University of Ottawa, Ottawa, Canada

##### 

*Introduction*. The term “Aboriginal” comprises three constitutionally recognized populations in Canada: First Nations, Métis and Inuit, each with unique cultural identities, social determinants of health and health care funding inequities. Data on birth outcomes in Canada have been limited due to the lack of Aboriginal birth identifiers on birth registrations in most provinces and territories, and almost entirely lacking for Métis. Based on data of limited quality, birth outcomes have been reported to be significantly poorer among Aboriginal peoples compared to their non-Aboriginal counterparts and suggest disparities among the three Aboriginal groups. *Objective*. To compare risks of infant mortality, stillbirth and preterm birth (<37 completed weeks) among the three Aboriginal populations. *Methods*. We analysed a cohort of births between May 2004 and May 2006 created by linking the Canadian perinatal health database with the long form of the 2006 Canadian census, which includes an Aboriginal self-reported identifier. *Results*. The crude infant mortality rate for the overall Aboriginal population was 9.8 (8.3, 11.4) per 1000, and 8.6 (7.1, 10.2), 10.8 (6.6, 15.1) and 11.9 (6.8, 17.0) per 1000 live births among First Nations, Métis and Inuit, respectively. For stillbirths, the corresponding rates were 8.9 (7.5, 10.4) per 1000 total births overall and 9.2 (7.6, 10.8), 6.1 (2.9, 9.2) and 8.5 (4.2, 12.8) in the three groups. Preterm birth rates were 99.1 (94.4, 103.7) per 1000 total births overall and 96.0 (91.1, 101.0), 81.6 (70.4, 92.8) and 124.3 (108.8, 139.8) in the three groups. *Conclusions*. The pan-Aboriginal approach to birth outcome reporting in Canada has masked substantial disparities across Canada’s three Aboriginal populations. The data demonstrate the need for targeted maternal and infant health programmes to reduce adverse birth outcomes in these populations.

## Visual graphics as a tool for communication and relationship building

### Melody Morton Ninomiya
Memorial University, St. John’s, Canada, melodym@mun.ca

#### 

Ethical and authentic relationships between (indigenous and non-indigenous) researchers and indigenous peoples are, in part, facilitated through effective and appropriate communication. In this poster, I illustrate and describe how researchers can improve communication of information through the use of visual graphics throughout all research phases. Visual graphics such as graphs, charts, figures and photographs are common in scientific research (especially dissemination) materials; however, they are less common as communication tools in research. Using examples from my own community-based health research project with a rural indigenous community in eastern Canada, I demonstrate how visual graphics made communication of information both accessible and engaging with community members, research informants and (indigenous and non-indigenous) government stakeholders at different phases of research. While I advocate that researchers use visual graphics to enhance and improve communication and build authentic relationships, I also argue that researchers must carefully consider how visual graphics are used and what they represent.

## Tell us what you need and we’ll build it together: Government–community partnerships for cancer prevention and support in the Northwest Territories

### Andre Corriveau^1^, Sabrina Broadhead^1^, Crystal Milligan^1^, Melinda Laboucan^2^ and Florence Barnaby^3^
^1^GNWT-HSS, Yellowknife, Canada, andre_corriveau@gov.nt.ca; ^2^K’asho Got’ine Community Band, Yellowknife, Canada; ^3^Elders’ Council, STHA, Yellowknife, Canada

#### 

Concerns about increasing rates of cancer have emerged as a priority in many aboriginal communities across the Northwest Territories (NWT). This has led to the development by the Department of Health and Social Services (GNWT-HSS) of its first 5-year cancer strategy. Among GNWT-HSS partners, the communities of the are arguably the most important. Communities have the purest understanding of their needs and challenges, and thus, their input is crucial to design sustainable programmes. They also advise on matters such as culturally appropriate care, traditional ways of healing and appropriate terminology. The GNWT-HSS follows a community engagement approach based on knowledge exchange, collaborative planning and respect. Cancer sharing circles – where communities share experiences and voice concerns and learn about cancer – prove to be highly effective to initiate conversations about cancer and identify community-based solutions. Participants leave with knowledge and motivation to own these solutions and access support from their local leaders, regional organizations and the GNWT-HSS. In 2012, a cancer sharing circle took place in Fort Good Hope. The community has since begun cancer terminology development in the local language and formed a committee that meets regularly to address challenges in cancer prevention and support. The NWT-HSS provides financial, logistical and in-kind support for their work, including the development of a community strategic plan for cancer that will align with the territorial cancer strategy. Government–community partnerships can drive community action. Community members with the capacity to support one another enhance the capacity of the health system as a whole. In the long term, we aim to establish a community-to-community mentorship and support model as part of a community-based strategy for sustainable improvement to cancer care.

## Circumpolar Inuit health priorities: best health practices and research

### Leanna Ellsworth, Eva Kruemmel, Annmaree O’Keeffe and Stephanie Meakin
Inuit Circumpolar Council (ICC), Ottawa, Canada, lellsworth@inuitcircumpolar.com

#### 

Inuit are indigenous peoples from four of the eight Arctic countries, Alaska (US), Canada, Greenland and Chukotka (RU), and are represented internationally by the Inuit Circumpolar Council (ICC). Inuit health and well-being is a policy priority for ICC. A part of ICC’s work is to document the different health and wellness experiences of Inuit and to advocate for solutions within international forums. ICC has completed two reports as part of this work: The first was *Health Systems serving Inuit communities across the Arctic*, completed in 2011 and presented at ICCH15. The second, *Circumpolar Inuit Health Priorities: Best Health Practices and Research*, identifies, documents and assesses the range of projects, studies and practices that have been implemented across the Arctic. Together, the material provides an important collection of information on the health practices and challenges, which impact on the health and well-being of Inuit across the four countries directly and indirectly. The main areas covered in the report include mental health and wellness, service delivery, food security and chronic disease. A total of 284 programmes and studies were found during the research for the report. The number does not represent an exhaustive compilation of all best programmes and studies being conducted, but reflect the extent of the search undertaken from July 2011 to February 2012. During the project, we found that there is an abiding constraint in accessing relevant data on indigenous health overall, but particularly of Inuit-specific data in Alaska and Russia. This limited availability influenced the type and breadth of information found. The lack of indigenous health data is a common concern globally and is frequently cited as a major constraining factor in understanding better the major issues impacting on indigenous health. This presentation will summarize ICC’s findings and highlight a new initiative of an online Inuit mental health and wellness map, created in 2014.

## Reindeer-herding Sami experiences of seeking care in the mainstream society

### Anette Edin-Liljegren, Klas-Göran Sahlén, Lars Jacobsson and Laila Daerga
Umeå University, Umeå, Sweden, laila.daerga@vll.se

#### 

*Background*. The Sami people are one of the five recognized national minorities in Sweden. A minority of the Sami is working with reindeer herding that periodically involves harsh working conditions that probably can lead to injury and disease. Previous studies (1,2) indicate that the confidence in primary health care and psychiatry was lower among the reindeer-herding Sami in Sweden compared with a control population taken from the same environments in northern Sweden. *Aims*. To identify possible underlying causes of the previously reported deficiencies in confidence between Sami and staff in health services and social services, respectively. The research will highlight subjectively perceived experiences of seeking healthcare services. *Methods*. Data will be collected through semi-structured research interviews and analysed by qualitative methods. A grounded theory approach will be used. Both men and women of various ages from reindeer-herding families in northern Sweden will be interviewed. The selection of informants will be chosen through a mix of purposive stratified sampling and snowball sampling. *Results*. Collection of data has just started, so there are no results yet. Preliminary results will be presented at conference in Oulu.

**References**

1. Nystad TA, Melhus M, Lund E. Sami speakers are less satisfied with general practioners services. Int J Circumpolar Health. 2008;67:114–21.

2. Daerga L, Sjölander P, Jacobsson L, Edin-Liljegren A. The confidence in health care and social services in northern Sweden – a comparison between reindeer-herding Sami and the non-Sami majority population. Scand J Public Health. 2012;40:516–22.

## Isonymy as an indicator of inbreeding in child populations of Yamal and Gyda tundras

### Vyacheslav Chasnyk^1^, Sergei Avrusin^1^, Irina Solodkova^1^, Elena Sinelnikova^1^, Yaroslav Bobko^1^ and Tatiana Burtseva^2^
^1^Saint-Petersburg State Pediatric Medical University, Saint Petersburg, Russian Federation, sinelnikovae@gmail.com; ^2^Yakut Research Centre for Complex Medical Problems SB RAMS, Yakutsk, Russian Federation

#### 

*Introduction*. The term “inbreeding” refers to a descendant of close relatives. As a result of inbreeding in population, the number of homozygotes increases and the number of heterozygotes reduces, which degrades the quality of the population (inbreeding depression), since it increases the frequency of diseases associated with recessive genes. Decrease in genetic diversity, as a result of inbreeding, leads to that the individual and the population lose their ability to adapt to changing environmental conditions. The inbreeding coefficient can be determined by analysing the family tree based on the number of ancestors to the shared ancestor for both parents. *Objectives*. The purpose of this study was to determine the inbreeding coefficient by means of the analysis of isonymy in the population of natives – inhabitants of Yamal and Gyda peninsula. *Materials and methods*. The frequency patronymic names of 2043 Nenets and Khanty children living in the villages of the Yamal-Nenets autonomous region of Russia as well as in tundras assigned to the boarding schools located in the villages of Salemal, Panaevsk, Yar-Sale, Novy Port and Gyda were analysed. Coefficient of inbreeding was calculated (1) to estimate the frequency of consanguineous marriages. *Results*. The coefficient of random inbreeding for inhabitants of Yamal and Gyda tundras varies from 0.02 to 0.058. In the population of Gyda tundra, we revealed the highest coefficient of random inbreeding (0.058). For one of the surnames, the highest frequency of prospective consanguineous marriages was revealed (0.189). *Conclusion*. The coefficient of inbreeding as measured by isonymy in the child population of Yamal and Gyda tundras is much higher than, for example, in the population of Yakutia (0.0007) (2) or in the population of Kirovsk region (0.00321–0.01063) (3). In the child population of Gyda tundra, it is the third world‘s largest value published to date.

**References**

1. Crow, JF, Mange AP. Measurement of inbreeding from the frequency of marriages between persons of the same surname. Eugenics Q. 1965;12:199–203.

2. Kucher A et al., The structure of marriages of Yakut populations: ethnic composition and isonymic inbreeding, Genetics. 2010;46:408–16.

3. Kadyshev VV. Epidemiology, clinical and genetic features of the self-contained inherited ophthalmic pathology in Kirovskii region. [PhD dissertation], Moscow 2011 (in Russian).

## Behavioural health aides in Rural Alaska: their experience in caring for Alaska Native cancer survivors

### Stacy Kelley, Xiomara Owens and Christine DeCourtney
Alaska Native Tribal Health Consortium, Anchorage, AK, USA, sfkelley@anthc.org

#### 

*Purpose*. The Alaska Native Tribal Health Consortium conducted a statewide survey of rural community behavioural health aides (BHAs) within the tribal health system to assess their need for psychological and emotional support training for their work with cancer survivors. *Background*. Cancer is the leading cause of death among Alaska Native people with rates of many types of cancer exceeding that seen in the US white population. Cancer survivors may face numerous physical, psychological, spiritual and social challenges. BHA education generally focuses on substance abuse, grief, depression and suicide prevention. *Methods*. An electronic survey was distributed to all 114 rural BHAs in Alaska. They were asked about cancer survivors living in their community, whether they had been called up to provide counselling to those survivors and about their comfort level in addressing cancer-related emotional issues and concerns experienced by the patients and their families. *Outcomes*. Sixty-one (54%) BHAs responded, 62% of whom knew of cancer survivors in their community and 88% of whom agreed that it is their job to provide support to those cancer survivors. Of the 47% of BHAs who had provided counselling to cancer survivors, 63% noted lack of adequate training about how to provide that counselling. Dealing with “emotional concerns” was reported as the most difficult issue. Almost all (98%) reported they would likely participate in training to improve counselling skills. *Conclusion*. Most BHAs in rural Alaska know of a cancer survivor in their community and may be called on to provide mental health services, but few report adequate training in how to provide these services. Given the remote locations in which many BHAs work and the lack of local resources to guide them, more education is needed about how to support cancer survivors. This study provided information to help guide development of content of that education.

## Cannabis use in relation to obesity and insulin resistance in the Inuit population

### Michel Lucas^1^, Gerard Ngueta^1^, Richard Bélanger^1^ and Elhadji A. Laouan-Sidi^2^
^1^Laval University, Québec, Canada, michel.lucas@crchuq.ulaval.ca; ^2^Population Health and Optimal Health Practices Research Unit, Centre hospitalier universitaire de Québec (CHUQ) Research Centre, Québec, Canada

#### 

*Objective*. To ascertain the relationship between cannabis use, obesity and insulin resistance. *Methods*. We analysed data on 786 Inuit adults from the Nunavik Inuit Health Survey (2004). Information on cannabis use was obtained from a self-completed, confidential questionnaire. Fasting blood glucose and insulin, and homeostasis model assessment of insulin resistance (HOMA-IR) served as surrogate markers of insulin resistance. Analysis of covariance and multivariate logistic regression ascertained relationships between cannabis use and outcomes. *Results*. Cannabis use was highly prevalent in the study population (57.4%) and was statistically associated with lower body mass index (BMI) (P<0.001), lower% fat mass (P<0.001), lower fasting insulin (P=0.04) and HOMA-IR (P=0.01), after adjusting for numerous confounding variables. Further adjustment for BMI rendered fasting insulin and HOMA-IR differences statistically non-significant between past year cannabis users and non-users. Mediation analysis showed that the effect of cannabis use on insulin resistance was indirect, through BMI. In multivariate analysis, past year cannabis use was associated with 0.56 lower likelihood of obesity (95% confidence interval 0.37–0.84). *Conclusions*. Cannabis use was associated with lower BMI, and such an association did not occur through the glucose metabolic process or related inflammatory markers. The association between cannabis use and insulin resistance was mediated through its influence on weight.

## The commercialization of country food and food security: the case of Greenland and what Nunavut can learn

### Joanna Petrasek MacDonald^1^, James Ford^1^, Catherine Huet^1^, MacRury Allison^2^ and Sara Statham^2^
^1^McGill University, Montréal, Canada, joannamacdonald08@gmail.com; ^2^Government of Nunavut, Iqaluit, Canada

#### 

Food security exists “when all people, at all times, have physical, social and economic access to sufficient, safe and nutritious food to meet their dietary needs and food preferences for an active and healthy life” (1). Access to adequate food has been identified as a major challenge in the Canadian Arctic, particularly for Inuit communities, where levels of food insecurity are consistently higher compared to southern Canada (2). In Greenland, preliminary studies indicate relatively secure food systems in communities of comparable size to those in Nunavut, albeit with emerging stresses in the light of climatic and socio-economic change. Qualitative work has pointed to the potential of country food markets in providing relatively affordable sources of traditional food in communities (3). Herein, open air traditional food markets (kalaaliiaraq) are common in Greenland in communities of diverse sizes, providing both a source of economic returns for hunters and secure access to food for those in waged employment or who do not have access to networks through which food is shared. In the light of the relative security of food systems in Greenland, the project presented here investigates the role of commercial country food markets in enhancing food security within Greenland and explores whether or not it is feasible to develop and promote similar markets in a Nunavut context. The project used a systematic literature review alongside semi-structured, in-depth interviews with decision makers, civil society organizations, the Kalaallit Nunaani Aalisartut Piniartullu Katuffiat (Organization of Hunters and Fishermen in Greenland), researchers and representatives of Inuit organizations (e.g. ICC) in Nuuk, Copenhagen and Iqaluit. The operation of commercial country food markets in Greenland, their impact on food security and transferable lessons from the Greenland experience will be discussed.

**References**

1. FAO. Declaration of the World Summit on food security. Proceedings of World Summit on Food Security, Rome, Italy; 2009.

2. Huet C, Rosol R, Egeland GM. The prevalence of food insecurity is high and the diet quality poor in Inuit communities. J Nutr. 2012;142:541–7.

3. Ford JD, Goldhar C. Climate change vulnerability and adaptation in resource dependent communities: a case study from West Greenland. Clim Res. 2012;54:181–96.

## Body mass index of First Nations children and youth on first entering Canadian Prairie Residential Schools – 1919 to 1953

### Paul Hackett, Sylvia Abonyi and Roland Dyck
University of Saskatchewan, Saskatoon, Canada, sylvia.abonyi@usask.ca

#### 

Historical research documenting nutrition experiments performed on First Nations (FN) residential school children in Canada from the 1940s indicates that students experienced hunger and malnutrition and suffered from overall poor health (1). Using cross-sectional residential school entrance examination data collected between 1919 and 1953, we report here on the BMIs of 1767 Canadian Prairie FN children. This is significant because it reflects conditions in their home communities rather than in the schools. Examinations captured height and weight data as well as general qualitative observations on the health of the child. Following (2,3), age-specific BMIs were calculated and categorized as underweight, normal weight and overweight/obese by age, sex, time period and residential school site. Height and weight quartiles were compared with a 1953 Canadian survey and BMIs with current WHO growth charts for Canadian children. On admission to residential school, FN children were more likely to have normal BMIs than Canadian children today, and to have lower rates of overweight/obesity and higher rates of underweight. FN children tended to be slightly taller than non-FN Canadian children from the 1953 survey, but shorter than Canadian children today. Most weights for older FN children were within the 25th and 75th percentiles of non-FN Canadian children from the 1953 survey. A general trend for diminishing levels of underweight and increasing levels of overweight/obesity over time was observed. Highest rates of underweight occurred before the depression. Those attending residential schools in the southern prairies were more likely to be underweight. These findings are consistent with reports of malnutrition in some communities during the study period. Overall, however, results suggest that many FN children were not leaving their home communities in a malnourished state, unlike the poor health reported for children in attendance at the schools.

**References**

1. Mosby I. Administering colonial science: nutrition research and human biomedical experimentation in Aboriginal communities and residential schools, 1942–1952. Histoire Sociale/Social History. 2013;46:145–72.

2. Cole TJ, Flegal KM, Nicholls D, Jackson AA. Body mass index cut offs to define thinness in children and adolescents: international survey. BMJ. 2007;335:194–201.

3. Cole TJ, Bellizzi MC, Flegal KM, Dietz WH. Establishing a standard definition for child overweight and obesity worldwide: international survey. BMJ. 2000;320:11240–3.

## Regional differences in subsistence practices and exposure to environmental contaminants and nutrients in Alaska’s Yukon-Kuskokwim Delta

### Marylynne Kostick^1^ and James Berner^2^
^1^Division of Subsistence, Alaska Department of Fish and Game, Juneau, AK, United States, mlkostick@hotmail.com; ^2^Alaska Native Tribal Health Consortium, Anchorage, AK, United States

#### 

Environmental contaminants are found throughout the environment, making their way into our dietary sources. Regional differences in lifestyle and access to dietary sources may influence a population’s exposure to dietary sources of environmental contaminants and nutrients. We present an overview of subsistence harvest practices and maternal and umbilical cord blood levels of selected contaminants and nutrients in coastal and riverine communities from the Yukon-Kuskokwim Delta. Data for this study come from the Alaska Department of Fish and Game, Division of Subsistence Community Subsistence Information System and the Alaska Maternal Organics Monitoring Study, a community-based participatory research study developed for the Yukon-Kuskokwim Health Corporation by the Alaska Native Tribal Health Consortium. Results present information on public health issues in rural Alaska and assist in the biomonitoring of environmental contaminants and their effect on maternal and infant health in the circumpolar north.

## Addressing childhood food insecurity in Nunavut: a life cycle approach to diabetes prevention

### Amy Caughey
Harvest Nutrition Consulting, Stella Ontario, Canada, amy.caughey@gmail.com

#### 

Inuit were once considered protected from the metabolic consequences of obesity, and rates of diabetes in Inuit were low. However, the Inuit Health Survey (2007–2008) demonstrated that the prevalence of type 2 diabetes in Inuit living in Nunavut was comparable to rates observed in the general Canadian population. At the same time, the nutrition status of children in Nunavut was assessed revealing a high prevalence of household food Insecurity in children, with nearly 70% of Inuit preschoolers living in households rated as food insecure. Inuit children in Nunavut experience negative health outcomes that are impacted by poor nutrition, such as a high prevalence of obesity and micronutrient deficiency, including vitamin D deficiency. Traditional food has been shown to contribute to higher dietary quality and is an important food source for Inuit children, while market food consumed is often of poorer quality. Maternal nutrition, including food insecurity and gestational diabetes, impacts child growth and health, and has been identified as a concern among healthcare workers in Inuit communities. Achieving food security for children in Nunavut is not only a necessary foundation for public health and child development, but also a fundamental component of diabetes prevention.

## A research methodology for informing a dialogue on health systems improvements in circumpolar regions using indigenous knowledge

### Susan Chatwood
ICHR, North York, Canada, susan.chatwood@ichr.ca

#### 

The health services’ challenges experienced by indigenous peoples in circumpolar nations are complex and engage a broad and inter-related range of sectors, including health, environment, education, justice and traditional institutions outside government departments. Declarations such as the United Nations Declaration on the Rights of Indigenous Peoples have recognized the rights of indigenous peoples to maintain access to their traditional medicines and health practices, including the conservation of vital medicinal plants, animals and minerals. The UN declaration also calls for the right to access, without discrimination, all social and health services. Circumpolar nations have agreed to the terms of these declarations, but have recognized that these rights will require a stewardship model informed not only by currently valued evidence but also by an appreciation of diverse value systems and different sources of evidence. There has been a call to expand the research agenda across sectors responsible for health and well-being and to recognize academic and indigenous knowledge bases. Such a research approach requires systematic understandings and comparisons in order to gain insight into health-systems strengths and adaptations applicable in the circumpolar setting. This paper describes current understandings of methodological approaches and presents a novel approach of a mixed methods methodology to meet this challenge.

## Disparities in infectious disease hospitalizations among Alaska Native persons compared to Non-Alaska Native persons in Alaska, USA

### Prabhu Gounder^1^, Robert Holman^1^, Sara Seeman^1^, Jason Mehal^1^, Alice Rarig^2^, Mary McEwen^2^, Claudia Steiner^3^, Michael L. Bartholomew^4^ and Thomas W. Hennessy^1^
^1^Centers for Disease Control and Prevention, Atlanta, GA, USA, iym4@cdc.gov; ^2^Division of Public Health, Alaska Department of Health and Social Services, Anchorage, USA; ^3^ Healthcare Cost and Utilization Project, Center for Delivery, Organization and Markets, Agency for Healthcare and Research and Quality, Rockville, USA; ^4^Indian Health Service, Anchorage, USA

#### 

*Background*. For the first time, a comprehensive data set exists with both Alaska Native (AN) and non-AN persons hospitalized in Alaska, USA. This study aims to estimate the rate of infectious disease (ID) hospitalizations among AN and non-AN people living in Alaska and provide comparisons between the two groups. *Methods*. Hospital discharge data for IDs from Alaska’s Indian Health Service and the Alaska State Inpatient Database (includes all but one non-tribally operated acute care community hospital) were combined and analysed for 2010–2011. The ID hospitalizations were defined as records with a first-listed *International Classification of Diseases, 9th Revision, Clinical Modification code* for IDs. Age-adjusted and age-specific hospitalization rates per 100,000 persons were calculated using corresponding annual population data from the Alaska Department of Labor and Workforce Development; rates were directly adjusted using the 2,000 US population as the standard. Rates were compared using the *z*-test and Poisson regression. *Results*. Among AN people, 19% (6,531) of hospitalizations were for ID versus 12% (7,752) among non-AN people in Alaska. The age-adjusted annual ID hospitalization rate in AN persons (3,004) was higher than in non-AN persons (898; rate ratio [RR]=3.3; p<0.05). The ID hospitalization rate disparity between AN and non-AN people was greatest for children aged <1 year (RR=5.4; p<0.05) and 1–4 years (RR=4.2; p<0.05). The lower respiratory tract infections (LRTI) ID category accounted for 38% of ID hospitalizations in AN persons; the age-adjusted rate was higher for AN compared to non-AN persons (RR=4.6; p<0.05). *Conclusions*. The AN people experience a disproportionate burden of ID hospitalizations compared with non-AN people in Alaska. The disparity in the rate for ID hospitalizations among AN persons compared with non-AN persons was greatest for children aged <5. LRTI hospitalizations contributed the greatest to the burden of ID hospitalizations.

## Culturally relevant social and health services in enhancing the sustainability of reindeer herding

### Lidia Heikkilä
University of Lapland, Rovaniemi, Finland, Lydia.Heikkila@ulapland.fi

#### 

The role of social and health services in enhancing the sustainability of reindeer herding is less investigated compared to the socio-ecological, economic and socio-cultural factors. The resilience and sustainability of reindeer herding is the result of inextricably linked multilevel adaptation processes to various challenges, changes and disturbances, where it is difficult to separate one factor from another. Social and health factors may sometimes carry a major importance for the outcome by framing the functioning of the other mechanisms in a significant way. Social and health services have the power to regenerate, reinforce and redistribute the human and socio-economic resources. Social security and services provide support in changes of life situation, personal and social problems and income troubles of reindeer herders and their families. Health care, especially occupational health and health promotion, has an important role in sustaining and securing the workability of reindeer herders and provides the required rehabilitation. It is important that delivery and production of these services are suitable and relevant for the reindeer herders’ needs and life situations and contribute to the well-being of individuals, families and communities as well as to the sustainability of the livelihood.

## Health research in Nunavut: an Inuit governance perspective

### Natan Obed and Sharon Edmunds-Potvin
Nunavut Tunngavik Inc., Iqaluit, Canada, sedmunds-potvin@tunngavik.com

#### 

Research in Nunavut has a tainted legacy, characterized by uneven power relationships between Inuit and researchers. While great strides have been made in the area of participatory research, aspects of this legacy are still present in research occurring in the Nunavut Settlement Area. Further, research is increasingly viewed as an important means of exercising Inuit self-determination, by communities and institutions of Inuit governance in Nunavut – this statement holds true for Nunavut Tunngavik Incorporated, the holder of the Nunavut Land Claims Agreement. Research has the ability to positively impact the lives of Inuit in Nunavut, particularly when research results meaningfully inform interventions and policy. The latter, from an Inuit governance perspective, is best achieved through co-driven research that is participatory in nature. Essentially when Inuit institutions, including Governments, are invested in the research question(s) and co-shaping the research with academic partners, our entire society can benefit. A new research reality is emerging in Nunavut with strong emphasis being placed on meaningful engagement – this includes the conceptualization phase and ranges to the dissemination of results and formulating the narrative.

## Suicides in the indigenous and non-indigenous populations of the Nenets Autonomous Okrug, Russia

### Yury Sumarokov, Tormod Brenn, Kudryavtsev Alexander and Nilssen Odd
UiT – The Arctic University of Norway, Tromsø, Norway, ysu002@post.uit.no

#### 

*Objective*. To explain suicide rates in the indigenous and non-indigenous populations of the Nenets Autonomous Okrug (NAO) in 2002–2012 with associated socio-demographic characteristics. *Design*. Retrospective population-based mortality study. *Methods*. Data from autopsy reports were used to identify 252 cases of suicide in the NAO in 2002–2012. Socio-demographic data of these cases were obtained from passports and medical records and then were linked to total population data from the censuses in 2002 and 2010. Suicide rates for the indigenous Nenets population and the non-indigenous population were calculated according to different socio-demographic characteristics. Corresponding relative risks for each population group were compared. *Results*. The crude standardized suicide rates were 72.7 per 100,000 person-years in the Nenets and 50.7 per 100,000 person-years in the non-indigenous population. Corresponding average suicide rate taken from Russian mortality data in 2002–2012 was 36.0 per 100,000 person-years. Socio-demographic characteristics associated with high-suicide rates for the Nenets were 20–39 years of age, male gender, urban residence, having secondary school or higher education, being employed and being single or divorced. Males aged 20–29 years and females aged 30–39 years and individuals aged 70 years or over had the highest suicide rates in the non-indigenous population. The high-suicide rates in the non-indigenous population were associated with male gender, rural residence, secondary school education, being an employee or employer and being single or divorced. *Conclusions*. Suicide rates in the NAO were higher than nationwide. Suicide rates were higher among the indigenous Nenets population than the non-indigenous population and were associated with different socio-demographic characteristics. This information is useful for targeted suicide prevention in different population groups in the Arctic.

**References**

1. Voitseh V. Problemy Suicida u Korennyh Narodov Severa, Vtoraya Letnyay Shkola po Meditsinskoy Antropologii. Moscow: Institute of Anthropology and Ethnography, Russian Academy of Sciences; 2009.

2. Young TK, Revich B, Soininen L. Suicide in circumpolar regions: an introduction and overview. Int J Circumpolar Health. 2015;74:103402. doi: http://dx.doi.org/10.3402/ijch.v74.27349

3. Sumarokov YA, Brenn T, Kudryavtsev AV, Nilssen O. Suicides in the indigenous and non-indigenous populations in the Nenets Autonomous Okrug, Northwestern Russia, and associated socio-demographic characteristics. Int J Circumpolar Health. 2014;73:24308. doi: http://dx.doi.org/10.3402/ijch.v73.24308

## D. Working in the Circumpolar Regions: Accidents and Injuries

### Young people and risk communication related to snowmobiling in Northern Norway: a focus group study

#### Grete Mehus, Sidsel Germeten and Henriksen Nils
University of Tromsø, Tromsø, Norway, grete.mehus@uit.no

##### 

This study aims to understand the communication of the risks of snowmobiling among Northern Norwegian youths. A qualitative design with focus-group interviews was chosen. Interviews centred on safety precautions and estimation of risks related to snowmobiling and driving patterns. Eighty-one students (31 girls and 50 boys) aged between 16 and 23 years from eight high schools were interviewed in 17 focus groups segregated by gender. Interview data were analysed using qualitative content analysis. Boys and girls communicated differently about risks. Peer-group conformity appeared stronger among boys than girls. Boys focused upon training, coping and balance between control and lack of control while driving. Girls talked about risks, were aware of risks and sought to avoid risky situations, in contrast to boys. Youths are familiar with accidents and know how to prevent them. Boys’ risk communication in groups was about how to manage challenging situations and how to maintain control while simultaneously testing the limits. Three risk categories emerged: those who drive as they ought to (mostly girls), those who occasionally take some risks (boys and girls) and some boys who are extreme risk-takers. Perceptions of and communication about risk are related to gender, peer-group and familiarity with risk-taking when snowmobiling. Northern Norwegian boys’ driving behaviour highlights a specific need for risk reduction. According to international research, there are some important safety recommendations for snomobilers: 1) Use safety equipment, helmet and well-insulated clothes, 2) do not drink and drive, 3) follow the trails and the rules – it is for your own safety, 4) avoid night driving, mountain climbing and showing off, 5) be aware of young boys – they are in the risk zone for accidents, 6) unfamiliar areas demand extra attention, 7) let someone know where you are going and when you will return, short trips can be long – be prepared and 8) small children are not allowed to drive.

**References**

1. Sy ML, Corden TE. The perils of snowmobiling. WMJ. 2005;104:32–4.

2. Grete M, Sidsel G, Henriksen N. Youth, snowmobiling and the ‘snowmobile feeling’. Tidsskrift for ungdomsforskning. 2010;10:39–56.

3. Douglas M. Risk acceptabillity according to the social sciences. New York: Russel Sage Foundation; 1985.

## Creating a collective narrative of social workers’ experiences in isolated, northern communities: a collaboration for support and change

### Kaila de Boer
McGill University School of Social Work, Montreal, Québec, Canada, kaila.deboer@mail.mcgill.ca

#### 

There is a growing recognition of the need for and challenges to health and social service work in remote areas. Of leading concern is the high rate of worker turnover that impedes service delivery and is costly (1). Within this context of high turnover, promoting retention is a top priority; research has explored ways of increasing worker retention including exploring worker characteristics, which are associated with retention; incentives, which encourage workers to remain; and employer factors and initiatives, which impact worker (1,2,3). Linked to the high turnover, research has found that many rural health and social service workers experience high levels of personal and professional stress (3). Building upon this research, in addition to knowledge gained through personal experience working within rural contexts, this project proposes a Participatory Action Research project involving social workers and allied professionals in the small, isolated, Northern communities of Labrador, Canada, to construct a collective narrative of the experiences of providing health and social services in the region and create a space for collaboration for change. Specifically, the intents of this project are threefold: to begin a dialogue on the experiences of working in the small, isolated, Northern communities of Labrador to reduce isolation; to develop a collective narrative out of these experiences such that the experiences can be shared without risking identifying particular individuals; and to foster an environment of collaboration should there be a desire for a mobilization for change. This poster will reflect the current proceedings of this research project including the detailed literature review, specific research questions, project structuring and ethical procedures, and any preliminary results (data collection expected to begin in May 2015).

**References**

1. Campbell N, McAllister L, Eley D. The influence of motivation in recruitment and retention of rural and remote allied health professionals: a literature review. Rural Remote Health. 2012;12:1900.

2. Chisholm M, Russell D, Humphreys J. Measuring rural allied health workforce turnover and retention: what are the patterns, determinants and costs? Aust J Rural Health. 2011;19:81–8. doi: http://dx.doi.org/10.1111/j.1440-1584.2011.01188.x

3. Hunsberger M, Baumann A, Blythe J, Crea M. Sustaining the rural workforce: nursing perspectives on worklife challenges. J Rural Health. 2009;25:17–25. doi: http://dx.doi.org/10.1111/j.1748-0361.2009.00194.x

## Whose head hurts in Alaska? TBI trends and disparities 1992–2011

### Hillary Strayer^1^, Jill Hodges^2^, Jaylene Wheeler^1^ and Gary Ferguson^1^
^1^Alaska Native Tribal Health Consortium, Anchorage, AK, USA, gferguson@anthc.org; ^2^Alaska Brain Injury Network, Inc., Anchorage, AK, USA

#### 

According to the Centers for Disease Control and Prevention, the Alaska Native/American Indian (AN/AI) population has one of the highest rates of traumatic brain injury (TBI) in the United States. People who acquire a TBI can experience severe or long-term life changes. Understanding how best to use limited funding for prevention and care is critical. This project determined the magnitude of TBI occurrence in Alaska and how it changed over time. It identified disparities based on demographic characteristics, activity at the time of injury and alcohol involvement. The State of Alaska Trauma Registry provided data on all injury hospitalizations in Alaska from 1992 to 2011. For the time trend, rates were calculated in 4-year intervals from 1992 to 2011. For other rates, the most recent 5 years (2007–2011) were examined. From 2007 to 2011, there were 3,353 hospitalizations in Alaska for TBI out of a total 22,669 injury hospitalizations (15%). Over the full 20-year period examined (1992–2011), only non-Native males showed a significant decrease in the rate of TBI hospitalizations (p<0.05). Mean injury hospitalization costs were $11,000 higher for TBI cases than other injury cases ($37,800 vs. $26,900, respectively). AN/AIs have higher rates of TBI than non-Natives in Alaska. Alaska has limited post-acute TBI rehabilitation; 66% of Alaskans hospitalized with TBI go home with no assistance. In rural Alaska, rehabilitation resources are nearly non-existent. More research is needed to understand reasons for the disparities seen and the social costs of limited rehabilitation and community supports. These data support advocacy efforts for TBI issues; provide policy makers and leaders a better scope of these injuries; and are intended to improve prevention, trauma care, community rehabilitation and ongoing supports.

## E. Environmental Health

### Habitual wintertime cooling and blood pressure in hypertensive and normotensive men: an experimental study

#### Heidi Hintsala^1^, Jouni J.K. Jaakkola^1^, Juhani Hassi^1^, Riitta Antikainen^2^ and Tiina M. Ikäheimo^3^
^1^Center for Environmental and Respiratory Health Research and MRC Oulu, University of Oulu, Oulu, Finland, heidi.hintsala@oulu.fi; ^2^Institute of Health Sciences, University of Oulu, Oulu, Finland, ^3^Center for Environmental and Respiratory Health Research and MRC Oulu, University of Oulu, Oulu, Finland

##### 

Short-term exposure to cold robustly increases blood pressure (BP) and cardiovascular strain (1,2), which could relate to the reported higher wintertime cardiovascular morbidity and mortality. The aim of our study was to examine brachial BP, heart rate (HR) and HR variability (HRV) among persons whose systolic BP level or response is high in cold. We conducted a population-based recruitment of 83 men (55–65 years), which included home BP measurement to distinguish hypertensive (n=51) and normotensive subjects. Electrocardiogram and brachial BP were recorded in control conditions (18°C, 15 min) and whole-body cold exposure restricted mainly to face (−10°C, winter clothes, 15 min). HRV was computed on low-frequency (LF, 0.04-0.15 Hz) and high-frequency (HF, 0.15-0.4 Hz) bands. We compared the results between 90th percentile of systolic BP level and response to others. Cooling increased systolic BP above 200 mmHg in 13% of subjects (1 normotensive). Systolic BP increased more than 60 mmHg in 10% of both hypertensive and normotensive men. Home-measured systolic BP (146±14 mmHg vs. 133±14 mmHg, p<0.01) and control HF HRV [93 ms2 (47,222) vs. 54 ms2 (27,120), p<0.05] were higher in men whose BP increased above 200 mmHg compared to the others. Also, men whose BP increased more than 60 mmHg had higher HF HRV [119 ms2 (69,204) vs. 54 ms2 (29,121), p<0.05] and lower HR [63 bpm (58,69) vs. 79 bpm (70,87), p<0.01] in control conditions than the others. Cold exposure corresponding to habitual winter circumstances, and restricted mainly to the face, increases systolic BP above 200 mmHg and more than 60 mmHg in middle-aged men. Those with high BP in cold were more likely to have higher BP in home, cardiac vagal activity in control conditions and increased cold-related BP response. Interestingly, an aggravated cold-induced increase in BP occurred in both hypertensive and normotensive men. Health care specialists should be aware of the remarkable rise in BP related to everyday winter conditions.

**References**

1. Hintsala H, Kandelberg A, Herzig KH, Rintamäki H, Mäntysaari M, Rantala A, et al. Central aortic blood pressure of hypertensive men during short-term cold exposure. Am J Hypertens. 2014;27:656–64.

2. Hintsala H, Kenttä TV, Tulppo M, Kiviniemi A, Huikuri HV, Mäntysaari M, et al. Cardiac repolarization and autonomic regulation during short-term cold exposure in hypertensive men: an experimental study. PLoS One. 2014;9:e99973. doi: http://dx.doi.org/ 10.1371/journal.pone.0099973

## Human rhinovirus infections are associated with temperature and humidity in a cold climate

### Kari Jaakkola^1^, Annika Saukkoriipi^2^, Jari Jokelainen^3^, Merja Roivainen^4^, Jouni Jaakkola^5^ and Tiina Ikäheimo^5^
^1^Finnish Defence Forces, Helsinki, Finland, kari.jaakkola@mil.fi; ^2^National Centre for Health and Wellbeing, Helsinki, Finland; ^3^Centre for Lifecourse Epidemiology, University of Oulu, Oulu, Finland; ^4^National Institute for Health and Wellbeing, Helsinki, Finland; ^5^Center for Environmental and Respiratory Health Research, University of Oulu, Oulu, Finland

#### 

*Objective*. Both temperature and humidity may independently or jointly contribute to the risk of human rhinovirus (HRV) infections either through the effects of climatic factors on the survival and spread of viruses in the environment, or due to changes in population susceptibility to acute respiratory infections. We examined the relations between the level and decrease of temperature, humidity and the risk of HRV infections in a subarctic climate during military training. In these conditions, both cold exposure and exercise may predispose subjects to respiratory infections (1,2). *Methods*. We conducted a case-crossover study among military conscripts (KIAS-study, n=892) seeking medical attention due to respiratory symptoms during their military training period and identified 146 HRV cases by PCR. Meteorological data such as measures of average and decline in ambient temperature and absolute humidity (AH) during the three preceding days of the onset (hazard period) and two reference periods, prior and after the onset, were obtained. *Results*. The average temperature preceding HRV onset was −9.9±4.9°C and AH 2.2±0.9g/m^−3^. Both an average [OR 1.07 (95% CI 1.00–1.15] and maximal [OR 1.08 (1.01–1.17)] change in temperature increased the risk of HRV infections by 8% per 1°C decrease. Furthermore, average [OR 1.20 (1.03–1.40)] and maximal decrease [1.13 (0.96–1.34)] in AH increased the HRV infection risk by 13 and 20% per 0.5 g decrease in humidity. A higher average temperature during the three preceding days was positively associated with HRV infections [1.07 (1.00–1.15)]. *Conclusions*. A decrease rather than low temperature and humidity per se during the three preceding days increases the risk of HRV infections in a cold climate. The information is applicable for proper prevention and protection from cold-related adverse health effects.

**References**

1. Jaakkola K, Saukkoriipi A, Jokelainen J, Juvonen R, Kauppila J, Vainio O, et al. Decline in temperature and humidity increases the occurrence of influenza in cold climate. Environ Health. 2014;13:22. doi: http://dx.doi.org/10.1186/1476-069X-13-22

2. Mäkinen TM, Juvonen R, Jokelainen J, Harju TH, Peitso A, Bloigu A, et al. Cold temperature and low humidity are associated with increased occurrence of respiratory tract infections. Respir Med. 2009;103:456–62. doi: http://dx.doi.org/10.1016/j.rmed.2008.09.011

## Asthma control and cold-related respiratory symptoms

### Henna Hyrkäs, Tiina Ikäheimo, Jouni Jaakkola and Maritta Jaakkola
University of Oulu, Oulu, Finland, henna.hyrkas@oulu.fi

#### 

*Background*. In the Northern Hemisphere, people are exposed recurrently to cold air, and previous studies have shown that asthmatics experience respiratory symptoms in cold more than people without asthma (1,2). The association between asthma control and the occurrence of cold-related respiratory symptoms has not been studied previously. We hypothesized that subjects with poor asthma control are more prone to experience cold-related symptoms than those with good asthma control. *Methods*. The Northern Finnish Asthma Study (NoFAS) was initiated in 2012 as a population-based cross-sectional study of 1995 17- to 73-year-old adults with asthma living in the Northern Finland. Cold-related respiratory symptoms (shortness of breath, cough, wheezing, phlegm production and chest pain) as well as questions related to asthma control were assessed by a questionnaire. The asthma control test (ACT) was defined based on five questions (disadvantage and occurrence of asthma symptoms, waking up for asthma symptoms, use of rescue medication and self-assessment of asthma control during the past 4 weeks) and was divided into quartiles where the highest represented complete control of asthma. *Results*. Reporting of cold-related respiratory symptoms was higher among asthmatics with poorly controlled asthma (ACT Q1 vs. ACT Q4): adjusted prevalence ratio (PR) for shortness of breath (men 1.47, 95% confidence interval 1.21–1.77; women 1.18, 1.07–1.30), cough (men 1.10, 0.91–1.34; women 1.18, 1.08–1.30), wheezing (men 1.91, 1.31–2.78; women 1.48, 1.17–1.87), phlegm production (men 1.51, 1.06–2.14; women 1.62, 1.27–2.08) and chest pain (men 4.47, 1.89–10.56; women 2.60, 1.64–4.12). The occurrence of symptoms increased with worsening asthma control. *Conclusions*. Our study indicates that subjects whose asthma is poorly controlled are more prone to cold- or weather-related respiratory symptoms, and even a slight worsening of asthma control increases symptom prevalence.

**References**

1. Harju T, Mäkinen T, Näyhä S, Laatikainen T, Jousilahti P, Hassi J. Cold-related respiratory symptoms in the general population. Clin Respir J. 2010;4:176–85.

2. Hyrkas H, Jaakkola MS, Ikaheimo TM, Hugg TT, Jaakkola JJ. Asthma and allergic rhinitis increase respiratory symptoms in cold weather among young adults. Respir Med. 2014;108:63–70.

## The impact of a polar environment on urinary status – an investigation of urinary status in the 54th Japanese Antarctic Research Expedition

### Atsushi Ikeda^1^, Koji Kawai^2^, Masakazu Tsutsumi^1^, Koji Yoshimura^3^, Giichiro Ohno^4^, Tatsuhisa Hasegawa^5^ and Oe Hirofumi^5^, Watanabe Kentaro^6^ and Hiroyuki Nishiyama^2^
^1^Department of Urology, Hitachi General Hospital, Hitachi, Japan, scrapsike716@gmail.com; ^2^Department of Urology, Institute of Clinical Medicine, University of Tsukuba, Tsukuba, Japan; ^3^Department of Urology, Shizuoka General Hospital, Shizuoka, Japan; ^4^Department of Surgery, Tokatsu Hospital, Matsudo, Japan; ^5^The 54th Japanese Antarctic Research Expedition, Japan; ^6^National Institute of Polar Research, Tokyo, Japan

#### 

*Background*. A number of factors have been linked to urinary disorders, but there are few reports on the urinary status of people living in unusual climates, such as a polar environment. With the aim of elucidating the impact of the midnight sun, polar nights and confined living conditions in the Showa Station on urination, we investigated the urinary status of members of the Japanese Antarctic Research Expedition (JARE). *Subjects and methods*. This was a prospective study conducted on 20 members of the 54th JARE (15 members of the winter party and 5 members of the summer party) who had given consent. The participants answered questionnaires concerning urination [International Prostate Symptom Score (IPSS), Overactive Bladder Syndrome Score (OABSS) and the Pittsburgh Sleep Quality Index (PSQI)] and kept daily voiding diary (for 3 days). We analysed the data of 12 members of the winter party who had responded to the questionnaires more than three times before departure and during their stays in Antarctica. *Results*. Compared to before departure, the mean scores for all of the questionnaires decreased during their stays in Antarctica, from 3.42 to 2.31 points for IPSS, from 1.25 to 0.986 points for OABSS and from 4.58 to 3.78 points for PSQI, but the mean volume voided per micturition increased from 213 to 233 ml. In regard to the amount of change in IPSS scores over the same period, a significant difference was noted between seven members with a pre-departure score ≥3 points and five members with a pre-departure score <3 points (−2.22 vs. 0.448, p=0.0245). *Conclusions*. This study is the first to report the urinary status of people in a polar environment investigated with questionnaires and voiding diaries. We initiated this study under the assumption that one’s urinary status would deteriorate in a polar environment, but our findings indicated that urination and sleep tend to improve in Antarctica.

## Making a tobacco-free hospital campus: an Alaska Native tribal health system experience

### Karen Doster, Stacy Kelley and Gary Ferguson
Alaska Native Tribal Health Consortium, Anchorage, AK, USA, gferguson@anthc.org

#### 

*Background*. According the State of Alaska (2014), more than twice as many Alaska Native people are estimated to be current smokers than non-Native Alaskans (38% vs.18%) and over three times as many smokeless tobacco users (15% vs. 4%). Cancer is the leading cause of death in Alaska Native people. The decision to implement a tobacco-free policy for the Alaska Native Medical Center campus originated with tribal leadership when presented with data about health impacts and costs of tobacco use. The Alaska Native Tribal Health Consortium (ANTHC) Board of Directors wanted to provide a supportive environment for employees and patients consistent with the mission and vision of ANTHC. *Methods*. Promising tobacco policies were researched to develop the tobacco-free policy that was implemented in November 2006. Work groups addressed needs for human resources, remote sites, patient needs, public relations and enforcement. ANTHC has subsequently offered technical assistance to regional tribal health facilities interested in adopting tobacco policies and system changes. *Results*. Since implementation, seven regional tribal health facilities have implemented tobacco-free campus policies. Additional health promotion policy changes at ANTHC and other tribal health corporations include the banning of sweetened beverages and the adoption of traditional foods served in hospital cafeterias. There is widespread support for clean indoor air policies throughout tribal country. The proportion of Alaska Native adults who agree that smoking should not be allowed in indoor work areas has increased from 73.2% in 1998 to 86.0% in 2011 (1). *Conclusions*. Tribes within the Alaska Tribal Health System and their board of directors understand and support tobacco-free policy efforts. Tobacco policies have subsequently led to the implementation of other health-related policies for Alaska Native tribal organizations.

**Reference**

1. State of Alaska, Department of Health and Social Services, “Alaska Tobacco Facts: 2014 Update.” 2014, http://dhss.alaska.gov/dph/Chronic/Documents/Tobacco/PDF/2014_alaska_tobacco_facts.pdf

## Characterization of environmental health properties of a fabricated polymeric fungi-based insulation material

### Philippe Amstislavski and Zhaohui (Joey) Yang
University of Alaska, Anchorage, AK, USA, pamstislavski@alaska.edu

#### 

*Significance*. Plastic polymers are commonly used for thermal insulation in infrastructure and housing in Circumpolar North. These materials may impact indoor air quality in buildings and are non-renewable. Their production and use involve substantial energy inputs and associated toxic wastes, presenting a well-documented global environmental problem. We found that when introduced into a nutrient-rich matrix, the mycelium of certain cold-resilient fungi bounds the matrix and produces a foam-like, lightweight, insulating composite. This renewable material has no chemical binders and may have excellent, but untested, applications as environmentally sound thermal insulation that could substantially reduce environmental health impacts of construction on permafrost. Using bench-top testing data, we will report on several environmental health aspects of using the new material. *Objectives*. Recognizing the growing demand for renewable, non-toxic thermal insulation materials, this study focuses on testing the key properties for fungi insulation material. Our objectives are to (1) test laboratory protocols for fungi inoculum and produce wood pulp matrices that afford rapid and sustained growth of the biologically active fungi inoculum, (2) produce composites using of cold-resilient fungi cultures and (3) test basic properties related to potential health implications of using the resultant fungi composites. *Methodology*. This study focuses on data from bench-top experiments. Key tasks include combining wood pulp, nutrient source, binding agent and the inoculum of white-rot fungi; incubating it under controlled conditions; and measuring thermal conductivity, fire rating, and volatile organic compounds (VOCs) of the resultant material. Full report documenting the findings will be provided. *Potential impact*. This study will provide initial data on health aspects of using fungi-based insulation, needed for future development of environmentally sound thermal insulations for circumpolar regions.

**References**

1. Novotny C, Cajthaml T, Svobodova K, Susla M, Sasek V. Irpex lacteus, a white-rot fungus with biotechnological potential – review. Folia Microbiol. 2009;54:375–90.

2. Arnot JA, Armitage JM, McCarty LS, Wania F, Cousins IT, Toose-Reid L. Toward a consistent evaluative framework for POP risk characterization. Environ Sci Technol. 2011;45:97–103.

3. Eben Bayer GM. Method for producing rapidly renewable chitinous material using fungal fruiting bodies and product made thereby. The United States Patent and Trademark Office; 2011.

## Birthweight and temperature on the day of conception

### Juhani Leppäluoto and Simo Näyhä
University of Oulu, Oulu, Finland, simo.nayha@oulu.fi

#### 

*Background*. The season of conception influences pregnancy outcome. In most studies, low outdoor temperatures have been found to be associated with low birthweight at term. Previous studies have used temperatures as monthly or seasonal averages. We have analysed the association of mean daily temperature on the day of conception with birthweight in Finland using a national database. *Methods*. Information on all 549,609 births that occurred in Finland during the period 1999–2008 was obtained from the Finnish Medical Birth Register. Mean daily temperatures were averaged over four meteorological stations (Jokioinen, Jyväskylä, Kajaani and Rovaniemi) and used as estimates of temperature on the day of conception. Birthweight was regressed on linear splines of temperature using quartiles of temperature as knots. The results were calculated for full-term (37–42 weeks) and pre-term infants (<37 weeks), separately for boys and girls and for infants of younger (age <30 years) and older (age ≥30) mothers. *Results*. The decline of temperature over the whole temperature range (+24°C to −31°C) was associated with a decline in birthweight of 125 g (95% confidence interval: 41–210) but only in pre-term infants. The decline was greater in girls (138 g [14–261]) than boys (118 g [3–233]) and greater in infants of older mothers (146 g [27–264]) than in those of younger mothers (102 g [19–223]). No decline of birthweight depending on temperature on the day of conception was observed in full-term infants. *Discussion*. Contrary to previous studies, we observed no significant changes in birthweights of term infants within the wide seasonal range of outdoor temperatures. However, we did observe reductions of 102–146 g in birthweight of preterm infants conceived on the coldest days compared with those conceived on the warmest days. This may be associated with low maternal serum LH and oestrogens and high-cortisol levels during the cold season which may slow foetal growth.

**References**

1. Kivelä A, Kauppila A, Ylöstalo P, Vakkuri O. Seasonal, menstrual and circadian secretions of melatonin, gonadotropins and prolactin in women. Acta Physiologica Scandinavica. 1988;132:321–7.

2. Lawlor DA, Leon DA, Davey Smith G. The association of ambient temperature throughout pregnancy and offspring birthweight: findings from the Aberdeen Children of the 1950s cohort. Int J Obstet Gynaecol. 2005;112:647–57.

3. Rantakallio P. The effect of a northern climate on seasonality of births and the outcome of pregnancies. Acta Paediatr Scand Suppl. 1971;218:1–67.

## Heat-related thermal sensation, comfort and symptoms in a northern population: the National Finrisk 2007 study

### Simo Näyhä^1^, Hannu Rintamäki^2^, Gavin Donaldson^3^, Juhani Hassi^1^, Pekka Jousilahti^4^, Tiina Laatikainen^4^, Jouni Jaakkola^1^ and Tiina Ikäheimo^1^
^1^University of Oulu, Oulu, Finland, simo.nayha@oulu.fi; ^2^Institute of Occupational Health, Oulu, Finland; ^3^Imperial College London, United Kingdom; ^4^National Institute for Health and Welfare, Oulu, Finland

#### 

*Background*. The occurrence of subjective symptoms related to heat strain in the general population is unknown. The present study aimed to describe the temperatures considered to be comfortable or hot, and the prevalence of heat-related complaints and symptoms in the Finnish population. *Methods*. A total of 4007 men and women aged 25–74 years, participants of the National FINRISK 2007 Study, answered a questionnaire inquiring about the ambient temperatures considered to be hot and the upper limit of comfortable temperature, and about heat-related complaints and symptoms. The age trends in threshold temperatures and symptom prevalence were examined in 1-year groups by gender after smoothing with loess regression. The prevalence estimates were also adjusted for age. *Results*. The temperature considered as hot averaged 26°C and the upper limit for thermal comfort was 22°C. Both temperatures declined with age (from 25 to 74 years) by 2–5°C. Approximately 80% of the subjects reported signs or symptoms of heat strain in warm weather, mostly thirst (68%), drying of mouth (43%), impaired endurance (43%), sleep disturbances (32%), flushing of skin (29%), headache (19%), impaired concentration (19%) and strong fatigue (16%). Cardiac and respiratory symptoms were reported by 6% and 7%, respectively, and their prevalence increased up to the age of 75 years. The exception was thirst, whose prevalence declined with age. Most symptoms and complaints were more prevalent in women than men. *Conclusions*. Even though summer heat in this northern area is rarely extreme, a considerable fraction of people suffer from various heat-related general or cardiorespiratory symptoms. Women and the oldest were identified as the most vulnerable groups. Information on these is an aid in assessing the burden of summer heat on population health and is a prerequisite for any rational planning of pre-emptive measures.

## F. Women’s Health and Well-being

### Girls’ well-being in northern Finland: promoting and hindering factors

#### Varpu Wiens, Helvi Kyngäs and Tarja Pölkki
University of Oulu, Oulu, Finland, varpu.wiens@gmail.com

##### 

*Background*. Results from previous studies indicate that gender and living conditions have an impact on health and well-being. Girls present different and to some extent more symptoms than boys, and seem to perceive their health as poorer than boys. Some aspects of the living conditions in northern Finland are demanding; for example, other studies have found that seasonal affective disorders are more common in females than males. *Objectives*. The aim of this study was to describe the factors that promote or hinder girls’ well-being in northern Finland. This study is part of a research project entitled Special Issues of Child and Family Well-being of the University of Oulu, Institute of Health Sciences. *Ethical issues*. Approvals were granted by the Northern Ostrobothnia Hospital District Ethics Committee and written consent was obtained from the girls themselves and their parents. During the research, attention was paid to human dignity, which includes respondents’ consent, voluntariness and anonymity, as well as confidence in the ability to understand the study questions. *Design*. A qualitative descriptive study approach was chosen to obtain descriptions of the girls’ perceived factors promoting and hindering their well-being. The 117 girls who participated in this study were living in northern Finland and were aged between 13 and 16 years. Data were collected online with open-end questions, which the girls answered during the school day by computer. Inductive content analysis was employed. *Results*. Factors hindering girls’ well-being were related to the experience of illness, negative social relationships and the girls’ own negative feelings and sensations about life and its circumstances. Well-being was promoted by social relationships that were perceived as supportive and by overall positive feelings towards life. More detailed results of the study will be reported at the conference.

**References**

1. Sourander A, Koskelainen M, Helenius H. Mood, latitude, and seasonality among adolescents. J Am Acad Child Adolesc Psychiatry. 1999;38:1271–6.

2. Saarijärvi S, Lauerma H, Helenius H, Saarilehto S. Seasonal affective disorders among rural Finns and Lapps. Acta Psychiatr Scand. 1999;99:95–101. doi: http://dx.doi.org/10.1111/j.1600-0447.1999.tb07206.x

3. Cavallo F et al, Girls growing through adolescence have a higher risk of poor health. Qual Life Res. 2006;15:1577–85.

## Violence as a part of family relationships – a multigenerational perspective

### Anu Kangas
University of Oulu, Oulu, Finland, anu.kangas@oulu.fi

#### 

The prevalence rates of intimate partner violence in Finland are higher than the European average (1). Previous research indicates that intimate partner violence is often accompanied with child maltreatment (2). Experiencing and witnessing violence in the childhood family has been found to increase the risk for later violent behaviour or victimization, but there is still a need for further exploration of the pathways and contributing factors as not all who experience or witness violence in their childhood family become a perpetrator or victim of violence in later life (3). This qualitative study approaches domestic violence on a family level. The aim is to explore multigenerational family relationships in northern Finnish families where there has been violence. The material consists of in-depth thematic interviews both with women who have experienced intimate partner violence and in some cases also with their family member in the previous or next generation (mother–daughter). In the interviews, women were asked about their experiences and relationships with their family members in current and past generations, and their life progression from childhood to present day. Most of the interviewed women had experienced and/or witnessed violence in their families of origin, including emotional abuse and neglect. Most of them were also aware that there is an intergenerational continuum of abuse in their families. This research sheds some light on complexities of living in a family with violence (as a child and as an adult) and its often far-reaching consequences, as well as on the difficult processes of change both on the individual and intergenerational level in northern Finnish context. The results may be used to better understand some of the aspects of how a family history of violence and relationships with the members of the family of origin may contribute to the progression of later intimate and family relationships that include violence.

**References**

1. Violence against women: an EU-wide survey. Main results report. FRA European Union Agency for Fundamental Rights. (Luxembourg: Publications Office of the European Union, 2014), http://fra.europa.eu/sites/default/files/fra-2014-vaw-survey-main-results-apr14_en.pdf

2. Daro D, Edleson JL, Pinderhughes H. Finding common ground in the study of child maltreatment, youth violence, and adult domestic violence. J Interpers Violence. 2004;19:282–98. doi: http://dx.doi.org/10.1177/0886260503261151

3. Kim K. The role of culture in theories of the intergenerational transmission of violence. Child Fam Soc Work. 2012;17:395–405. doi: http://dx.doi.org/10.1111/j.1365-2206.2011.00793.x

## G. Community-Driven Research

### Indigenous community food security in Yukon territory

#### Norma Kassi, Jody Butler Walker, Katelyn Friendship and Marilyn Van Bibber
Arctic Institute of Community-Based Research, Whitehorse, Canada, onnexi@aicbr.ca

##### 

With changing climate and environmental conditions and increasing costs for food, food security is of increasing concern in Yukon, Canada. Indeed, Yukon First Nations’ Elders have been advising their communities for some time that hard times are coming and that it is time to plan for long-term changes related to food security. To that end, the Arctic Institute of Community-Based Research has been working in partnership with communities to develop locally based food security strategies. Our presentation will focus on the community-based approach we follow to engage with indigenous communities in a respectful and ethically responsible manner. Youth participation and capacity building, culturally appropriate research design and methods, elder participation, traditional knowledge, community research capacity development and community engagement are some examples of our methods. It is evident that for long-term food sustainability and security, communities want clear plans that they can build from, which include being more self-sufficient by increasing local food production, building community gardens, increasing animal husbandry, building microenterprises and returning to ancient methods of sharing and wildlife management. Following a community-based approach has resulted in tangible food security strategies tailored to meet the needs of each community circumstances and cultural heritage.

## Hearing loss in the Kivalliq: a distinctive pattern of hearing loss in an extended family

### Laura Sutherland, Lindsay Du Val and Heather Schilling
University of Manitoba, Winnipeg, Canada, linduval@mymts.net

#### 

Hearing loss (HL) is one of the most common complaints in Nunavut. Although inherited, sensorineural HL is uncommon, it is typically severe and individuals at risk can be identified by a pedigree. As access to audiologic testing is limited in the North, early identification of affected individuals through a pedigree is beneficial. We have followed a family in the Kivalliq region of Nunavut that presents in their late teens with a non-syndromic, non-congenital, mild high-frequency sensorineural HL that progresses within a few years to a moderately severe loss affecting all frequencies. This unusual, distinct pattern of HL points to a genetic aetiology – commonly a mutation in the GJB2 gene. Although this is typically autosomal recessive, there have been reports of autosomal dominant (AD) inheritance, which tend to present post-lingually with subsequent progression of the HL. Of interest to our work is a French family with a similar late onset non-syndromic HL caused by an AD mutation in GJB2. This poster represents a pedigree that we compiled over 25 years and illustrates an extended family including 16 affected individuals across 5 generations and 3 communities. There are 21 additional untested individuals at risk for HL. The atypical age of presentation, unusual configuration and progression of the HL, geographic separation and pedigree would argue in favour of a genetic cause. Future work will involve completing audiologic testing on suspected at-risk individuals in the pedigree. We hope to perform genetic testing at the GJB2 locus to determine if this is a known or de novo mutation, but a mutation at a different or unidentified locus is possible. Finding a common causative mutation in this pedigree could have implications for the molecular basis of hearing loss. Whether this mutation is unique to the Inuit or not, the results may lead us to search for ancestral ties between individuals with the same genetic mutation in other circumpolar populations.

**References**

1. Homøe P, Koch A, Rendtorff ND, Lodahl M, Andersen T, Andersen S, et al. GJB2 (Connexin-26) mutations are not frequent among hearing impaired patients in East Greenland. Int J Audiol. 51:433–6. doi: http://dx.doi.org/10.3109/14992027.2012.660575

2. Kenneson A, Braun KVN, Boyle C. GJB2 (ÿonnexion 26) variants and nonsyndromic sensorineural hearing loss: a HuGE review, Genet Med. 2002; 4:258–274. doi: http://dx.doi.org/10.1097/01.GIM.0000020750.60733.CA

3. Morlé L, Bozon M, Alloisio N, Latour P, Vandenberghe A, Plauchu H, et al. A novel C202F mutation in the connexin26 gene (GJB2) associated with autosomal dominant isolated hearing loss. J Med Genet. 37:368–70.

## Influences on quality of life of older adults in Canada’s Northwest Territories: community-based participatory action research

### Pertice Moffitt and Brianne Timpson
Aurora College, Forth Smith, Canada, pmoffitt@auroracollege.nt.ca

#### 

Quality of life and factors that influence quality of life are salient to the health of northerners. Older adults are an increasing demographic in Canada’s Arctic following a similar national trend. The purpose of this presentation is to describe a community-based participatory action (CBPAR) approach that was used to illuminate the quality of life of the older adults. The Northwest Territories (NWT) Seniors’ Society approached researchers at Aurora Research Institute to work together to explore the influences of quality of life on older adults. Through individual interviews, focus groups and town hall meetings, data were collected and analysed. Participants (n = 92) were 50 years of age and older, from communities across the NWT with 40.2% indigenous. Influences on quality of life for older adults are captured under “the good life” and “life’s struggles”. Ten themes were explicated that include social connection and support, being active and independent, traditional living and place, safety and security, pitiful times and transitions, cost of living, health concerns, housing issues, social isolation, and environment and geography. Recommendations provided are included under four headings: advocacy, education, leadership and research. These recommendations will be used by the NWT Seniors’ Society within their strategic plans, lobbying efforts on behalf of seniors, and in collaboration with networks and government officials as they work to improve the livelihood of older adults in the territory.

## Inuit traditional knowledge for adapting to the health effects of climate change

### Joanna Petrasek MacDonald^1^, Ashlee Cunsolo Willox^2^, James Ford^1^, Susan Chatwood^3^, Victoria Edge^4^, Khosrow Farahbakhsh^4^, Chris Furgal^5^, Sherilee Harper^4^, Ian Mauro^6^ and Tristan Pearce^7^
^1^McGill University, Montreal, Canada, joannamacdonald08@gmail.com; ^2^Cape Breton University, Sydney, Canada; ^3^University of Toronto, Toronto, Canada; ^4^University of Guelph, Guelph, Canada; ^5^Trent University, Peterborough, Canada; ^6^University of Winnipeg, Winnipeg, Canada; ^7^University of the Sunshine Coast, Queensland, Australia

#### 

The Inuit Traditional Knowledge for Adapting to the Health Effects of Climate Change (IK-ADAPT) project was launched in May 2012. Funded through the Canadian Institutes for Health Research (CIHR), IK-ADAPT combines scientific research and Inuit traditional knowledge to develop an evidentiary base to inform policy and programming needed to adapt to the health effects of climate change. Working with Canadian Inuit communities in the Inuvialuit Settlement Region, the Northwest Territories, Nunavut and Nunatsiavut, as well as knowledge users at multiple levels, the project is examining ways to preserve, promote and disseminate Inuit knowledge in order to prevent, prepare for and manage the health impacts of climate change. Having just come to the end of its final phase, this presentation provides an overview of the project, shares results from projects conducted under IK-ADAPT and identifies next steps for enhancing the resilience of communities and northern health systems in the light of a rapidly changing climate.

## Everyday life of reindeer herders – a partnership research project

### Snefrid Møllersen^1^, Vigdis Stordahl^1^, Snefrid Tørres^1^ and Inger Marit Eira-Åhren^2^
^1^Finnmark Hospital Trust, Hammerfest, Norway, vigdis.stordahl@finnmarkssykehuset.no; ^2^Norwegian Reindeer Herders’ Association, Kautokeino, Norway

#### 

*Background*. The Norwegian reindeer herders’ association (NRL) had over the years learned that reindeer herders reported more and more strain in their daily work. Increasing encroachment on their gracing land, new laws and regulations, negative media focus were examples of strain that they feared might have an impact on their health and well-being. In 2008, NRL turned to SANKS, The Sami National Center for Mental Health, asking them to carry out research on what kind of stress and strain reindeer herders were experiencing in their everyday life. *Objective*. The objective of the project is to analyse the Sami reindeer herders’ experiences of psychosocial strain in their daily work and possible impact on mental health and quality of life. *Design*. The project is community driven and is carried out as partnership research. Due to lack of research ethic guidelines in Norway that specifically address challenges in research on indigenous groups, ethic guidelines for health research on indigenous peoples from Canada are used. In order to integrate the reindeer herders’ knowledge and evidence-based knowledge, community-based participatory research (CBPR) is used. The researchers and the reindeer herders’ association are working closely throughout the whole research process: project steering, planning, development of a specific questionnaire for reindeer husbandry, implementation, interpretation of the results and knowledge dissemination.

**References**

1. Canadian Institutes of Health Research, Natural Sciences and Engineering Research Council of Canada, and Social Sciences and Humanities Research Council of Canada, Tri-council Policy Statement. Ethical conduct for research involving humans, December 2010. Chapter 9. http://www.pre.ethics.gc.ca/pdf/eng/tcps2/TCPS_2_FINAL_Web.pdf

2. Møllersen S, Eira-Åhren IM, Stordahl V, Tørres G. Everyday life of reindeer herding. Developing a study to investigate factors that may affect mental health in the Sami reindeer herder population of Norway. Int J Circumpolar Health. 2013;72(Suppl 1):1000–1.

## H. Nature and Health

### Backcountry travel emergencies in northern Canada: a case series of media-reported events

#### Stephanie Young^1^, Taha Tabish^2^, Nathaniel Pollock^3^, Katie O’Beirne^1^ and Kue Young^4^
^1^Institute of Circumpolar Health Research, Yellowknife, Canada; ^2^Qaujigiartiit Health Research Centre, Iqaluit, Canada; ^3^Labrador Institute of Memorial University, Happy Valley-Goose Bay, Canada, nathaniel.pollock@med.mun.ca; ^4^University of Alberta, Edmonton, Canada

##### 

*Introduction*. Rural and indigenous populations in northern Canada regularly travel in backcountry areas where they have limited telecommunications and emergency access. This travel is necessary for employment, hunting, cultural practices, medical care and recreation, and often involves transport by snowmobile or boat. Travel in remote areas can pose risks for injuries and the media commonly reports on such incidents. In this study, we described the extent and characteristics of backcountry travel emergencies. *Methods*. We used a case-series design to examine backcountry travel emergencies in the Northwest Territories (NWT) and Nunavut, Canada, from 2004 to 2013. We identified cases by conducting an online search for articles from two media outlets, Northern News Services and Nunatsiaq News using the terms “rescue,” “missing” and “search.” We searched for cases related to travel that resulted in an emergency event such as a collision or missing person. We used descriptive statistics to examine demographic, environmental and health-related trends. *Results*. Our analyses showed that backcountry travel emergencies are most frequent among males, between the ages of 20 and 39 years. In NWT, most travellers originated in Yellowknife and Inuvik, and events occurred most often in July and August. In Nunavut, travellers often originated in Iqaluit or Baker Lake, and events occurred mainly in November. The key factors that lead to emergencies were accidents in NWT and poor weather in Nunavut. In NWT and Nunavut, we found that 28 and 31% of events, respectively, resulted in the death of at least one group member. *Conclusion*. We showed that backcountry travel emergencies are frequent in northern Canada. They most often occurred among adult men, during the summer and fall, and were often fatal. Northern communities may benefit from improved emergency response infrastructure and public health interventions to increase access to safety gear and survival training.

## Evaluation functional health and well-being among ethnic minority in rural area and urban populations at the Kola North by using the SF-36 test

### Natalia Belisheva^1^, Lena Kim^2^, Poman Mikhailov^1^, Anna Putyatina^2^, Alla Martynova^1^, Sergey Pryanichnikov^1^, Natalia Solov’evskaya^1^, Svetlana Kozlova^1^, Tatyana Zavadskaya^1^ and Janna Kasparyan^3^
^1^Kola Science Centre RAS, Apatity, Russia, natalybelisheva@mail.ru; ^2^Institute Basic and Clinical Research SO RAMS, Moscow, Russia; ^3^Humanitarian Centre, Kola Science Centre RAS, Apatity, Russia

#### 

Purpose of this research is to evaluate the psychometrics of the SF-36 Health Survey among ethnic minority in rural area and urban populations at the Kola North, and to compare the functional health and well-being between different groups of people living in contrast to socio-economic and environmental conditions (Belisheva et al., 2014). Data were derived from the survey of ethnic minority in rural area (Komi Izhemtsy, Sami Nenets people, n=77) and urban respondents (n=280). The urban residents were employed in the production of ore (n=107) and not associated with these activities (n=173). It was found that index of the Physical Component Summary was highest for urban respondents not associated with the production of ore (49.55±9.38); for rural respondents, it was lower (46.65±7.48), and it was lowest for respondents employed in the production of ore (30.22±31.27, p<0.05). Index of the Mental Component Summary was highest for rural respondents (47.98±9.38), it was lower for respondents employed in the production of ore (44.27±10.15) and it was lowest for urban respondents not associated with the production of ore (42.25±11.64). Our preliminary results are evident that objective indicators of physical health based on heart rate variability were lowest for rural residents, so as economic resources. Current well-being has to do with both economic resources and with non-economic aspects of people’s life (what they do and what they can do, how they feel and the natural environment they live in) (Stiglitz, Sen, Fitoussi, 2009). We believe that the way of life and the natural environment of rural respondents are the leading components in their mental well-being. The study was supported by Russian Humanitarian Fund and Administration of Murmansk region, project number 14-16-51003, and by programme of the Presidium of Russian Academy of Sciences in 2014 “Searching basic research for the development of the Arctic zone of the Russian Federation.”

## The value of everyday environment: environmental changes and their influence on human well-being

### Outi Autti and Marjo Tourula
University of Oulu, Oulu, Finland, outi.autti@oulu.fi

#### 

The exploitation of natural resources has various impacts on people. Recently, the numerous ongoing and planned mining projects – hundreds of applications only in Finland – have raised debates on the environmental and social impacts of the project. The discussion mostly considers employment opportunities of local people, pollution and other changes in the environment. The threats of projects around natural resources are mostly seen from the physical health point of view, although the changes can cause wider consequences in the well-being of people affected, such as stress and depression, deteriorating the quality of life. Personal sense of belonging can change along the physical and social changes in the environment. We focus our interest on everyday environment and the meanings of significant places, the importance of their stability and their role in human well-being. Our study is based on an interview data that have been collected along rivers Kemi and Ii in northern Finland. Caused by the electrification, both rivers have gone through dramatic environmental changes. The Kemijoki (length of 550 km, basin area of 51,000 km^2^) was one of the most significant salmon rivers in Europe, and the Iijoki (length of 370 km, basin area of 14,191 km^2^) was one of the most important rivers in Finland. Thematic interviews were conducted in 2009–2010 among 24 elderly, aged between 60 and 91 years old, salmon fishermen living alongside the rivers. The interviewees were asked to recount their experiences concerning the changes they had witnessed in their environment. They were asked about their everyday life and activities before the electrification of the rivers, like fishing migratory fish and the activities that fishing and the use of fish included. The end of salmon fishing caused a crack in the rivermen’s relationship to their environment. The results reveal a strong bond between experiences of well-being and a stable physical, cultural and social environment as well as feeling of belonging.

## Brief historical study of the polar dyspnea

### Dmitrii Tikhonov
North-Eastern Federal University, Yakutsk, Russia, Tikhonov.Dmitri@yandex.ru

#### 

During the first scientific staff travel of Academy of Medicine Sciences of USSR to Dickson Island and Tiksi in 1946, “frequent complaints of breath shortness and pain in the heart, even after minor physical activity, were marked among clinically healthy people.” This phenomenon is called the polar dyspnea. Kandror I. S. writes about this phenomenon: “When there is a strong, especially opposite wind and snow, which close up mouth and eyes, breathing becomes clogged, the person starts to choke and try to roll back to the wind.” Further, the author writes: “… The Soviet polar explorer Kanaka B. indicates that there were cases of deaths during a blizzard not because of freeze, but suffocation caused by respiratory distress.” In the 60–80 years of the last century, “polar dyspnea” was thoroughly studied in Russia. It was called “circumpolar hypoxic syndrome” (1) by a group of Academician Avtsyn A.P., but up to the present time the reason of syndrome occurrence is not clear. Modern researchers have found that the syndrome is connected with bronchial obstruction. It should be noted that in 1940, researchers led by the American physiologist Lawrence Irving discovered reflex of cold breath-holding or diving reflex among human. Subsequently, a number of researchers aroused the diving reflex without dipping his head into the water, but by application of moistened towels with cold water in the area of nasolabial triangle (2). The same phenomenon causes the ingress of water into the face when taking a cold shower. There is an assumption that “polar dyspnea” and diving reflex are related to each other (3). Studies of Grishina and Ustyuzhaninova showed that the development of protective mechanisms in the lungs aimed at eliminating the damaging effect of inhaled cold air to the lung tissue, “restrict ventilation reserves”, which leads to respiratory hypoxia already at an ambient temperature below –25°C. Respiratory minute volume of a healthy person is about 8L/min.

**References**

1. Avtsyn AP, Marachev AG, Matveev LN. Circumpolar hypoxic syndrome. Vestn Akad Med Nauk SSSR. 1979;6:32–9. [Article in Russian.]

2. Speck DF, Bruce DS. Effects of varying thermal and apneic conditions on the human diving reflex. Undersea Biomed Res. 1978;5:9–14.

3. Tikhonov D, Arctic medicine (Yakutsk: Yakutsk Science Centre, Russian Academy of Sciences, 2010), 171–3. [in Russian.]

## Subjective evaluation of health and health-related quality of life of adolescents, inhabitants of the Arctic Yamal

### Victor S. Rukavishnikov, Olga A. Diakovich and Marina P. Diakovich
East-Siberian Institution of Medical and Ecological Research, Russia, marik914@rambler.ru

#### 

There are about 92% of natural gas and 10% of the Russian’s oil reserves in Yamal-Nenets Autonomous Area – one of the most northern regions of Russia. The investigation of health-related quality of life (HRQoL) of the indigenous and the rooting population is important for resolving the issue of sustainable development of the region. Objective was to assess HRQoL and the health risk (HR) in adolescents living in the Yamal. Subjects were adolescents from the district centre at ages 14–17, 58 Nenets from a boarding school and 26 Russian from families. The assessment of the HR (from 0 to 1) was calculated with Bayes’s method. The PedsQL4.0 Generic Core scheme Self-report was used to assess HRQoL with age-appropriate components. The psychological (mood, communication, school), physical (actual health) and total HRQoL components were identified. Adolescents with extremely high-health risk (<0.95) were 31 and 46% among the Nenets and Russian. Nenets had lower risk of hypertension, functional disorders of the respiratory system, neurological disorders and borderline mental disorders. Regardless of ethnicity, risk levels for boys were lower, and risks of digestive system and borderline mental disorders were more prevalent. HRQoL of Nenets was higher compared with Russian as a whole and on the role functioning scale that characterizes the communication. Gender differences in HRQoL were not identified. Nenets had psychological and physical components of HRQoL higher than the Russian. These results can be linked with less secrecy in the presentation of health complaints in adolescents living in families compared with the Nenets, was brought up in a boarding school. Further subjective and objective study of health-related quality of life, social well-being and frustrations of adolescents living in the North to search for the interdependence of these factors is necessary. Studies supported by the programme fundamental researches of Presidium of RAS, the project no. AZ RF-44P.

## I. Educational Programmes

### Innovation in paediatric nursing education: using robotic technology as a pedagogical strategy in rural and remote circumpolar communities

#### Jill MG Bally, Shelley Spurr, Lorna Butler and Alyssa Hayes
College of Nursing, University of Saskatchewan, Saskatoon, Canada, jill.bally@usask.ca

##### 

*Learning objectives*. To describe an innovative research project designed to develop effective paediatric oral health teaching using robotic technology in a third-year clinical nursing practicum. *Background*. Paediatric dental disease is the leading chronic illness in Canada and can result in intense pain and missed school days, and is directly linked to higher incidences of obesity and diabetes. The Caring for Kids Where They Live programme was established in a Canadian circumpolar region using remote presence (RP) robotic technology to provide nursing education to northern and Aboriginal students in their home communities. In this programme, nursing students learned how to conduct overall health assessments including oral health screening and preventive treatment. *Methods*. An exploratory qualitative study will be used to collect data including journal entries, in-depth open-ended interview transcripts and field notes to explore nursing students’ learning experiences when using RP to learn paediatric oral health care. *Summary*. Through robotic technology, nursing students learn with and about other health and education professionals, community members and leaders, families, children and school students as they develop paediatric nursing knowledge and skills. Students will gain the knowledge and skills to develop local solutions and build capacity to address the disparate oral health challenges experienced in remote communities in one Canadian circumpolar region. It creates a pathway by which nursing students in remote areas can learn within an interprofessional setting by connecting clients in rural and remote areas with urban-based dental professionals. Findings will add valuable knowledge to our understanding of the usefulness of RP technology as a learning tool for use in nursing education in remote settings and will serve as a guide to support the restructuring and revision of the oral health component in the third-year paediatric clinical course.

## Wednesday June 10th 2015

### J. Sexually Transmitted Infections (STIs)

#### Safe in the village: developing a sexual health video programme for American Indian/Alaska Native youth in Alaska

##### Cornelia Jessen^1^, Taija Revels^1^, David Driscoll^2^, Janet Johnston^2^ and Sarah Shimer^2^
^1^Alaska Native Tribal Health Consortium, Anchorage, AK, USA, cmjessen@anthc.org; ^2^University of Alaska Anchorage, Anchorage, AK, USA

###### 

American Indian/Alaska Native (AI/AN) youth aged 15–24 in rural Alaska communities experience high rates of sexually transmitted infections (STIs) and have limited access to sexual health programmes, disparities that they share with indigenous youth across the circumpolar north. The Alaska Native Tribal Health Consortium HIV/STD Prevention Program partnered with the University of Alaska Anchorage: Institute for Circumpolar Health Studies to address the need for culturally and age appropriate sexual health programmes for this population. The outcome is Safe in the Village (SITV), a new healthy relationships and safe behaviours video programme. SITV was developed through formative data collection with AI/AN youth (n=97) in rural Alaska communities (n=5) using in-depth interviews and Likert scale surveys to understand perceptions, attitudes and knowledge of HIV/STIs and healthy relationships and associated risk and protective behaviours and factors. In-depth interviews were transcribed and analysed using a grounded theory approach to identify common themes. Surveys were developed based on a cultural consensus approach to ascertain the degree of agreement among participants on key themes. Researchers actively engaged communities by obtaining community support and tribal approvals, employing local site coordinators and involving a community advisory committee to provide guidance and input during the programme development. Sexual health and healthy relationship messages targeted at AI/AN youth need to incorporate traditional values and culturally appropriate conflict resolution while emphasizing protective factors and framing STIs and sex in the context of alcohol abuse and intimate partner violence. A qualitative approach grounded in active community engagement is crucial to developing culturally appropriate and relevant sexual health programmes for AI/AN youth residing in remote Native communities. The SITV programme may also have relevance in circumpolar communities outside Alaska.

## HPV variants in Inuit women from Nunavik, Quebec

### Barbara Gauthier^1^, Francois Coutlée^2^, Eduardo Franco^3^ and Paul Brassard^1^
^1^Department of Epidemiology, Biostatistics, and Occupational Health, McGill University, Montreal, Canada, paul.brassard@mcgill.ca; ^2^Département de Microbiologie et Infectiologie, Centre Hospitalier de l’Université de Montréal, Montreal, Canada; ^3^Division of Cancer Epidemiology, McGill University, Montreal, Canada

#### 

*Background*. Inuit communities in northern Quebec have been shown to have high rates of human papillomavirus (HPV) infection, cervical cancer and cervical cancer-related mortality as compared to the Canadian population. High-risk (HR) HPV can be further classified as intratypic variants which may differ in risk of cervical lesions or neoplasia. There is limited information on which variants are found in circumpolar areas. *Methods*. 629 Inuit women (aged 15–69) from Nunavik had HPV DNA collected along with Pap smear samples between 2002 and 2010 and sequenced to determine the intratypic variant. The different variants and lineages present were described and compared to similar circumpolar populations. *Results*. There were 174 women that were positive for HPV – 16, 18, 31, 33, 35, 45, 52, 56 or 58 during follow-up. There were five different variants, all of which were of European lineage, amongst the 57 women positive for HPV-16. The majority (n=31, 54%) were prototypic. There were eight different variants of HPV-18 present. All were non-prototypic and of European lineage (n=21). There was one woman who tested positive for a prototypic lineage A variant of HPV-31 and 52 women (96%) were from lineage B and one from lineage C (2%). Only one prototypic, lineage A1, HPV-45 variant was present. The rest (n=16) were from lineage B. All women HPV-52 variants (n=23) were in lineage A and 39% were prototypic. One prototype sample was seen for HPV-56 with the majority in lineage A (n=14, 82%). All HPV-58 samples were in lineage A (n=40), and none were prototypic. *Conclusion*. These trends are similar to what was seen in other circumpolar regions of Canada, although there appears to be less diversity as no non-European variants of HPV-16 or 18 were present. This study shows that most variants were clustered in one lineage for each HPV type. We are currently exploring associations of HR-HPV variant with infection persistence.

## Rapid change in the ciprofloxacin-resistance pattern among *Neisseria gonorrhoeae* strains in Nuuk, Greenland

### Anne Rolskov^1^, Karen Bjørn-Mortensen^2^, Gert Mulvad^3,4^, Peter Poulsen^5^, Jørn Skov Jensen^6^ and Michael Lynge Pedersen^3^
^1^Department of Gynaecology and Obstetrics, Hillerød Hospital, Hillerød, Denmark, asrolskov@gmail.com; ^2^Department of Epidemiology Research, Statens Serum Institut, Copenhagen, Denmark; ^3^Queen Ingrid Health Care Center, Nuuk, Greenland; ^4^Greenland Center for Health Research, Institute of Nursing and Health, University of Greenland, Nuuk, Greenland; ^5^The Central Laboratory, Queen Ingrid Hospital, Nuuk, Greenland; ^6^Microbiology and Infection Control, Statens SerumInstitut, Copenhagen, Denmark

#### 

*Objectives*. Sexually transmitted infections (STIs), including infections with *Neisseria gonorrhoeae* (GC), are highly incident in Greenland. Since January 2011, GC testing has been performed on urine with nucleic acid amplification tests (NAATs) by stand displacement amplification (Becton Dickinson ProbeTec). Monitoring of GC antibiotic susceptibility by culture was introduced in Nuuk in 2012. Until 2014, no cases of ciprofloxacin-resistant GC strains were reported. In this paper, we report the finding of ciprofloxacin-resistant GC and describe the most recent incidence of GC infections in Greenland. *Methods*. Men in Nuuk with a positive urine test (NAAT) for GC have been encouraged to provide a urethral swab for culture and susceptibility testing. The number of urine NAATs and culture positive swabs from January to October 2014 were obtained from the Central Laboratory at Queens Ingrid’s Hospital in Nuuk and stratified on gender, place and period of testing. Incidence rates were estimated as number of urine NAAT * (12/10) per 100,000 inhabitants. *Results*. From January to October 2014, a total of 5,436 urine GC NAATs were performed in Nuuk and 9,031 in the rest of Greenland. Of these, 422 (8%) and 820 (9%) were positive, respectively. From January to August, 6 (15%) cultures from Nuuk were ciprofloxacin resistant, while in September and October 26 (59%) were resistant (p<0.01). In total, 35 (40%) of 88 culture-positive isolates showed ciprofloxacin resistance. GC incidence in Nuuk was 3,017 per 100,000 inhabitants per year, while in the rest of Greenland 2,491 per 100,000 inhabitants/year. *Conclusion*. Within a short period, a rapid and dramatic change in ciprofloxacin susceptibility among GC strains isolated in Nuuk was documented and recommendation for first-line treatments has changed. Continued monitoring and re-thinking of primary and secondary preventive initiatives are highly recommended in this high GC incidence setting.

**References**

1. Pedersen ML, Clausen-Dichow P, Poulsen P, Nyborg H, Jensen JS. Low prevalence of ciprofloxacin-resistant *Neisseria gonorrhoeae* in Nuuk, Greenland. Sex Transm Dis. 2013;8:639–40.

2. Statistics Greenland [cited 2014 Nov 13]. Available from: http://www.stat.gl/Bignell C, Unemo M, European STI Guidelines Editorial Board. 2012 European guideline on the diagnosis and treatment of gonorrhoea in adults. Int J STD AIDS. 2013;24:85–92.

## K. Development of Health Care

### Development of a trauma screening and brief intervention process for Alaska Native people in a primary care setting

#### Denise Dillard^1^, Vanessa Hiratsuka^1^, Lisa Dirks^1^, Jaedon Avey^1^, Laurie Moore^2^, Barbara Beach^3^ and Douglas Novins^2^
^1^Southcentral Foundation, Anchorage, AK, USA, vhiratsuka@scf.cc; ^2^University of Colorado Denver, Denver, CO, USA; ^3^Cherokee Nation, Tahlequah, OK, USA

##### 

*Background*. Behavioural health disorders in primary care are common yet are not often recognized and, accordingly, are left untreated. Trauma-related health histories are not regularly completed in primary care settings despite the fact that many people with trauma seek physical, emotional and behavioural health care through primary care. Within this project, we used a community-based participatory research (CBPR) approach to develop a screening, brief intervention and referral process for trauma among Alaska Native/American Indian (AN/AI) adults in two primary care settings: Southcentral Foundation in Anchorage, AK, and Cherokee Nation Heath Services in Tahlequah, OK. In this paper, we will describe the formative research process and findings used to develop a screening and brief intervention for trauma in primary care. *Methods*. A cross-site steering committee was formed to guide the research process. To inform future development of a pilot screening and brief intervention pilot project, we conducted iterative semi-structured interviews and focus groups with customer–owners, clinical providers and administrative leaders in two tribally owned and operated health systems. Data collection processes elicited participants’ views on trauma screening, brief intervention processes and referral for treatment. Data were transcribed and analysed for key themes. *Results*. The most common traumatic experiences mentioned were trauma related to physical or sexual abuse. Respondents reported that trauma services should be provided in a safe, timely, culturally appropriate and community-oriented manner. Preferences for screening for trauma differed by location. Process recommended for brief intervention differed based on available clinical and human resources. *Conclusion*. The process to conduct screening and brief intervention differed at each location due to available resources, particularly immediate access to behavioural health providers.

## Developing a programme evaluation plan of medical rehabilitation services in the Kivalliq Region of Nunavut, Canada; learning from 15 years of community

### Monica Achtemichuk, Anne Durcan, Moni Fricke and Melanie MacKinnon
University of Manitoba, Winnipeg, Canada, monica.achtemichuk@umanitoba.ca

#### 

The University of Manitoba Northern Medical Unit (NMU) has provided medical rehabilitation services to the Kivalliq region of Nunavut, funded by the Government of Nunavut, since 2000. The Kivalliq region has a population of 10,467, the majority of whom self-identify as Inuit. The programme was developed based on the findings of a community needs assessment. The programme provides PT, OT and SLP services that are based in Rankin Inlet, Nunavut. The therapists also provide clinical services to the six widely distributed remote hamlets. Services to Sanikiluaq are provided from therapists based in Winnipeg, Manitoba. A Community Therapy Assistant (CTA) programme was also developed, guided by a feasibility study carried out in 2002, to train local community members to act as rehabilitation assistants in the hamlets across Nunavut. The poster will discuss the elements of the mixed methods programme evaluation that will be conducted. The goals are to evaluate access and quality of care in Rankin Inlet, as well as the hamlets in the region; determine if the current PT, OT, SLP and CTA staffing complement is meeting regional needs; and evaluate the cost-effectiveness of services. A literature review will be conducted on remote medical rehabilitation services and evaluation of such programmes. Partnerships with stakeholders will be identified in the evaluation process and include territorial health authorities as appropriate, health professionals and local health committees. Quantitative data will include statistical programme data as well as survey results from consumers of PT, OT and SLP services. Qualitative data will include discussions with local community members as well as consumers of PT, OT and SLP services. An evaluation plan is an important step to conducting effective programme evaluations. The results will be used to gauge programme effectiveness, address lessons learned regarding administration of the programme and make recommendations on staffing complements and funding.

**References**

1. Developing an Effective Evaluation Plan. Atlanta, GA: Centers for Disease Control and Prevention, National Center for Chronic Disease Prevention and Health Promotion, Office on Smoking and Health; Division of Nutrition, Physical Activity, and Obesity, 2011.

2. Fricke M, Achtemichuk M, Cooper J, Martin B, Macaulay A, Durcan A, Development of a community-based medical rehabilitation programme in the Kivalliq Region of Nunavut, Canada. Int J Circumpolar Health. 2004;63: 101–6.

3. Finlayson M. Assessing need for services. In: G. Kielhofner, editor. Research in occupational therapy: methods of inquiry for enhancing practice. Philadelphia, PA: FA Davis Company; 2006. p. 591–606.

## Supporting remote health care provision in a rapidly changing climate: a case study from Nunatsiavut, Labrador

### Emily Budden^1^, Ashlee Cunsolo Willox^1^, Inez Shiwak^1^, Michele Wood^1^, The IMHACC Team^2^; The Rigolet Inuit Community Government^2^
^1^Cape Breton University, Sydney, Canada, ashlee_cunsolowillox@cbu.ca; ^2^Rigolet Inuit Community Government, Rigolet, Canada

#### 

Anthropogenic climate change has been an increasing concern for Inuit across Canada, and the rapid changes to ice thickness and extent, precipitation levels, weather patterns and wildlife and vegetation dispersion are disrupting livelihoods and lifestyles for many. Evidence is continuing to emerge that these changes are also causing challenges to physical and mental health outcomes throughout the North. Remote healthcare providers and healthcare systems are on the frontlines of these climatic and environmental changes; yet, little is known about the extent of the pressures that will be placed on these systems. Working in partnership with the Inuit Community Governments of Nain, Hopedale, Postville and Makkovik, and the Department of Health and Social Development, the Rigolet Inuit Community Government led a project examining the climatic and environmental determinants of mental health conducted in Nunatsiavut, Labrador, Canada. As part of this research, in-depth conversational interviews were conducted with 18 health professionals throughout all five communities of Nunatsiavut, including nurses, community health workers, social workers and mental health and addictions professionals, to examine challenges related to providing remote health care within the context of a rapidly changing climate. Participants shared that climate change was directly and indirectly affecting the “three Ps” – provision, providers and patients – by amplifying already-present challenges in remote health care and by creating additional challenges related to shifts in climate and environment. This research contributes to growing research in climate-sensitive health adaptation across the North and, if incorporated into health planning at the local and regional levels, may assist in enhancing health care to support climate-sensitive health outcomes in remote communities across the North and enhance healthcare provision.

## Alaska Native breast cancer patients: a care coordination quality improvement project

### Stacy Kelley, Gretchen Day, Christine DeCourtney and Karen Morgan
Alaska Native Tribal Health Consortium, Anchorage, AK, USA, sfkelley@anthc.org

#### 

Breast cancer is the leading cancer among Alaska Native women. When an Alaska Native woman in a village is suspected of having breast cancer, a long journey begins. She must travel by airplane for a screening mammogram, and then again for further tests, diagnosis and treatment. Travel coordination, tribal policies, treatment guidelines, cultural barriers and provider communication create a potential for delays and gaps in the cancer care continuum and all impact diagnosis and timely treatment. To address breast cancer disparities within the Alaska Tribal Healthcare System (ATHS), a collaborative quality improvement study was conducted to identify variances and gaps in the cancer care continuum. We analysed SEER Tumor Registry Data for Alaska Native women at point of breast cancer diagnosis to treatment for years 2009–2014 (N=284) by residence, age, stage of cancer diagnosis and treatment type. We calculated adjusted proportions with logistic regression to examine diagnosis and treatment within programme benchmarks (<60 days). We also identified data measures and gaps within the Electronic Health Record (EHR) and variances within clinical protocols and processes throughout the ATHS through in-depth provider and administrator interviews. Median diagnosis, date to time, of initial treatment for breast cancer was 26 days. No significant rural versus urban differences were found. Additional findings related to the breast cancer data analysis will be presented. Qualitative results of interviews as well as process evaluation results will be presented. This retrospective population-based data analysis suggests that most breast cancer patients are receiving timely care regardless of home community, age and stage of cancer. Treatment for breast cancer patients are well within the established U.S. national core performance indicators and standards. Strengths and challenges of the ATHS will provide recommendations for different models for coordinating care.

## The Nunavut end of life care research project

### Tracey Galloway^1^, Roberta Woodgate^1^, Gwen Healey^2^, Lily Amagoalik^2^, Sharon Edmunds-Potvin^3^, Shylah Elliott^3^, Jennifer Colepaugh^4^, Michelle Doucette Issaluk^4^, Linnea Ingebrigtson^4^, Dawn Stewart^4^, Victor Akande^4^ and Madeleine Cole^4^
^1^University of Manitoba, Winnipeg, Canada, tracey.galloway@umanitoba.ca; ^2^Qaujigiartiit Health Research Centre, Iqaluit, Canada; ^3^Nunavut Tunngavik Incorporated, Iqaluit, Canada; ^4^Government of Nunavut, Iqaluit, Nunavu

#### 

Chronic disease rates are high in Nunavut, as they are in many northern jurisdictions, and patients sometimes die while receiving supportive care, either at home, in long-term care, or in referral centres outside the territory. The purpose of this study is to improve the systems and supports available for Nunavummiut receiving end of life care. End of life narratives from residents of Nunavut (patients, families, community members) will be analysed in the context of existing supports and services and the systems and processes currently in place around end of life decision-making and care. Objectives are to understand the following: Where, when and how do Nunavummiut want to receive end of life care and from what types of formal and informal care providers? What do these care providers need to know about Nunavummiut in order to provide effective end of life care? How can practices around end of life care be shaped to improve the experiences of Nunavummiut? Practical goals include the revision of existing process around advanced care directives; the development of tools and processes used by staff in hospital, long-term care facilities and referral centres; training and support for Home Care and Elder Centre staff who currently provide the majority of formal end of life care in communities; and improved networks of support for informal end of life care providers in families and communities.

## Suicide-related visits to a northern emergency department in Canada

### Nathaniel Pollock^1^, Michael Jong^2^ and Shree Mulay^3^
^1^Labrador Institute, Happy Valley-Goose Bay, Canada, nathaniel.pollock@med.mun.ca; ^2^Labrador Grenfell Regional Health Authority, Happy Valley-Goose Bay, NL, Canada; ^3^ Memorial University of Newfoundland, St. John’s, canada

#### 

*Introduction*. Many northern communities have limited access to mental health services such as outpatient care or culturally safe treatment. As a result, patients may be more likely to delay seeking help until a suicidal crisis. By then, the emergency department (ED) may be the most accessible option, and therefore a common entry point into the health system. In a circumpolar context, the ED may have a critical role to play in initiating interventions for those with suicide-related behaviour. *Methods*. We examined the characteristics and patterns of suicide-related visits to the ED at the Labrador Health Centre. This hospital is located in a large, remote region in northeastern Canada and serves a diverse indigenous population. Our objectives were to determine the incidence rate of suicide-related visits (SRV) and describe the demographic and clinical characteristics of service users. We extracted ED data from the electronic medical record database for 2010 and 2011, and linked this to paper medical charts. We used descriptive statistics to compare SRV rates and proportions by community, sex and age group. *Results*. Our initial analyses revealed that the majority of individuals who visited the ED for suicide-related behaviour were female (57%), less than 40 years old (87%) and from Happy Valley-Goose Bay (39%) or the northern Inuit communities (36%). Over the 2 year period, the rates varied substantially by community, from no visits to 17.1 per 1,000 people. *Conclusion*. Our results showed that more women than men had SRVs, and that they tended to be young adults. We found large differences in the visit rates between communities, and at a regional level the highest rates were in the Inuit communities. Although the cause of the geographic variation is not known, it may be related to local differences in access to services and social support, attitudes towards help seeking or prevalence of risk and protective factors for suicidality.

## Experiences of public services among young people at risk of exclusion

### Piia Rantakokko, Marjo Suhonen and Leena Paasivaara
University of Oulu, Oulu, Finland, piia.rantakokko@oulu.fi

#### 

*Background*. Efficient public services are founded on understanding the actions and needs of service users. Client-centredness means the ability of public service workers to recognise clients’ individual needs on a case-by-case basis. Here, public social and healthcare services are looked at from the viewpoint of young people at risk of exclusion. In this study, young people at risk of exclusion refers to persons under 25 years of age with no upper secondary education or job. This is an important subject to study because prevention of young people’s exclusion and their participation in social activities are important from a human as well as from a national economic viewpoint. *Research question*. What are the views of young people at risk of exclusion on public social and healthcare services? *Data*. The study participants were young people at risk of exclusion (n=10). *Method*. The data were collected with an open interview and analysed with data-driven content analysis. *Results*. The young people’s views on public services included impressions and experiences of the quality of public services that were related to service visits. Quality was associated with accessibility, client-centredness and the professional competence of staff. Based on the impressions the respondents had, public services were not easily accessible to them. The service use behaviour of the young people at risk of exclusion was guided by the impressions they had. For example, the assumption that waiting times are long and of staff acting as gatekeepers had a negative impact on their service-seeking. Staff’s professional ability to provide client-centred services was also seen as deficient. *Conclusion*. In order to respond to young people’s service needs and to prevent exclusion, public services need to be made more client-centred, with more consideration for individual needs. The key here is education of staff to increase their professional competence in terms of client-centredness.

## L. Mental Well-being in the North

### Functional mapping of dynamic happy and fearful facial expressions in young adults with familial risk for psychosis (FR)

#### Johannes Pulkkinen, Juha Nikkinen and Juha Veijola
University of Oulu, Oulu, Finland, johannes.pulkkinen@oulu.fi

##### 

*Background*. Psychotic disorders and their prodromal states have been connected to impaired social functioning. We compared the brain activity between young adults with familial risk for psychosis (FR) and matched controls during visual exposure to emotional facial expression. *Methods*. Fifty-one FR and 52 control subjects were drawn from the northern Finland 1986 birth cohort (Oulu Brain and Mind Study). Participants underwent functional MRI (fMRI) using visual presentation of dynamic happy and fearful facial expressions. FMRI data were processed to produce maps of blood oxygen level dependent (BOLD) responses for happy and fearful facial expressions, which were then compared between groups. *Results*. FR subjects had increased BOLD response in the superior frontal gyrus and supplementary motor area and reduced negative BOLD response in the paracingulate cortex during happy facial expressions. The FR group also showed a statistically significant linear correlation between mean amygdala BOLD response and facial expression recognition. PPI showed that there was a significant negative interaction between the amygdala and the dorsolateral prefrontal cortex (dlPFC) and superior temporal gyrus in FR subjects. *Conclusions*. Our results indicate abnormal function of PFC in FR subjects. This was also suggested by PPI, as the dlPFC showed decreased functional connectivity with the amygdala in the FR group. This may indicate that in FR subjects the amygdala have to take a greater role in emotion recognition and social functioning. This inference was supported by our discovery of statistically significant correlations between the amygdala BOLD response and emotion recognition in the FR group but not in controls.

## Frontline workers’ community response to intimate partner violence in the Northwest Territories, Canada

### Pertice Moffitt, Heather Fikowski, Elizabeth Thompson and Kyla Cherwaty
Aurora College, Inuvik, NWT, Canada, pmoffitt@auroracollege.nt.ca

#### 

Intimate partner violence (IPV) is deleteriously affecting the lives of women and their families in the Northwest Territories. The statistics show a staggering rate of reported violence in the many scattered communities where resources are minimal. The purpose of this presentation is to address the findings, to date, of a 5-year Social Sciences Health Research Council funded project and share implications which further our understanding of IPV in the arctic and subarctic communities of Canada. Utilizing IPV statistics and an environmental scan in the first year of the project, geographical information system maps spatially portrayed the picture of IPV and resources available. These maps helped to design and target communities in years 2 and 3 data collection processes. In year 2, frontline workers (n=31) were interviewed and data were analysed using the constant comparative method of grounded theory. A central phenomenon of hands are tied was identified with social processes of put-up, shut-up and get on with life. This year the model is shared with two communities being profiled in the north. The communities were selected based on the criteria of north/south and resourced/minimally resourced. Narratives are being created in the community profiling using focus groups, document appraisals and purposeful interviews. It is anticipated that through the knowledge generated interventions can be identified to assist health planners develop policies to create and sustain non-violent communities.

## Suicide in the Northwest Territories (1999–2013): a review of the Coroner’s data

### John Omura^1^, Cathy Menard^2^ and Kami Kandola^3^
^1^University of British Columbia, Vancouver, Canada; ^2^Department of Justice, Government of Northwest Territories, Yellowknife, Canada; ^3^Department of Health & Social Service, Government of the Northwest Territories, Yellowknife, Canada, kami_kandola@gov.nt.ca

#### 

*Background*. Suicide is a common cause of mortality in Canada and has reached epidemic levels in regions of the North especially among Inuit populations. This study assesses suicide trends within the Northwest Territories (NWT) and also identifies risk factors associated with suicide as well as opportunities for enhanced surveillance and prevention. *Methods*. From 1999 to 2013, data were collected on all suicide deaths in the NWT by the Office of the Chief Coroner using the NWT Coroner Service Suicide Form (CSSF). After compiling all data into a database, rates were calculated and descriptive analyses performed. *Results*. A total of 125 suicide cases were identified during the study period. Age-standardized suicide rates were 8.0 per 100,000 person-years at risk (PY) for females (95% confidence interval [CI] 4.9–11.2), 30.0 for males (95% CI 23.8–36.2) and 19.4 for males and females combined (95% CI 15.9–22.9). Rates were highest in males aged 14–44 years. While rates remain higher in males than females, the male rate appears to be declining. In terms of seasons, 74 deaths by suicide occurred in the spring and summer compared to 45 in the fall and winter seasons. Ethnic variation was identified, with highest rates among the Inuit population (58.2 suicides per 100,000 PY, 95% CI 40.6–75.8). Frequently identified risk factors included: single status; unemployment; alcohol and drug use; depressed; emotionally distressed; issuing a statement of suicide intent; family breakup or separation; relationship breakup; death of a friend or relative; and extended separation from family due to school, medical and other. *Discussion*. Suicide rates in the NWT are high, particularly among males aged 14–44 years. Specific high-risk populations include the Beaufort-Delta region and the Inuit. These findings, along with several identified risk factors, highlight opportunities for prevention strategies to mitigate the profound impact of suicide in the NWT.

**References**

1. Statistics Canada. 2009 Leading causes of death in Canada – 2009. Available from: http://www.statcan.gc.ca/pub/84-215-x/2012001/tbl/T001-eng.pdf

2. Kirmayer LJ, Brass GM, Holton T, Paul K, Simpson C, Tait C. Suicide among Aboriginal people in Canada. The Aboriginal Healing Foundation.

3. Health Canada. First Nations and Inuit health: suicide prevention; 2013. Available from: http://www.hc-sc.gc.ca/fniah-spnia/promotion/suicide/index-eng.php

## Application of the PEN-3 model on tobacco initiation, use and cessation among American Indian and Alaska Native dults

### Vanessa Hiratsuka^1^, Susan Trinidad^2^, Jaedon Avey^1^ and Renee Robinson^1^
^1^Southcentral Foundation, Anchorage, AK, USA, vhiratsuka@scf.cc; ^2^University of Washington, Seattle, WA, USA

#### 

American Indian (AI) and Alaska Native (AN) communities confront some of the highest rates of tobacco use and its sequelae. As part of a formative research project investigating stakeholder understandings, preferences and needs surrounding the use of pharmacogenetics (PGX) towards tobacco cessation treatment, we sought to characterize sociocultural issues related to tobacco use and cessation. We used the PEN-3 cultural model to frame the research question and analysis of stakeholder interviews with 20 AI/AN patients, 12 healthcare providers and 9 tribal leaders. Our study found high knowledge levels of the negative health effects of tobacco use; however, most patient participants ascribed negative health effects only to regular, heavy tobacco use and not to light use, which is more common in the population. The majority of patient participants did not endorse use of tobacco cessation treatment despite evidence of efficacy among AI/AN adults. Health educators should develop health promotion messaging to target low tobacco consuming AI/AN people and should promote tobacco cessation treatment using successful AI/AN former tobacco users to improve community perception of tobacco cessation treatment.

## Growing up tobacco-free (GUTF) in Alaska: a study to implement tobacco system changes into a Head Start programme in rural Alaska

### Karen Doster, Gary Ferguson and Crystal Meade
Alaska Native Tribal Health Consortium, Anchorage, AK, USA, kldoster@anthc.org

#### 

The Growing up Tobacco-Free in Alaska Project (GUTF) addresses the high level of tobacco use within Head Start families to influence and increase healthy outcomes and educational achievement. The goal of GUTF is to build capacity for the Head Start system to address tobacco or any health risk a family may be facing, while ultimately helping children in Alaska grow up tobacco-free. Using tobacco is the single greatest cause of preventable disease and death in the United States and Alaska. Tobacco use in Alaska contributes to more deaths than all other preventable causes combined. State of Alaska BRFSS statistics show that 38% of low SES non-natives use tobacco, 20% of adults aged between 18 and 29 use tobacco and 36% of Alaska Native people use tobacco, all numbers that represent many of the families that are served by Head Start programs in Alaska. ANTHC worked with the RurAL CAP Head Start programme during a 3-year pilot period to establish a system to address tobacco use through education, resources and training in order to reduce the high prevalence of tobacco use. A model was created that addressed tobacco use through culturally appropriate education and training, evidence-based approaches to tobacco control, provision of easily accessible tobacco cessation services and evaluation to be implemented into RurAL CAP Head Start sites. At the end of the pilot period, 24 RurAL CAP Head Start sites were part of the project. A total of 589 families were surveyed (5,474 individuals). Of the individuals surveyed, there were 227 quit attempts and 63 successful quits. The project proved to be a promising approach to educate Head Start staff and families about the use of tobacco and implement a system within the RurAL CAP Head Start programme to address tobacco use among Head Start families. This approach can be tailored to address all behaviours and health and socioeconomic issues in order to change social norms and improve the life of children growing up in Alaska.

## Sleep disturbances associated with psychosocial factors in female population 25–64 years in Russia/Siberia: MONICA-psychosocial epidemiological study

### Valery Gafarov^1^, Dmitriy Panov^2^, Elena Gromova^2^, Igor Gagulin^1^, Almira Gafarova^1^ and Mikhail Voevoda^3^
^1^Collaborative Laboratory of Epidemiology Cardiovascular Diseases, SB RAMS, Novosibirsk, Russia; ^2^FSBI Institute of Internal and Preventive Medicine SB RAMS, Novosibirsk, Russia; ^3^FSBI Research Institute of Therapy and Preventive Medicine SB RAMS, Novosibirsk, Russia, alex@traveleasy.fi

#### 

*Objective*. To study the prevalence of sleep disturbances (SD) and its relation with other psychosocial factors in female population aged of 25–64 years in Russia (Novosibirsk). *Methods*. Under the third screening of the WHO "MONICA-psychosocial" (MOPSY) programme random representative sample of women aged 25–64 years (n=870) were surveyed in Novosibirsk. The response rate was 72.5%. Estimation of sleep was assessed by the questionnaire Jenkins (1). Chi-square test (χ^2^) was used to assess the statistical significance. *Results*. The prevalence of SD in the female population aged 25–64 years was 65.3%. Poor sleep associates with high personal anxiety more frequently (93.8%) than good sleep (p<0.01). The rate of major depression was fourfold higher in women with poor sleep (p<0.001). Prevalence of high vital exhaustion as well as low close contacts index grows linearly with deteriorating quality of sleep (p<0.001). Changes in marital status are twofold higher and conflicts in family are also increased in women with SD (p<0.05). Those women rarely have the opportunity to relax at home (p<0.05). With regard to job stress poor sleep is associated with stopping or reducing the additional work in two times higher. Women with SD in three times more likely to report decline in their working capacity and responsibility at work (p<0.001). *Conclusions*. The prevalence of SD in female population 25–64 years in Russia is high. SD often related to high personal anxiety and vital exhaustion, major depression, high job and family stress. Supported by Grant of Russian Foundation for Humanities no. 140600227.

**Reference**

1. Gafarov V, Gagulin I, Gromova E, Gafarova A. Sleep disorders in 45–69-year-old population in Russia/Siberia (Epidemiology study). Int J Med Med Sci. 2013;6: 470–5.

## M. Population Health

### The structure of congenital heart defects (CHD) among newborn in the Republic of Sakha (Yakutia)

#### Tuyara Nelunova^1^, Vyacheslav Chasnyk^1^ and Tatyana Burtseva^2^
^1^Saint-Petersburg State Pediatric Medical University, Saint Petersburg, Russia, nelunova-ti@mail.ru; ^2^Yakut Scientific Center, Yakutsk, Russia

##### 

*Aim of research*. To study the structure of congenital heart defects (CHD) among newborn in the Republic of Sakha (Yakutia) on ethnic criteria. *Data and methods of research*. The research was conducted on the basis of the Perinatal Center State Budgetary Institution of the Republic of Sakha (Yakutia) Republican Hospital No. 1 among live-born newborns for the period from 2011 till 2015. CHD were registered according to the nomenclature headings Q20–Q28 (ICD10). Nosological diagnoses CHD were confirmed with data of echocardiography and Doppler sonography of heart and vessels, electrocardiograms, roentgenograms, computed tomograms in angio regimen, and angiographic researches. The indicator of frequency was counted in an amount of 1,000 live-born children. The proportion of the Yakuts in ethnic composition of population is 49.9%, the Russians 37.8%, the Ukranians 2.2%, the Evenks 2.2%, the Evens 1.6%, the Tatars 0.9% and others 5.4% (according to the data of All-Russia Census in 2010). *Results and discussion*. According to our data, there were registered 899 cases of CHD among live-born newborns for the period from 2011 till 2013. In ethnic composition, the proportion of the Yakuts was 72.08% (648 newborn children), the Russians 16.70% (150), the Evenks 4.89% (44), the Evens 1.33% (12), the Yukagirs 0.11% (1), the Dolgans 0.11% (1), the Chukchis 0.11% (1) and others 4.67% (42) among all the cases of CHD (899). The indigenous minorities of the North amounted to 6.56% (59 newborns) in total among all the diagnosed cases of CHD. *Conclusions*. 1) The absolute prevalence of the Yakut children with CHD was elicited: 72.08% ( 648 cases) of all the diagnosed cases of CHD. 2) The Russians are on the second place in frequency, i.e. 16.70% (150 cases). Indigenous minorities of the North amounted to 6.56% (59 cases) which made the third place in frequency. 3) The given data correspond to proportions in ethnic composition according to statistics (based on the results of All-Russia census in 2010).

## Controversies in newborn screening and alternative approaches to prevention of hypoglycaemia for the common Arctic variant (P479L) in CPT1A

### Laura Arbour^1^, Cheryl Greenberg^2^, Graham Sinclair^3^, Sharon Edmunds-Potvin^4^, Charmaine Enns^5^, Hilary Vallance^1^, S. Collins^1^, Bob Thompson^6^ and Jessica Hartley^2^
^1^University of British Columbia, Vancouver, Canada, larbour@uvic.ca; ^2^University of Manitoba, Winnipeg, Canada; ^4^Nunavut Tunngavik Inc, Ottawa, Canada; ^5^Island Health, Victoria, Canada; ^6^Cadham Provincial Laboratory, Winnipeg, Canada

#### 

CPT1a is an enzyme required for the use of fats for energy during fasting. Severe CPT1a deficiency with little or no enzyme activity leads to hypoglycaemia, seizures and death if not treated. In contrast, the P479L variant of CPT1A has reduced but not severely deficient activity but may still lead to hypoglycaemia. The P479L variant is highly prevalent in Inuit, Inuvialuit, Alaska Native and Vancouver Island First Nations populations but not seen in non-indigenous populations. There is evidence that the variant conferred a strong historical advantage during early migration, allowing survival in harsh environments on a high fat, low carbohydrate diet. However, in all areas with a high prevalence of P479L homozygosity, there is also an associated increase in infant mortality (IM). Significant association of the P479L allele and IM caused by SIDS/SUDI and/or infectious disease has been established in all populations studied, but the underlying mechanism is unknown and causation has not been established. There is substantial controversy as to whether newborn screening should be carried out, leading to significant variability in practice. Given the high prevalence of the variant in First Nations of Vancouver Island, but insufficient evidence to support newborn screening, medical guidelines to prevent hypoglycaemia in First Nations infants have been developed to raise the awareness of the possible deleterious effects. Similarly, Manitoba medical specialists covering the Kivalliq region of Nunavut (>70% homozygosity) have also focused on increasing awareness through dissemination of information and education of local health professionals. All Kivalliq newborns born in Winnipeg are screened for hypoglycaemia and select genotyping is performed. Although more research is urgently needed to address or refute the increasing evidence that the variant has a negative impact on infant and child health, these interim approaches to care aim to inform and mitigate potential risk.

**References**

1. Greenberg CR, Dilling LA, Thompson GR, Seargeant LE, Haworth JC, Phillips S, et al. The paradox of the carnitine palmitoyltransferase type Ia P479L variant in Canadian Aboriginal populations. Mol Genet Metab. 2009;96:201–7.

2. Collins SA, Sinclair G, McIntosh S, Bamforth F, Thompson R, Sobol I, et al. Carnitine palmitoyltransferase 1A (CPT1A) P479L prevalence in live newborns in Yukon, Northwest Territories, and Nunavut. Mol Genet Metab. 2010;101:200–4.

3. Sinclair GB, Collins S, Popescu O, McFadden D, Arbour L, Vallance HD. Carnitine palmitoyltransferase I and sudden unexpected infant death in British Columbia First Nations. Pediatrics. 2012;130:1162–9.

## The Yukon CANRISK project: lessons learned in collaborative research

### Brendan Hanley^1^, Ying Jiang^2^ and Gail Peekeekoot^3^
^1^Chief Medical Officer of Health, Yukon, Canada, brendan.hanley@gov.yk.ca; ^2^Public Health Agency of Canada, Ottawa, Canada; ^3^Department of Health and Social Services, Government of Yukon, Whitehorse, Canada

#### 

*Introduction*. Collaboration between Public Health Agency of Canada (PHAC), Government of Yukon, the Office of the Chief Medical Officer of Health (CMOH), the Council of Yukon First Nations (CYFN), Kwanlin Dun First Nation and several rural Yukon communities supported a research project to validate the CANRISK screening tool for young Canadian First Nation adults (20–39 years). Six other Yukon First Nations played, supported and facilitated the research. In follow-up to the research, a “lessons learned” report was compiled by the study author. The lessons learned are particularly relevant for a multi-stakeholder collaboration which saw a project completed within a relatively short time, in a jurisdiction with little formal research infrastructure. Discussion of research lessons learned may be of value to other northern jurisdictions considering research projects. *Methods*. In a separate report to the research results, a “lessons learned” document was prepared by the project coordinator. The report in draft was discussed at a post-project wrap-up meeting where a considerable discussion of the project’s successes and shortcomings was addressed. The “lessons learned” document was finalized and distributed after this meeting. *Results*. Several recommendations to strengthen future collaborative research projects were made and will be discussed. Specific areas of interest include: Administrative requirements: financial coordination, technological and communications infrastructure, and logistical support. Recruitment: Finding the best way to reach a young adult, primarily First Nation audience. Agreements: Establishing written contribution agreements between collaborating bodies. Consent: Mechanisms to ensure adequate informed consent for participants. Follow-up: Mechanisms to ensure adequate follow-up is provided for all participants. Next steps: The lessons learned will be applied to future research and collaborative projects in Yukon.

## Congenital anomalies in the Canadian North 2001–2012: are there east/west differences in rates and type of anomalies?

### Shannon Ryan^1^, Sirisha Asuri^2^, Bryany Denning^3^, Derek Graf^3^, Sharon Edmunds-Potvin^4^ and Michael Jong^5^
^1^Government of Yukon, Whitehorse, Canada; ^2^University of British Columbia, Vancouver, Canada; ^3^Government of NWT, Yellowknife, Canada; ^4^Nunavut Tunngavik Inc., Ottawa, Canada; ^5^Memorial University, St. John’s, Canada

#### 

*Background*. Congenital anomalies (CA) are an important cause of childhood mortality and morbidity in all parts of the world. Socioeconomic, genetic, infectious and environmental factors all influence rates. The northern regions of Canada including Yukon, Northwest Territories, Nunavut and Labrador are inhabited by approximately 142,000 people. Primary care is usually delivered through local health centres, but tertiary care is accessed through southern centres increasing the impact of CAs on families and the cost of health care. *Methods*. CAs reported from discharge abstracts from 2001 to 2012 for stillbirths and live births for the first 30 days of life were compiled into CA categories for each region through the Canadian Congenital Anomalies Surveillance System. Totals and categories were compared per region to those of the rest of Canada using odds ratios (OR) and 95% confidence intervals (CI). *Results*.There were 26,780 births in the North and 3,306,783 South. Total anomalies in Canada, Yukon, NWT, Nunavut and Labrador per 1000 births, respectively, are 54.7, 59.1, 51.3, 85.7 and 65.0. The rate of total CAs are elevated in the North versus. South (OR 1.2; 95% CI 1.15–1.3). Nunavut and Labrador had a significantly higher total rate than the rest of Canada (OR 1.6; 95% CI 1.5–1.7; OR 1.2; 95% CI 1.1–1.3) accounted for by a higher rate of congenital heart and other circulatory anomalies in both regions. Total rates of CAs in Yukon and Northwest Territories were the same or lower when compared to the rest of Canada, partly due to significantly decreased rates of urinary tract anomalies. *Conclusion*. In this evaluation of CAs in the North covering greater than a 10-year span, regional differences emerge. The two more western territories have over-all rates of CA the same or lower than the rest of Canada, whereas the two eastern regions demonstrate increased rates. Congenital heart and other circulatory anomalies in the eastern regions drive the over-all increase in rates of North versus South.

**Acknowledgement**

Michael Jong, Memorial University, Labrador Health Centre, Happy Valley-Goose Bay; We acknowledge the assistance of Jocelyn Rouleau of the Public Health Agency of Canada.

## Patients after percutaneous coronary intervention in Western Siberia: is there a north-south gradient in the prevalence of psychosocial risk factors?

### Georgiy Pushkarev, Vadim Kuznetsov and Elena Yaroslavskaya
Tyumen Cardiology Center, Moscow, Russia, kuznets@cardio.tmn.ru

#### 

*Background*. Our hypothesis was that the prevalence of psychosocial risk factors in patients with coronary artery disease (CAD) after percutaneous coronary intervention (PCI) is higher in the Arctic (north) than in the south regions of Western Siberia. *Purpose*. To compare the prevalence of psychosocial risk factors in two regions of Western Siberia (the north and the south regions) in ischemic patients after PCI. *Methods*. The study included 434 patients with CAD (345 men and 89 women, mean age 58.0±9.1 years) who underwent PCI. Type D Scale (DS14) was used to assess personality type, depressive and anxiety symptoms measured by the Hospital Anxiety and Depression Scale (HADS). Psychosocial stress was investigated with a questionnaire based on the Reeder scale. All patients were divided into two groups according to the region of residence. The first group consisted of 179 patients living in the south regions of Western Siberia. The second group included 255 patients living in the Arctic region of Western Siberia. *Results*. The prevalence of type D personality was not significantly higher in the first group compared to the second (35.8% vs. 27.8%, p=0.08). Clinically relevant depressive and anxiety symptoms were observed in 9.0 and 17.9% patients of the first group and in 9.4% (p=0.90) and 20.4% (p=0.51) patients of the second group, respectively. The prevalence of high level of psychosocial stress was 9.5% in the first group and 13.3% in the second (p=0.22). *Conclusions*. There were no significant differences in the prevalence of psychosocial risk factors between the north and south regions of Western Siberia in patients with CAD after PCI.

## Qualitative research on the experience of cancer in Nunavut, Canada

### Tracey Galloway^1^, Roberta Woodgate^1^, Gwen Healey^2^, Lily Amagoalik^2^, Sharon Edmunds-Potvin^3^, Shylah Elliott^3^, Jennifer Colepaugh^4^, Michelle Doucette Issaluk^4^, Linnea Ingebrigtson^4^, Dawn Stewart^4^, Victor Akande^4^ and Madeleine Cole^4^
^1^University of Manitoba, Winnipeg, Canada, tracey.galloway@umanitoba.ca; ^2^Qaujigiartiit Health Research Centre, Iqaluit, Canada; ^3^Nunavut Tunngavik Incorporated, Ottawa, Canada; ^4^Government of Nunavut, Iqaluit, Canada

#### 

The pattern of cancer incidence and mortality in Nunavut is unique in Canada. Rates of lung, oesophageal and oral-nasopharyngeal cancers are higher than in Canada as a whole. Nunavut is moving from an ad hoc approach to cancer diagnostic and treatment services towards a more coordinated, territory-wide approach to cancer screening and management programmes. Currently, a large proportion of cancer treatment occurs outside the territory in referral centres. This study examines the perspectives of Nunavummiut (patients, families and community members) experiencing cancer: What are the supports/capacities that lead to positive experiences? How can practices be shaped to improve the experiences of Nunavummiut with cancer? The study explores cancer narratives within a context of existing supports, services, medical travel and referral care patterns. The goals are to support territorial efforts to provide culturally-sensitive cancer care and develop/inform tools used by physicians, nurses and support workers in home care, hospitals and long-term care facilities within Nunavut. In addition, the project seeks to enhance the quality and cultural relevance of cancer care delivery in referral care centres in the south. As well, the project aims to share stories of strength, resilience and survival that characterize the particular experiences of people living in Nunavut.

## Characterization of invasive *Haemophilus influenzae* isolated from Nunavut, Canada, 2000–2012

### Tsang Raymond^1^, Rotondo J.A.L.^1^, Mullen A.^2^, Baikie M.^2^, Whyte K.^1^ and Shuel M.^1^
^1^Public Health Agency of Canada, Ottawa, Canada, raymond.tsang@phac-aspc.gc.ca; ^2^Government of Nunavut, Iqaluit, Canada

#### 

*Introduction*. With invasive *Haemophilus influenzae* serotype b (Hib) disease controlled by vaccination with conjugate Hib vaccines, there is concern that invasive disease due to non-serotype b strains may emerge. This study characterized invasive *H. influenzae* (Hi) isolates from Nunavut, Canada, in the post-Hib vaccine era. *Methods*. Invasive Hi isolates were identified by conventional methods at local hospitals and further characterized at the provincial and federal public health laboratories, including detection of serotype antigens and genes, multilocus sequence typing and antibiotics susceptibility. *Results*. Of the 89 invasive Hi cases identified from 2000 to 2012, 75 case isolates were available for study. There were 47 serotype a (Hia), 12 Hib, 2 Hic, 1 Hid, 1 Hie, 2 Hif and 10 were non-typeable (NT). All 47 Hia were biotype II, sequence type (ST)-23. Three related STs were found among the Hib isolates: ST-95 (n=9), ST-635 (n=2) and ST-44 (n=1). Both Hif belonged to ST-124, and the 2 Hic were typed as ST-9. The remaining Hid (ST-1288) and Hie (ST-18) belonged to two separate clones. Of the 10 NT strains, 3 were typed as ST-23 and the remaining 7 isolates each belonged to a unique ST. Eight Hib and one NT-Hi were found to be resistant to ampicillin due to β-lactamase production. No resistance to other antibiotics was detected. *Conclusion*. During the period of 2000–2012, Hia was the predominant serotype causing invasive disease in Nunavut. This presents a public health concern given the severity of illness due to Hia and the lack of published guidelines for the prophylaxis of contacts. The clonal nature of Hia could be the result of spread within an isolated population and/or unique characteristics of this strain to cause invasive disease. Further study of Hia in other populations may provide important information of this emerging pathogen. The only antibiotic resistance detected was to ampicillin due to β-lactamase production and was found mainly in Hib.

## Central haemodynamics and arterial stiffness in northern Russian inhabitants: a comparative study of two populations

### Vladimir Melnikov and Sergey Krivoschekov
Institute of Physiology and Basic Medicine, Novosibirsk, Russia, mevlanic@yandex.ru

#### 

Arterial stiffness is a primary determinant of cardiovascular risk. However, it is still unknown whether the central haemodynamics and blood vessel’s elasticity in northerners differ from those of people living in middle latitudes. The purpose of this study was to compare pulse wave analysis (PWA) parameters obtained with applanation tonometry from two apparently healthy populations living in two regions. Radial artery and aortic PWA testing was performed in winter on 122 persons (mean age 43 years, range 21–64, 53 women) at the north of Kola Peninsula (68°N) and 65 individuals (27 women) of age-matched control population in Novosibirsk city (55°N, south-west Siberia). A partial correlation matrix (BMI and age=const.) between all the studied variables and a stepwise multiple regression were performed. Men and women living in south Siberia have higher levels of blood pressures, augmentation index and lower pulse pressure amplification and thus more stiff arteries compared with their northern counterparts. The correlation analysis found no association between arterial stiffness parameters and length of living in the North after controlling for the influence of age. It is concluded that the conditions of life in Kola peninsula improve the elastic properties of large muscle arteries.

**Acknowledgement**

We are grateful to the Presidium of Russian Academy of Sciences for the financial support of this Arctic project.

## A community-based design process for reaffirming cultural heritage

### Amanda McLeod^1^, Carol Kauppi^1^, Jessica Hein^2^ and Henri Pallard^1^
^1^Laurentian University, Greater Sudbury, Canada, homeless@laurentian.ca; ^2^University of Toronto Scarborough, Toronto, Canada

#### 

A design charrette was conducted with northern indigenous people with a focus on their housing needs as related to their cultural heritage. The research activities were conducted in 2010 and 2011 with Cree people from several First Nation communities on the western James Bay in northern Ontario, Canada. The results of the workshops and the architectural designs that emerged were presented at a conference held in a James Bay community in 2012 as a way to continue the dialogue with Cree people. This community-based project reflects a set of activities that supported cultural affirmation through the use of focus groups combined with a design charrette activity. Using a wide range of materials and drawing, modelling and sculpting techniques, the Cree participants designed their ideal housing. The research process encouraged discussion about memories of their traditional patterns of life, cultural heritage and living circumstances. Participants also discussed the housing issues and challenges they were facing at the time of the study. This poster presentation summarizes the research process and results from the focus groups and from the design charrette, which reveal the strengths of utilizing community-driven research methods. The poster presentation includes images of materials used, the housing models created by the participants and the architectural designs emerging from this project. The traditional forms found within the snowshoe, the canoe, the tipi and the shaapuhtuwaan inspired the creative process and the design of culturally appropriate housing. The designs centred on housing as a response to Cree material culture, social structure and ways of experiencing the land. The research and design process investigate the myriad ways in which the architecture can act as a cultural tool that reaffirms a sense of place and responds to living patterns and the northern climate.

## Examples of health campaigns in Greenland: Max4Tassa (alcohol campaign) and “Seize the Opportunity” (food, smoking, alcohol and exercise – campaign)

### Margit Sander Granlien, Nadja Frederiksen and Ea Cecilie Aidt
Ministry of Health, Government of Greenland, Nuuk, Greenland, masg@nanoq.gl

#### 

*Max4Tassa*. Max4Tassa is a campaign from the Ministry of Health, Government of Greenland. The campaign has two main characters, Heilmann and Rosing, who are officials in the fictitious Department in the Government of Greenland. They need to spread the message that you should maximum drink four units at a time, and that women should drink max 7 and men max 14 units a week. But Heilmann and Rosing have their own difficulties in adhering to the limit. The campaign is built around six TV spots, each of which addresses an alcohol-related problem in a humorous tone. The six TV spots have appeared on national TV and may also be viewed on the Internet: http://www.peqqik.gl/Emner/Kampagner/Max4tassa/Video.aspx?mupid=%7b72E988FC-E3E9-4C71-94DB-BD197F7D4B9E%7d&sc_lang=da-DK. In addition, there have been newspaper ads and posters around in two cities and an active facebook page about Max4Tassa. *“Seize the Opportunity”*.”Seize the Opportunity” is a campaign from PAARISA, Ministry of Health, Government of Greenland. “Seize the Opportunity” consists of several elements. Programmes for TV presentation, different materials for citizen presentation which can be used locally around Greenland, a lifestyle magazine, different teaching materials for public school children and a subside on the official Greenlandic portal for health: http://www.peqqik.gl/Emner/Kampagner/gribmuligheden.aspx?sc_lang=da-DK&mupid=%7b72E988FC-E3E9-4C71-94DB-BD197F7D4B9E%7d The programmes concern health promotion in relation to alcohol/hash, tobacco, nutrition and physical activity. The programmes are made with a mix of exaggerated humour – sketched by a fictive family – the Sussa’s, which have some difficulties living a healthy life, facts and motivation – presented by a Greenlandic psychologist, and Greenlandic cases on how you can make healthy changes in your life in an easy way.

## Distribution of the founder mutation in ATP13A2 gene causing Kufor–Rakeb syndrome (PARK9) in Greenland

### Peder Kern^1^, Inge-Merete Nielsen^2^, Marie-Luise Bisgaard^2^ and Hans Eiberg^2^
^1^Queen Ingrids Hospital, Nuuk, Greenland; ^2^University of Copenhagen, Copenhagen, Denmark, he@sund.ku.dk

#### 

Kufor–Rakeb syndrome (KRS) is a rare autosomal recessive inherited juvenile parkinsonian syndrome caused by a frame-shift mutation in exon 22 in ATP13A2 (c.2473C>AA, p.Leu825AsnfsX32) (1). Disease onset varied from 10 to 29 years of age, the latest reported for KRS, and were found in a family from Atammik (six persons) and a person from Upenavik/Illulisat. Symptoms at onset were asymmetric in three patients, and the clinical features were highly variable within a wide spectrum of an extrapyramidal–pyramidal syndrome with cognitive/psychiatric features in all patients. Ataxia was seen in two patients and electrophysiologically verified axonal neuropathy in one, features not previously related to KRS. Dopamine transporter scans showed symmetrical, severely reduced uptake in striatum in two patients. MRI was without atrophy in one patient despite disease duration of 17 years, and cerebral and cerebellar atrophy was seen in another patient after disease duration of 4 years. None of the eight heterozygous carriers from the family in Atammik have KRS symptoms, suggesting that the mutant protein does not interfere and destroy the function of the wild-type ATP13A2 protein. In the present work, we want to type about 1000 samples from 11 cities in Greenland to calculate the allele frequencies of this founder mutation. Primary studies show that the founder mutation is widely spread in Greenland. At the moment, we have the following results of carriers: Aasiaat 0/102, Nuuk 3/60, Sisimiut 0/17, Tasiilaq 2/44, Maniitsoq 1/3 and Upernaviik 0/58. All the samples are anonymous tested and originating from filter paper for the screening of pregnant women for cholestasis familiaris groenlandica (CFG) and propionic acidaemia (PCCB).

**References**

1. Eiberg H, Hans L, et al. Novel mutation in ATP13A2 widens the spectrum of Kufor-Rakeb syndrome. Clin Genet. 2012;82:256–63.

## Seasonality of clinical symptoms among high-risk families for bipolar disorders in the Arctic

### Sami Pirkola^1^, Heidi Eriksen^1^, Tiina Paunio^2^, Tuula Kieseppä^2^, Timo Partonen^2^, Juha Veijola^3^, Erika Jääskeläinen^3^ and Eeva-Maija Mylläri-Figuerola^4^
^1^University of Tampere, Tampere, Finland, heidi.eriksen@utsjoki.fi; ^2^The National Institute of Health and Welfare, Helsinki, Finland; ^3^University of Oulu, Oulu, Finland; ^4^Hospital District of Lapland, Rovaniemi, Finland

#### 

*Background*. Bipolar disorder (BD) is characterized by periods of manic and depressive behaviour, often precipitated by stressful life-events, substance abuse and sleep-cycle disturbances. Regional variation as well as particularly high penetrance families in Finland exist. These are likely to relate both to genetic and specific environmental factors. Seasonality in bipolarity symptoms is common and circadian, and seasonal rhythms are often disturbed. We explored the clinical characteristics of subjects living in latitudes 68°–70°, with extreme annual variation in daylight. Three groups were studied: 1) 15 subjects with a bipolar spectrum disorder from known high-prevalence pedigrees; 2) 16 healthy family members and 3) 18 healthy non-related controls from the same geographical area. Possible seasonal fluctuation in mood, distress, sleep, social activity and alcohol consumption were followed up at the four most demarcated photoperiodic time points of a year. Groups 1 and 2 represented the indigenous population on the northern latitudes, the Sámi people, who have settled in the area for 8,000–10,000 years. *Data*. The diagnostic SCID interview and the data from traumatic experiences (TSQ), lifetime manic behaviour (MDQ) and self-reported seasonality factors (SPAQ1 and SPAQ2) were collected in the baseline, and self-reported depressive symptoms (BDI), psychic distress (GHQ-12), sleep duration, alcohol consumption (g/week) and vitamin D levels at the four time points. *Results*. The baseline: The affected had the highest MDQ, SPQ1 and TSQ sums. The follow-up: There was variation in measures both within and between the groups. In individual statistical significance testing, the affected scored higher than healthy relatives in winter and spring GHQ12 (4.00 vs. 1.25 and 3.78 vs. 1.08) and BDI (11.88 vs. 4.50 and 11.20 vs. 5.17), but not in summer and autumn. Summer vitamin D –levels were the highest among the affected subjects. *Discussion*. The baseline differences validate the study groups and support seasonal patterns in bipolarity. Seasonal variation in follow-up was observed in affective and distress symptoms, although the sample is relatively small for statistical significances. Individual findings will be explored in further genetic analyses.

**References**

1. Craddock N, Sklar P. Genetics of bipolar disorder. Lancet. 2013;381:1654–62.

2. Wang B, Chen D. Evidence for seasonal mania: a review. J Psychiatr Pract. 2013;19:301–8.

3. Akhter A, Fiedorowicz JG, Zhang T, et al. Seasonal variation of manic and depressive symptoms in bipolar disorder. Bipolar Disord. 2013.

## N. Disease Profiles in the Changing Climate

### Tularaemia mapping in northernmost Sweden: seroprevalence and a case–control study of risk factors

#### Maria Furberg and Johansson Anders
Umea University, Umeå, Sweden, maria.furberg@climi.umu.se

##### 

*Background*. Sweden has one of the highest incidences of tularaemia type B in the world, with major local variations. Our recent study has shown a tenfold increase in Sweden between the periods of 1984–1998 and 1999–2012 (1), with the highest incidences in the north. What proportion of the population in Sweden that has had tularaemia is unknown, and it is still uncertain what life activities are associated with high risk of being infected. Tularaemia creates lifelong immunity and antibodies are detectable decades after the infection. No modern studies of tularaemia seroprevalence exists in Sweden, and the true number of cases may be larger than what is suggested by the disease notifications. Tularaemia is considered a climate sensitive infection and it is important to establish baseline data for monitoring changes over time. *Method*. We are analysing 1,613 serums from a random population sample from Norrbotten (BD) and Västerbotten (AC) counties. The serum donors have answered questions about suspected tularaemia contraction risk factors. We will also investigate if it is possible to correlate seroprevalence in different areas with incidence figures calculated using disease surveillance data (1). In addition, we will analyse risk factors of tularaemia using a case–control study design with questionnaires sent out after an outbreak in 2012 in BD and AC counties. Potential risk factors investigated include time spent on various outdoor activities, housing and the residence distance to water. The study includes 195 unique cases and controls. *Results*. The results will describe which areas and what activities in northern Sweden that carries the highest risk of contracting tularaemia and also give an idea about to what extent people get sick without seeking medical care. This study is part of a larger project aiming at creating a tularaemia early warning system. The data have been collected and are at present being analysed. Preliminary results will be presented at the conference.

**Reference**

1. Amélie D, Maria F, Marika H, Linda V, Anders S, Patrik R, et al. Epidemiology and ecology of tularemia in Sweden, 1984–2012. Emerg Infect Dis. 2015;21. doi: http://dx.doi.org/10.3201/eid2101.140916

## Cardiovascular risk factors in patients with diabetes mellitus and coronary artery disease in Western Siberia: north-south difference

### Vadim Kuznetsov, Elena Yaroslavskaya, Grigoriy Kolunin, Georgiy Pushkarev, Elena Gorbatenko and Luiza Marinskikh
Tyumen Cardiology Center, Moscow, Russia, kuznets@cardio.tmn.ru

#### 

*Background*. The north-south gradient of cardiovascular risk factors exists in many countries. We hypothesized that the north-south gradient could be observed in patients with coronary artery disease (CAD) and diabetes mellitus (DM) in Western Siberia. *Aim*. To compare cardiovascular risk factors in patients with CAD and DM in the North and the South of Tyumen region (Western Siberia). *Methods*. The study included 512 patients with type 2 DM selected from 8,875 consecutive patients with angiographically proven CAD (>50% stenosis). 384 patients were permanent residents of the north of Tyumen region (north of latitude 64°N) (group 1), and 128 patients were permanent residents of the south of Tyumen region (south of latitude 57°N) (group 2). Cases of acute coronary syndrome were excluded. *Results*. Patients of group 1 were significantly younger compared to group 2 (53.9±6.7 vs. 58.1±7.2 years, p<0.001). The smoking and alcohol consumption were higher in group 1 (58.9% vs. 30.0%, p<0.001 and 29.3% vs. 10.5%, p=0.001, respectively). Most of patients in both groups had arterial hypertension (90.4 and 96.1%) and obesity (85.9 and 80.4%). The level of plasma total cholesterol did not differ significantly between the groups (5.5±1.5 and 5.6±1.5 mmol/L). The rate of patients with three CAD risk factors or more was significantly higher in patients of group 1 (34.3% vs. 19.0%, p<0.001). The clinical manifestations of CAD were not significantly different between the groups. Multivariate analysis showed the independent associations of young age and smoking with residence in high latitudes. *Conclusion*. The north-south gradient of cardiovascular risk factors was observed in patients with DM and CAD in Western Siberia.

## Identifying the contribution of physical, social and chemical stressors in morbidity and mortality in Yamal with mathematical models

### Natalia Efimova^1^, Aleksandr Gornov^2^, Victor Rukavishnikov^1^, Igor Bychkov^2^, Inna Mylnikova^1^ and Anton Anikin^2^
^1^East-Siberian Institute Medical and Ecological Research, Irkutsk, Russia, medecolab@inbox.ru; ^2^Institute of System Dynamics and Control Theory, Irkutsk, Russia

#### 

The relative contribution of chemical (air pollution), physical (meteorological conditions) and social health stressors to morbidity is the objective of the current study. Incidence of diseases of population in the research area and the degree of intensity of medical and environmental situation of the north areas with the definition of the category of its distress were assessed in terms of the relative risks of morbidity diseases of individual classes and all classes in the dynamics of the data and month periods. The data used in the mathematical models included the monthly number of incidences and mortality rate, mean concentrations of CO, NO_2_, SO_2_, PM total, hazards index (summary of hazards coefficients of air pollutants) and meteorological data (temperature, relative humidity, wind speed, magnetic variations, shortly changes of atmospheric pressures) in Yar-Sale (Yamal area, North Russian). The relations among the data above were studied using operators of Shepard and Gurman-Rosenraukh Models (1,2). Time series prediction is made on the basis of controlled dynamic models (3). Elevated hazards index are the dominant parameters related to incidences, followed by PM total and meteorological parameters. Quantity medical care resources also play a decisive role in the formation of levels affecting public health.The results prediction showed that an increase in pollution 1.7 times leads to an increased morbidity on 40%, an increase in three times financing of medical care to a decrease of 70% in incidences. The major finding of the study lies on the flexibility and the adaptation of the methodological approach for assessing nonlinear problems and specifically the effect of nonlinear parameters. Moreover, the importance of climate parameters is established even when the whole data set is modelled, reflecting the effect of temperature on health of the Yamal population.

**References**

1. Matorova N, Efimova N, Potorochenko N, Rosenrauch D. Assessing methods in child’s sick rate dynamics under exposure of environmental factors. Paper presented at the annual meeting 1st International Symposium of Ecosystem Health & Medicine, Ottawa, Canada, June, 19–23, 1994.

2. Matorova NI, et al. Child health in heavy chemical air pollution. Paper presented at International Conference Scientific and Practical Aspects of Air Quality Management. Air-95. St. Petersburg, Russian, July 7–9, 1995 (in Russian; in English).

3. Aleksandr Gornov. Numerical technologies for optimal control problems, Novosibirsk, Nauka, 2009. (in Russian).

## Arterial hypertension resistance in the North depending on the adaptive type of the organism adaptive reserves mobilization

### Vyacheslav Hasnulin^1,2^, Mikhail Voevoda^1^, Olga Artamonova^1^ and Pavel Hasnulin^1^
^1^FSBI Research Institute of Therapy and Preventive Medicine SB RAMS, Novosibirsk, Russia, alex@traveleasy.fi; ^2^Siberian Institute of Management, Branch of Russian Presidential Academy of National Economy and Public Administration, Novosibirsk, Russia

#### 

It is found that non-indigenous inhabitants of the North with the hyporeactive adaptive type of an organism adaptive reserves efficient mobilization in the chronic northern stress conditions (“stayers”), showing a higher resistance to emotional stress, desynchronosis in unusual photoperiodism, high functional possibilities of the liver, exhibit lower risks of arterial hypertension as well as associated pathologies development and progression while living in the extreme conditions. Hyper-reactive persons (“sprinters”) proved to be less resistant to the northern stress and, respectively, to the arterial hypertension formation; they also have a higher level of psychoemotional stress, a higher degree of dysadaptation manifestations, reliable decrease in recovery potential, mental capacity reduction and an increase in desynchronoses and hepatocellular dysfunction. The notion that the individuals with ”stayer” adaptive type have gene and phenotypically fixed mechanisms for more effective regulation of the circulatory system in extreme climatic conditions in the North, which reduce the risk of arterial hypertension, is now making its appearance. The possibility of stimulation of recovery processes and blood pressure correction and desynchronoses in the Arctic inhabitants with the arterial hypertension at the health resorts in middle latitudes is considered.

## O. Health Monitoring

### Creating a comprehensive picture of research in the circumpolar Arctic

#### Christy Garrett
Alaska Medical Library, University of Alaska, Anchorage, AK, USA, cgarrett@uaa.alaska.edu

##### 

Research is a key component of good evidence-based public health; knowledge of what research is being done can make for a more informed process. Towards this end, the National Library of Medicine (NLM) in conjunction with the Alaska Medical Library has begun a project to ultimately establish data sharing partnerships, secure depositories and create a comprehensive record of research in Alaska and eventually throughout the circumpolar Arctic. As part of the project NLM will be seeking collaborations with organizations, researchers and community representatives in order to create an easily accessible database where researchers, public health professionals and others can go to see what type of research is being done, by whom and where, to facilitate wider sharing and collaboration, eventually creating a more comprehensive picture of the health issues being studied in the Arctic. Additionally, part of this profile will reference where the data and reports are held and whether access might be possible. It is hoped that these collaborations can eventually lead to discussion about data depositories for projects dealing with circumpolar Arctic human health issues with a central research record that is as comprehensive as possible while still respecting the populations involved. Ultimately, the hope is to create a forum for the discussion of where data resides, how it is stored and if it can be accessed, by whom, and whether a centralized depository for the data from projects around the artic might be possible. In year 1, the focus is on researchers and research conducted in Alaska. In future years, the projects will expand to the circumpolar Arctic research community. The researcher profiles will be made available through the Arctic Health Website, a project of NLM and the Alaska Medical Library. This presentation will introduce the project, setting the stage for the future goal of collaborations with researchers throughout the arctic.

## Addressing historical legacies in the assessment of resource development: why it matters for indigenous well-being

### Ben Bradshaw and Jennifer Jones
University of Guelph, Guelph, Canada, jjones14@uoguelph.ca

#### 

Environmental assessment has long struggled to effectively identify and mitigate community and human health impacts associated with resource development, especially in northern, largely indigenous, jurisdictions. This is changing with routine use of assessment mechanisms like Health Impact Assessments (HIA), supplemental permitting rules and Impact and Benefit Agreements (IBAs) between indigenous groups and mine developers. Coincident with this shift in practice is a growth in research that recognizes diverse concepts and complex drivers of indigenous well-being; as a result, it is increasingly common for researchers to speak of the “good life” and to recognize health disparities that are based in experiences with poverty, stress, trauma, cultural erosion and environmental dispossession. Unfortunately, little of this research has come to influence contemporary HIA practices, supplemental permitting rules and the content of IBAs. Hence, missing from these governance and assessment mechanisms is the recognition that indigenous well-being and resource development are complicated by legacies of colonialism and assimilation policies. What matters to indigenous communities, and what is captured in an HIA, supplemental permitting rules or an IBA, seldom coincide. Drawing on empirical research that documented indigenous participation in an HIA and the negotiation and implementation of IBAs, this paper calls for the refinement of frameworks governing northern development in order to better understand and respond to the complexities that inform indigenous well-being. Ideas for moving forward current research and practice will be shared and discussed. Environmental assessment has long struggled to effectively identify and mitigate community and human health impacts associated with resource development, especially in Northern, largely indigenous, jurisdictions. This is changing with routine use of assessment mechanisms like Health Impact Assessments (HIA), supplemental permitting rules, and Impact and Benefit Agreements (IBAs) between indigenous groups and mine developers. Coincident with this shift in practice is a growth in research that recognizes diverse concepts and complex drivers of indigenous well-being; as a result, it is increasingly common for researchers to speak of the “good life” and to recognize health disparities that are based on experiences with poverty, stress, trauma, cultural erosion and environmental dispossession. Unfortunately, little of this research has come to influence contemporary HIA practices, supplemental permitting rules and the content of IBAs. Hence, missing from these governance and assessment mechanisms is the recognition that indigenous well-being and resource development are complicated by legacies of colonialism and assimilation policies. What matters to indigenous communities, and what is captured in an HIA, supplemental permitting rules or an IBA, seldom coincide. Drawing on empirical research that documented indigenous participation in an HIA and the negotiation and implementation of IBAs, this paper calls for the refinement of frameworks governing northern development in order to better understand and respond to the complexities that inform indigenous well-being. Ideas for moving forward current research and practice will be shared and discussed.

## Establishment of oral health surveillance in Alaska: use of electronic dental records

### Timothy Thomas^1^, Dane Lenaker^2^, Dana Bruden^3^, Richard Baum^3^ and Tom Hennessy^3^
^1^Alaska Native Tribal Health Consortium, Anchorage, AK, USA, tkthomas@anthc.org; ^2^Yukon Kuskokwim Health Corporation, Bethel, AK, USA; ^3^Arctic Investigation Program, Centers for Disease Control and Prevention, Anchorage, AK, USA

#### 

*Background*. For many American Indian and Alaska Native (AI/AN) communities, the prevalence of dental cavities among children is the highest in the USA A 2008 oral health survey in five rural Alaska communities showed 91% of children aged 4–15 years had cavities. Conducting comprehensive oral surveys is costly and labour intensive. AI/AN dental care in south-western Alaska is provided through the regional tribal health corporation. We explored the use of electronic dental records for surveillance purposes. *Methods*. The regional dental unit maintains three databases that capture the existing condition (e.g. decayed, missing teeth) and treatment provided on- and off-site (e.g. fillings, crowns). We merged data for all children aged ≤6 years seen by dental providers between 2003 and 2011 and determined the accumulated decayed, missing, filled teeth (dmft) score for each child. We provide an average dmft score for children aged 6 years in 2009, 2010 and 2011 seen by the dental system within 2 years and compare dmft scores for communities with/without in-home piped water or a dental health aid therapist (DHAT). *Results*. Between 2009 and 2011, the proportion of children seen by the dental system increased from 58 to 83%; however, there was no change in dmft score (9.6 in 2009, 10.9 in 2011). The 2011 dmft scores in communities with and without in-home piped water were 10.3 and 12.0, respectively (p<0.01). In the 20 DHAT communities, 99% of the children were seen compared to 73% in the 29 non-DHAT communities. dmft scores were lower in DHAT (10.3) versus non-DHAT villages (11.2), (p=0.05). *Conclusions*. Using the electronic dental record, we were able to establish dmft scores for a representative portion of the region’s population of 6 years olds and compare by characteristics of community of residence. Continued surveillance will allow monitoring of trends and evaluation of interventions.

## Is self-rated health associated with clinical measures of health among the Inuit of Greenland and Nunavik?

### Inger Katrine Dahl-Petersen^1^, Marie Baron^2^, Christina Viskum Lytken Larsen^1^, Mylène Riva^2^ and Peter Bjerregaard^1^
^1^National Institute of Public Health, University of Southern Denmark, Copenhagen, Denmark, idp@niph.dk; ^2^Centre de Recherche du CHU de Quebec, Universite Laval, Quebec City, Canada

#### 

*Background*. Self-rated health (SHR) has been found to be an indicator of mortality and morbidity in different populations worldwide and is widely used as a measure of health status in population health surveys. However, few studies have examined SRH in relation to objectives health measures among indigenous populations in the circumpolar area. The objective of this study is to assess if SRH is associated with objective health measures in Nunavik and Greenland and to identify whether associations differ between men and women. *Methods*. Cross-sectional comparable data among adults (18+ years) from the Nunavik Inuit Health Survey (2004) and Inuit Health in Transition Greenland Study (2005–2010) were analysed. SRH was dichotomized into good health (excellent, very good or good health) versus poor health (fair and poor health). Age-adjusted logistic regression models were used to examine the associations between SRH and several clinical health measures: waist circumference, diabetes (impaired fasting glucose), blood pressure and cholesterol. Analyses were stratified by sex and conducted separately for the two surveys. *Results*. In overall regional sample, poor SRH was associated only with impaired fasting glucose among participants from Greenland, and with higher waist circumference among participants from Nunavik. In sex-specific analyses, poor SRH was associated with impaired fasting glucose and with higher waist circumference among women in Greenland, but not among men. In Nunavik, poor SRH was associated with higher waist circumference among men only. *Conclusions*. Associations between SRH and objective health measures provided mixed and sex-specific results. More in-depth analyses are needed to understand the associations between SRH and other health measures with relevance for clinical practice and preventive strategies, and to assess the strength of SRH in predicting mortality among Inuit populations.

## Surveillance of congenital anomalies in Greenland

### Turid B. Skifte^1^ and Flemming Kleist Stenz^2^
^1^The National Board of Health, Nuuk, Greenland, tbs@nanoq.gl; ^2^Chief Medical Officer, Nuuk, Greenland

#### 

*Background*. Congenital anomalies in Greenland are since 1995 reported to the National Board of Health. A population less than 57,000 and ~800 births yearly, only 10–15 cases per year were reported and have not been investigated previously. The reported prevalence is similar to other countries (149 per 10,000 in 2007–11), but paediatricians for years suspected under-reporting. Active case finding revealed several cases, indicating reasonable worry. In 2014, Greenland became an affiliate member of the European registry, Eurocat: a network collecting epidemiological information on anomalies to 1) facilitate early warning of teratogenic exposures, 2) evaluate effectiveness of primary prevention, 3) assess impact in prenatal screening, 4) act as information and resource centre, and 5) provide collaborative network and infrastructure for research. The National Board of Health has accessed relevant software, and anomalies diagnosed during pregnancy, at birth and until 5 years of age was included to obtain complete and valid information. *Objective*. To explore any change of occurrence in congenital anomalies and reveal any patterns, and to compare prevalence with other Arctic areas, where similar initiatives are taken. *Method*. Paperforms reported to the National Board of Health are entered in the database, and a search in the National Patient register will be performed. All cases with relevant ICD10 codes for children ≤5 years of age will be followed up. Background data on child, exposition of mother during pregnancy, parent’s workplace, genetic disposition and anomalies or syndrome will be collected. Data are too few for statistical analysis. *Results*. Results will be presented at the conference. Data from this national surveillance database might reveal areas for further investigation. In future, the register gives long-term data for research and knowledge on congenital anomaly patterns in Greenland that may pinpoint areas for prevention or improved diagnosis.

## Research to action: applying the I-Track surveillance results to improve service provision for people who use drugs in Whitehorse, Yukon, Canada

### Brendan Hanley^1^ and Patricia Bacon^2^
^1^Government of Yukon, Whitehorse, Canada, brendan.hanley@gov.yk.ca; ^2^Blood Ties Four Directions Yukon, Whitehorse, Canada

#### 

*Introduction*. The I-Track survey is part of a national enhanced surveillance system that describes patterns of drug injecting practices, sexual risk behaviour and HIV and hepatitis C testing behaviour among people who use drugs in Canada. Whitehorse was selected as a participating site in Phase 3 of the I-Track survey in 2011–2012 and invited both inhalational and injection drug users to participate. Survey results were both published and presented in 2014 (1,2). Three main findings from the I-Track study were 1) high levels of sharing of drug injection equipment, particularly among newer users and long-term users; 2) high levels of sharing drug injection equipment apart from needles and 3) a need to improve access for women who inject. A communication strategy and targeted programme changes were promptly put into place following the surveillance results. *Communication strategy*. After compiling the results, a three-phase communication strategy was developed, including 1) an easy-to-read pamphlet designed for survey participants presented at an evening drop-in reception; 2) an in-depth technical report for service providers and decision makers and 3) a short report with highlights for the public, media and survey participants. *Programme changes*. In light of the survey findings, programme changes included:

1. A pilot programme using multi-coloured needles to reduce the accidental risk of sharing equipment at sites known to be higher risk for sharing.
2. New simple language materials acquired to encourage safer injecting practices.
3. Stickers saying “not just needles, everything new every time” added to injection kits and posters.
4. One-page summaries of the new Canadian Best Practice harm reduction standards and harm reduction refresher courses introduced to improve provider competence and confidence.
5. Enhanced outreach to women and new users previously identified as under-using services.

**References**

1. Machalek K, Hanley BE, Bacon P. Whitehorse I-Track report: monitoring risk behaviour among people who inject or inhale drugs in Whitehorse, Yukon. Whitehorse: Blood Ties Four Directions Centre; 2014. Available from: http://bloodtiesyukon.files.wordpress.com/2009/10/whitehorse-i-track-report.pdf

2. Machalek K, Hanley BE, Bacon P. HIV and hepatitis C prevalence and risk behavior among people who inject or inhale drugs in Whitehorse, Yukon, Canada. Presented at the 20th International Epidemiology Association World Congress of Epidemiology, Anchorage AK; Alaska, August 17–20, 2014. https://wce.confex.com/wce/2014/webprogram/Paper3408.html

## P. Smart Technology

### Inuktitut Mental Health Glossary for clinicians: an online tool and mobile app

#### Alex Drossos
McMaster University, Hamilton, Canada, drossos@mcmaster.ca

##### 

*Introduction*. Previous efforts to develop a general medical terminology translation glossary and electronic tool from English to Inuktitut (“MedInuktitut”) sparked a particular interest to expand and enhance the section on mental health terminology. This prior work, together with active clinical training in the field of psychiatry, informs the current project. The goals were to 1) learn about and better understand the use of terminology regarding mental health in Inuktitut, 2) publish and disseminate the glossary through a simple to use online tool and mobile app for clinicians at the point of care, and 3) test the use of the terminology and make ongoing additions and changes to reflect more appropriate and locally understandable language. *Methods*. While initiating the current project, it became clear that such “mental health” terminology does not exist in the fullest (and Western) sense in Inuktitut, at least in the same way that it does in English. As a result, ethnographic and linguistic reports and other documents were researched and reviewed, concurrently with reports prepared by the Government of Nunavut, Canada, which has ongoing efforts to generate and standardize medical and health terms. A subset of terms that were deemed the most important and likely to be used by mental health clinicians were then selected to be used in the online tool and mobile app. The design and platform of the electronic media (e.g. the online tool and mobile app) existed from previous efforts as described above; thus, the newly generated Mental Health Glossary was simply added to the previous database. *Conclusions*. Testing the use of the Mental Health Glossary is ongoing and will take some time given the small numbers of mental health clinicians able to test the electronic tools in the field. It is hoped that the continued efforts and updates to the glossary will improve clinician–patient communication and do so in a more culturally appropriate and safe manner.

**References**

1. Kirmayer L, Fletcher C, Corin E, Boothroyd L. Inuit concepts of mental health and illness: an ethnographic study. Montreal: Department of Psychiatry, McGill University; 1997.

2. Evic L, Nesbitt G, Douglas C. (Eds.). Inuktitut for health professionals: a phrasebook. Iqaluit, Canada: Pirurvik Press; 2011.

3. Penney C. Medical glossary. Iqaluit, Canada: Nunavut Arctic College; 1995.

## Are we there yet? What’s freely available on the internet for circumpolar health researchers?

### Janice Linton
University of Manitoba, Winnipeg, Canada, janice.linton@umanitoba.ca

#### 

Circumpolar health research is international, interdisciplinary, distinctive and often based in collaborative partnerships between community, government and academic stakeholders. A search of the interdisciplinary scholarly or peer-reviewed literature was carried out on the topic of human health research in the circumpolar regions, 2004–2014. Analysis was carried out to identify publishing trends for research outputs, with a focus on determining the accessibility of the research to stakeholders outside the academic community. In recent years, funding agencies in several countries have established requirements for researchers to publish results in open access venues freely accessible on the Internet. This study was carried out to see how well this community has been doing in knowledge dissemination. The result of this examination of the literature confirms that the circumpolar research community is meeting the challenge of disseminating research findings accessible to stakeholders and communities as well as to other communities who can benefit from the leading-edge research being carried out and the dissemination of indigenous knowledge. The results of this study can be used by seasoned researchers along with practitioners, policy makers and those new to Arctic research, to identify where to access published research for free, efficiently and effectively, and which journals provide the most fruitful knowledge dissemination. A guide to circumpolar health research is available at http://libguides.lib.umanitoba.ca/circumpolar

## “When crisis hits” – electronic tools for those who are to follow-up on crises, accidents and disasters

### Gro Berntsen, Marianne Larssen, Hans Lander and Ingrid Nesje
Resource Centre on Violence, Traumatic Stress and Suicide Prevention, University Hospital of North Norway, Tromsø, Norway, gro.berntsen2@unn.no

#### 

Resources Center on Violence, Traumatic Stress and Suicide Prevention – northern Norway (RVTS North) is organizationally situated under the University Hospital in Northern Norway (UNN-HF), and their geographical area of responsibility is northern Norway and Svalbard. These areas span, as with other circumpolar areas, over a relatively large geographical area with a population that is comparatively spread out. These conditions have made it necessary to develop alternative methods for the dissemination of knowledge, including utilizing different forms of telemedicine and web-based solutions. In Norway, the municipalities have a legally required responsibility to offer psychosocial follow-up of residents affected by crisis. One has also experienced that in many municipalities, there is a need for a strengthening of the support system’s competence regarding psychosocial follow-up of this group. The acts of terror in Oslo and at Utøya on July 22, 2011, actualized the need for a strengthening of both the national and the regional emergency preparedness. In 2011, RVTS North initiated cooperation with Norwegian Centre for Integrated Care and Telemedicine (NST), to develop web-based methods for competence enhancement in order to reach relevant professional groups within the northern region. An e-learning course (electronic learning) has been developed, called “When crisis hits”, which has professionals working with postvention after crisis, accidents and disasters, as its target group. This course has been developed with the aim of enhancing knowledge and competence to relevant professional groups in their meetings with those affected and bereaved after crisis, accidents and disasters. The course is available freely online and can, therefore, be utilized by anyone who is interested. The e-learning course was launched in June 2012, and the response so far shows that the course is both useful and user-friendly.

## Managing chronic kidney disease in northern Manitoba: an innovative model of care using Telehealth and a northern-based nurse clinician

### Kirsten Bourque^1^, Sharon Bruce^2^ and James Zacharias^2^
^1^University of Manitoba, Winnipeg, Canada, kirstenjb_123@hotmail.com; ^2^Department of Community Health Sciences, University of Manitoba, Winnipeg, Canada

#### 

*Purpose*. The purpose of this study is to look at the implementation and effectiveness of a Renal Telehealth Program in northern Manitoba. *Background*. Chronic kidney disease (CKD) is a complex illness that requires regular care by a multidisciplinary team of healthcare professionals (1). In Canada, patients with CKD who live far from a nephrology team have poorer health outcomes than those who live in urban areas (2,3). Nephrologists in Winnipeg, Manitoba, noted a cohort of patients from northern Manitoba who had high "no show" rates with associated complications and co-morbidities when travel to Winnipeg was required for care. In order to address the problems associated with travel, an innovative programme was developed that paired the services of a specially trained renal nurse clinician in Thompson Manitoba with an interdisciplinary nephrology team in Winnipeg. *Methods*. This study will provide a detailed look at the process of developing and implementing the Renal Telehealth Program, including the interventions provided by the nurse clinician. Also, a retrospective analysis of the patient data set with over 100 participants will describe the patient cohort by age, sex and date of enrolment. Health status variables consisting of diabetes, obesity, smoking, hypertension and CKD stage will be included. An assessment of programme effectiveness will look at clinic attendance and adherence to clinical protocols (i.e. blood collected prior to clinic attendance, completion of referrals) and clinical indicator variables at time of enrolment and after 1 year (to look at blood pressure, control of diabetes and progression of CKD). *Results*. It is anticipated that this study shows the interventions provided by the Renal Telehealth Program for CKD patients in northern Manitoba and shows improved clinic attendance and adherence to protocols without compromising control of clinical indicator variables.

**References**

1. KDIGO. KDIGO clinical practice guideline for the evaluation and management of chronic kidney disease. Kidney Int. 2013;3:5–14. doi: http://dx.doi.org/10.1038/kisup.2012.75

2. Miller LM, et al. The association between geographic proximity to a dialysis facility and use of dialysis catheters. BMC Nephrol. 2014;15:1–8. doi: http://dx.doi.org/10.1186/1471-2369-15-40

3. Rucker D, et al. Quality of care and mortality are worse in chronic kidney disease patients living in remote areas. Kidney Int. 2011;79:210–17. doi: http://dx.doi.org/10.1038/ki.2010.376

## “My Word”: storytelling and digital media lab: the evolution of an Inuit-owned digital media and research organization

### Inez Shiwak^1^, Ashlee Cunsolo Willox^2^, Sherilee Harper^3^ and Victoria Edge^3^
^1^The Rigolet Inuit Community Government “My Word”: Storytelling & Digital Media Lab, Rigolet, Canada, inezs@rigolet.ca; ^2^Cape Breton University, Sydney, Canada; ^3^University of Guelph, Guelph, Canada

#### 

Canada’s polar regions are experiencing rapid transformations from climatic and environmental change. These alterations in weather, temperature, snow and ice patterns, and wildlife and vegetation are impacting Canada’s Inuit populations, and negatively impacting their abilities to hunt, fish, forage, trap and travel on the land. These changes are also negatively affecting physical, mental, and emotional health and well-being, as land-based lifestyles and socio-cultural structures are also being disrupted. Inuit communities have been subjected to much climate-related research in the past decade, and there is a wide recognition that this research needs to be community-driven, community-directed and participatory, ensuring that Inuit are leading the process and enhancing and expanding community research capacities. Recognizing this dual need for locally appropriate and culturally relevant adaptation strategies and the development of research capacities in the community, in 2009 the Rigolet Inuit Government in Rigolet, Nunatsiavut, Labrador, undertook an innovative plan to develop the first Inuit-run centre dedicated to digital media and research. Since its inception, the “My Word”: Storytelling and Digital Media Lab has developed expertise in numerous areas: facilitating digital storytelling and participatory video workshops; designing and developing research plans; conducting interviews and surveys; filming, editing and producing videos; consulting with multiple stakeholders for research and adaptation goals and strategies; disseminating information through print and digital media; and presenting at conferences. The “My Word” Lab has also developed particular research capacities for climate-health research and health adaptation strategies. This poster will explain the evolution of the “My Word”: Storytelling and Digital Media Lab and discuss the opportunities and challenges in setting up this type of indigenous-led research and capacity-development organization.

## www.arctichealth.org: an evolving website reflecting a changing Arctic

### Sigrid Brudie
University of Alaska Anchorage, Anchorage, AK, USA, sbrudie@uaa.alaska.edu

#### 

The Arctic Health website is a central source of information on diverse aspects of the Arctic environment and the health of northern peoples. The site was created by the (US) National Library of Medicine (NLM) in 2001, and management of the site was assumed by the Alaska Medical Library (AML). Since the website’s beginnings, NLM and AML have worked together to reshape the site to meet the changing information needs of Arctic researchers and residents. Particularly with changes brought on by climate change, the Arctic is undergoing stress and adaptation in both environmental and human terms, and the Arctic Health website is designed to reflect those changes. For over a decade, librarians and researchers from AML at University of Alaska Anchorage and from NLM’s Outreach and Special Populations Branch have met regularly in person and remotely to discuss and implement changes to the Arctic Health website that will allow users to access burgeoning information on Arctic human and environmental health. In 2013, usability testing was done in the remote Arctic community of Barrow, Alaska, to observe how community members navigated the website and to document their suggestions. With this poster, the hope is to do a unique type of usability testing. The poster will consist of screenshots from the Arctic Health website, with a design that encourages viewers to interact with the poster. There will be “windows” that open to different locations of the website, and the goal is to observe where people look for information on a topic. Unlike conventional website usability testing, the interactive poster will allow casual participation by passers-by, which can provide helpful information on the site’s navigability. Changes continue to be made to the Arctic Health website based on input from users, and the International Congress on Circumpolar Health will provide a valuable opportunity to evaluate the website through the eyes of Arctic researchers.

## More than a funding relationship: government–NGO partnerships for cancer prevention and support in the Northwest Territories

### Sabrina Broadhead^1^, Crystal Milligan^1^, Rosanna Strong^2^ and Andre Corriveau^1^
^1^GNWT-HSS, Yellowknife, Canada, andre_corriveau@gov.nt.ca; ^2^NWT Breast Health/Breast Cancer Action Group, Yellowknife, Canada

#### 

The Government of the Northwest Territories Department of Health and Social Services (GNWT-HSS) is currently working on its first 5-year cancer strategy. In the context of limited funds, human resources and infrastructure, partnerships between government and non-governmental organizations (NGOs) offer innovative, cost-effective solutions to improve the cancer patient experience. The GNWT-HSS is committed to cultivating sustainable partnerships with a variety of groups to leverage resources, improve services and complement existing cancer activities. One such group is the NWT Breast Health/Breast Cancer Action Group (BHBCAG), a non-profit, charitable organization comprising volunteers who promote breast health awareness and support northern women diagnosed with breast cancer. The success of this partnership hinges on shared goals to enhance public awareness and knowledge of cancer, and improve care and support for those affected by cancer. It extends beyond a funding relationship. The two parties meet regularly to exchange information, provide updates and eliminate duplication of efforts. They contribute to one another’s activities as committee/advisory members and working group participants. While the GNWT-HSS provides access to territorial networks and perspective to achieve its goals, the BHBCAG offers specific expertise, skill, credibility and effective access to key target populations. Phase 1 of the BHBCAG NWT Breast Cancer Journeys Project generated evidence that directly informed the development of the new territorial cancer strategy. Phase 2, currently ongoing, will contribute to territorial initiatives in the domains of survivorship care, patient rights and peer support. Government–NGO partnerships formed around common goals, open communication and reciprocal accountability allow for innovation and flexibility, as well as can contribute to both partners’ respective initiatives.

## Looking for (Arctic) indigenous health information? Databases, hedges and international options

### Kathy Murray
University of Alaska Anchorage, Anchorage, AK, USA, kmurray10@uaa.alaska.edu

#### 

*Purpose*. This poster will describe strategies for finding indigenous health information from various resources available in the US, Canada and across the circumpolar north countries. *Setting/participants/resources*. The Alaska Medical Library has worked with the National Library of Medicine to create a website for health information for individuals living in the far north. Work on how to search PubMed to find indigenous health information has been done by librarians in the US and Canada and their discoveries will be shared. *Brief description*. While the website began as a way to improve access to quality health information for Alaska Natives, it has since grown to include publication and research databases, multimedia, climate change and traditional healing. Content has expanded beyond Alaska and now covers the circumpolar north. Finding published and grey literature for indigenous peoples is made more difficult by the lack of good indexing terms in PubMed. This poster will describe: databases from New Mexico to Alberta and across the globe to Norway which should be considered when looking for health information for indigenous groups and others living in the far north, hedges used to pull information from PubMed, and why the Arctic Council should be added to your list of great resources. *Results/outcome*. The growth of this website from local information to both national and international content makes this a good starting point when looking for health information for individuals living in the circumpolar north.

## Q. Living in the Arctic

### Health and housing among indigenous people in northern Canada

#### Carol Kauppi^1^, Jessica Hein^2^, Amanda McLeod^1^, Henri Pallard^1^, Carol Kauppi^1^, Jessica Hein^2^ and Amanda McLeod^1^
^1^Laurentian University, Greater Sudbury, Canada, ckauppi@laurentian.ca; ^2^University of Toronto, Scarborough, Toronto, Canada

##### 

This paper focuses on the health impacts of housing within remote indigenous communities in a subarctic region of Canada – the Western James Bay lowlands. According to UN Special Rapporteur on the Rights of Indigenous Peoples James Anaya (1), the housing circumstances of Inuit and First Nations communities constitute a crisis. He notes that their housing situation contributes to health challenges, particularly in northern communities. This project utilized multiple methods to study housing and health in two Cree communities. The comparison of two remote indigenous communities in the Canadian north make this study unique. In the town of Moosonee, where 85% of the residents are indigenous, a community survey was conducted in 2012 people, as well as a digital storytelling project in which participants narrated issues relating to housing and health. In Fort Albany First Nation, a remote fly-in community on the James Bay coast, a photovoice project was conducted in 2012 in which residents took photographs of their housing situation and explained the impacts on their health. In the results, we identified 1) common themes from the survey data and from photographs and narratives, allowing for a better understanding of the negative impacts of poor housing on physical and mental health; 2) common issues such as “couch surfing” and overcrowding, sleeping rough and substandard or inadequate housing. Furthermore, 3) project’s design enables us to compare the housing circumstances of Cree people living in James Bay communities with those who have migrated from the James Bay to an urban centre in the near north. The findings from this project are similar to those from medical studies on the effects of poverty and housing challenges on health. The implications of the findings will be discussed in terms of the health and housing policies of the Canadian federal government, responsible for funding housing and health in First Nations.

**Reference**

1. UN Human Rights Council. Report of the special rapporteur on the rights indigenous peoples, James Anaya. Addendum: the situation of Indigenous peoples in Canada; 2014. [cited 2015 Jan 9]. Available from: http://daccess-dds-ny.un.org/doc/UNDOC/GEN/G14/075/08/PDF/G1407508.pdf

## Housing interventions in the Arctic: baseline results of a study assessing the impacts of moving to a new house for Inuit health and well-being

### Mylene Riva
Centre de Recherche du CHU de Quebec, Universite Laval, Quebec city, Canada, mylene.riva@crchuq.ulaval.ca

#### 

Across the circumpolar north, a large proportion of Inuit households live in overcrowded conditions. This situation is compromising people’s health and communities’ capacity for social and economic development. Studies have shown that moving to a new house improves health directly and indirectly through psychosocial pathways. To date, few studies have assessed the impacts of housing interventions for health and well-being in the Arctic. Set in Nunavik and Nunavut, this project aims to examine whether moving to a new house – by reducing exposure to overcrowding and improving housing quality – is associated with improved health, and to assess the mediating role of psychosocial factors. Adults in single-person and family households on the waitlist for social housing were recruited to the study. Baseline data were collected 4–6 weeks before people moved into a new house in six communities in Nunavik (October to November 2014) and in three communities in Nunavut (May 2015). Follow-up data collection was conducted 18 months after moving to a house. Results of associations between housing conditions (objective and perceived crowding, housing quality), physical and mental health, and psychosocial factors (control, identity, satisfaction, privacy, safety) were presented for a sample of about 130 participants in Nunavik. Baseline results provided novel evidence pertaining to health and psychosocial factors associated with housing conditions in the Arctic. Overall, results produced by this project have the potential to be integrated in the formulation of housing strategies in Nunavik and Nunavut. Partners: Kativik Municipal Housing Bureau; Nunavik Regional Board of Health and Social Services; Kativik Regional Government; Société d’Habitation du Québec; Nunavut Housing Corporation; Government of Nunavut Department of Health; Nunavut Tunngavik Inc.

## Flood hazard in Kashechewan First Nation: an environmental justice perspective

### Jessica Hein^1^, Carol Kauppi^2^, Arshi Shaikh^3^, Henri Pallard^2^ and Amanda McLeod^2^
^1^University of Toronto Scarborough, Toronto, Canada; ^2^Laurentian University, Greater Sudbury, Canada; ^3^Renison University College, Waterloo, Canada, phm_admin@laurentian.ca

#### 

A key aspect of environmental health for many indigenous communities of the Canadian north pertains to floods and water damage to homes and communities. Floods are costly in terms of damage to property and infrastructure as well as adverse social, psychological and health impacts. Many First Nations face annual flooding and evacuations due to the geographic location of their communities. Kashechewan First Nation, situated in the James Bay lowlands in northern Ontario, has experienced annual flooding over the past several years. This presentation explores the intersecting issues of flooding and displacement in Kashechewan from an environmental justice perspective and considers sustainable, viable long-term policy options to break the annual cycle of flood hazard and its consequences. The environmental justice framework remains largely underutilized for policy development in Canada and this project addressed this knowledge gap. We explored the historical, colonial processes associated with the flooding, the magnitude of the flood hazard, current flood hazard management policies and programmes operating in Kashechewan and policy solutions devised by different jurisdictions within Canada. The environmental justice lens revealed an undercurrent of ongoing environmental colonialism, oppression and racism embedded in the floods encountered by Kashechewan First Nation. In examining contextual factors and policy solutions, we developed viable long-term policy alternatives for flood hazard mitigation by utilizing the environmental justice framework. We recommend flood hazard mitigation policy initiatives that can be integrated with civic engagement and community capacity building, and potentially with broader regional development in the western James Bay area. While the paper focuses on the localized issue of floods, evacuation and displacement for Kashechewan, the recommendations may offer solutions for other First Nations facing similar issues.

## Food security in Alaska: definitions of “urban” and “rural” make a difference

### Tracey Burke
University of Alaska Anchorage, Anchorage, AK, USA, tkburke@uaa.alaska.edu

#### 

Research on food security provides a case study of the ambiguity of the terms rural and urban when referring to some areas of Alaska and Canada. This presentation draws on a qualitative study conducted with urban and rural users of Alaskan food pantries to illustrate how different types of communities are associated with different discourses of food security and possible interventions. The project is part of a long-standing collaboration between the PI and the state’s largest hunger-assistance NGO. Academic and colloquial references to “rural Alaska” often refer to the very-remote, mostly Alaska Native communities found off the road and ferry systems. Yet, Alaska has numerous communities on the road and ferry systems, the residents of which are typically non-Native, and very few of which are conventionally “urban.” There is no sanctioned typology of Alaskan communities; to conduct our study, we developed our own. It is based on the US Department of Agriculture’s “frontier and remote” codes (41) and uses two dimensions – population size and order of services available, and remoteness. Alaska’s three discourses of food security map onto the typology easily. The remoteness dimension reflects favoured local foods. The discourse of food security in remote Alaska focuses on subsistence hunting/fishing. In more accessible communities, the discourse of “locally grown” produce gains prominence. The size/services dimension captures the variation in the response to poverty-related food insecurity. Relatively larger communities, even when remote, are more likely to have more social and health services than small ones. Anticipating how the dominant discourse of food security and the capacity to respond to poverty will vary with the type of community facilitates discussions of food security. The policy and services implications of recognizing nuanced meanings of rural and urban were discussed.

**Reference**

1. Cromartie J, Nulph D, Hart G. Mapping frontier and remote areas in the U.S; 2012. [cited 2013 Aug 1]. Available from: http://www.ers.usda.gov/amber-waves/2012-december/data-feature-mapping-frontier-and-remote-areas-in-the-us.aspx#.UfrDj43CZ8

**Figure:**